# Physiological Studies of *Chlorobiaceae* Suggest that Bacillithiol Derivatives Are the Most Widespread Thiols in Bacteria

**DOI:** 10.1128/mBio.01603-18

**Published:** 2018-11-27

**Authors:** Jennifer Hiras, Sunil V. Sharma, Vidhyavathi Raman, Ryan A. J. Tinson, Miriam Arbach, Dominic F. Rodrigues, Javiera Norambuena, Chris J. Hamilton, Thomas E. Hanson

**Affiliations:** a School of Marine Science and Policy and Delaware Biotechnology Institute, University of Delaware, Newark, Delaware, USA; b School of Pharmacy, University of East Anglia, Norwich, United Kingdom; c Department of Biochemistry and Microbiology, Rutgers University, New Brunswick, New Jersey, USA; d Department of Biological Sciences, University of Delaware, Newark, Delaware, USA; North Carolina State University; Max Planck Institute for Marine Microbiology

**Keywords:** cellular redox status, *Chlorobaculum tepidum*, chlorobiaceae, low molecular weight thiol, sulfur

## Abstract

Low-molecular-weight (LMW) thiols mediate redox homeostasis and the detoxification of chemical stressors. Despite their essential functions, the distribution of LMW thiols across cellular life has not yet been defined. LMW thiols are also thought to play a central role in sulfur oxidation pathways in phototrophic bacteria, including the *Chlorobiaceae*. Here we show that Chlorobaculum tepidum synthesizes a novel LMW thiol with a mass of 412 ± 1 Da corresponding to a molecular formula of C_14_H_24_N_2_O_10_S, which suggests that the new LMW thiol is closely related to bacillithiol (BSH), the major LMW thiol of low-G+C Gram-positive bacteria. The *Cba. tepidum* LMW thiol structure was N-methyl-bacillithiol (N-Me-BSH), methylated on the cysteine nitrogen, the fourth instance of this modification in metabolism. Orthologs of bacillithiol biosynthetic genes in the *Cba. tepidum* genome and the CT1040 gene product, N-Me-BSH synthase, were required for N-Me-BSH synthesis. N-Me-BSH was found in all *Chlorobiaceae* examined as well as *Polaribacter* sp. strain MED152, a member of the *Bacteroidetes*. A comparative genomic analysis indicated that BSH/N-Me-BSH is synthesized not only by members of the *Chlorobiaceae*, *Bacteroidetes*, *Deinococcus-Thermus*, and *Firmicutes* but also by *Acidobacteria*, *Chlamydiae*, *Gemmatimonadetes*, and *Proteobacteria.* Thus, BSH and derivatives appear to be the most broadly distributed LMW thiols in biology.

## Abstract (precis)

Low-molecular-weight thiols are key metabolites that participate in many basic cellular processes: central metabolism, detoxification, and oxidative stress resistance. Here we describe a new thiol, N-methyl-bacillithiol, found in an anaerobic phototrophic bacterium and identify a gene that is responsible for its synthesis from bacillithiol, the main thiol metabolite in many Gram-positive bacteria. We show that the presence or absence of this gene in a sequenced genome accurately predicts thiol content in distantly related bacteria. On the basis of these results, we analyzed genome data and predict that bacillithiol and its derivatives are the most widely distributed thiol metabolites in biology.

## INTRODUCTION

**FIG 1:**
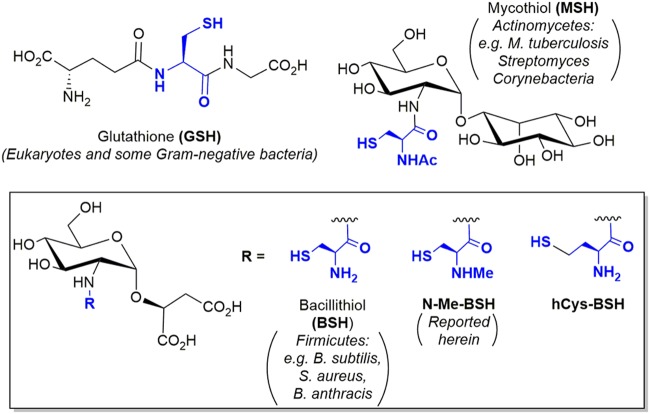
Structures of LMW thiols referred to in the text with the sulfhydryl-bearing motif derived from cysteine or homocysteine colored blue. BSH derivatives are boxed together with the R group on the common backbone indicated. The distributions of previously studied LMW thiols across different organisms are indicated.

In eukaryotes and some Gram-negative bacteria, the cysteine-containing tripeptide glutathione (L-γ-glutamyl-L-cysteinyl-glycine) (GSH) is the major low-molecular-weight (LMW) thiol cofactor (Fig. 1). GSH maintains a reducing intracellular environment, regulates protein function and protects exposed cysteine residues by S-glutathionylation, conjugates electrophilic metabolites/xenobiotics for detoxification, and maintains metal ion homeostasis. GSH is the most extensively studied LMW thiol thus far, but not all prokaryotes produce GSH; it is restricted to the *Cyanobacteria* and certain *Proteobacteria* (1). Gram-positive bacteria produce structurally distinct LMW thiols that serve similar metabolic functions (Fig. 1): mycothiol (MSH) in the *Actinomycetes* (2, 3) and bacillithiol (BSH) in the low-G+C *Firmicutes* (4). Like GSH, BSH detoxifies metabolites/xenobiotics including fosfomycin and methylglyoxal (5–7), maintains metal ion homeostasis (8), and protects/regulates protein function during oxidative stress via reversible S-bacillithiolation catalyzed by bacilliredoxins (9, 10). However, there are still many prokaryotes where a LMW thiol has not yet been identified to carry out these critical functions.

The green sulfur bacteria (the *Chlorobiaceae*) are anaerobic, anoxygenic phototrophs that are found in anoxic water and sediments that contain reduced sulfur compounds and are exposed to light. The *Chlorobiaceae* have contributed to our understanding of CO_2_ fixation via the reductive TCA cycle (11) and light-harvesting mechanisms through studies of the chlorosome (12, 13), the light-harvesting antenna in this group. Chlorobaculum tepidum (formerly Chlorobium tepidum) is a model system for the *Chlorobi* because of its rapid growth rate (14), whole-genome sequence availability (15), and genetic system (16–19).

Chlorobaculum tepidum oxidizes reduced sulfur compounds (sulfide, elemental sulfur, and thiosulfate) to feed electrons into the photosynthetic electron transport chain where they ultimately reduce ferredoxin, which in turn is used to drive CO_2_ fixation (11), N_2_ fixation (20), and the reduction of NAD(P)^+^ (21). LMW thiols have been proposed as a sulfur atom shuttle between the periplasm and cytoplasm to feed sulfide into biosynthetic pathways, the dissimilatory sulfite reductase and ATP-sulfurylase in the *Chlorobiaceae* and other phototrophic sulfur oxidizers (22, 23). This function requires that the LMW thiol cycle between the thiol (R-SH) and perthiol (R-S_n_-SH) forms, as observed for glutathione amide in the purple sulfur bacterium Chromatium gracile (24). However, LMW thiols have not yet been identified in *Cba. tepidum* or other *Chlorobiaceae*. Prior studies suggested the existence of a novel thiol, named U11, in Chlorobium limicola, which also lacked detectable amounts of GSH and other common LMW thiols (25). More recent reports indicate that the genome of Chlorobium limicola encodes enzymes that can synthesize ergothioneine *in vitro* (26).

Here we show that *Cba. tepidum* contains N-methyl-bacillithiol (N-Me-BSH) consisting of BSH modified by N-methylation of the cysteine amino group. Orthologs of BSH biosynthesis genes present in the *Cba. tepidum* genome are required for the synthesis of N-Me-BSH, as is an S-adenosyl-L-methionine (SAM) methyltransferase encoded by CT1040 that performs the methylation of BSH. N-Me-BSH was detected in all members of the *Chlorobiaceae* examined. Orthologs of BSH biosynthesis genes and CT1040 co-occur in the genomes of all *Chlorobiaceae* and extremely diverse members of the *Bacteria*, and the presence or absence of a CT1040 ortholog was shown to accurately predict the LMW thiol content in *Polaribacter* sp. strain MED152 and Thermus thermophilus HB27. The distribution of these genes suggests that BSH and/or N-Me-BSH may be the most widespread LMW thiols in biology.

## RESULTS

### Chlorobaculum tepidum contains a novel LMW thiol.

**FIG 2:**
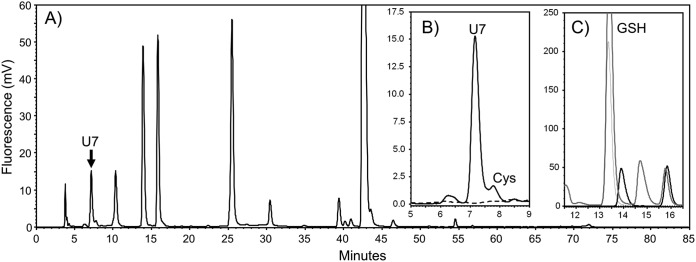
Chlorobaculum tepidum contains a novel LMW thiol. (A) Full HPLC chromatogram of bimane extract from a stationary-phase *Cba. tepidum* culture grown under standard conditions. The arrow indicates the novel thiol U7. (B) U7 and cysteine (Cys) are not detected if extract is treated with N-ethyl-maleimide (dashed line) before mBBr. (C) Glutathione (purified standard, dashed-dotted line) is readily detected in E. coli bimane extract (gray line), but not *Cba. tepidum* (solid line).

LMW thiol compounds in Cba. tepidum were examined by HPLC analysis of S-bimane derivatives produced by simultaneous thiol extraction and treatment with the thiol-selective fluorophore monobromobimane (mBBr). *Cba. tepidum* cells, grown with sulfide plus thiosulfate as electron donors to late exponential phase (approximately 20 µg Bchl *c* ml^−1^), produced one bimane derivative with a unique retention time (Fig. 2A, arrow) relative to standard LMW thiol compounds and reagent blanks (see Fig. S1 in the supplemental material). The bimane derivative, named U7 for its retention time, was not observed in extracts treated with the sulfhydryl blocking agent N-ethylmaleimide (NEM) prior to mBBr derivatization (Fig. 2B, dashed trace). The observed retention time for this compound was unique relative to all authentic standards analyzed here and to those reported in the literature for other LMW thiol compounds (4, 24, 25, 27–29). For example, extraction of Escherichia coli, where glutathione is the predominant LMW thiol (30), yielded an mBBr derivative that comigrated with a glutathione standard and was absent from *Cba. tepidum* extracts (Fig. 2C). This result confirms earlier reports that other members of the *Chlorobiaceae* lack GSH (25).

10.1128/mBio.01603-18.2 FIG S1 Representative HPLC chromatograms of bimane derivatized compounds: Chlorobaculum tepidum stationary-phase extract (A), mixture of authentic standards (B), and reagent blank (C). The retention times of standard compounds are noted with blue dashed lines. The retention times of reagent blank peaks are noted with red dashed lines. The identities of standard bimane derivatives are labeled: cysteine (Cys), thiosulfate (S_2_O_3_^2−^), β-mercaptoethanol (β-ME), sulfide (HS-), and dithiothreitol (DTT). Note that DTT elutes in two distinct peaks. Download FIG S1, PDF file, 0.6 MB. Copyright © 2018 Hiras et al. 2018 Hiras et al. This content is distributed under the terms of the Creative Commons Attribution 4.0 International license.

### U7 is BSH modified by N-methylation of cysteine.

Treating bimane-labeled extracts with tris(2-carboxyethyl)phosphine (TCEP) according to Franz et al. (31) did not change the U7 peak area (Fig. S2), demonstrating that U7 is a monothiol rather than a perthiol. HPLC fractions containing U7 were collected, concentrated, and analyzed by Fourier transform ion cyclotron resonance mass spectrometry (FT-ICR-MS) in positive ion mode, yielding a predominant ion at 603.196 *m/z* for two independent samples (Fig. S3 and Table S1). This is consistent with a mass of 412 Da for the thiol before the addition of the bimane tag (191 Da). DECOMP analysis of the FT-ICR-MS data for monoisotopic element combinations within 2 ppm error and formulas containing at minimum bimane (C_10_H_11_N_2_O_2_), one sulfur atom, and 10 more carbon atoms produced 13 possible formulas. The composition of bimane was replaced with hydrogen to arrive at likely formulas for the original thiol (Table S2, first column). Candidates were further evaluated by comparison to MS/MS data. MS^3^ data on the 469 *m/z* ion indicated that masses observed in MS^2^ of the 603 *m/z* ion were produced by sequential decomposition events: 603 > 469 > 433 > 391 *m/z*. Only one formula, C_14_H_24_N_2_O_10_S, could produce the correct decomposition masses, given the starting formula (Table S2, bold text): U7 > *a*-C_10_H_18_N_2_O_5_S > *b-*C_10_H_14_N_2_O_3_S > *c-*C_8_H_12_N_2_O_2_S. Fragment *a* indicated a loss of malic acid (C_4_H_6_O_5_) from the U7 bimane adduct. Malic acid addition to UDP-N*-*acetylglucosamine (UDP-Glc-NAc) is the first step of BSH biosynthesis (4). This led us to hypothesize that that U7 could be related to BSH.

10.1128/mBio.01603-18.3 FIG S2 Effect of TCEP on mBBr derivatized compounds in *Cba. tepidum* extracts. The solid line is untreated mBBr extract; the dashed line is the same sample after reduction with TCEP. (A) Region surrounding U7; (B) region surrounding sulfide. Bimane derivatives are labeled with their identity determined by cochromatography with authentic standards. Download FIG S2, PDF file, 0.3 MB. Copyright © 2018 Hiras et al. 2018 Hiras et al. This content is distributed under the terms of the Creative Commons Attribution 4.0 International license.

10.1128/mBio.01603-18.4 FIG S3 FT-ICR-MS spectrum of U7mB as purified from *Cba. tepidum* (A) and MS/MS spectrum of the 603.1964 ion after collision-induced dissociation (B). Download FIG S3, PDF file, 0.3 MB. Copyright © 2018 Hiras et al. 2018 Hiras et al. This content is distributed under the terms of the Creative Commons Attribution 4.0 International license.

10.1128/mBio.01603-18.8 TABLE S1 Masses of intact U7mB and CID fragments detected by MS^n^. Download Table S1, PDF file, 0.2 MB. Copyright © 2018 Hiras et al. 2018 Hiras et al. This content is distributed under the terms of the Creative Commons Attribution 4.0 International license.

10.1128/mBio.01603-18.9 TABLE S2 Deduced neutral mass values and associated formulas for FT-ICR-MS and MS/MS fragments with the mass of bimane subtracted. Download Table S2, PDF file, 0.1 MB. Copyright © 2018 Hiras et al. 2018 Hiras et al. This content is distributed under the terms of the Creative Commons Attribution 4.0 International license.

**FIG 3:**
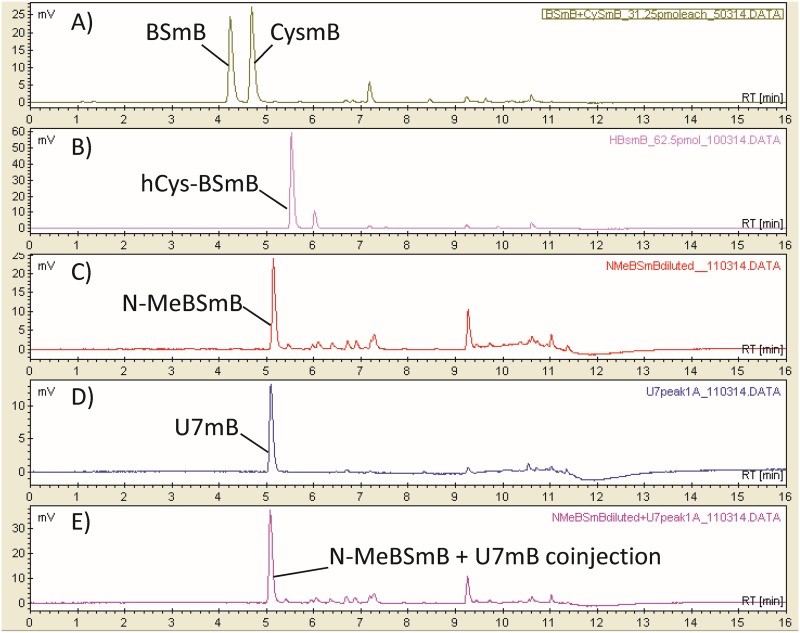
The novel *Cba. tepidum* U7-bimane adduct (U7mB) is N-Me-BSmB. (A to E) HPLC chromatograms of CysmB and synthetic BSmB (A), synthetic hCys-BSmB (B), synthetic N-Me-BSmB (C), purified *Cba. tepidum* U7mB (D), mixture of N-Me-BSmB and purified *Cba. tepidum* U7mB (E). The retention times are different from those shown in Fig. 1, because a different column and elution gradient were used to better separate these compounds.

The deduced mass and formula for U7 differ from those of BSH by an additional methylene unit. Two plausible structures that could account for this are a BSH derivative where the cysteine is replaced with either homocysteine (hCys-BSH) or N-methylcysteine (N-Me-BSH). To address this, S-bimane derivatives of hCys-BSH and N-Me-BSH were chemically synthesized as analytical reference samples for comparison with bimane-labeled U7 by HPLC separation (Text S1). U7mB extracted and purified from *Cba. tepidum* comigrated with N-Me-BSmB, but not hCys-BSmB or BSmB, when analyzed in separate runs and exhibited a single symmetrical peak when U7mB was spiked with synthetic N-Me-BSmB (Fig. 3). Therefore, we conclude that *in vivo*, U7 is N-Me-BSH. N-Me-BSH could be quantified in *Cba. tepidum* and other *Chlorobiaceae* (Table 1): Chlorobium phaeobacteroides DSM265 and *Prosthecochloris* sp. strain CB11, which was recently enriched from the Chesapeake Bay (32). N-Me-BSH was also observed in Chlorobium luteolum DSM273 and Prosthecochloris aestuarii DSM271 at ∼2 to 8 pmol mg (dry weight) (mg dw)^−1^, with the variability due to being close to the detection limit of 0.5 pmol (mg dw)^−1^ for N-Me-BSH.

**TABLE 1:** Detection of N-Me-BSH and BSH in selected bacteria grown to early stationary phase

Organism	*nmbA* ortholog	N-Me-BSH concn [pmol (mg dw)^−1^]	BSH concn [pmol (mg dw)^−1^]
Chlorobaculum tepidum			
Wild type	CT1040	592 + 373	∼200^*a*^
ΔCT1040 (*nmbA*)	None	BDL^*b*^	791 + 157
*Prosthecochloris* sp. strain CB11	?^*c*^	65 + 29	BDL
Chlorobium phaeovibrioides DSM265	Cvib_0902	109 + 10	BDL
*Polaribacter* sp. strain MED152	MED152_02425	1,151 + 392	∼200^*a*^
Thermus thermophilus HB27	ND^*d*^	BDL	27 + 9

a Quantification of BSH at low levels is inaccurate due to an earlier eluting, overlapping peak in cell extracts from these strains.

b BDL, below detection limit which was ∼0.5 pmol (mg dw)^−1^ for N-Me-BSH and 20 pmol (mg dw)^−1^ for BSH.

c No whole-genome sequence is available for this organism.

d No bidirectional BLASTP best hit with an E value of <1e−30 was detected.

10.1128/mBio.01603-18.1 TEXT S1 Additional information on analytical methods and the synthesis of candidate LMW thiols. Download Text S1, PDF file, 0.2 MB. Copyright © 2018 Hiras et al. 2018 Hiras et al. This content is distributed under the terms of the Creative Commons Attribution 4.0 International license.

### Genetic identification of the *Cba. tepidum* N-Me-BSH biosynthetic pathway.

**FIG 4:**
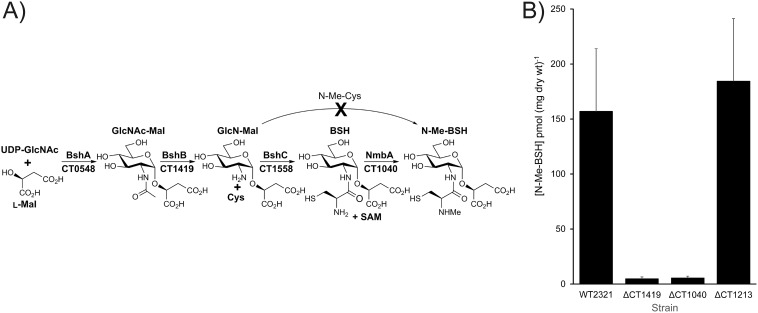
The proposed biosynthetic pathway for N-Me-BSH (A) and N-Me-BSH pool size (B) in *Cba. tepidum* deletion mutant strains ΔCT1419 (*bshB*), ΔCT1040 (putative SAM-dependent methyltransferase), and ΔCT1213 (putative SAM-dependent methyltransferase). The values shown for strains ΔCT1419 and ΔCT1040 indicate the limit of detection for N-Me-BSH. There is currently no evidence to support N-Me-BSH synthesis via N-Me-Cys addition to GlcNMal.

All *Chlorobiaceae* genome sequences encode orthologs of the three enzymes required for BSH biosynthesis from UDP-Glc-NAc, malic acid, and cysteine in Bacillus subtilis (Fig. S4). BshA (CT0548 in *Cba. tepidum*) condenses UDP-Glc-NAc and malic acid to produce D-Glc-NAc-L-Mal that is hydrolyzed to D-GlcN-L-Mal by BshB (CT1419), which is condensed with cysteine by BshC (CT1558) to produce BSH (Fig. 4A) (33). The requirement for this pathway for N-Me-BSH synthesis was confirmed by deleting CT1419/*bshB* from the *Cba. tepidum* genome. The resulting mutant strain did not contain detectable levels of N-Me-BSH (Fig. 4B) or BSH.

10.1128/mBio.01603-18.5 FIG S4 BLASTP search results with *Cba. tepidum* “orphan” methyltransferases as queries against *Chlorobi* and *Ignavibacteriales* complete genome sequences. Queries were selected as containing SAM methyltransferase domains (cd02440) but lacking obvious functional annotation. A match with an E value of <1e−40 is indicated with a + symbol. The pattern of BLASTP matches with *Cba. tepidum* BSH biosynthetic protein homologs (light green) were used to identify the most likely candidates for a BSH methyltransferase (dark green). Download FIG S4, PDF file, 0.6 MB. Copyright © 2018 Hiras et al. 2018 Hiras et al. This content is distributed under the terms of the Creative Commons Attribution 4.0 International license.

N-Me-BSH biosynthesis could potentially proceed via the ligation of N-Me-cysteine with GlcN-Mal or the N-methylation of BSH. BSH is often codetected with N-Me-BSH in *Cba. tepidum* extracts (Table 1), but N-Me-cysteine has never been observed (data not shown). This suggests that a BSH methyltransferase is required to synthesize N-Me-BSH. Orthologs of two putative SAM-dependent methyltransferases in the *Cba. tepidum* genome, CT1040 and CT1213, are found in all *Chlorobiaceae* genomes, as are genes for BSH biosynthesis (Fig. S4). Each gene was deleted from the *Cba. tepidum* genome, and the resulting mutant strains were analyzed for LMW thiols. The strain lacking CT1040 did not contain N-Me-BSH, while the strain lacking CT1213 contained levels of N-Me-BSH similar to those of the wild type (Fig. 4B). Furthermore, the strain lacking CT1040 contained levels of BSH similar to the concentration of N-Me-BSH in the parental wild-type strain (Table 1), indicating that the deletion of CT1040 causes a complete blockage of BSH methylation in this strain. Therefore, we conclude that the CT1040 gene product functions *in vivo* as a SAM-dependent BSH methyltransferase for which we propose the name NmbA for N-Me-BSH synthase A.

The mutant strains did not exhibit a strong growth phenotype relative to the wild type. In media with both sulfide and thiosulfate as electron donors, the *bshB*, *bshC*, and CT1040 deletion mutants grew ∼20% slower than the wild type did (doubling time of 3.8 to 3.9 h versus 3.2 h), while the CT1213 deletion strain grew at the same rate as the wild type (doubling time of 3.3 h). Most importantly, we did not observe any evidence of excess S(0) accumulation in cultures of the mutant strains (data not shown), which indicates that BSH and N-Me-BSH are not required for the oxidation of S(0) as had been predicted.

### N-methyl-BSH pool size varies with physiological status and is in the reduced state *in vivo*.

**FIG 5:**
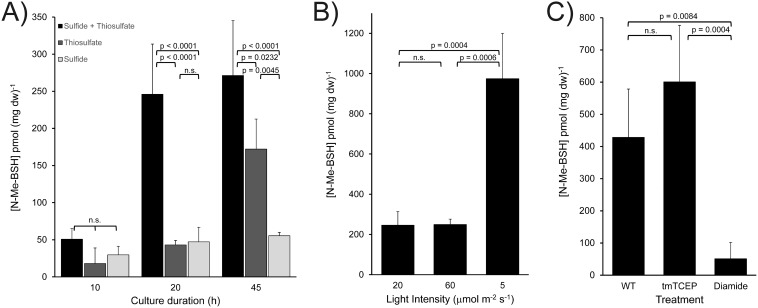
Dynamics of N-Me-BSH pool sizes in *Cba. tepidum.* (A) Pool size in *Cba. tepidum* grown for the indicated times (mid log phase, late log phase, early stationary phase) with different electron donor combinations. (B) Pool size in the wild-type cells grown with the indicated light fluxes grown to stationary phase. (C) Pool size in stationary-phase wild-type cells or cells treated with 1 mM trimethyl-TCEP (tmTCEP) or 2 mM diamide. Significant differences are indicated by *P* values calculated by the Tukey-Kramer HSD test after ANOVA. n.s., not significant (*P* > 0.05).

*Cba. tepidum* was grown with a variety of sulfur compounds as electron donors for photosynthesis, and the N-Me-BSH pool size was quantified at different growth stages. N-Me-BSH pool sizes increased during growth and were always highest in stationary phase (45 h, Fig. 5A). N-Me-BSH pool size and biomass were strongly correlated regardless of the electron donor used for growth (*r*^2^ = 0.788). *Cba. tepidum* cultures grown with thiosulfate and sulfide had the highest biomass concentrations, followed by thiosulfate or sulfide alone. This suggests that as cell density increases, the decrease in light intensity due to self-shading may drive an increase in the N-Me-BSH pool size. This was supported by the fact that *Cba. tepidum* grown at low light intensity contained fivefold-more N-Me-BSH than cells grown at standard or high light intensity (Fig. 5B, *P* = 0.047).

The redox state of the N-Me-BSH pool was assessed by treating stationary-phase *Cba. tepidum* cultures with trimethyl-TCEP, a phosphine reductant that is able to cross phospholipid bilayers (34), or diamide, a disulfide-generating electrophile that is used to induce sulfhydryl-specific oxidative stress (35–37). The addition of trimethyl-TCEP increased the N-Me-BSH pool size 1.4-fold compared to the untreated culture, but this change was not significant (*P = *0.13, Fig. 5C). In contrast, the addition of diamide decreased the N-Me-BSH pool size 8.4-fold (*P = *0.027, Fig. 5C), presumably by oxidation to the disulfide. Together, these results demonstrate that N-Me-BSH is found predominantly in its reduced state in cells and is a redox-responsive LMW thiol in *Cba. tepidum*.

### Phylogenetic distribution of LMW thiol biosynthetic genes.

**FIG 6:**
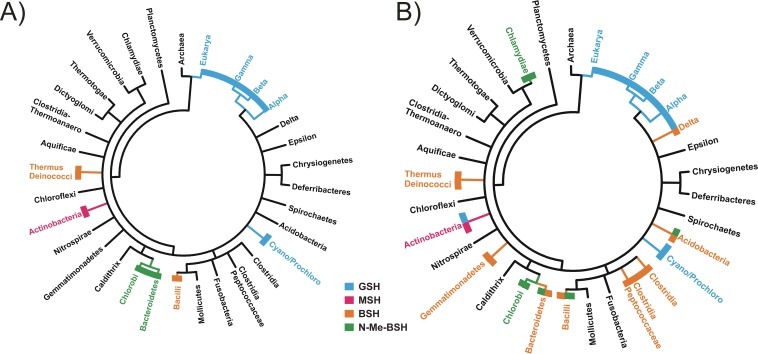
Distribution of LMW thiols in bacteria as determined by mBBr derivatization-HPLC (A) and the potential distribution based on an analysis of complete genome sequences (B) for the presence of orthologs encoding complete pathways for GSH (*gshA-B*), MSH (*mshA-D*), BSH (*bshA-C*), or N-Me-BSH (*bshA-C* plus *nmbA*).

The direct detection and structural analysis of LMW thiol metabolites are the gold standard for assessing their distribution (27, 38). The current distribution of directly detected LMW thiols in bacteria is outlined in Fig. 6A. We attempted to use gene content to predict LMW thiol content of bacteria phylogenetically distant from the *Chlorobiaceae*. *Polaribacter* sp. strain MED152 (*Bacteroidetes*) genome contains *bshA-C* and CT1040/*nmbA*, while the Thermus thermophilus HB27 (*Deinococcus-Thermus*) genome contains only *bshA-C*, and we had previously shown that BSH pools in this organism contribute to mercury resistance (39). This gene content predicts that *Polaribacter* sp. strain MED152 should synthesize N-Me-BSH, while T. thermophilus HB27 should synthesize BSH, but not N-Me-BSH. This prediction was confirmed by HPLC analysis of S-bimane derivatives (Table 1). On the basis of this clear relationship between gene and LMW thiol content, all complete microbial genome sequences in the Integrated Microbial Genomes database were searched for the presence of orthologs of *bshA-C* and *nmbA*. A complete N-Me-BSH biosynthetic pathway is found not only in the *Chlorobi* and *Bacteroidetes* but also in members of the phyla *Acidobacteria*, *Firmicutes*, and in a basal member of the *Chlamydiae*, Waddlia chondrophila (Fig. 6B). BSH biosynthesis has been chemically demonstrated in the *Deinococcus-Thermus* lineage and *Firmicutes* (4), and the gene content analysis here predicts that BSH would also be found in members of the *Bacteroidetes* and *Acidobacteria*. In comparison, mycothiol biosynthesis genes (*mshA-D*) are found only in the *Actinobacteria* and glutathione biosynthesis genes (*gshA-B*) in one member of the *Actinobacteria* (*Frankia* sp. strain EAN1pec), the *Cyanobacteria*, *Proteobacteria*, and *Eukarya*.

Within the *Chlorobi* and sister phylum *Ignavibacteriae*, all genomes appear to have a complete N-Me-BSH biosynthetic pathway except for the draft genome of NICIL-2 (40), which contains *bshA-C*, but not CT1040/*nmbA*. Outside of these taxa, around one third of the BSH biosynthesis pathway-positive *Acidobacteria* (33%) and *Bacteroidetes* (31%) contain an *nmbA* ortholog, while smaller numbers of *Firmicutes* appear to possess *nmbA* (7%, multiple *Paenibacillus* spp. and Brevibacillus brevis). This pattern and the presence of BSH in the deeply branching *Deinococcus-Thermus* group indicate that BSH was likely the ancestral thiol and that N-Me-BSH has been acquired in different lineages by gain of the *nmbA* gene. The *gshA* gene, encoding glutamate-cysteine ligase, is less widely distributed than the *gshB* gene, encoding glutathione synthetase that ligates glycine with γ-glutamyl cysteine. Only 79% of genomes encoding *gshB* also contain *gshA*. The *mshD* gene, encoding mycothiol synthase that adds an acetyl group to 1-D-myo-inosityl-2-L-cysteinylamido-2-deoxy-alpha-D-glucopyranoside (Cys-GlcN-Ins) to complete mycothiol synthesis is the least widely distributed of the *msh* genes. It occurs in only 27% of genomes that encode *mshC*, which encodes the enzyme that produces Cys-GlcN-Ins; *mshC* is the most widely distributed *msh* gene.

## DISCUSSION

The chemical and genetic data presented here indicate that N-Me-BSH is the major LMW thiol in the *Chlorobiaceae*. N-Me-BSH biosynthesis requires the function of gene products orthologous to *bshA-C* and a SAM-dependent methyltransferase named *nmbA* with CT1040 as the defining member of this gene family. N-methylation of cysteine in secondary metabolites is rarely observed. To the best of our knowledge, N-Me-BSH is only the fourth example of this modification. The others are pyochelin, an N-methylated thiazolidine-containing siderophore produced by Pseudomonas aeruginosa (41), kendarimide A, a poly-N-methylated oligopeptide including N-Me-Cys isolated from the Indonesian sponge *Haliclona* sp. (42), and thiocoraline, a depsipeptide isolated from a marine *Micromonospora* sp. (43). Pyochelin is, and the others are likely to be, synthesized by extremely large multidomain nonribosomal peptide synthetases where a specific MTase domain catalyzes the cysteine modification (41). N-Me-BSH appears to be the first case of cysteine N-methylation outside of oligopeptide metabolites where the N-methylation is catalyzed by a standalone MTase.

Chlorobaculum tepidum has previously been suggested to synthesize glutathione (44), and the Chlorobium limicola genome contains two genes, Clim_1148 and Clim_1149, whose recombinant products convert histidine to trimethylhistidine by a methyltransferase (Clim_1148, EanA) and trimethylhistidine to ergothioneine by a sulfur transferase reaction (Clim_1149, EanB) *in vitro* (26). The ergothioneine biosynthetic pathway was thought to be restricted to aerobic organisms, because one of the enzymes in the pathway, EgtB, required molecular oxygen (26). EanB does not require molecular oxygen, and its activity has been used to suggest an anaerobic origin for the ergothioneine biosynthetic pathway (26). Our data do not provide any evidence for either glutathione or ergothioneine as a major LMW thiol in *Cba. tepidum* or the other members of the *Chlorobiaceae* tested. CT1040/NmbA, the methyltransferase that our genetic data indicates is the sole route of N-Me-BSH synthesis in *Cba. tepidum*, is not closely related to Clim_1148/EanA. BLASTP searches using Clim_1148 indicate that this gene product is encoded only by a few *Chlorobiaceae* genomes (*C. limicola* strains DSM245 and Frassassi, *Prostecochloris* sp. strains V1 and GSB1, and *C. phaeobacteroides* BS1), indicating that if ergothioneine synthesis occurs *in vivo*, it is not widespread in the *Chlorobiaceae*. No homolog of Clim_1148/EanA is encoded by the *Cba. tepidum* genome. Our data show that the N-Me-BSH biosynthetic pathway is a universal feature of the *Chlorobiaceae*.

The functional consequences of producing N-Me-BSH versus BSH are currently unknown. While BSH is often detected in N-Me-BSH-producing bacteria, N-Me-BSH levels are always greater than those of BSH, suggesting that the methylated form is the most physiologically relevant. Modification of their predominant LMW thiol structure during stationary phase is known in some bacteria. Some marine actinomycetes produce MSH with a N-propionyl group instead of the normal N-acetyl group. The diversion of propionyl-CoA into N-propionyl-MSH is proposed to limit propionyl-CoA accumulation during degradation of odd-chain and branched-chain fatty acids (45). E. coli bacteria have also been shown to convert much of their GSH pool to glutathionylspermidine during stationary phase under anaerobic conditions, which is believed to modulate free GSH and/or spermidine in response to different environmental conditions (46). However, the *Chlorobiaceae* predominantly make N-Me-BSH irrespective of growth phase, suggesting that there has been selection for this molecule in their physiology.

The concentrations of N-Me-BSH detected in the *Chlorobi* under normal anaerobic growth conditions, 65 to 700 pmol thiol (mg [dw])^−1^, are at the lower end of the range of the BSH values previously reported in various *Bacilli* and Deinococcus radiodurans, 200 to 2,600 pmol thiol (mg dw)^−1^ (4, 47). The elevation in N-Me-BSH levels during exponential growth and reaching a maximum in stationary phase is similar to observations of BSH in B. subtilis (48). However, growth in low light elevated the N-Me-BSH level to ∼1,050 pmol thiol (mg dw)^−1^ in *Cba. tepidum*, similar to the level of N-Me-BSH in aerobically grown *Polaribacter* sp. strain MED152, ∼1,150 pmol (mg dw)^−1^. Thus, N-Me-BSH content in *Cba. tepidum* is influenced by growth state and environmental conditions. The comparison between *Cba. tepidum*, an obligate anaerobe, *Polaribacter* sp. strain MED152, and BSH-synthesizing *Bacilli* and D. radiodurans, all obligate aerobes, may imply a connection between LMW thiol content and growth in the presence of oxygen. Indeed, *Cba. tepidum* has multiple oxidative stress defense mechanisms that appear to be constitutively expressed (49), and N-Me-BSH may be a key component of these defense mechanisms. This may provide an advantage in environments where the *Chlorobiaceae* are often found: microbial mats alongside oxygenic phototrophs and at interfaces between oxic and anoxic waters. Under these conditions, redox balancing LMW thiols should provide an advantage to anaerobic anoxygenic phototrophs like the *Chlorobiaceae* and other anaerobic bacteria as well.

Physiologically, N-Me-BSH is the best current candidate for a LMW thiol proposed to facilitate the trafficking of sulfur atoms between the periplasm and cytoplasm in phototrophic sulfur-oxidizing bacteria, a role proposed for glutathione amide in *Chromatium gracile* (21). However, the fact that *Polaribacter* sp. strain MED152, which does not employ sulfur-based energy metabolism, synthesizes N-Me-BSH and many other members of the *Bacteroidetes*, *Acidobacteria*, and *Firmicutes* carry *nmbA* orthologs and likely synthesize N-Me-BSH indicate that the molecule cannot be tied exclusively to sulfur oxidation. Furthermore, in dissimilatory sulfate reduction, the DsrC protein has been shown to stimulate the activity of dissimilatory sulfite reductase, DsrAB, and appears to act as the preferred acceptor for the reduced sulfur atom by forming a trisulfide bridge between two cysteine side chains (50). DsrC trisulfide is then proposed to be reduced by DsrMKJOP, generating sulfide and regenerating DsrC to accept another sulfur atom. As phototrophic sulfur oxidation is proposed to involve a reversed Dsr system for oxidizing elemental sulfur to sulfite (22, 23), sulfur atom transfer to DsrC as a cytoplasmic acceptor may operate in place of a LMW thiol shuttle. This would explain the lack of correlation between N-Me-BSH and sulfur-based energy metabolism in these organisms.

Another possible function in the *Chlorobiaceae* is that N-Me-BSH is the *in vivo* reductant for the recently described mechanism for regulating excitation energy transfer in the FmoA protein (44). Cysteine localized thiyl radicals in FmoA are proposed to interact with excited bacteriochlorophyll *a* to prevent excitation energy transfer to the reaction center under unfavorable conditions. *In vitro*, dithionite, dithiothreitol, sulfide, glutathione, and TCEP were capable of regulating FmoA energy transfer, and GSH was proposed as the *in vivo* mediator (44). The results presented here indicate that GSH is not a good candidate for this function, as no *Chlorobiaceae* genomes encode GSH biosynthesis (Fig. 6B and Data Set S1) and GSH was not detected in *Cba. tepidum* (Fig. 2), *Chlorobium phaeobacteroides* DSM265, *Prosthecochloris* sp. strain CB11, *Chlorobium luteolum* DSM273, or *Prosthecochloris aestuarii* DSM271. Rather, these data suggest N-Me-BSH as the most likely thiol-based redox modulator of FmoA energy transfer. The data here predict that *Chloracidobacterium thermophilum* (51), the only organism outside of the *Chlorobi* to utilize FmoA in light harvesting, should synthesize N-Me-BSH (see Data Set S1 in the supplemental material), supporting this assertion. BSH-based thiols may be more suitable for this function because they have significantly lower redox potential than GSH (48). Detailed examinations of the physical and redox properties of N-Me-BSH and the mutant strains generated here will help to address this question. However, as with sulfur-based energy metabolism, the occurrence of N-Me-BSH in organisms that do not contain FmoA means that N-Me-BSH cannot be exclusively associated with light harvesting.

10.1128/mBio.01603-18.7 DATA SET S1 Metadata for genomes containing orthologs of the indicated LMW thiol biosynthetic genes identified as described in Materials and Methods. These data were analyzed to infer the presence of a complete biosynthetic pathway for BSH (orange cells), N-Me-BSH (green cells), GSH (blue cells), and MSH (magenta cells). Download Data Set S1, PDF file, 0.6 MB. Copyright © 2018 Hiras et al. 2018 Hiras et al. This content is distributed under the terms of the Creative Commons Attribution 4.0 International license.

Many LMW thiol reactions are enzyme mediated, e.g., by bacilliredoxins (9) or bacillithiol S-transferases (5), while others depend on the intrinsic chemical reactivity of LMW thiols, e.g., metal ion chelation (8) and conjugation with methylglyoxal (7). The N-methylation of BSH by NmbA may significantly alter its biophysical properties. N-methylation could enhance the basicity of the cysteinyl amine, making it more readily protonated and better positioned to stabilize the thiolate anion, thereby lowering the pKa. This would increase the abundance of N-Me-BSH in its more chemically reactive thiolate form at physiological pH; however, this remains to be experimentally determined.

The genetic data indicate that N-Me-BSH is synthesized in *Cba. tepidum* after BSH biosynthesis by the CT1040/*nmbA* gene product, a SAM-dependent methyltransferase. The role of methylation can thus be explored by generating mutant strains expressing noncognate thiols, i.e., N-Me-BSH in B. subtilis and BSH in *Cba. tepidum*, to address the functional significance of this rare metabolic modification. As a stand-alone methyltransferase, NmbA could potentially be used to methylate a wide range of small-molecule targets to improve properties or activities. Identifying *nmbA* allowed us to predict and confirm LMW thiol biosynthetic capacity in complete genome sequences over long phylogenetic distances. This, in turn, led us to conclude that LMW thiols based on the BSH backbone are likely the most widely distributed thiols in biology. The analysis also highlighted groups of organisms that should be targeted to more fully understand the diversity of LMW thiol structure and function. Major bacterial lineages e.g., *Verrucomicrobia*, *Planctomycetes*, *Spirochaetes*, and others, have no documented or predicted LMW thiol for redox homeostasis, a critical cellular function. Future genome-directed studies of LMW thiol diversity, focusing on strains whose genomes encode partial or no recognized LMW thiol biosynthetic pathways, should uncover further variations on LMW thiol molecular backbones that underlie critical metabolic processes and where enzymes generating this biochemical diversity may find applications for engineered product synthesis.

## MATERIALS AND METHODS

### Bacterial growth conditions and media.

All strains and antibiotic selections used in this study are listed in Table S3 in the supplemental material. Escherichia coli strains were grown in lysogeny broth at 37°C (52). Chlorobaculum tepidum strains were grown in Pf-7 medium buffered to pH 6.95 with the addition of 1,3-bis(tris(hydroxymethyl)methylamino)propane (BTP) (MP Biomedicals, Solon, OH) as previously described (14, 53) in 250-ml narrow-neck screw-cap medium bottles sealed with black open top phenolic caps containing a flanged butyl rubber septum (Fisher Scientific, Pittsburgh, PA). All cultures were maintained anaerobically and pressurized to 10 lb/in^2^ with 5% CO_2_/95% N_2_. *Cba. tepidum* cultures were grown at 47°C with 20 µmol photons m^−2^ s^−1^ (standard light), 60 µmol photons m^−2^ s^−1^ (high light), or 5 µmol photons m^−2^ s^−1^ (low light), supplied by 40- or 100-W neodymium full-spectrum bulbs (Lumiram Electric Corp., Larchmont, NY). All irradiance measurements were made with a light meter equipped with a quantum PAR sensor (LI-COR, Lincoln, NE).

10.1128/mBio.01603-18.10 TABLE S3 Strains, plasmids, and primers used in this study. Download Table S3, PDF file, 0.1 MB. Copyright © 2018 Hiras et al. 2018 Hiras et al. This content is distributed under the terms of the Creative Commons Attribution 4.0 International license.

### Metabolite extraction and mBBr derivatization.

A modified version of the mBBr extraction and derivatization protocol of Fahey and Newton (28) was used to extract thiols from both *Cba. tepidum* and E. coli. Details on equipment and HPLC separations are provided in Text S1 in the supplemental material.

### Effect of reductant or oxidant on LMW thiol pool size.

Stationary-phase wild-type *Cba. tepidum* cells (48 h of growth) were treated with 2 mM trimethyl-TCEP, which was synthesized as described in Text S1 (34), or treated with 1 mM diamide or not treated. The cultures were then incubated for 40 min in an anaerobic chamber before samples were harvested for mBBr extraction and derivatization.

### Liquid chromatography-tandem mass spectrometry.

Bimane derivatives of interest were collected from multiple HPLC runs of the same sample and lyophilized (Labconco FreeZone 4.5, Kansas City, MO) for 12 h. The concentrated material was resuspended in 0.075% (vol/vol) glacial acetic acid and 68% (vol/vol) methanol, followed by further concentration in a SpeedVac and reconstitution in water.

High-resolution, positive-ion-mode ESI LC-FT-ICR-MS was performed with a 50-mm C_18_ column. Solvent A was 0.1% aqueous acetic acid, pH 3.5, and solvent B was methanol. The 30-min elution protocol (0.2 ml min^−1^) was as follows: 0 min, 15% solvent B; 5 min, 15% solvent B; 15 min, 23% solvent B; 17 min, 42% solvent B; 20 min, 42% solvent B; 20.02 min, 15% solvent B. This method and solvent system resulted in one bimane derivative eluting at 5.7 min, which was then analyzed by a 7 T Fourier-transform ion cyclotron resonance mass spectrometer (FT-ICR MS) from Thermo Scientific (LTQ FT Ultra hybrid mass spectrometer). The front end of the instrument is a linear ion trap mass spectrometer (LTQ MS) which serves as the ion accumulation site for ultrahigh-resolution analysis in the ICR cell. Fragmentation was provided by collision-induced dissociation (CID) in the linear ion trap, followed by FT-ICR MS analysis.

### Synthesis and analysis of bacillithiol derivatives.

S-bimane derivatives of N-Me-BSH and hCys-BSH were synthesized following similar procedures previously developed for BSH synthesis (54), but using suitably protected N-methyl cysteine and homocysteine building blocks in place of the protected cysteine building block used for BSH synthesis (Text S1).

### Gene deletion and mutant analysis.

Full details of the protocol for in-frame deletion of genes in *Cba. tepidum* will be described elsewhere (J. M. Hilzinger, V. Raman, and T. E. Hanson, in preparation). Briefly, regions flanking the gene to be deleted were amplified by PCR using primers listed in Table S3 and inserted into pKO2.0-Sm/Sp by Gibson Assembly (New England Biolabs, Ipswich, MA). Plasmid pKO2.0-Sm/Sp is based on pKO2.0 used to delete genes in Shewanella oneidensis (55). pKO2.0 was modified by replacing the gentamicin resistance gene with the streptomycin/spectinomycin resistance cassette from pHP45Ω (56). Plasmids were mobilized from E. coli strain β-2155 to *Cba. tepidum* by conjugation (19, 55). Single recombinants were selected with spectinomycin and streptomycin, verified to contain both the WT and deletion alleles of the gene by PCR, and then grown in Pf-7 medium without antibiotic selection. Double recombinants were obtained by plating this culture onto solid medium containing 10% (wt/vol) sucrose, and strains containing only the deletion allele of the gene of interest were identified by PCR. These strains were grown in liquid Pf-7 medium for thiol analysis as described above.

### Bioinformatic analysis of LMW thiol biosynthetic pathways.

Orthologs of genes for LMW thiol biosynthetic pathways were collected from finished microbial genome sequences in the Integrated Microbial Genomes database (https://img.jgi.doe.gov) using the Custom Homolog Selection tool requiring a minimum amino acid sequence identity of 30%, a minimum BLASTP E value of 1^−20^, and similar length of query and subject. Genes used as queries to collect the orthologs were CT0548 (*bshA*), CT1419 (*bshB*), CT1558 (*bshC*), b2688 (*gshA*), b2947 (*gshB*), slr0990 (*gshA*), slr1238 (*gshB*), Rv0486 (*mshA*), Rv1170 (*mshB*), Rv2130c (*mshC*), and Rv0819 (*mshD*). Taxonomic information was retrieved for each genome containing an ortholog of a given gene and combined lists for genomes containing multiple orthologs (i.e. all genomes containing *mshA* plus *mshB* plus *mshC* plus *mshD*) constituting a pathway assembled from single ortholog lists using the UNIX command “grep” and the LOOKUP function in Microsoft Excel. The complete list of genomes inferred to encode the components involved in biosynthesis of each thiol is provided as Data Set S1 in the supplemental material. SAM-dependent methyltransferases were identified in the *Cba. tepidum* genome by searching for proteins containing domain cd02440 (AdoMet_MTases) but lacking obvious functional annotation. These protein sequences were used as queries for BLASTP searches against all other *Chlorobi* proteins to determine their distribution relative to the *bshA-C* orthologs above.

10.1128/mBio.01603-18.6 FIG S5 Synthesis routes for the analytical standards N-Me-BSmB (A) and hCys-BSmB (B). Download FIG S5, PDF file, 0.1 MB. Copyright © 2018 Hiras et al. 2018 Hiras et al. This content is distributed under the terms of the Creative Commons Attribution 4.0 International license.
