# Supplementary material for: Physiological Studies of Chlorobiaceae Suggest that Bacillithiol Derivatives Are the Most Widespread Thiols in Bacteria
Source: mBio. 2018 Nov 27;9(6):e01603-18. doi: 10.1128/mBio.01603-18 (PMC6282198; doi:10.1128/mBio.01603-18)

Figure S1. Representative HPLC chromatograms of bimane derivatized compounds: A) *Cba. tepidum* stationary phase extract, B) mixture of authentic standards, C) reagent blank. Retention times of standard compounds are noted with blue dashed lines. Retention times of reagent blank peaks are noted with red dashed lines. The identities of standard bimane derivatives are labeled: cysteine (Cys), thiosulfate ($S_2O_3^{2-}$), β-mercaptoethanol (β-ME), sulfide (HS-), and dithiothreitol (DTT). Note that DTT elutes in two distinct peaks.

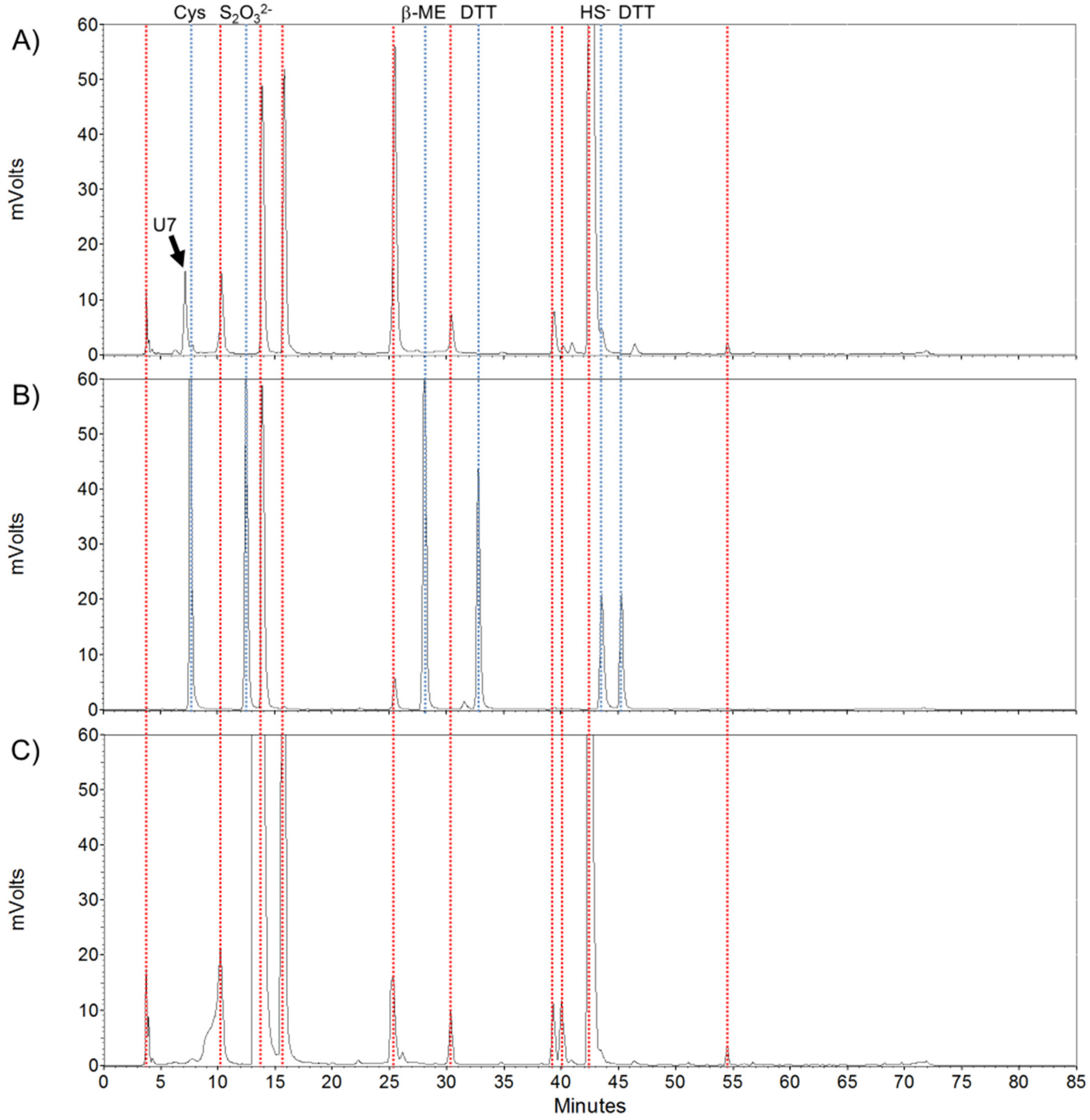

Supplement: FIG S1 [file mbo006184195sf1.pdf]
