# Supplementary material for: Physiological Studies of Chlorobiaceae Suggest that Bacillithiol Derivatives Are the Most Widespread Thiols in Bacteria
Source: mBio. 2018 Nov 27;9(6):e01603-18. doi: 10.1128/mBio.01603-18 (PMC6282198; doi:10.1128/mBio.01603-18)

Figure S2. The effect of TCEP on mBBr derivatized compounds in *Cba. tepidum* extracts. The solid line is untreated mBBr extract, the dashed line is the same sample after reduction with TCEP. A) Region surrounding U7 B) Region surrounding sulfide. Bimane derivatives are labeled with their identity determined by co-chromatography with authentic standards.

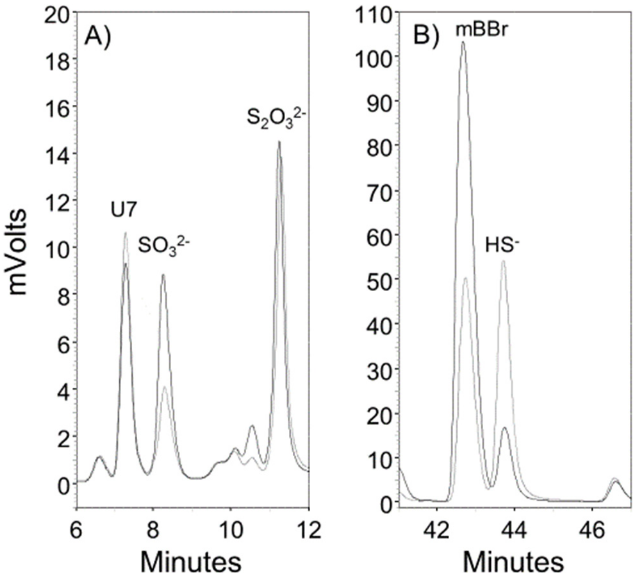

Supplement: FIG S2 [file mbo006184195sf2.pdf]
