# Supplementary material for: Physiological Studies of Chlorobiaceae Suggest that Bacillithiol Derivatives Are the Most Widespread Thiols in Bacteria
Source: mBio. 2018 Nov 27;9(6):e01603-18. doi: 10.1128/mBio.01603-18 (PMC6282198; doi:10.1128/mBio.01603-18)

Figure S3. FT-ICR-MS spectrum of U7mB as purified from *Cba. tepidum* (A) and MS/MS spectrum of the 603.1964 ion after collision induced dissociation (B).

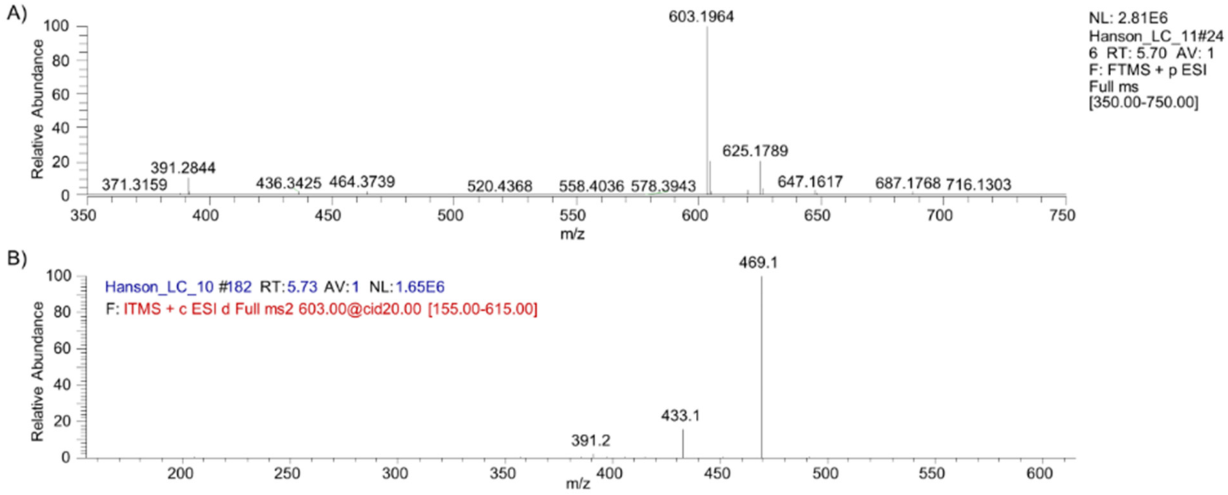

Supplement: FIG S3 [file mbo006184195sf3.pdf]
