# Supplementary material for: Physiological Studies of Chlorobiaceae Suggest that Bacillithiol Derivatives Are the Most Widespread Thiols in Bacteria
Source: mBio. 2018 Nov 27;9(6):e01603-18. doi: 10.1128/mBio.01603-18 (PMC6282198; doi:10.1128/mBio.01603-18)
Supplement: TABLE S1 [file mbo006184195st1.pdf]

Table S1. Masses of intact U7mB and CID fragments detected by MS^n.

High resolution masses of U7mB adduct and subsequent fragments

ID	Ion Mass $[M+H]^+$ (Da)	Calculated neutral exact mass minus bimane (Da)	Neutral loss (Da)
Sample A	603.1961	412.11	
Sample B	603.1964	412.11	
Fragment *a*	469.1753	278.09	134
Fragment *b*	433.1543	242.07	170
Fragment *c*	391.1437	200.06	212

Deduced formulas of neutral loss fragments

Lost fragment	Mass lost	Formula lost	Possible compound[a] lost
U7mB-Frag *a*	134	$C_4H_6O_5$	Malic acid<br>3-Dehydro-L-threonate<br>diglycolic acid
Frag *a*-Frag *b*	36	H_4O_2	$2H_2O$
Frag *b*-Frag *c*	42	C_2H_2O	Acetyl as a ketene (CH_2=C=O)

a-Compounds were identified by literature searches with the deduced formulas and masses of neutral lost fragments.
