# Supplementary material for: Physiological Studies of Chlorobiaceae Suggest that Bacillithiol Derivatives Are the Most Widespread Thiols in Bacteria
Source: mBio. 2018 Nov 27;9(6):e01603-18. doi: 10.1128/mBio.01603-18 (PMC6282198; doi:10.1128/mBio.01603-18)
Supplement: TABLE S2 [file mbo006184195st2.pdf]

Table S2. Deduced neutral mass values and associated formulas for FT-ICR-MS and MS/MS fragments with the mass of bimane subtracted.

**U7 candidates**	**MS/MS Fragments**		
***m/z* 412.11**	***a*-278.09**	***b*-242.07**	***c*-200.06**
$C_{19}H_{28}N_2S_4$	$C_{11}H_{22}N_2S_3$	**$C_{10}H_{14}N_2O_3S$**	**$C_8H_{12}N_2O_2S$**
$C_{19}H_{20}N_6OS_2$	**$C_{10}H_{18}N_2O_5S$**	$C_3H_{22}N_4S_4$	$CH_{12}N_8S_2$
$C_{18}H_{26}N_2O_3P_2S$	$C_5H_{15}N_{10}PS$	$C_3H_{14}N_8OS_2$	$H_{18}N_4O_2P_2S$
$C_{17}H_{33}OPS_4$	$C_3H_{18}N_8O_3S_2$	$C_2H_{20}N4O_3P_2S$	
$C_{17}H_{25}N_4O_2PS_2$	$C_2H_{24}N_4O_5P_2S$		
$C_{16}H_{31}O_4P_3S_4$	$C_2H_{22}N_4O_7S_2$		
$C_{16}H_{27}O_6PS_2$			
**$C_{14}H_{24}N_2O_{10}S$**			
$C_{12}H_{12}N_{16}S$			
$C_{11}H_{34}N_4P_2S_4$			
$C_{11}H_{26}N_8OP_2S_2$			
$C_{11}H_{16}N_{12}O_4S$			
$C_{10}H_{32}N_4O_3P_4S$			
