# Supplementary material for: Physiological Studies of Chlorobiaceae Suggest that Bacillithiol Derivatives Are the Most Widespread Thiols in Bacteria
Source: mBio. 2018 Nov 27;9(6):e01603-18. doi: 10.1128/mBio.01603-18 (PMC6282198; doi:10.1128/mBio.01603-18)
Supplement: TEXT S1 [file mbo006184195s1.pdf]

SUPPORTING INFORMATION

MATERIALS AND METHODS

Metabolite extraction and derivatization. At the indicated times, cultures were transferred to an anaerobic chamber (Coy Laboratories Inc., Grass Lake, MI) and cells were harvested from 25 to 100 ml of culture by filtration onto pre-weighed Whatman GF/F filters (Fisher Scientific, Fair Lawn, NJ). An aliquot (50 µl) of the filtrate was added to 500 µl of derivatization mix [D-mix = 9.4 mM mBBr (Sigma-Aldrich, St. Louis, MO), 50% acetonitrile (Fisher Scientific, Fair Lawn, NJ), 50 mM HEPES (Acros Organics, Morris Plains, NJ) + 5 mM EDTA (EM Science, Gibbstown, NJ), pH=8.0] in an amber HPLC vial and incubated in the dark for 15 min. Filters were then washed once with 50 ml sterile anoxic sulfur free Pf-7 medium. The filter holder was transferred to a new 25 ml vacuum filtration flask and 1 ml derivatization mix allowed to inundate the washed filter and cells for 20 seconds before filtration. The filtrate was collected and passed through the filter two more times. The filtrate was transferred to an amber HPLC vial and incubated in the dark for 15 min at room temperature. Methanesulfonic acid (Sigma-Aldrich, Milwaukee, WI) was added to all samples at a final concentration of 25 mM to stop the derivatization reaction. Residual biomass retained by the filters was determined after drying and used to normalize thiol content between samples. Samples were stored at -20 °C until analysis by HPLC.

HPLC separation and quantification of bimane derivatives. Bimane derivatives were separated on a class VP gradient HPLC system (Shimadzu Scientific Instruments, Kyoto, Japan) equipped with a column oven, UV/visible detection, fluorescence detection, and a fraction collector using a 4.6 x 250 mm Beckman Coulter Ultrasphere ODS C18 5 µm analytical column (Beckman Instruments, Fullerton, CA) and a Beckman Coulter Ultrasphere ODS 4.6 mm x 4.5 cm

guard column (Beckman Coulter Inc., Fullerton, CA). The method was essentially method 1 described by Fahey and Newton (16). Solvent A was 0.25% aqueous acetic acid, pH 3.5, and Solvent B was methanol. The elution protocol (1 ml min^{-1}) was as follows: 0 min, 15% B; 5 min, 15% B; 15 min, 23% B; 45 min, 42% B; 65 min, 75% B; 67 min, 100% B; 70 min, 15% B; 85 min, 15% B. Standard thiol derivatives were used to identify and quantify thiols extracted from cultures. Aliquots (10 µl) of freshly prepared 100 mM solutions of cysteine (Acros Organics, Morris Plains, NJ), glutathione (Acros Organics, Morris Plains, NJ), β-mercaptoethanol (Acros Organics, Morris Plains, NJ), sodium thiosulfate (Sigma-Aldrich, St. Louis, MO), sodium sulfide (Fisher Scientific, Fair Lawn, NJ), dithiothreitol (Em Science, Gibbstown, NJ), and sodium sulfite (Sigma-Aldrich, St. Louis, MO) were derivatized with 100 µl D-mix. Standards were incubated at room temperature for 15 minutes in the dark. Reactions were stopped with methanesulfonic acid at 25 mM final concentration. Excluding sulfite, standards were combined to create a 50 µM mixed standard thiol derivatives that was used as a reference sample and to construct a standard curve based on fluorescence detector peak area for a range of injection volumes. Retention times for the standard compounds (Table 2) are somewhat different from those reported by Fahey and Newton (16), which we attribute to differences in the guard column and HPLC system.

Effect of thiol blocking agent on LMW thiol pool size. Late-log phase wild-type *Cba. tepidum* cells were harvested as described above. Prior to treatment with mBBr, 5 mM N-ethylmaleimide (NEM, Acros Organics, Morris Plains, NJ) was applied to the filters and incubated at room temperature for 15 minutes in the dark.

Effect of reductant on bimane derivatives. Extracts containing thiol derivatives were reduced with TCEP (Thermo Scientific, Rockford, IL) to test for the presence of internal disulfide bonds. Reduction of samples with TCEP was performed essentially according to Franz *et al.* (17). Samples were neutralized with 1 M NaOH followed by addition of 50 µL 50 mM HEPES + 5 mM EDTA, pH 8.0, per ml sample. Then, 20 µL 5 mM TCEP in 50 mM HEPES + 5 mM EDTA, pH 8.0, was added followed by incubation at room temperature for 30 min. Before terminating the reduction reaction with methanesulfonic acid, a 100 µL aliquot was transferred to a new HPLC vial. This new sample was treated with fresh derivatization mix and analyzed by HPLC as described above.

Synthesis of trimethyl-TCEP. Trimethyl-TCEP was synthesized by adding one drop of concentrated HCl to a test tube containing 100 mg of TCEP dissolved in 2 mL of methanol and stirring at room temperature for 3 days (10). The mixture was lyophilized using a Labconco FreeZone 4.5 freeze dry system (Kansas City, MO) for 12 hours. The final product was approximately 250 µL of 1.4 M trimethyl-TCEP, a viscous liquid.

Synthesis of N-Me-BSmB. The overall pathways for the synthesis of N-Me-BSmB and hCys-BSmB are detailed in Figure S6. Compound **1** (46) and S-Trt-N-(Me)Boc Cys (49) were prepared as previously described. Protected N-Me-BSH (**2**, Fig. S5A) was synthesized starting from a solution of amine **1** (854mg, 0.89 mmoles) and (*S*-Trt-*N*-(Me)BocCysOH (453mg, 0.98 mmoles) under N_2 in dry DMF (4.5 mL) to which PyBOP (555mg, 1.1 mmoles) and DIPEA (464µL, 2.7 mmoles) were sequentially added. The reaction was then stirred under inert atmosphere for 12 h. The mixture was diluted with EtOAc (50 mL) and washed successively

with 10% $NaHCO_3$ (20 mL), H_2O (20 mL), and brine (20 mL). The organic layer was dried over $MgSO_4$ and solvent evaporated under reduced pressure. The crude mixture was purified by chromatography using 10:1 hexanes: EtOAc to EtOAc gradient yielding the fully protected product **2** (454 mg, 53%). ^1H NMR (400 MHz, $CDCl_3$) δ 7.37 - 7.32 (m, 6H), 7.18 – 7.12 (m, 6H), 7.12 – 7.08 (m, 3H), 6.86 (d, J = 7.3 Hz, 1H), 5.95 – 5.70 (m, 2H), 5.29 (dq, J = 4.3, 1.5 Hz, 1H), 5.26 – 5.21 (m, 2H), 5.20 (dd, J = 2.0, 1.1 Hz, 1H), 5.02 – 4.84 (m, 2H), 4.58 – 4.51 (m, 4H), 4.35 (dd, J = 8.6, 4.2 Hz, 1H), 4.24 – 4.10 (m, 3H), 3.97 – 3.86 (m, 1H), 3.78 (dd, J = 10.5, 9.2 Hz, 1H), 2.81 – 2.71 (m, 3H), 2.57 (s, 3H), 2.56 – 2.51 (m, 3H), 2.04 – 1.89 (m, 9H), 1.32 (d, J = 4.6 Hz, 9H). ^{13}C NMR (100 MHz, $CDCl_3$) δ 170.82, 170.39, 169.84, 155.49, 144.82, 131.28, 131.09, 129.57, 127.85, 126.55, 119.56, 119.19, 99.68, 80.03, 75.44, 71.75, 70.72, 68.84, 66.26, 66.03, 61.93, 59.76, 54.76, 36.94, 32.08, 29.69, 28.41, 28.34, 22.72, 20.88, 20.79. HRMS *m/z* calc. for $[C_{50}H_{61}N_2O_{15}S]$ = $[M+H]^+$: 961.3784 found 961.3787, 100%.

N-Me-BSH disulfide ((Glc-Mal-N-MeCys)$_2$, **4**, Fig. S5A). In a flame dried flask, a solution of **2** (135 mg, 0.14 mmol) and imidazole (38 mg, 0.56 mmol) in anhydrous DCM (5 mL) was stirred under N_2 for 30 min. Tetra-*kis*(triphenylphosphine)palladium (7.4 mg, 0.028 mmol) was added and the reaction was stirred at rt for 14 h. The mixture was filtered through a small column of Dowex-H^+ resin eluting with DCM and the solvents were then evaporated under reduced pressure. The crude product was then dissolved in anhydrous MeOH (5 mL), cooled to 0 ºC followed by the addition of freshly prepared 1M NaOMe in anhydrous MeOH (200 µL, 0.2 mmol) and left to stir at 0 °C for 3 h. The reaction was quenched by filtration through Dowex-H^+ resin and the solvents were then evaporated under reduced pressure. Under an inert atmosphere, the crude product was cooled to 0 °C and an ice cold 80:20:2 mixture of TFA: CH_2Cl_2: Et_3SiH (4 mL) was added. The mixture was stirred at 0 °C for 75-90 min. The solvent was evaporated

under reduced pressure. The oily residue was triturated with cold ether (3 × 2 mL) and the resultant white solid was dissolved in H_2O (1 mL), filtered and freeze-dried and partially purified by reverse-phase (C-18) chromatography using water-methanol (0.1% TFA) eluents to give a thiol/disulfide mixture of **3** and **4**. This was fully oxidized to the disulfide by dissolving in an aqueous solution of 100 mM ammonium bicarbonate (1 mL, (100 mM), which was then stirred in the presence of air for 1 hour until no more free thiol was detected by Ellman's reagent test. The reaction was freeze dried and purified by anion exchange chromatography (DEAE, gradient of 10-200 mM ammonium bicarbonate eluent) to give **4** (20mg, 34% yield over 3 steps). δ_H (400 MHz, D_2O) 5.04 (d, J = 3.7 Hz, 1H), 4.45-4.42 (m, 1H), 4.11 (t, J = 5.7 Hz, 1H), 3.91 (dd, J = 10.8, 3.7 Hz, 1H), 3.82 – 3.63 (m, 5H), 3.46 (t, J = 9.5 Hz, 1H), 3.31 (qd, J = 15.3, 6.0 Hz, 2H), 2.82 (m, 2H), 2.72 (s, 3H). ^{13}C NMR (100 MHz, D_2O) 175.35, 174.10, 166.99, 97.66, 74.14, 72.73, 70.40, 69.65, 60.06, 54.04, 37.36, 37.07, 31.94. $[\alpha]_D^{22}$ = +35 (c = 0.5, H_2O, pH 2 TFA). HRMS *m/z* calc. for $[C_{28}H_{46}N_4O_{20}S_2]$ = $[M+H]^+$: 821. 2074; found 821.2051, 100%.

N-Me-BSmB was prepared by dissolving disulfide **4** (5mg, 6.1nmoles) in H_2O: MeOH (50:50v/v) (1mL) at pH 5-6 and adding the solution to a 1.5 mL column of TCEP immobilised on 4% cross-linked beaded agarose. The disulfide was left on the column for a period of 1hr before eluting with H_2O: MeOH (50: 50v/v) and two column volumes of ultrapure water. Thiol solutions were combined and quantified by titration against Ellman's reagent (DTNB) (2 mM) in phosphate buffer (pH 7.5) and measurement of the absorbance increase at 412 nm (ε= 14150 M^{-1} cm^{-1}) due to formation of 5-thio-2-nitrobenzoic acid (TNB) (50) prior to being labelled with monobromobimane as previously described (20).

Synthesis of hCys-BSmB. Protected hCys-BSH (**6**, Fig. S5B) was synthesized starting with a solution of S-Trt-N-Boc-hCysOH (186 mg, 0.39 mmol) in dry DMF (2 mL), to which PyBOP (220 mg, 0.43 mmol), HOBt (58 mg, 0. 43 mmol) and DIPEA (110 µL, 0.65 mmol) were successively added. The mixture was stirred for 30 min and a solution of amine **1** (215 mg, 4.2 mmol) and DIPEA (110 µL, 0.65 mmol) in DMF (1 mL) were added. The reaction was stirred at room temperature under inert atmosphere for 12 h, at which point the mixture was diluted with EtOAc (30 mL) and washed successively with 10% $NaHCO_3$ (10 mL), H_2O (5 x 10 mL), and brine (20 mL). The organic layer was dried over $MgSO_4$ and solvent evaporated under reduced pressure. The crude mixture was purified by flash chromatography using 10:1 hexanes: EtOAc to EtOAc gradient yielding the fully protected product as clear colourless oil (165 mg, 44%). ^{1}H NMR (400 MHz) δ 7.34 – 7.28 (m, 6H), 7.28 - 7.19 (m, 6H), 7.16 – 7.09 (m, 3H), 6.41 (d, *J* = 9.2 Hz, 1H), 5.88-5.72 (m, 2H), 5.31 – 5.17 (m, 4H), 5.16 – 5.11 (m, 1H), 5.05 (t, *J* = 9.8 Hz, 1H), 4.98 (d, *J* = 8.6 Hz, 1H), 4.80 (d, *J* = 3.6 Hz, 1H), 4.59 – 4.49 (m, 4H), 4.37 – 4.23 (m, 2H), 4.23 – 4.17 (m, 1H), 4.12 (dd, *J* = 12.6, 3.6 Hz, 1H) 4.05 – 3.99 (m, H), 3.92 (dd, *J* = 12.5, 2.2 Hz, 1H), 2.82 (dd, *J* = 16.7, 9.3 Hz, 1H), 2.70 (dd, *J* = 16.8, 3.7 Hz, 1H), 2.30 – 2.15 (m, 1H), 2.14 – 2.05 (m, 1H), 2.01 (s, 4H), 1.93 (s, 3H), 1.87 (s, 3H), 1.85 – 1.72 (m, 1H), 1.51 – 1.39 (m, 1H), 1.32 (s, 9H). ^{13}C NMR (100 MHz, $CDCl_3$) δ 171.88, 171.02, 170.74, 169.95, 169.72, 169.34, 155.07, 144.74, 131.37, 131.12, 129.58, 127.86, 126.64, 119.55, 119.22, 99.39, 79.80, 75.52, 70.33, 68.86, 67.81, 66.85, 66.30, 66.15, 61.57, 54.19, 51.88, 37.04, 32.27, 28.31, 21.04, 20.75, 20.59.

hCys-BSH was synthesized by making, in a flame dried flask, a solution of **6** (186 mg, 0.39 mmol) and imidazole (42 mg, 0.62 mmol) in anhydrous DCM (5 mL) that was stirred under N_2 for 30 min. Tetra-*kis*(triphenylphosphine)palladium (8.4 mg, 0.032 mmol) was added and the

reaction was stirred at rt for 14 h. The mixture was filtered through a small column of Dowex-H^+ resin eluting with DCM to remove the imidazole. The crude mixture was used evaporated to dryness and used without further purification. Freshly prepared 1M NaOMe in anhydrous MeOH (200 µL, 0.2 mmol) was added to above triacetate in anhydrous MeOH (4 mL) at 0 °C. The reaction was stirred at 0 °C for 3 h, and quenched by filtration through Dowex-H^+ resin. This product was evaporated to dryness and used for the final deprotection without further purification. Under an inert atmosphere, intermediate obtained above (55 mg, 0.072 mmol) was cooled to 0 °C and an ice cold 80:20:2 mixture of TFA: CH_2Cl_2: Et_3SiH (4 mL) was added. The mixture was stirred at 0 °C for 75-90 min. The solvent was evaporated under reduced pressure. The oily residue was triturated with cold ether (3 × 2 mL). Resultant white solid was dissolved in H_2O (1 mL), filtered and freeze-dried to afford crude **7** as a TFA salt. This product was further purified by reverse-phase (C-18) chromatography using water-methanol (0.1% TFA) eluents to give the final product **7** a clear oil (~25 mg, 85%). ^1H NMR (400 MHz, D_2O) δ 5.10 (d, J = 3.6 Hz, 1H), 4.54 (dd, J = 8.2, 3.8 Hz, 1H), 4.12 (t, J = 6.6 Hz, 1H), 3.96 (dd, J = 10.8, 3.7 Hz, 1H), 3.90 – 3.67 (m, 4H), 3.51 (t, J = 9.6 Hz, 1H), 2.96 (dd, J = 12.4, 4.2 Hz, 1H), 2.89 (dd, J = 17.2, 8.7 Hz, 1H), 2.65 (t, J = 7.4 Hz, 2H), 2.20 (q, J = 7.1 Hz, 2H). ^{13}C NMR (100 MHz, D_2O) δ 175.39, 174.64, 170.08, 98.76, 74.87, 73.37, 70.90, 70.42, 60.70, 54.31, 52.98, 37.55, 35.38, 19.57.

hCys-BSmB was prepared from hCys-BSH dissolved in water as described for N-Me-BSmB above.
