# Supplementary material for: Physiological Studies of Chlorobiaceae Suggest that Bacillithiol Derivatives Are the Most Widespread Thiols in Bacteria
Source: mBio. 2018 Nov 27;9(6):e01603-18. doi: 10.1128/mBio.01603-18 (PMC6282198; doi:10.1128/mBio.01603-18)
Supplement: FIG S4 [file mbo006184195sf4.pdf]

Figure S4. BLASTP search results with *Cba. tepidum* "orphan" methyltransferases as queries against *Chlorobi* and *Ignavibacteriales* complete genome sequences. Queries were selected as containing SAM methyltransferase domains (cd02440), but lacking obvious functional annotation. A match with an e-value of < 1e-40 is indicated with "+". The pattern of BLASTP matches with *Cba. tepidum* BSH biosynthetic protein homologs (light green) were used to identify the most likely candidates for a BSH methyltransferase (dark green).

Gene	Description	Accession	Cp DSM265	Pp BU-1	Cp DSM266	Cf	Cl DSM245	Cl DSM273	Pa DSM271	Cc CaD3	Cp BS1	Cp NCIB8327	Ct ATCC35110	Ia JCM16511	Mr P3M-2	
CT0100	hypothetical protein	NP_661006	+	+	+	+	+	+	+	+	+		+			
CT0383	methyltransferase	NP_661287			+	+	+		+		+	+				
CT0759	hypothetical protein	NP_661654	+	+	+	+	+	+	+	+	+	+	+			
CT0890	methyltransferase	NP_661783	+	+	+	+	+	+	+	+			+			
CT0964	methyltransferase	NP_661857	+	+	+	+	+	+	+	+	+	+	+			
CT1040	methyltransferase	NP_661931	+	+	+	+	+	+	+	+	+	+	+	+	+	
CT1203	hypothetical protein	NP_662094							+			+				
CT1213	hypothetical protein	NP_662103	+	+	+	+	+	+	+	+	+	+	+	+	+	
CT1637	hypothetical protein	NP_662520	+	+	+	+	+	+	+	+	+	+	+			
CT1678	hypothetical protein	NP_662561	+	+	+	+	+	+	+		+	+				
CT1909	hypothetical protein	NP_662786														
CT2121	methyltransferase	NP_662995		+		+	+				+					
CT0548	glycosyl transferase family protein	NP_661448	+	+	+	+	+	+	+	+	+	+	+	+	+	BshA homolog
CT1419	hypothetical protein	NP_662305	+	+	+	+	+	+	+	+	+	+	+	+	+	BshB homolog
CT1558	hypothetical protein	NP_662441	+	+	+	+	+	+	+	+	+	+	+	+	+	BshC homolog
