# Supplementary material for: Physiological Studies of Chlorobiaceae Suggest that Bacillithiol Derivatives Are the Most Widespread Thiols in Bacteria
Source: mBio. 2018 Nov 27;9(6):e01603-18. doi: 10.1128/mBio.01603-18 (PMC6282198; doi:10.1128/mBio.01603-18)
Supplement: DATA SET S1 [file mbo006184195sd1.pdf]

Dataset S1

Metadata for genomes containing orthologs of the indicated LMW thiol biosynthetic genes identified as described in the Methods and Materials . These data were analyzed to infer the presence of a complete biosynthetic pathway for BSH (orange cells), N-Me-BSH (green cells), glutathione (blue cells), and mycothiol (magenta cells).

Inferred BSH synthesis: genomes encoding orthologs of CT0548/bshA + CT1419/bshB + CT1558/bshC

taxon_oid	Genome Name / Sample Name	Phylum	Class	Order	Family	Genus	Species	Genome Size	Gene Count
643692001	Acidobacterium capsulatum ATCC 51196	Acidobacteria	Acidobacteriia	Acidobacteriales	Acidobacteriaceae	Acidobacterium	Acidobacterium capsulatum	4127356	3425
637000001	Candidatus Koribacter versatilis Ellin345	Acidobacteria	Acidobacteriia	Acidobacteriales	Acidobacteriaceae	Candidatus Koribacter	Candidatus Koribacter versatilis	5650368	4837
2503982037	Granulicella mallensis MP5ACTX8	Acidobacteria	Acidobacteriia	Acidobacteriales	Acidobacteriaceae	Granulicella	Granulicella mallensis	6237577	4960
649633002	Granulicella tundricola MP5ACTX9	Acidobacteria	Acidobacteriia	Acidobacteriales	Acidobacteriaceae	Granulicella	Granulicella tundricola	5503984	4757
639633060	Candidatus Solibacter usitatus Ellin6076	Acidobacteria	Solibacteres	Solibacterales	Solibacteraceae	Candidatus Solibacter	Candidatus Solibacter usitatus	9965640	8003
2512047033	Candidatus *Chloracidobacterium thermophilum* B	Acidobacteria	unclassified	unclassified	unclassified	Candidatus Chloracidobacterium	Candidatus *Chloracidobacterium thermophilum*	3695372	3103
2509276058	Belliella baltica BA134, DSM 15883	Bacteroidetes	Cytophagia	Cytophagales	Cyclobacteriaceae	Belliella	Belliella baltica	4196595	3879
2506381023	Cyclobacterium marinum Raj, DSM 745	Bacteroidetes	Cytophagia	Cytophagales	Cyclobacteriaceae	Cyclobacterium	Cyclobacterium marinum	6221273	5151
2509276020	Echinicola vietnamensis KMM 6221, DSM 17526	Bacteroidetes	Cytophagia	Cytophagales	Cyclobacteriaceae	Echinicola	Echinicola vietnamensis	5608040	4684
637000087	Cytophaga hutchinsonii ATCC 33406	Bacteroidetes	Cytophagia	Cytophagales	Cytophagaceae	Cytophaga	Cytophaga hutchinsonii	4433218	3843
644736356	Dyadobacter fermentans NS114, DSM 18053	Bacteroidetes	Cytophagia	Cytophagales	Cytophagaceae	Dyadobacter	Dyadobacter fermentans	6967790	5854
2506485001	Emticicia oligotrophica GPTSA100-15, DSM 17448	Bacteroidetes	Cytophagia	Cytophagales	Cytophagaceae	Emticicia	Emticicia oligotrophica	5220361	4365
2509276059	Flexibacter litoralis Fx l1, DSM 6794	Bacteroidetes	Cytophagia	Cytophagales	Cytophagaceae	Flexibacter	Flexibacter litoralis	4919337	4090
2588253767	Hymenobacter sp. APR13	Bacteroidetes	Cytophagia	Cytophagales	Cytophagaceae	Hymenobacter	Hymenobacter sp. APR13	5010594	4303
2576861456	Hymenobacter swuensis DY53	Bacteroidetes	Cytophagia	Cytophagales	Cytophagaceae	Hymenobacter	Hymenobacter swuensis	5252070	4668
649633063	Leadbetterella byssophila 4M15, DSM 17132	Bacteroidetes	Cytophagia	Cytophagales	Cytophagaceae	Leadbetterella	Leadbetterella byssophila	4059653	3665
2505679030	Runella slithyformis LSU4, DSM 19594	Bacteroidetes	Cytophagia	Cytophagales	Cytophagaceae	Runella	Runella slithyformis	6919729	6025
646311954	Spirosoma linguale DSM 74	Bacteroidetes	Cytophagia	Cytophagales	Cytophagaceae	Spirosoma	Spirosoma linguale	8491258	7130
649633065	Marivirga tractuosa H-43, DSM 4126	Bacteroidetes	Cytophagia	Cytophagales	Flammeovirgaceae	Marivirga	Marivirga tractuosa	4516490	3857
646311949	Rhodothermus marinus R-10, DSM 4252	Bacteroidetes	Cytophagia	Rhodothermales	Rhodothermaceae	Rhodothermus	marinus	3386737	2965
2503707007	Fluviicola taffensis RW262, DSM 16823	Bacteroidetes	Flavobacteriia	Flavobacteriales	Cryomorphaceae	Fluviicola	Fluviicola taffensis	4633577	4131
2508501098	Owenweeksia hongkongensis DSM 17368	Bacteroidetes	Flavobacteriia	Flavobacteriales	Cryomorphaceae	Owenweeksia	Owenweeksia hongkongensis	4000057	3563
2509276063	Aequorivita sublithincola QSSC9-3, DSM 14238	Bacteroidetes	Flavobacteriia	Flavobacteriales	Flavobacteriaceae	Aequorivita	Aequorivita sublithincola	3520671	3242
650716019	Capnocytophaga canimorsus Cc5	Bacteroidetes	Flavobacteriia	Flavobacteriales	Flavobacteriaceae	Capnocytophaga	Capnocytophaga canimorsus	2571406	2469
644736338	Capnocytophaga ochracea VPI 2845, DSM 7271	Bacteroidetes	Flavobacteriia	Flavobacteriales	Flavobacteriaceae	Capnocytophaga	Capnocytophaga ochracea	2612925	2252
649633031	Cellulophaga algicola IC166, DSM 14237	Bacteroidetes	Flavobacteriia	Flavobacteriales	Flavobacteriaceae	Cellulophaga	Cellulophaga algicola	4888353	4344
649633032	Cellulophaga lytica LIM-21, DSM 7489	Bacteroidetes	Flavobacteriia	Flavobacteriales	Flavobacteriaceae	Cellulophaga	Cellulophaga lytica	3765936	3358
2597490179	Cellulophaga lytica HI1	Bacteroidetes	Flavobacteriia	Flavobacteriales	Flavobacteriaceae	Cellulophaga	Cellulophaga lytica	3824196	3378
2526164557	Croceibacter atlanticus HTCC2559 (Re-annotation of existing genome that was not originally submitted by me)	Bacteroidetes	Flavobacteriia	Flavobacteriales	Flavobacteriaceae	Croceibacter	Croceibacter atlanticus	2952962	2727
650716043	Krokinobacter diaphorus 4H-3-7-5	Bacteroidetes	Flavobacteriia	Flavobacteriales	Flavobacteriaceae	Dokdonia	Dokdonia diaphoros	3389993	3048
2597489917	Dokdonia sp. PRO95	Bacteroidetes	Flavobacteriia	Flavobacteriales	Flavobacteriaceae	Dokdonia	Dokdonia sp. PRO95	3305093	3038
2561511155	Flavobacterium branchiophilum FL-15	Bacteroidetes	Flavobacteriia	Flavobacteriales	Flavobacteriaceae	Flavobacterium	Flavobacterium branchiophilum	3563292	3087
2511231122	Flavobacterium columnare ATCC 49512	Bacteroidetes	Flavobacteriia	Flavobacteriales	Flavobacteriaceae	Flavobacterium	Flavobacterium columnare	3162865	2735
2540341066	Flavobacterium indicum GPTSA100-9	Bacteroidetes	Flavobacteriia	Flavobacteriales	Flavobacteriaceae	Flavobacterium	Flavobacterium indicum	2993089	2790
644736369	Flavobacterium johnsoniae UW101, ATCC 17061	Bacteroidetes	Flavobacteriia	Flavobacteriales	Flavobacteriaceae	Flavobacterium	Flavobacterium johnsoniae	6096872	5099
640753027	Flavobacterium psychrophilum JIP02/86	Bacteroidetes	Flavobacteriia	Flavobacteriales	Flavobacteriaceae	Flavobacterium	Flavobacterium psychrophilum	2861988	2505
2597490156	Flavobacterium psychrophilum CSF259-93	Bacteroidetes	Flavobacteriia	Flavobacteriales	Flavobacteriaceae	Flavobacterium	Flavobacterium psychrophilum	2900735	2718
2623620349	Flavobacterium psychrophilum JIP02/86 (re-annotation)	Bacteroidetes	Flavobacteriia	Flavobacteriales	Flavobacteriaceae	Flavobacterium	Flavobacterium psychrophilum	2860382	2702
639633025	Gramella forsetii KT0803	Bacteroidetes	Flavobacteriia	Flavobacteriales	Flavobacteriaceae	Gramella	Gramella forsetii	3798465	3642
2623620203	Gramella forsetii KT0803 (re-annotation)	Bacteroidetes	Flavobacteriia	Flavobacteriales	Flavobacteriaceae	Gramella	Gramella forsetii	3798465	3507
650716044	Lacinutrix sp. 5H-3-7-4	Bacteroidetes	Flavobacteriia	Flavobacteriales	Flavobacteriaceae	Lacinutrix	Lacinutrix sp. 5H-3-7-4	3296168	3026
648028027	Maribacter sp. HTCC2170	Bacteroidetes	Flavobacteriia	Flavobacteriales	Flavobacteriaceae	Maribacter	Maribacter sp. HTCC2170	3868304	3455
2505679007	Muricauda ruestringensis B1, DSM 13258	Bacteroidetes	Flavobacteriia	Flavobacteriales	Flavobacteriaceae	Muricauda	Muricauda ruestringensis	3842422	3525
2540341128	Donghaeana dokdonensis DSW-6	Bacteroidetes	Flavobacteriia	Flavobacteriales	Flavobacteriaceae	Nonlabens	Nonlabens dokdonensis	3914632	3666

2509601001	Ornithobacterium rhinotracheale DSM 15997	Bacteroidetes	Flavobacteriia	Flavobacteriales	Flavobacteriaceae	Ornithobacterium	Ornithobacterium rhinotracheale	2399175	2371
638341218	Polaribacter sp. MED152	Bacteroidetes	Flavobacteriia	Flavobacteriales	Flavobacteriaceae	Polaribacter	Polaribacter sp. MED152	2967100	2723
2623620847	Polaribacter sp. MED152 (re-annotation)	Bacteroidetes	Flavobacteriia	Flavobacteriales	Flavobacteriaceae	Polaribacter	Polaribacter sp. MED152	2961474	2699
649633092	Riemerella anatipestifer A350/72, DSM 15868	Bacteroidetes	Flavobacteriia	Flavobacteriales	Flavobacteriaceae	Riemerella	Riemerella anatipestifer	2155121	2052
651053063	Riemerella anatipestifer RA-GD	Bacteroidetes	Flavobacteriia	Flavobacteriales	Flavobacteriaceae	Riemerella	Riemerella anatipestifer	2166384	2036
2512564079	Riemerella anatipestifer A350/72, DSM 15868	Bacteroidetes	Flavobacteriia	Flavobacteriales	Flavobacteriaceae	Riemerella	Riemerella anatipestifer	2164087	2087
2518645536	Riemerella anatipestifer RA-CH-1	Bacteroidetes	Flavobacteriia	Flavobacteriales	Flavobacteriaceae	Riemerella	Riemerella anatipestifer	2309519	2237
2521172679	Riemerella anatipestifer RA-CH-2	Bacteroidetes	Flavobacteriia	Flavobacteriales	Flavobacteriaceae	Riemerella	Riemerella anatipestifer	2166321	2095
2588253739	Riemerella anatipestifer CH3	Bacteroidetes	Flavobacteriia	Flavobacteriales	Flavobacteriaceae	Riemerella	Riemerella anatipestifer	2234477	2231
646311950	Robiginitalea biformata HTCC2501	Bacteroidetes	Flavobacteriia	Flavobacteriales	Flavobacteriaceae	Robiginitalea	Robiginitalea biformata	3530383	3255
644736368	Flavobacteriaceae bacterium 3519-10	Bacteroidetes	Flavobacteriia	Flavobacteriales	Flavobacteriaceae	unclassified	Flavobacteriaceae bacterium 3519-10	2768102	2582
650377986	Weeksella virosa 9751, DSM 16922	Bacteroidetes	Flavobacteriia	Flavobacteriales	Flavobacteriaceae	Weeksella	Weeksella virosa	2272954	2125
2619619092	Zobellia galactanivorans DsijT	Bacteroidetes	Flavobacteriia	Flavobacteriales	Flavobacteriaceae	Zobellia	Zobellia galactanivorans	5521712	4563
646564591	Zunongwangia profunda SM-A87	Bacteroidetes	Flavobacteriia	Flavobacteriales	Flavobacteriaceae	Zunongwangia	Zunongwangia profunda	5128187	4709
644736340	Chitinophaga pinensis UQM 2034, DSM 2588	Bacteroidetes	Sphingobacteriia	Sphingobacteriales	Chitinophagaceae	Chitinophaga	Chitinophaga pinensis	9127347	7396
2506783006	Niabella soli JS13-8, DSM 19437	Bacteroidetes	Sphingobacteriia	Sphingobacteriales	Chitinophagaceae	Niabella	Niabella soli	4697343	3931
2506520016	Niastella koreensis GR20-10, DSM 17620	Bacteroidetes	Sphingobacteriia	Sphingobacteriales	Chitinophagaceae	Niastella	Niastella koreensis	9033684	7444
2504756004	Haliscomenobacter hydrossis O, DSM 1100	Bacteroidetes	Sphingobacteriia	Sphingobacteriales	Saprospiraceae	Haliscomenobacter	Haliscomenobacter hydrossis	8771651	6918
2512564032	Saprospira grandis Lewin	Bacteroidetes	Sphingobacteriia	Sphingobacteriales	Saprospiraceae	Saprospira	Saprospira grandis	4400185	4324
644736398	Pedobacter heparinus HIM 762-3	Bacteroidetes	Sphingobacteriia	Sphingobacteriales	Sphingobacteriaceae	Pedobacter	Pedobacter heparinus	5167383	4339
649633082	Pedobacter saltans Steyn 113, DSM 12145	Bacteroidetes	Sphingobacteriia	Sphingobacteriales	Sphingobacteriaceae	Pedobacter	Pedobacter saltans	4635236	3921
2509276062	Solitalea canadensis USAM 9D, DSM 3403	Bacteroidetes	Sphingobacteriia	Sphingobacteriales	Sphingobacteriaceae	Solitalea	Solitalea canadensis	5202069	4490
2505679080	Sphingobacterium sp. 21	Bacteroidetes	Sphingobacteriia	Sphingobacteriales	Sphingobacteriaceae	Sphingobacterium	Sphingobacterium sp. 21	6226409	5340
2597490280	Sphingobacterium sp. ML3W	Bacteroidetes	Sphingobacteriia	Sphingobacteriales	Sphingobacteriaceae	Sphingobacterium	Sphingobacterium sp. ML3W	5329010	4669
2505679078	Rhodothermus marinus SG0.5JP17-172	Bacteroidetes	unclassified	Bacteroidetes Order II. Incertae sedis	Rhodothermaceae	Rhodothermus	Rhodothermus marinus	3334122	2968
637000250	Salinibacter ruber M31, DSM 13855	Bacteroidetes	unclassified	Bacteroidetes Order II. Incertae sedis	Rhodothermaceae	Salinibacter	Salinibacter ruber	3587328	2899
2561511240	Salinibacter ruber M8	Bacteroidetes	unclassified	Bacteroidetes Order II. Incertae sedis	Rhodothermaceae	Salinibacter	Salinibacter ruber	3832918	3302
2623620645	Salinibacter ruber M31, DSM 13855 (re-annotation)	Bacteroidetes	unclassified	Bacteroidetes Order II. Incertae sedis	Rhodothermaceae	Salinibacter	Salinibacter ruber	3587328	3086
646564588	Waddlia chondrophila WSU 86-1044	Chlamydiae	Chlamydiia	Chlamydiales	Waddliaceae	Waddlia	Waddlia chondrophila	2131905	1999
642555120	Chlorobaculum parvum DSM 263	Chlorobi	Chlorobia	Chlorobiales	Chlorobiaceae	Chlorobaculum	Chlorobaculum parvum	2289249	2133
637000073	*Chlorobium tepidum* TLS	Chlorobi	Chlorobia	Chlorobiales	Chlorobiaceae	Chlorobaculum	*Chlorobaculum tepidum*	2154946	2317
637000072	Chlorobium chlorochromatii CaD3	Chlorobi	Chlorobia	Chlorobiales	Chlorobiaceae	Chlorobium	Chlorobium chlorochromatii	2572079	2100
642555121	Chlorobium limicola DSM 245	Chlorobi	Chlorobia	Chlorobiales	Chlorobiaceae	Chlorobium	Chlorobium limicola	2763181	2576
639633020	Chlorobium phaeobacteroides DSM 266	Chlorobi	Chlorobia	Chlorobiales	Chlorobiaceae	Chlorobium	Chlorobium phaeobacteroides	3133902	2805
642555122	Chlorobium phaeobacteroides BS1	Chlorobi	Chlorobia	Chlorobiales	Chlorobiaceae	Chlorobium	Chlorobium phaeobacteroides	2736403	2611
640427130	Chlorobium phaeovibrioides DSM 265	Chlorobi	Chlorobia	Chlorobiales	Chlorobiaceae	Chlorobium	Chlorobium phaeovibrioides	1966858	1831
642555123	Chloroherpeton thalassium ATCC 35110	Chlorobi	Chlorobia	Chlorobiales	Chlorobiaceae	Chloroherpeton	Chloroherpeton thalassium	3293456	2778
637000205	Pelodictyon luteolum DSM 273	Chlorobi	Chlorobia	Chlorobiales	Chlorobiaceae	Pelodictyon	Pelodictyon luteolum	2364842	2146
642555146	Pelodictyon phaeoclathratiforme BU-1	Chlorobi	Chlorobia	Chlorobiales	Chlorobiaceae	Pelodictyon	Pelodictyon phaeoclathratiforme	3018238	2969
642555149	Prosthecochloris aestuarii SK413, DSM 271	Chlorobi	Chlorobia	Chlorobiales	Chlorobiaceae	Prosthecochloris	Prosthecochloris aestuarii	2579695	2451
643692020	Deinococcus deserti VCD115	Deinococcus-Thermus	Deinococci	Deinococcales	Deinococcaceae	Deinococcus	Deinococcus deserti	3855329	3511
649633035	Deinococcus proteolyticus MRP, DSM 20540	Deinococcus-Thermus	Deinococci	Deinococcales	Deinococcaceae	Deinococcus	Deinococcus proteolyticus	2886836	2799
646564586	Truepera radiovictrix RQ-24, DSM 17093	Deinococcus-Thermus	Deinococci	Deinococcales	Trueperaceae	Truepera	Truepera radiovictrix	3260398	3046
2504643006	Marinithermus hydrothermalis T1, DSM 14884	Deinococcus-Thermus	Deinococci	Thermales	Thermaceae	Marinithermus	Marinithermus hydrothermalis	2269167	2310
646564546	Meiothermus silvanus VI-R2, DSM 9946	Deinococcus-Thermus	Deinococci	Thermales	Thermaceae	Meiothermus	Meiothermus silvanus	3721669	3720
649633077	Oceanithermus profundus 506, DSM 14977	Deinococcus-Thermus	Deinococci	Thermales	Thermaceae	Oceanithermus	Oceanithermus profundus	2439291	2445
643348508	Anoxybacillus flavithermus WK1, DSM 2641	Firmicutes	Bacilli	Bacillales	Bacillaceae	Anoxybacillus	Anoxybacillus flavithermus	2846746	2933

640753005	Bacillus amyloliquefaciens plantarum FZB42	Firmicutes	Bacilli	Bacillales	Bacillaceae	Bacillus	Bacillus amyloliquefaciens	3918589	3814
649633008	Bacillus amyloliquefaciens Campbell F, DSM 7	Firmicutes	Bacilli	Bacillales	Bacillaceae	Bacillus	Bacillus amyloliquefaciens	3980199	4046
651053001	Bacillus amyloliquefaciens LL3	Firmicutes	Bacilli	Bacillales	Bacillaceae	Bacillus	Bacillus amyloliquefaciens	4001985	4322
651053002	Bacillus amyloliquefaciens TA208	Firmicutes	Bacilli	Bacillales	Bacillaceae	Bacillus	Bacillus amyloliquefaciens	3937511	4177
2511231119	Bacillus amyloliquefaciens CAU-B946	Firmicutes	Bacilli	Bacillales	Bacillaceae	Bacillus	Bacillus amyloliquefaciens	4019861	3948
2511231180	Bacillus amyloliquefaciens XH7	Firmicutes	Bacilli	Bacillales	Bacillaceae	Bacillus	Bacillus amyloliquefaciens	3939203	4286
2513237190	Bacillus amyloliquefaciens plantarum YAU B9601-Y2	Firmicutes	Bacilli	Bacillales	Bacillaceae	Bacillus	Bacillus amyloliquefaciens	4242774	4110
2519899543	Bacillus amyloliquefaciens plantarum AS43.3	Firmicutes	Bacilli	Bacillales	Bacillaceae	Bacillus	Bacillus amyloliquefaciens	3961368	3979
2540341129	Bacillus amyloliquefaciens Y2	Firmicutes	Bacilli	Bacillales	Bacillaceae	Bacillus	Bacillus amyloliquefaciens	4238624	4352
2541047004	Bacillus amyloliquefaciens Plantarum UCMB5113	Firmicutes	Bacilli	Bacillales	Bacillaceae	Bacillus	Bacillus amyloliquefaciens	3889532	3854
2541047086	Bacillus amyloliquefaciens plantarum UCMB5033	Firmicutes	Bacilli	Bacillales	Bacillaceae	Bacillus	Bacillus amyloliquefaciens	4071167	4095
2558309054	Bacillus amyloliquefaciens CC178	Firmicutes	Bacilli	Bacillales	Bacillaceae	Bacillus	Bacillus amyloliquefaciens	3916828	4074
2558309083	Bacillus amyloliquefaciens LFB112	Firmicutes	Bacilli	Bacillales	Bacillaceae	Bacillus	Bacillus amyloliquefaciens	3942754	4023
2563366559	Bacillus amyloliquefaciens IT-45	Firmicutes	Bacilli	Bacillales	Bacillaceae	Bacillus	Bacillus amyloliquefaciens	3936866	3985
2563366598	Bacillus amyloliquefaciens TrigoCor1448	Firmicutes	Bacilli	Bacillales	Bacillaceae	Bacillus	Bacillus amyloliquefaciens	3957904	3857
2576861459	Bacillus amyloliquefaciens SQR9	Firmicutes	Bacilli	Bacillales	Bacillaceae	Bacillus	Bacillus amyloliquefaciens	4117023	4171
2623620239	Bacillus amyloliquefaciens plantarum FZB42 (re-annotation)	Firmicutes	Bacilli	Bacillales	Bacillaceae	Bacillus	Bacillus amyloliquefaciens	3918589	3879
637000013	Bacillus anthracis Ames Ancestor A2084	Firmicutes	Bacilli	Bacillales	Bacillaceae	Bacillus	Bacillus anthracis	5503926	5853
637000014	Bacillus anthracis Ames	Firmicutes	Bacilli	Bacillales	Bacillaceae	Bacillus	Bacillus anthracis	5227293	5545
637000015	Bacillus anthracis Sterne	Firmicutes	Bacilli	Bacillales	Bacillaceae	Bacillus	Bacillus anthracis	5228663	5521
643692005	Bacillus anthracis A0248	Firmicutes	Bacilli	Bacillales	Bacillaceae	Bacillus	Bacillus anthracis	5503926	5418
643692006	Bacillus anthracis CDC 684	Firmicutes	Bacilli	Bacillales	Bacillaceae	Bacillus	Bacillus anthracis	5506763	6031
2513237215	Bacillus anthracis H9401	Firmicutes	Bacilli	Bacillales	Bacillaceae	Bacillus	Bacillus anthracis	5495471	5917
2558860177	Bacillus anthracis A16R	Firmicutes	Bacilli	Bacillales	Bacillaceae	Bacillus	Bacillus anthracis	5409446	6046
2558860236	Bacillus anthracis A16	Firmicutes	Bacilli	Bacillales	Bacillaceae	Bacillus	Bacillus anthracis	5504501	6099
2563366594	Bacillus anthracis SVA11	Firmicutes	Bacilli	Bacillales	Bacillaceae	Bacillus	Bacillus anthracis	5487517	5905
2588253743	Bacillus anthracis HYU01	Firmicutes	Bacilli	Bacillales	Bacillaceae	Bacillus	Bacillus anthracis	5490124	5617
2597489971	Bacillus anthracis 8903-G	Firmicutes	Bacilli	Bacillales	Bacillaceae	Bacillus	Bacillus anthracis	5504767	5838
2597489974	Bacillus anthracis 9080-G	Firmicutes	Bacilli	Bacillales	Bacillaceae	Bacillus	Bacillus anthracis	5508663	5879
2597489977	Bacillus anthracis 52-G	Firmicutes	Bacilli	Bacillales	Bacillaceae	Bacillus	Bacillus anthracis	5504444	5837
2597490031	Bacillus anthracis BFV	Firmicutes	Bacilli	Bacillales	Bacillaceae	Bacillus	Bacillus anthracis	5504355	5856
2597490107	Bacillus anthracis delta Sterne	Firmicutes	Bacilli	Bacillales	Bacillaceae	Bacillus	Bacillus anthracis	5226650	5582
2597490108	Bacillus anthracis STI	Firmicutes	Bacilli	Bacillales	Bacillaceae	Bacillus	Bacillus anthracis	5409096	5734
2597490111	Bacillus anthracis Vollum	Firmicutes	Bacilli	Bacillales	Bacillaceae	Bacillus	Bacillus anthracis	5506189	5840
2597490114	Bacillus anthracis 2000031021	Firmicutes	Bacilli	Bacillales	Bacillaceae	Bacillus	Bacillus anthracis	5331737	5602
2597490165	Bacillus anthracis Han	Firmicutes	Bacilli	Bacillales	Bacillaceae	Bacillus	Bacillus anthracis	5225433	5892
2597490166	Bacillus anthracis Cvac02	Firmicutes	Bacilli	Bacillales	Bacillaceae	Bacillus	Bacillus anthracis	5227170	5754
2623620891	Bacillus anthracis Ames Ancestor A2084 (re-annotation)	Firmicutes	Bacilli	Bacillales	Bacillaceae	Bacillus	Bacillus anthracis	5503926	5896
2623620892	Bacillus anthracis Ames (re-annotation)	Firmicutes	Bacilli	Bacillales	Bacillaceae	Bacillus	Bacillus anthracis	5227293	5613
649633009	Bacillus atrophaeus 1942	Firmicutes	Bacilli	Bacillales	Bacillaceae	Bacillus	Bacillus atrophaeus	4168266	4282
2597490137	Bacillus atrophaeus globigii BSS	Firmicutes	Bacilli	Bacillales	Bacillaceae	Bacillus	Bacillus atrophaeus	4174560	4207
649633010	Bacillus cellulosilyticus N-4, DSM 2522	Firmicutes	Bacilli	Bacillales	Bacillaceae	Bacillus	Bacillus cellulosilyticus	4681672	4443
637000016	Bacillus cereus ATCC 10987	Firmicutes	Bacilli	Bacillales	Bacillaceae	Bacillus	Bacillus cereus	5432652	6126
637000017	Bacillus cereus ATCC 14579	Firmicutes	Bacilli	Bacillales	Bacillaceae	Bacillus	Bacillus cereus	5427083	5513
637000018	Bacillus cereus E33L (ZK)	Firmicutes	Bacilli	Bacillales	Bacillaceae	Bacillus	Bacillus cereus	5843235	5886
643348510	Bacillus cereus AH187 (F4810/72)	Firmicutes	Bacilli	Bacillales	Bacillaceae	Bacillus	Bacillus cereus	5599857	5903
643348511	Bacillus cereus AH820	Firmicutes	Bacilli	Bacillales	Bacillaceae	Bacillus	Bacillus cereus	5588834	5941
643348512	Bacillus cereus B4264	Firmicutes	Bacilli	Bacillales	Bacillaceae	Bacillus	Bacillus cereus	5419036	5557
643348513	Bacillus cereus G9842	Firmicutes	Bacilli	Bacillales	Bacillaceae	Bacillus	Bacillus cereus	5736823	5994
643348514	Bacillus cereus Q1	Firmicutes	Bacilli	Bacillales	Bacillaceae	Bacillus	Bacillus cereus	5506207	5621
643692007	Bacillus cereus 03BB102	Firmicutes	Bacilli	Bacillales	Bacillaceae	Bacillus	Bacillus cereus	5449308	5767
648028007	Bacillus cereus bv. anthracis CI	Firmicutes	Bacilli	Bacillales	Bacillaceae	Bacillus	Bacillus cereus	5486649	5723
2511231081	Bacillus cereus NC7401	Firmicutes	Bacilli	Bacillales	Bacillaceae	Bacillus	Bacillus cereus	5552031	5900
2513237193	Bacillus cereus F837/76	Firmicutes	Bacilli	Bacillales	Bacillaceae	Bacillus	Bacillus cereus	5288498	5609
2561511153	Bacillus cereus FRI-35	Firmicutes	Bacilli	Bacillales	Bacillaceae	Bacillus	Bacillus cereus	5382319	5580

2623620893	Bacillus cereus ATCC 10987 (re-annotation)	Firmicutes	Bacilli	Bacillales	Bacillaceae	Bacillus	Bacillus cereus	5432652	5650
2623620894	Bacillus cereus ATCC 14579 (re-annotation)	Firmicutes	Bacilli	Bacillales	Bacillaceae	Bacillus	Bacillus cereus	5427083	5701
637000019	Bacillus clausii KSM-K16	Firmicutes	Bacilli	Bacillales	Bacillaceae	Bacillus	Bacillus clausii	4303871	4261
2623620997	Bacillus clausii KSM-K16 (re-annotation)	Firmicutes	Bacilli	Bacillales	Bacillaceae	Bacillus	Bacillus clausii	4303871	4420
650716010	Bacillus coagulans 2-6	Firmicutes	Bacilli	Bacillales	Bacillaceae	Bacillus	Bacillus coagulans	3073079	3052
2503242012	Bacillus coagulans 36D1, ATCC PTA-5827 (Bacillus coagulans 36D1 UF finished)	Firmicutes	Bacilli	Bacillales	Bacillaceae	Bacillus	Bacillus coagulans	3552226	3449
640753006	Bacillus cereus cytotoxis NVH 391-98	Firmicutes	Bacilli	Bacillales	Bacillaceae	Bacillus	Bacillus cytotoxicus	4094159	4250
637000020	Bacillus halodurans C-125	Firmicutes	Bacilli	Bacillales	Bacillaceae	Bacillus	Bacillus halodurans	4202352	4239
2623620895	Bacillus halodurans C-125 (re-annotation)	Firmicutes	Bacilli	Bacillales	Bacillaceae	Bacillus	Bacillus halodurans	4202352	4225
2585427654	Bacillus lehensis G1	Firmicutes	Bacilli	Bacillales	Bacillaceae	Bacillus	Bacillus lehensis	3993073	4108
639279303	Bacillus licheniformis DSM 13 Goettingen	Firmicutes	Bacilli	Bacillales	Bacillaceae	Bacillus	Bacillus licheniformis	4222645	4356
639279304	Bacillus licheniformis DSM 13 Novozymes	Firmicutes	Bacilli	Bacillales	Bacillaceae	Bacillus	Bacillus licheniformis	4222334	4420
2551306715	Bacillus licheniformis 9945A	Firmicutes	Bacilli	Bacillales	Bacillaceae	Bacillus	Bacillus licheniformis	4376305	4341
2623620896	Bacillus licheniformis DSM 13 Goettingen (re-annotation)	Firmicutes	Bacilli	Bacillales	Bacillaceae	Bacillus	Bacillus licheniformis	4222645	4419
2623620897	Bacillus licheniformis DSM 13 Novozymes (re-annotation)	Firmicutes	Bacilli	Bacillales	Bacillaceae	Bacillus	Bacillus licheniformis	4222597	4420
646564507	Bacillus megaterium DSM 319	Firmicutes	Bacilli	Bacillales	Bacillaceae	Bacillus	Bacillus megaterium	5097447	5248
646564508	Bacillus megaterium QM B1551	Firmicutes	Bacilli	Bacillales	Bacillaceae	Bacillus	Bacillus megaterium	5523192	5788
2511231161	Bacillus megaterium WSH-002	Firmicutes	Bacilli	Bacillales	Bacillaceae	Bacillus	Bacillus megaterium	5075293	5493
2585427643	Bacillus methanolicus MGA3	Firmicutes	Bacilli	Bacillales	Bacillaceae	Bacillus	Bacillus methanolicus	3425208	3424
2597490127	Bacillus mycoides 219298	Firmicutes	Bacilli	Bacillales	Bacillaceae	Bacillus	Bacillus mycoides	5675302	5787
2623621012	Bacillus mycoides ATCC 6462	Firmicutes	Bacilli	Bacillales	Bacillaceae	Bacillus	Bacillus mycoides	5637053	5851
646311908	Bacillus pseudofirmus OF4	Firmicutes	Bacilli	Bacillales	Bacillaceae	Bacillus	Bacillus pseudofirmus	4249248	4434
640753007	*Bacillus pumilus* SAFR-032	Firmicutes	Bacilli	Bacillales	Bacillaceae	Bacillus	*Bacillus pumilus*	3704465	3823
2565956572	*Bacillus pumilus* B6033	Firmicutes	Bacilli	Bacillales	Bacillaceae	Bacillus	*Bacillus pumilus*	3763493	3807
2623620368	*Bacillus pumilus* SAFR-032 (re-annotation)	Firmicutes	Bacilli	Bacillales	Bacillaceae	Bacillus	*Bacillus pumilus*	3704465	3816
2506520047	Bacillus sp. 1NLA3E	Firmicutes	Bacilli	Bacillales	Bacillaceae	Bacillus	Bacillus sp. 1NLA3E	4815602	4833
2513237216	Bacillus sp. JS	Firmicutes	Bacilli	Bacillales	Bacillaceae	Bacillus	Bacillus sp. JS	4120406	4363
2554235446	Bacillus infantis NRRL B-14911	Firmicutes	Bacilli	Bacillales	Bacillaceae	Bacillus	Bacillus sp. NRRL B-14911	4884713	5179
646311909	*Bacillus subtilis* subtilis 168	Firmicutes	Bacilli	Bacillales	Bacillaceae	Bacillus	*Bacillus subtilis*	4215606	4354
648028008	*Bacillus subtilis* spizizenii W23	Firmicutes	Bacilli	Bacillales	Bacillaceae	Bacillus	*Bacillus subtilis*	4027676	4168
649633011	*Bacillus subtilis* BSn5	Firmicutes	Bacilli	Bacillales	Bacillaceae	Bacillus	*Bacillus subtilis*	4093599	4258
650377907	*Bacillus subtilis* natto BEST195	Firmicutes	Bacilli	Bacillales	Bacillaceae	Bacillus	*Bacillus subtilis*	4097429	4508
2511231064	*Bacillus subtilis* spizizenii TU-B-10, DSM 15029	Firmicutes	Bacilli	Bacillales	Bacillaceae	Bacillus	*Bacillus subtilis*	4207222	4475
2511231173	*Bacillus subtilis* subtilis RO-NN-1	Firmicutes	Bacilli	Bacillales	Bacillaceae	Bacillus	*Bacillus subtilis*	4011949	4257
2518645535	*Bacillus subtilis* QB928	Firmicutes	Bacilli	Bacillales	Bacillaceae	Bacillus	*Bacillus subtilis*	4146839	4236
2521172700	*Bacillus subtilis* subtilis BSP1	Firmicutes	Bacilli	Bacillales	Bacillaceae	Bacillus	*Bacillus subtilis*	4043754	3948
2540341072	*Bacillus subtilis* subtilis 6051-HGW	Firmicutes	Bacilli	Bacillales	Bacillaceae	Bacillus	*Bacillus subtilis*	4215610	4337
2540341094	*Bacillus subtilis* XF-1	Firmicutes	Bacilli	Bacillales	Bacillaceae	Bacillus	*Bacillus subtilis*	4061186	3957
2540341124	*Bacillus subtilis* BEST7613	Firmicutes	Bacilli	Bacillales	Bacillaceae	Bacillus	*Bacillus subtilis*	7585470	7430
2554235394	*Bacillus subtilis* subtilis BAB-1	Firmicutes	Bacilli	Bacillales	Bacillaceae	Bacillus	*Bacillus subtilis*	4021944	4119
2558309080	*Bacillus subtilis* PY79	Firmicutes	Bacilli	Bacillales	Bacillaceae	Bacillus	*Bacillus subtilis*	4033459	4257
2558860255	*Bacillus subtilis* BEST7003	Firmicutes	Bacilli	Bacillales	Bacillaceae	Bacillus	*Bacillus subtilis*	4043042	4133
2585427616	*Bacillus subtilis* subtilis AG1839	Firmicutes	Bacilli	Bacillales	Bacillaceae	Bacillus	*Bacillus subtilis*	4193640	4349
2585427617	*Bacillus subtilis* subtilis JH642 subAG174	Firmicutes	Bacilli	Bacillales	Bacillaceae	Bacillus	*Bacillus subtilis*	4188369	4345
2585427626	*Bacillus subtilis* subtilis OH 131.1	Firmicutes	Bacilli	Bacillales	Bacillaceae	Bacillus	*Bacillus subtilis*	4039155	4061
637000023	Bacillus thuringiensis konkukian sv. H34 97-27	Firmicutes	Bacilli	Bacillales	Bacillaceae	Bacillus	Bacillus thuringiensis	5314794	5452
639633008	Bacillus thuringiensis Al Hakam	Firmicutes	Bacilli	Bacillales	Bacillaceae	Bacillus	Bacillus thuringiensis	5313030	5050
643886171	Bacillus thuringiensis Bt407	Firmicutes	Bacilli	Bacillales	Bacillaceae	Bacillus	Bacillus thuringiensis	6008143	6425
646564510	Bacillus thuringiensis BMB171	Firmicutes	Bacilli	Bacillales	Bacillaceae	Bacillus	Bacillus thuringiensis	5643051	5495
650377908	Bacillus thuringiensis sv. finitimus YBT-020	Firmicutes	Bacilli	Bacillales	Bacillaceae	Bacillus	Bacillus thuringiensis	5682383	5931
651053003	Bacillus thuringiensis sv. chinensis CT-43	Firmicutes	Bacilli	Bacillales	Bacillaceae	Bacillus	Bacillus thuringiensis	6151150	6330
2518645577	Bacillus thuringiensis HD-789	Firmicutes	Bacilli	Bacillales	Bacillaceae	Bacillus	Bacillus thuringiensis	6334630	6626
2521172680	Bacillus thuringiensis HD-771	Firmicutes	Bacilli	Bacillales	Bacillaceae	Bacillus	Bacillus thuringiensis	6438373	6704
2521172701	Bacillus thuringiensis MC28	Firmicutes	Bacilli	Bacillales	Bacillaceae	Bacillus	Bacillus thuringiensis	6694533	6679
2522572172	Bacillus thuringiensis Bt407	Firmicutes	Bacilli	Bacillales	Bacillaceae	Bacillus	Bacillus thuringiensis	6134344	6590

2561511152	Bacillus thuringiensis YBT-1518	Firmicutes	Bacilli	Bacillales	Bacillaceae	Bacillus	Bacillus thuringiensis	6672921	6877
2561511195	Bacillus thuringiensis sv. thuringiensis IS5056	Firmicutes	Bacilli	Bacillales	Bacillaceae	Bacillus	Bacillus thuringiensis	6771593	7046
2561511198	Bacillus thuringiensis sv. kurstaki HD73	Firmicutes	Bacilli	Bacillales	Bacillaceae	Bacillus	Bacillus thuringiensis	5908575	6334
2576861478	Bacillus thuringiensis sv. kurstaki YBT-1520	Firmicutes	Bacilli	Bacillales	Bacillaceae	Bacillus	Bacillus thuringiensis	6580536	6699
2585427662	Bacillus thuringiensis sv. kurstaki HD-1	Firmicutes	Bacilli	Bacillales	Bacillaceae	Bacillus	Bacillus thuringiensis	6766594	6920
2597490277	Bacillus thuringiensis sv. kurstaki YBT-1520	Firmicutes	Bacilli	Bacillales	Bacillaceae	Bacillus	Bacillus thuringiensis	6520413	6757
2558309069	Bacillus toyonensis BCT-7112	Firmicutes	Bacilli	Bacillales	Bacillaceae	Bacillus	Bacillus toyonensis	5025419	5075
641228477	Bacillus weihenstephanensis KBAB4	Firmicutes	Bacilli	Bacillales	Bacillaceae	Bacillus	Bacillus weihenstephanensis	5872743	5983
2623621013	Bacillus weihenstephanensis WSBC 10204	Firmicutes	Bacilli	Bacillales	Bacillaceae	Bacillus	Bacillus weihenstephanensis	5608349	5685
637000118	Geobacillus kaustophilus HTA426	Firmicutes	Bacilli	Bacillales	Bacillaceae	Geobacillus	Geobacillus kaustophilus	3592666	3714
2623620701	Geobacillus kaustophilus HTA426 (re-annotation)	Firmicutes	Bacilli	Bacillales	Bacillaceae	Geobacillus	Geobacillus kaustophilus	3592666	3686
646564535	Geobacillus sp. C56-T3	Firmicutes	Bacilli	Bacillales	Bacillaceae	Geobacillus	Geobacillus sp. C56-T3	3650813	3595
2519103198	Geobacillus sp. GHH01	Firmicutes	Bacilli	Bacillales	Bacillaceae	Geobacillus	Geobacillus sp. GHH01	3583134	3587
2561511211	Geobacillus sp. JF8	Firmicutes	Bacilli	Bacillales	Bacillaceae	Geobacillus	Geobacillus sp. JF8	3485960	3648
644736370	Geobacillus sp. WCH70	Firmicutes	Bacilli	Bacillales	Bacillaceae	Geobacillus	Geobacillus sp. WCH70	3508804	3597
649633047	Geobacillus sp. Y4.1MC1	Firmicutes	Bacilli	Bacillales	Bacillaceae	Geobacillus	Geobacillus sp. Y4.1MC1	3911947	4031
649633048	Geobacillus sp. Y412MC52	Firmicutes	Bacilli	Bacillales	Bacillaceae	Geobacillus	Geobacillus sp. Y412MC52	3673940	3750
646311930	Geobacillus sp. Y412MC61	Firmicutes	Bacilli	Bacillales	Bacillaceae	Geobacillus	Geobacillus sp. Y412MC61	3667901	3718
2597490147	Geobacillus stearothermophilus NUB3621	Firmicutes	Bacilli	Bacillales	Bacillaceae	Geobacillus	Geobacillus stearothermophilus	3622285	3739
2597490167	Geobacillus stearothermophilus X1	Firmicutes	Bacilli	Bacillales	Bacillaceae	Geobacillus	Geobacillus stearothermophilus	3422674	3613
640069312	Geobacillus thermodenitrificans NG80-2	Firmicutes	Bacilli	Bacillales	Bacillaceae	Geobacillus	Geobacillus thermodenitrificans	3608012	3642
2623620299	Geobacillus thermodenitrificans NG80-2 (re-annotation)	Firmicutes	Bacilli	Bacillales	Bacillaceae	Geobacillus	Geobacillus thermodenitrificans	3608012	3691
650716039	Geobacillus thermoglucosidasius C56-YS93	Firmicutes	Bacilli	Bacillales	Bacillaceae	Geobacillus	Geobacillus thermoglucosidasius	3993793	4135
2511231121	Geobacillus thermoleovorans CCB_US3_UF5	Firmicutes	Bacilli	Bacillales	Bacillaceae	Geobacillus	Geobacillus thermoleovorans	3596620	3996
643692011	Brevibacillus brevis NBRC 100599	Firmicutes	Bacilli	Bacillales	Paenibacillaceae	Brevibacillus	Brevibacillus brevis	6296436	6119
2523231078	Paenibacillus larvae larvae 4-309, DSM 25430	Firmicutes	Bacilli	Bacillales	Paenibacillaceae	Paenibacillus	Paenibacillus larvae	4056006	4133
650716070	Paenibacillus mucilaginosus KNP414	Firmicutes	Bacilli	Bacillales	Paenibacillaceae	Paenibacillus	Paenibacillus mucilaginosus	8663821	7956
2512564039	Paenibacillus mucilaginosus 3016	Firmicutes	Bacilli	Bacillales	Paenibacillaceae	Paenibacillus	Paenibacillus mucilaginosus	8739048	7528
2513237182	Paenibacillus mucilaginosus K02	Firmicutes	Bacilli	Bacillales	Paenibacillaceae	Paenibacillus	Paenibacillus mucilaginosus	8770140	7476
648028048	Paenibacillus polymyxa E681	Firmicutes	Bacilli	Bacillales	Paenibacillaceae	Paenibacillus	Paenibacillus polymyxa	5394884	4932
649633079	Paenibacillus polymyxa SC2	Firmicutes	Bacilli	Bacillales	Paenibacillaceae	Paenibacillus	Paenibacillus polymyxa	6241931	6228
2517572207	Paenibacillus polymyxa M1	Firmicutes	Bacilli	Bacillales	Paenibacillaceae	Paenibacillus	Paenibacillus polymyxa	6231122	5516
2556921003	Paenibacillus polymyxa CR1 (Complete genome sequence)	Firmicutes	Bacilli	Bacillales	Paenibacillaceae	Paenibacillus	Paenibacillus polymyxa	6024666	5510
2588253742	Paenibacillus polymyxa SQR-21	Firmicutes	Bacilli	Bacillales	Paenibacillaceae	Paenibacillus	Paenibacillus polymyxa	5828436	5174
2576861424	Paenibacillus sabinae T27	Firmicutes	Bacilli	Bacillales	Paenibacillaceae	Paenibacillus	Paenibacillus sabinae	5270569	4896
646311929	Paenibacillus lautus Y412MC10	Firmicutes	Bacilli	Bacillales	Paenibacillaceae	Paenibacillus	Paenibacillus sp. Y412MC10	7121665	6444
2511231079	Paenibacillus terrae HPL-003	Firmicutes	Bacilli	Bacillales	Paenibacillaceae	Paenibacillus	Paenibacillus terrae	6083395	5642
2508501069	Thermobacillus composti KWC4, DSM 18247	Firmicutes	Bacilli	Bacillales	Paenibacillaceae	Thermobacillus	Thermobacillus composti	4355525	4120
646564509	Bacillus selenitireducens MLS10	Firmicutes	Bacilli	Bacillales	Sporolactobacillaceae	Bacillus	[Bacillus] selenitireducens	3592487	3420
643692023	Exiguobacterium sp. AT1b	Firmicutes	Bacilli	Bacillales	unclassified	Exiguobacterium	Exiguobacterium sp. AT1b	2999895	3138
649633101	Thermaerobacter marianensis 7p75a, DSM 12885	Firmicutes	Clostridia	Clostridiales	Clostridiales Family XVII. Incertae Sedis	Thermaerobacter	Thermaerobacter marianensis	2844696	2435
641522632	Heliobacterium modesticaldum Ice1	Firmicutes	Clostridia	Clostridiales	Heliobacteriaceae	Heliobacterium	Heliobacterium modesticaldum	3075407	3142
2505679104	Desulfotomaculum carboxydivorans CO-1-SRB, DSM 14880	Firmicutes	Clostridia	Clostridiales	Peptococcaceae	Desulfotomaculum	Desulfotomaculum carboxydivorans	2892255	2844
640069310	Desulfotomaculum reducens MI-1	Firmicutes	Clostridia	Clostridiales	Peptococcaceae	Desulfotomaculum	Desulfotomaculum reducens	3608104	3488
650716033	Desulfotomaculum ruminis DL, DSM 2154	Firmicutes	Clostridia	Clostridiales	Peptococcaceae	Desulfotomaculum	Desulfotomaculum ruminis	3969014	3986
646564578	Thermincola potens JR	Firmicutes	Clostridia	Clostridiales	Peptococcaceae	Thermincola	Thermincola potens	3157416	3081
648028002	Acetohalobium arabaticum Z-7288, DSM 5501	Firmicutes	Clostridia	Halanaerobiales	Halobacteroidaceae	Acetohalobium	Acetohalobium arabaticum	2469596	2438
642555142	Natranaerobius thermophilus JW/NM-WN-LF	Firmicutes	Clostridia	Natranaerobiales	Natranaerobiaceae	Natranaerobius	Natranaerobius thermophilus	3191453	3018
637000060	Carboxydothermus hydrogenoformans Z-2901, DSM 6008	Firmicutes	Clostridia	Thermoanaerobacterales	Thermoanaerobacteraceae	Carboxydothermus	Carboxydothermus hydrogenoformans	2401520	2738
2623620911	Carboxydothermus hydrogenoformans Z-2901, DSM 6008 (re-annotation)	Firmicutes	Clostridia	Thermoanaerobacterales	Thermoanaerobacteraceae	Carboxydothermus	Carboxydothermus hydrogenoformans	2401520	2555
643692024	Gemmatimonas aurantiaca T-27T	Gemmatimonadetes	Gemmatimonadetes	Gemmatimonadales	Gemmatimonadaceae	Gemmatimonas	Gemmatimonas aurantiaca	4636964	3987
2576861455	Gemmatimonadetes bacterium KBS708	Gemmatimonadetes	Gemmatimonadetes	unclassified	unclassified	unclassified	Gemmatimonadetes bacterium KBS708	7479215	6423
2513237218	Ignavibacterium album Mat9-16, JCM 16511	Ignavibacteriae	Ignavibacteria	Ignavibacteriales	Ignavibacteriaceae	Ignavibacterium	Ignavibacterium album	3658997	3243
2517093030	Melioribacter roseus P3M	Ignavibacteriae	Ignavibacteria	Ignavibacteriales	Melioribacteraceae	Melioribacter	Melioribacter roseus	3300414	2902

649633096	Stigmatella aurantiaca DW4/3-1	Proteobacteria	Deltaproteobacteria	Myxococcales	Cystobacteraceae	Stigmatella	Stigmatella aurantiaca	10260756	8407
2512564078	Corallococcus coralloides DSM 2259	Proteobacteria	Deltaproteobacteria	Myxococcales	Myxococcaceae	Corallococcus	Corallococcus coralloides	10080619	8101
650716065	Myxococcus fulvus HW-1	Proteobacteria	Deltaproteobacteria	Myxococcales	Myxococcaceae	Myxococcus	Myxococcus fulvus	9003593	7362
2521172697	Myxococcus stipitatus DSM 14675	Proteobacteria	Deltaproteobacteria	Myxococcales	Myxococcaceae	Myxococcus	Myxococcus stipitatus	10350586	8128
637000186	Myxococcus xanthus DK 1622	Proteobacteria	Deltaproteobacteria	Myxococcales	Myxococcaceae	Myxococcus	Myxococcus xanthus	9139763	7454
2623620856	Myxococcus xanthus DK 1622 (re-annotation)	Proteobacteria	Deltaproteobacteria	Myxococcales	Myxococcaceae	Myxococcus	Myxococcus xanthus	9139763	7408

Inferred N-Me-BSH synthesis: genomes encoding orthologs of CT0548/bshA + CT1419/bshB + CT1558/bshC + CT1040/nmbA

taxon_oid	Genome Name / Sample Name	Phylum	Class	Order	Family	Genus	Species	Genome Size	Gene Count
639633060	Candidatus Solibacter usitatus Ellin6076	Acidobacteria	Solibacteres	Solibacterales	Solibacteraceae	Candidatus Solibacter	Candidatus Solibacter usitatus	9965640	8003
2512047033	Candidatus *Chloracidobacterium thermophilum* B	Acidobacteria	unclassified	unclassified	unclassified	Candidatus Chloracidobacterium	Candidatus *Chloracidobacterium thermophilum*	3695372	3103
637000087	Cytophaga hutchinsonii ATCC 33406	Bacteroidetes	Cytophagia	Cytophagales	Cytophagaceae	Cytophaga	Cytophaga hutchinsonii	4433218	3843
2506485001	Emticicia oligotrophica GPTSA100-15, DSM 17448	Bacteroidetes	Cytophagia	Cytophagales	Cytophagaceae	Emticicia	Emticicia oligotrophica	5220361	4365
2509276059	Flexibacter litoralis Fx l1, DSM 6794	Bacteroidetes	Cytophagia	Cytophagales	Cytophagaceae	Flexibacter	Flexibacter litoralis	4919337	4090
2588253767	Hymenobacter sp. APR13	Bacteroidetes	Cytophagia	Cytophagales	Cytophagaceae	Hymenobacter	Hymenobacter sp. APR13	5010594	4303
2576861456	Hymenobacter swuensis DY53	Bacteroidetes	Cytophagia	Cytophagales	Cytophagaceae	Hymenobacter	Hymenobacter swuensis	5252070	4668
2503707007	Fluviicola taffensis RW262, DSM 16823	Bacteroidetes	Flavobacteriia	Flavobacteriales	Cryomorphaceae	Fluviicola	Fluviicola taffensis	4633577	4131
2508501098	Owenweeksia hongkongensis DSM 17368	Bacteroidetes	Flavobacteriia	Flavobacteriales	Cryomorphaceae	Owenweeksia	Owenweeksia hongkongensis	4000057	3563
644736369	Flavobacterium johnsoniae UW101, ATCC 17061	Bacteroidetes	Flavobacteriia	Flavobacteriales	Flavobacteriaceae	Flavobacterium	Flavobacterium johnsoniae	6096872	5099
2597490156	Flavobacterium psychrophilum CSF259-93	Bacteroidetes	Flavobacteriia	Flavobacteriales	Flavobacteriaceae	Flavobacterium	Flavobacterium psychrophilum	2900735	2718
640753027	Flavobacterium psychrophilum JIP02/86	Bacteroidetes	Flavobacteriia	Flavobacteriales	Flavobacteriaceae	Flavobacterium	Flavobacterium psychrophilum	2861988	2505
650716044	Lacinutrix sp. 5H-3-7-4	Bacteroidetes	Flavobacteriia	Flavobacteriales	Flavobacteriaceae	Lacinutrix	Lacinutrix sp. 5H-3-7-4	3296168	3026
638341218	Polaribacter sp. MED152	Bacteroidetes	Flavobacteriia	Flavobacteriales	Flavobacteriaceae	Polaribacter	Polaribacter sp. MED152	2967100	2723
650377986	Weeksella virosa 9751, DSM 16922	Bacteroidetes	Flavobacteriia	Flavobacteriales	Flavobacteriaceae	Weeksella	Weeksella virosa	2272954	2125
644736340	Chitinophaga pinensis UQM 2034, DSM 2588	Bacteroidetes	Sphingobacteriia	Sphingobacteriales	Chitinophagaceae	Chitinophaga	Chitinophaga pinensis	9127347	7396
2506520016	Niastella koreensis GR20-10, DSM 17620	Bacteroidetes	Sphingobacteriia	Sphingobacteriales	Chitinophagaceae	Niastella	Niastella koreensis	9033684	7444
2512564032	Saprospira grandis Lewin	Bacteroidetes	Sphingobacteriia	Sphingobacteriales	Saprospiraceae	Saprospira	Saprospira grandis	4400185	4324
649633082	Pedobacter saltans Steyn 113, DSM 12145	Bacteroidetes	Sphingobacteriia	Sphingobacteriales	Sphingobacteriaceae	Pedobacter	Pedobacter saltans	4635236	3921
2509276062	Solitalea canadensis USAM 9D, DSM 3403	Bacteroidetes	Sphingobacteriia	Sphingobacteriales	Sphingobacteriaceae	Solitalea	Solitalea canadensis	5202069	4490
2623620645	Salinibacter ruber M31, DSM 13855 (re-annotation)	Bacteroidetes	unclassified	Bacteroidetes Order II. Incertae sedis	Rhodothermaceae	Salinibacter	Salinibacter ruber	3587328	3086
2561511240	Salinibacter ruber M8	Bacteroidetes	unclassified	Bacteroidetes Order II. Incertae sedis	Rhodothermaceae	Salinibacter	Salinibacter ruber	3832918	3302
637000250	Salinibacter ruber M31, DSM 13855	Bacteroidetes	unclassified	Bacteroidetes Order II. Incertae sedis	Rhodothermaceae	Salinibacter	Salinibacter ruber	3587328	2899
646564588	Waddlia chondrophila WSU 86-1044	Chlamydiae	Chlamydiia	Chlamydiales	Waddliaceae	Waddlia	Waddlia chondrophila	2131905	1999
642555120	Chlorobaculum parvum DSM 263	Chlorobi	Chlorobia	Chlorobiales	Chlorobiaceae	Chlorobaculum	Chlorobaculum parvum	2289249	2133
637000072	Chlorobium chlorochromatii CaD3	Chlorobi	Chlorobia	Chlorobiales	Chlorobiaceae	Chlorobium	Chlorobium chlorochromatii	2572079	2100
642555121	Chlorobium limicola DSM 245	Chlorobi	Chlorobia	Chlorobiales	Chlorobiaceae	Chlorobium	Chlorobium limicola	2763181	2576
639633020	Chlorobium phaeobacteroides DSM 266	Chlorobi	Chlorobia	Chlorobiales	Chlorobiaceae	Chlorobium	Chlorobium phaeobacteroides	3133902	2805
642555122	Chlorobium phaeobacteroides BS1	Chlorobi	Chlorobia	Chlorobiales	Chlorobiaceae	Chlorobium	Chlorobium phaeobacteroides	2736403	2611
640427130	Chlorobium phaeovibrioides DSM 265	Chlorobi	Chlorobia	Chlorobiales	Chlorobiaceae	Chlorobium	Chlorobium phaeovibrioides	1966858	1831
637000073	*Chlorobium tepidum* TLS	Chlorobi	Chlorobia	Chlorobiales	Chlorobiaceae	Chlorobium	*Chlorobium tepidum*	2154946	2317
642555123	Chloroherpeton thalassium ATCC 35110	Chlorobi	Chlorobia	Chlorobiales	Chlorobiaceae	Chloroherpeton	Chloroherpeton thalassium	3293456	2778
637000205	Pelodictyon luteolum DSM 273	Chlorobi	Chlorobia	Chlorobiales	Chlorobiaceae	Pelodictyon	Pelodictyon luteolum	2364842	2146
642555146	Pelodictyon phaeoclathratiforme BU-1	Chlorobi	Chlorobia	Chlorobiales	Chlorobiaceae	Pelodictyon	Pelodictyon phaeoclathratiforme	3018238	2969
642555149	Prosthecochloris aestuarii SK413, DSM 271	Chlorobi	Chlorobia	Chlorobiales	Chlorobiaceae	Prosthecochloris	Prosthecochloris aestuarii	2579695	2451
643692011	Brevibacillus brevis NBRC 100599	Firmicutes	Bacilli	Bacillales	Paenibacillaceae	Brevibacillus	Brevibacillus brevis	6296436	6119
2523231078	Paenibacillus larvae larvae 4-309, DSM 25430	Firmicutes	Bacilli	Bacillales	Paenibacillaceae	Paenibacillus	Paenibacillus larvae	4056006	4133
2513237182	Paenibacillus mucilaginosus K02	Firmicutes	Bacilli	Bacillales	Paenibacillaceae	Paenibacillus	Paenibacillus mucilaginosus	8770140	7476
2512564039	Paenibacillus mucilaginosus 3016	Firmicutes	Bacilli	Bacillales	Paenibacillaceae	Paenibacillus	Paenibacillus mucilaginosus	8739048	7528
650716070	Paenibacillus mucilaginosus KNP414	Firmicutes	Bacilli	Bacillales	Paenibacillaceae	Paenibacillus	Paenibacillus mucilaginosus	8663821	7956
2556921003	Paenibacillus polymyxa CR1 (Complete genome sequence)	Firmicutes	Bacilli	Bacillales	Paenibacillaceae	Paenibacillus	Paenibacillus polymyxa	6024666	5510
649633079	Paenibacillus polymyxa SC2	Firmicutes	Bacilli	Bacillales	Paenibacillaceae	Paenibacillus	Paenibacillus polymyxa	6241931	6228
2588253742	Paenibacillus polymyxa SQR-21	Firmicutes	Bacilli	Bacillales	Paenibacillaceae	Paenibacillus	Paenibacillus polymyxa	5828436	5174

648028048	Paenibacillus polymyxa E681	Firmicutes	Bacilli	Bacillales	Paenibacillaceae	Paenibacillus	Paenibacillus polymyxa	5394884	4932
2517572207	Paenibacillus polymyxa M1	Firmicutes	Bacilli	Bacillales	Paenibacillaceae	Paenibacillus	Paenibacillus polymyxa	6231122	5516
2576861424	Paenibacillus sabinae T27	Firmicutes	Bacilli	Bacillales	Paenibacillaceae	Paenibacillus	Paenibacillus sabinae	5270569	4896
2511231079	Paenibacillus terrae HPL-003	Firmicutes	Bacilli	Bacillales	Paenibacillaceae	Paenibacillus	Paenibacillus terrae	6083395	5642
2513237218	Ignavibacterium album Mat9-16, JCM 16511	Ignavibacteriae	Ignavibacteria	Ignavibacteriales	Ignavibacteriaceae	Ignavibacterium	Ignavibacterium album	3658997	3243
2517093030	Melioribacter roseus P3M	Ignavibacteriae	Ignavibacteria	Ignavibacteriales	Melioribacteraceae	Melioribacter	Melioribacter roseus	3300414	2902
**Inferred GSH synthesis: genomes encoding orthologs of b2688/slr0990/gshA + b2947/slr1238/gshB**									
**taxon_oid**	**Genome Name / Sample Name**	**Phylum**	**Class**	**Order**	**Family**	**Genus**	**Species**	**Genome Size**	**Gene Count**
641228492	Frankia sp. EAN1pec	Actinobacteria	Actinobacteria	Actinomycetales	Frankiaceae	Frankia	Frankia sp. EAN1pec	8982042	7250
649633045	Frankia sp. EuI1c	Actinobacteria	Actinobacteria	Actinomycetales	Frankiaceae	Frankia	Frankia sp. EuI1c	8815781	7262
2576861435	Rhodococcus opacus PD630	Actinobacteria	Actinobacteria	Actinomycetales	Nocardiaceae	Rhodococcus	Rhodococcus opacus	9169032	9005
648028006	Amycolatopsis mediterranei U32	Actinobacteria	Actinobacteria	Actinomycetales	Pseudonocardiaceae	Amycolatopsis	Amycolatopsis mediterranei	10236715	9292
2511231181	Amycolatopsis mediterranei S699, ATCC 13685	Actinobacteria	Actinobacteria	Actinomycetales	Pseudonocardiaceae	Amycolatopsis	Amycolatopsis mediterranei	10236779	9639
2517093034	Amycolatopsis mediterranei S699	Actinobacteria	Actinobacteria	Actinomycetales	Pseudonocardiaceae	Amycolatopsis	Amycolatopsis mediterranei	10246920	9291
2554235376	Amycolatopsis mediterranei RB	Actinobacteria	Actinobacteria	Actinomycetales	Pseudonocardiaceae	Amycolatopsis	Amycolatopsis mediterranei	10246864	9293
2563366593	Streptomyces rapamycinicus NRRL 5491	Actinobacteria	Actinobacteria	Actinomycetales	Streptomycetaceae	Streptomyces	Streptomyces rapamycinicus	12700734	10144
2558309063	Gloeobacter kilaueensis JS1	Cyanobacteria	Gloeobacteria	Gloeobacterales	unclassified	Gloeobacter	Gloeobacter kilaueensis	4724791	4562
637000121	Gloeobacter violaceus PCC 7421	Cyanobacteria	Gloeobacteria	Gloeobacterales	unclassified	Gloeobacter	Gloeobacter violaceus	4659019	4490
2623620803	Gloeobacter violaceus PCC 7421 (re-annotation)	Cyanobacteria	Gloeobacteria	Gloeobacterales	unclassified	Gloeobacter	Gloeobacter violaceus	4659019	4574
641228474	Acaryochloris marina MBIC11017	Cyanobacteria	unclassified	Chroococcales	unclassified	Acaryochloris	Acaryochloris marina	8361599	8488
646311970	Candidatus Atelocyanobacterium thalassa ALOHA	Cyanobacteria	unclassified	Chroococcales	unclassified	Candidatus Atelocyanobacterium	Candidatus Atelocyanobacterium thalassa	1443806	1241
2510436000	Chamaesiphon minutus PCC 6605	Cyanobacteria	unclassified	Chroococcales	unclassified	Chamaesiphon	Chamaesiphon minutus	6761765	6427
2503707009	Cyanobacterium aponinum PCC 10605	Cyanobacteria	unclassified	Chroococcales	unclassified	Cyanobacterium	Cyanobacterium aponinum	4176973	3614
2503283023	Cyanobacterium stanieri PCC 7202	Cyanobacteria	unclassified	Chroococcales	unclassified	Cyanobacterium	Cyanobacterium stanieri	3163381	2941
2508501011	Cyanobium gracile PCC 6307	Cyanobacteria	unclassified	Chroococcales	unclassified	Cyanobium	Cyanobium gracile	3342364	3439
641522622	Cyanothece sp. BH68, ATCC 51142	Cyanobacteria	unclassified	Chroococcales	unclassified	Cyanothece	Cyanothece sp. ATCC 51142	5460377	5354
643348533	Cyanothece sp. PCC 7424	Cyanobacteria	unclassified	Chroococcales	unclassified	Cyanothece	Cyanothece sp. PCC 7424	6554169	5933
643348534	Cyanothece sp. PCC 7425	Cyanobacteria	unclassified	Chroococcales	unclassified	Cyanothece	Cyanothece sp. PCC 7425	5786110	5481
648028021	Cyanothece sp. PCC 7822	Cyanobacteria	unclassified	Chroococcales	unclassified	Cyanothece	Cyanothece sp. PCC 7822	7841948	7041
643348535	Cyanothece sp. PCC 8801	Cyanobacteria	unclassified	Chroococcales	unclassified	Cyanothece	Cyanothece sp. PCC 8801	4787694	4615
644736348	Cyanothece sp. PCC 8802	Cyanobacteria	unclassified	Chroococcales	unclassified	Cyanothece	Cyanothece sp. PCC 8802	4803347	4697
2509276056	Dactylococcopsis salina PCC 8305	Cyanobacteria	unclassified	Chroococcales	unclassified	Dactylococcopsis	Dactylococcopsis salina	3781008	3685
2503538028	Halothece sp. PCC 7418	Cyanobacteria	unclassified	Chroococcales	unclassified	Halothece	Halothece sp. PCC 7418	4179170	3920
641522640	Microcystis aeruginosa NIES-843	Cyanobacteria	unclassified	Chroococcales	unclassified	Microcystis	Microcystis aeruginosa	5842795	6360
637000310	Synechococcus sp. CC9605	Cyanobacteria	unclassified	Chroococcales	unclassified	Synechococcus	sp.	2510659	2761
637000311	Synechococcus sp. CC9902	Cyanobacteria	unclassified	Chroococcales	unclassified	Synechococcus	sp.	2234828	2410
637000314	Synechococcus sp. WH8102	Cyanobacteria	unclassified	Chroococcales	unclassified	Synechococcus	sp.	2434428	2588
637000307	Synechococcus elongatus PCC 6301	Cyanobacteria	unclassified	Chroococcales	unclassified	Synechococcus	Synechococcus elongatus	2696255	2585
637000308	Synechococcus elongatus PCC 7942	Cyanobacteria	unclassified	Chroococcales	unclassified	Synechococcus	Synechococcus elongatus	2742269	2719
2623620978	Synechococcus elongatus PCC 6301 (re-annotation)	Cyanobacteria	unclassified	Chroococcales	unclassified	Synechococcus	Synechococcus elongatus	2696255	2748
637000309	Synechococcus sp. CC9311	Cyanobacteria	unclassified	Chroococcales	unclassified	Synechococcus	Synechococcus sp. CC9311	2606748	2951
2623620876	Synechococcus sp. CC9311 (re-annotation)	Cyanobacteria	unclassified	Chroococcales	unclassified	Synechococcus	Synechococcus sp. CC9311	2606748	2940
637000312	Synechococcus sp. JA-2-3B'a(2-13)	Cyanobacteria	unclassified	Chroococcales	unclassified	Synechococcus	Synechococcus sp. JA-2-3B'a(2-13)	3046682	2947
2623620979	Synechococcus sp. JA-2-3B'a(2-13) (re-annotation)	Cyanobacteria	unclassified	Chroococcales	unclassified	Synechococcus	Synechococcus sp. JA-2-3B'a(2-13)	3046682	2897
637000313	Synechococcus sp. JA-3-3Ab	Cyanobacteria	unclassified	Chroococcales	unclassified	Synechococcus	Synechococcus sp. JA-3-3Ab	2932766	2900
2623620831	Synechococcus sp. JA-3-3Ab (re-annotation)	Cyanobacteria	unclassified	Chroococcales	unclassified	Synechococcus	Synechococcus sp. JA-3-3Ab	2932766	2814
2507262013	Synechococcus sp. KORDI-100 (genome sequencing)	Cyanobacteria	unclassified	Chroococcales	unclassified	Synechococcus	Synechococcus sp. KORDI-100	2789000	3058
2507262011	Synechococcus sp. KORDI-49 (genome sequencing)	Cyanobacteria	unclassified	Chroococcales	unclassified	Synechococcus	Synechococcus sp. KORDI-49	2585813	2717
2507262012	Synechococcus sp. KORDI-52 (genome sequencing)	Cyanobacteria	unclassified	Chroococcales	unclassified	Synechococcus	Synechococcus sp. KORDI-52	2572069	2823
2509276030	Synechococcus sp. PCC 6312	Cyanobacteria	unclassified	Chroococcales	unclassified	Synechococcus	Synechococcus sp. PCC 6312	3720499	3794
641522654	Synechococcus sp. PCC 7002	Cyanobacteria	unclassified	Chroococcales	unclassified	Synechococcus	Synechococcus sp. PCC 7002	3409935	3237
2508501041	Synechococcus sp. PCC 7502	Cyanobacteria	unclassified	Chroococcales	unclassified	Synechococcus	Synechococcus sp. PCC 7502	3583735	3669
640427148	Synechococcus sp. RCC307	Cyanobacteria	unclassified	Chroococcales	unclassified	Synechococcus	Synechococcus sp. RCC307	2224914	2588

2623620283	Synechococcus sp. RCC307 (re-annotation)	Cyanobacteria	unclassified	Chroococcales	unclassified	Synechococcus	Synechococcus sp. RCC307	2224914	2591
640427149	Synechococcus sp. WH7803	Cyanobacteria	unclassified	Chroococcales	unclassified	Synechococcus	Synechococcus sp. WH 7803	2366980	2591
2623620330	Synechococcus sp. WH7803 (re-annotation)	Cyanobacteria	unclassified	Chroococcales	unclassified	Synechococcus	Synechococcus sp. WH 7803	2366980	2610
2563366603	Synechococcus sp. WH 8109	Cyanobacteria	unclassified	Chroococcales	unclassified	Synechococcus	Synechococcus sp. WH 8109	2111515	2713
637000315	Synechocystis sp. PCC 6803 initial version	Cyanobacteria	unclassified	Chroococcales	unclassified	Synechocystis	Synechocystis sp. PCC 6803	3947019	3628
651053076	Synechocystis sp. GT-S, PCC 6803	Cyanobacteria	unclassified	Chroococcales	unclassified	Synechocystis	Synechocystis sp. PCC 6803	3571103	3220
2513237195	Synechocystis sp. PCC 6803, PCC-N	Cyanobacteria	unclassified	Chroococcales	unclassified	Synechocystis	Synechocystis sp. PCC 6803	3570114	3217
2513237196	Synechocystis sp. PCC 6803, GT-I	Cyanobacteria	unclassified	Chroococcales	unclassified	Synechocystis	Synechocystis sp. PCC 6803	3570103	3217
2514885032	Synechocystis sp. PCC 6803 (updateJune2012)	Cyanobacteria	unclassified	Chroococcales	unclassified	Synechocystis	Synechocystis sp. PCC 6803	3947019	3771
2524023216	Synechocystis sp. PCC 6803, PCC-P	Cyanobacteria	unclassified	Chroococcales	unclassified	Synechocystis	Synechocystis sp. PCC 6803	3570114	3218
2561511183	Synechocystis sp. PCC 6803	Cyanobacteria	unclassified	Chroococcales	unclassified	Synechocystis	Synechocystis sp. PCC 6803	3949306	3610
637000320	Thermosynechococcus elongatus BP-1	Cyanobacteria	unclassified	Chroococcales	unclassified	Thermosynechococcus	Thermosynechococcus elongatus	2593857	2555
2623620740	Thermosynechococcus elongatus BP-1 (re-annotation)	Cyanobacteria	unclassified	Chroococcales	unclassified	Thermosynechococcus	Thermosynechococcus elongatus	2593857	2624
2597489959	Thermosynechococcus sp. NK55a	Cyanobacteria	unclassified	Chroococcales	unclassified	Thermosynechococcus	Thermosynechococcus sp. NK55a	2520064	2497
2503754017	Gloeocapsa sp. PCC 7428	Cyanobacteria	unclassified	Chroococcales	unclassified	unclassified	unclassified	5882710	5304
2503982047	Anabaena cylindrica PCC 7122	Cyanobacteria	unclassified	Nostocales	Nostocaceae	Anabaena	Anabaena cylindrica	7063285	6258
2561511140	Anabaena sp. 90	Cyanobacteria	unclassified	Nostocales	Nostocaceae	Anabaena	Anabaena sp. 90	5305675	4797
646564504	Anabaena variabilis ATCC 29413	Cyanobacteria	unclassified	Nostocales	Nostocaceae	Anabaena	Anabaena variabilis	7105752	5772
2509601025	Cylindrospermum stagnale PCC 7417	Cyanobacteria	unclassified	Nostocales	Nostocaceae	Cylindrospermum	Cylindrospermum stagnale	7610589	6739
2562617131	Nodularia spumigena CCY9414	Cyanobacteria	unclassified	Nostocales	Nostocaceae	Nodularia	Nodularia spumigena	5465271	5363
642555144	Nostoc punctiforme PCC 73102	Cyanobacteria	unclassified	Nostocales	Nostocaceae	Nostoc	Nostoc punctiforme	9059191	6791
2503707008	Nostoc sp. PCC 7107	Cyanobacteria	unclassified	Nostocales	Nostocaceae	Nostoc	Nostoc sp. PCC 7107	6329823	5538
637000199	Nostoc sp. PCC 7120	Cyanobacteria	unclassified	Nostocales	Nostocaceae	Nostoc	Nostoc sp. PCC 7120	7211789	6222
2623620956	Nostoc sp. PCC 7120 (re-annotation)	Cyanobacteria	unclassified	Nostocales	Nostocaceae	Nostoc	Nostoc sp. PCC 7120	7211789	6252
2509601032	Nostoc sp. PCC 7524	Cyanobacteria	unclassified	Nostocales	Nostocaceae	Nostoc	Nostoc sp. PCC 7524	6718869	5688
648028001	Nostoc azollae 0708	Cyanobacteria	unclassified	Nostocales	Nostocaceae	Trichormus	Trichormus azollae	5486145	5379
2503982036	Calothrix sp. PCC 6303	Cyanobacteria	unclassified	Nostocales	Rivulariaceae	Calothrix	Calothrix sp.	6960392	5840
2505679032	Calothrix sp. PCC 7507	Cyanobacteria	unclassified	Nostocales	Rivulariaceae	Calothrix	Calothrix sp. PCC 7507	7023215	6250
2510065008	Rivularia sp. PCC 7116	Cyanobacteria	unclassified	Nostocales	Rivulariaceae	Rivularia	Rivularia sp. PCC 7116	8728773	6946
650377906	Arthrospira platensis NIES-39	Cyanobacteria	unclassified	Oscillatoriales	unclassified	Arthrospira	Arthrospira platensis	6788435	6676
2504643013	Crinalium epipsammum PCC 9333	Cyanobacteria	unclassified	Oscillatoriales	unclassified	Crinalium	Crinalium epipsammum	5620407	5059
2503538020	Geitlerinema sp. PCC 7407	Cyanobacteria	unclassified	Oscillatoriales	unclassified	Geitlerinema	Geitlerinema sp. PCC 7407	4681111	3913
2503754048	Leptolyngbya sp. PCC 7376	Cyanobacteria	unclassified	Oscillatoriales	unclassified	Leptolyngbya	Leptolyngbya sp. PCC 7376	5125950	4654
2509276031	Microcoleus sp. PCC 7113	Cyanobacteria	unclassified	Oscillatoriales	unclassified	Microcoleus	Microcoleus sp. PCC 7113	7966510	6822
2509276028	Oscillatoria acuminata PCC 6304	Cyanobacteria	unclassified	Oscillatoriales	unclassified	Oscillatoria	Oscillatoria acuminata	7804270	6101
2503982035	Oscillatoria nigro-viridis PCC 7112	Cyanobacteria	unclassified	Oscillatoriales	unclassified	Oscillatoria	Oscillatoria nigro-viridis	8272254	7006
2504643012	Pseudanabaena sp. PCC 7367	Cyanobacteria	unclassified	Oscillatoriales	unclassified	Pseudanabaena	Pseudanabaena sp. PCC 7367	4885680	4015
637000329	Trichodesmium erythraeum IMS101	Cyanobacteria	unclassified	Oscillatoriales	unclassified	Trichodesmium	Trichodesmium erythraeum	7750108	5156
2503538021	Chroococcidiopsis thermalis PCC 7203	Cyanobacteria	unclassified	Pleurocapsales	unclassified	Chroococcidiopsis	Chroococcidiopsis thermalis	6689401	6033
2509276061	Pleurocapsa sp. PCC 7327	Cyanobacteria	unclassified	Pleurocapsales	unclassified	Pleurocapsa	Pleurocapsa minor	4986817	4667
2503754019	Stanieria cyanosphaera PCC 7437	Cyanobacteria	unclassified	Pleurocapsales	unclassified	Stanieria	Stanieria cyanosphaera	5544990	5041
637000210	Prochlorococcus sp. MIT9312	Cyanobacteria	unclassified	Prochlorales	Prochlorococcaceae	Prochlorococcus	Prochlorococcus marinus	1709204	1860
637000211	Prochlorococcus marinus MIT9313	Cyanobacteria	unclassified	Prochlorales	Prochlorococcaceae	Prochlorococcus	Prochlorococcus marinus	2410873	2334
637000212	Prochlorococcus sp. NATL2A	Cyanobacteria	unclassified	Prochlorales	Prochlorococcaceae	Prochlorococcus	Prochlorococcus marinus	1842899	1985
637000213	Prochlorococcus marinus marinus CCMP1375	Cyanobacteria	unclassified	Prochlorales	Prochlorococcaceae	Prochlorococcus	Prochlorococcus marinus	1751080	1933
637000214	Prochlorococcus marinus pastoris CCMP 1986	Cyanobacteria	unclassified	Prochlorales	Prochlorococcaceae	Prochlorococcus	Prochlorococcus marinus	1657990	1766
640069321	Prochlorococcus sp. AS9601	Cyanobacteria	unclassified	Prochlorales	Prochlorococcaceae	Prochlorococcus	Prochlorococcus marinus	1669886	1988
640069322	Prochlorococcus sp. MIT9301	Cyanobacteria	unclassified	Prochlorales	Prochlorococcaceae	Prochlorococcus	Prochlorococcus marinus	1641879	1967
640069323	Prochlorococcus sp. MIT9303	Cyanobacteria	unclassified	Prochlorales	Prochlorococcaceae	Prochlorococcus	Prochlorococcus marinus	2682675	3133
640069324	Prochlorococcus sp. MIT9515	Cyanobacteria	unclassified	Prochlorales	Prochlorococcaceae	Prochlorococcus	Prochlorococcus marinus	1704176	1968
640069325	Prochlorococcus sp. NATL1A	Cyanobacteria	unclassified	Prochlorales	Prochlorococcaceae	Prochlorococcus	Prochlorococcus marinus	1864731	2251
640753041	Prochlorococcus sp. MIT9215	Cyanobacteria	unclassified	Prochlorales	Prochlorococcaceae	Prochlorococcus	Prochlorococcus marinus	1738790	2059
641228501	Prochlorococcus sp. MIT9211	Cyanobacteria	unclassified	Prochlorales	Prochlorococcaceae	Prochlorococcus	Prochlorococcus marinus	1688963	1901
2606217555	Prochlorococcus sp. MIT9211 (re-annotation)	Cyanobacteria	unclassified	Prochlorales	Prochlorococcaceae	Prochlorococcus	Prochlorococcus marinus	1688963	1891
2606217556	Prochlorococcus sp. AS9601 (re-annotation)	Cyanobacteria	unclassified	Prochlorales	Prochlorococcaceae	Prochlorococcus	Prochlorococcus marinus	1669886	1914

2606217557	Prochlorococcus sp. NATL1A (re-annotation)	Cyanobacteria	unclassified	Prochlorales	Prochlorococcaceae	Prochlorococcus	Prochlorococcus marinus	1864731	2171
2606217558	Prochlorococcus sp. MIT9301 (re-annotation)	Cyanobacteria	unclassified	Prochlorales	Prochlorococcaceae	Prochlorococcus	Prochlorococcus marinus	1641879	1906
2606217559	Prochlorococcus sp. MIT9215 (re-annotation)	Cyanobacteria	unclassified	Prochlorales	Prochlorococcaceae	Prochlorococcus	Prochlorococcus marinus	1738790	1999
2606217667	Prochlorococcus sp. MIT9303 (re-annotation)	Cyanobacteria	unclassified	Prochlorales	Prochlorococcaceae	Prochlorococcus	Prochlorococcus marinus	2682675	2758
2606217669	Prochlorococcus sp. MIT9515 (re-annotation)	Cyanobacteria	unclassified	Prochlorales	Prochlorococcaceae	Prochlorococcus	Prochlorococcus marinus	1704176	1920
2623620316	Prochlorococcus sp. MIT9301 (re-annotation)	Cyanobacteria	unclassified	Prochlorales	Prochlorococcaceae	Prochlorococcus	Prochlorococcus marinus	1641879	1927
2623620345	Prochlorococcus sp. MIT9515 (re-annotation)	Cyanobacteria	unclassified	Prochlorales	Prochlorococcaceae	Prochlorococcus	Prochlorococcus marinus	1704176	1949
2623620346	Prochlorococcus sp. AS9601 (re-annotation)	Cyanobacteria	unclassified	Prochlorales	Prochlorococcaceae	Prochlorococcus	Prochlorococcus marinus	1669886	1940
2623620347	Prochlorococcus sp. MIT9303 (re-annotation)	Cyanobacteria	unclassified	Prochlorales	Prochlorococcaceae	Prochlorococcus	Prochlorococcus marinus	2682675	2796
2623620348	Prochlorococcus sp. NATL1A (re-annotation)	Cyanobacteria	unclassified	Prochlorales	Prochlorococcaceae	Prochlorococcus	Prochlorococcus marinus	1864731	2208
2623620732	Prochlorococcus sp. NATL1A (re-annotation)	Cyanobacteria	unclassified	Prochlorales	Prochlorococcaceae	Prochlorococcus	Prochlorococcus marinus	1864731	2208
2623620733	Prochlorococcus marinus marinus CCMP1375 (re-annotation)	Cyanobacteria	unclassified	Prochlorales	Prochlorococcaceae	Prochlorococcus	Prochlorococcus marinus	1751080	1959
2623620959	Prochlorococcus sp. AS9601 (re-annotation)	Cyanobacteria	unclassified	Prochlorales	Prochlorococcaceae	Prochlorococcus	Prochlorococcus marinus	1669886	1940
2623620960	Prochlorococcus sp. MIT9211 (re-annotation)	Cyanobacteria	unclassified	Prochlorales	Prochlorococcaceae	Prochlorococcus	Prochlorococcus marinus	1688963	1902
2623620961	Prochlorococcus sp. MIT9301 (re-annotation)	Cyanobacteria	unclassified	Prochlorales	Prochlorococcaceae	Prochlorococcus	Prochlorococcus marinus	1641879	1927
2623620962	Prochlorococcus sp. MIT9303 (re-annotation)	Cyanobacteria	unclassified	Prochlorales	Prochlorococcaceae	Prochlorococcus	Prochlorococcus marinus	2682675	2796
2623620963	Prochlorococcus sp. MIT9515 (re-annotation)	Cyanobacteria	unclassified	Prochlorales	Prochlorococcaceae	Prochlorococcus	Prochlorococcus marinus	1704176	1949
2606217688	Prochlorococcus sp. MIT0604	Cyanobacteria	unclassified	Prochlorales	Prochlorococcaceae	Prochlorococcus	Prochlorococcus sp. MIT 0604	1780061	2089
2606217560	Prochlorococcus sp. MIT0801	Cyanobacteria	unclassified	Prochlorales	Prochlorococcaceae	Prochlorococcus	Prochlorococcus sp. MIT 0801	1929203	2218
649633007	Asticcacaulis excentricus CB 48	Proteobacteria	Alphaproteobacteria	Caulobacterales	Caulobacteraceae	Asticcacaulis	Asticcacaulis excentricus	4308776	3921
648028010	Brevundimonas subvibrioides ATCC 15264	Proteobacteria	Alphaproteobacteria	Caulobacterales	Caulobacteraceae	Brevundimonas	Brevundimonas subvibrioides	3445263	3393
646564519	Caulobacter segnis ATCC 21756	Proteobacteria	Alphaproteobacteria	Caulobacterales	Caulobacteraceae	Caulobacter	Caulobacter segnis	4655622	4325
641522612	Caulobacter sp. K31	Proteobacteria	Alphaproteobacteria	Caulobacterales	Caulobacteraceae	Caulobacter	Caulobacter sp. K31	5889399	5499
637000061	Caulobacter crescentus CB15	Proteobacteria	Alphaproteobacteria	Caulobacterales	Caulobacteraceae	Caulobacter	Caulobacter vibrioides	4016947	3812
643348526	Caulobacter crescentus NA1000	Proteobacteria	Alphaproteobacteria	Caulobacterales	Caulobacteraceae	Caulobacter	Caulobacter vibrioides	4042929	3933
2623620841	Caulobacter crescentus CB15 (re-annotation)	Proteobacteria	Alphaproteobacteria	Caulobacterales	Caulobacteraceae	Caulobacter	Caulobacter vibrioides	4016947	3786
642555147	Phenylobacterium zucineum HLK1	Proteobacteria	Alphaproteobacteria	Caulobacterales	Caulobacteraceae	Phenylobacterium	Phenylobacterium zucineum	4379231	3899
648028050	Parvularcula bermudensis HTCC2503	Proteobacteria	Alphaproteobacteria	Parvularculales	Parvularculaceae	Parvularcula	Parvularcula bermudensis	2902643	2733
2526164707	Bartonella australis Aust/NH1	Proteobacteria	Alphaproteobacteria	Rhizobiales	Bartonellaceae	Bartonella	Bartonella australis	1596490	1313
639633009	Bartonella bacilliformis KC583	Proteobacteria	Alphaproteobacteria	Rhizobiales	Bartonellaceae	Bartonella	Bartonella bacilliformis	1445021	1377
2623620344	Bartonella bacilliformis KC583 (re-annotation)	Proteobacteria	Alphaproteobacteria	Rhizobiales	Bartonellaceae	Bartonella	Bartonella bacilliformis	1445021	1289
649633014	Bartonella clarridgeiae 73	Proteobacteria	Alphaproteobacteria	Rhizobiales	Bartonellaceae	Bartonella	Bartonella clarridgeiae	1522743	1393
644736328	Bartonella grahamii as4aup	Proteobacteria	Alphaproteobacteria	Rhizobiales	Bartonellaceae	Bartonella	Bartonella grahamii	2369520	1816
637000027	Bartonella henselae Houston-1	Proteobacteria	Alphaproteobacteria	Rhizobiales	Bartonellaceae	Bartonella	Bartonella henselae	1931047	1671
2585427667	Bartonella henselae BM1374165	Proteobacteria	Alphaproteobacteria	Rhizobiales	Bartonellaceae	Bartonella	Bartonella henselae	1975503	1726
2585427673	Bartonella henselae BM1374163	Proteobacteria	Alphaproteobacteria	Rhizobiales	Bartonellaceae	Bartonella	Bartonella henselae	1905383	1658
2623620900	Bartonella henselae Houston-1 (re-annotation)	Proteobacteria	Alphaproteobacteria	Rhizobiales	Bartonellaceae	Bartonella	Bartonella henselae	1931047	1689
641228478	Bartonella tribocorum CIP 105476	Proteobacteria	Alphaproteobacteria	Rhizobiales	Bartonellaceae	Bartonella	Bartonella tribocorum	2642404	2169
2588253764	Bartonella tribocorum BM1374166	Proteobacteria	Alphaproteobacteria	Rhizobiales	Bartonellaceae	Bartonella	Bartonella tribocorum	2631180	2351
2526164714	Bartonella vinsonii berkhoffii Winnie	Proteobacteria	Alphaproteobacteria	Rhizobiales	Bartonellaceae	Bartonella	Bartonella vinsonii	1802699	1484
641522606	Beijerinckia indica indica ATCC 9039	Proteobacteria	Alphaproteobacteria	Rhizobiales	Beijerinckiaceae	Beijerinckia	Beijerinckia indica	4418616	3850
637000038	Bradyrhizobium japonicum USDA 110	Proteobacteria	Alphaproteobacteria	Rhizobiales	Bradyrhizobiaceae	Bradyrhizobium	Bradyrhizobium diazoefficiens	9105828	8402
2623620631	Bradyrhizobium japonicum USDA 110 (re-annotation)	Proteobacteria	Alphaproteobacteria	Rhizobiales	Bradyrhizobiaceae	Bradyrhizobium	Bradyrhizobium diazoefficiens	9105828	8584
2511231207	Bradyrhizobium japonicum USDA 6	Proteobacteria	Alphaproteobacteria	Rhizobiales	Bradyrhizobiaceae	Bradyrhizobium	Bradyrhizobium japonicum	9207384	8886
2576861420	Bradyrhizobium japonicum SEMIA 5079	Proteobacteria	Alphaproteobacteria	Rhizobiales	Bradyrhizobiaceae	Bradyrhizobium	Bradyrhizobium japonicum	9583027	8705
2597489926	Agromonas oligotrophica S58	Proteobacteria	Alphaproteobacteria	Rhizobiales	Bradyrhizobiaceae	Bradyrhizobium	Bradyrhizobium oligotrophicum	8264165	7210
640427103	Bradyrhizobium sp. BTAi1	Proteobacteria	Alphaproteobacteria	Rhizobiales	Bradyrhizobiaceae	Bradyrhizobium	Bradyrhizobium sp. BTAi1	8493513	7819
640427104	Bradyrhizobium sp. ORS278	Proteobacteria	Alphaproteobacteria	Rhizobiales	Bradyrhizobiaceae	Bradyrhizobium	Bradyrhizobium sp. ORS 278	7456587	6825
2623620269	Bradyrhizobium sp. ORS278 (re-annotation)	Proteobacteria	Alphaproteobacteria	Rhizobiales	Bradyrhizobiaceae	Bradyrhizobium	Bradyrhizobium sp. ORS 278	7456587	6744
2513237178	Bradyrhizobium sp. S23321	Proteobacteria	Alphaproteobacteria	Rhizobiales	Bradyrhizobiaceae	Bradyrhizobium	Bradyrhizobium sp. S23321	7231841	6943
637000192	Nitrobacter hamburgensis X14	Proteobacteria	Alphaproteobacteria	Rhizobiales	Bradyrhizobiaceae	Nitrobacter	Nitrobacter hamburgensis	5011522	4747
637000193	Nitrobacter winogradskyi Nb-255	Proteobacteria	Alphaproteobacteria	Rhizobiales	Bradyrhizobiaceae	Nitrobacter	Nitrobacter winogradskyi	3402093	3215
637000237	Rhodopseudomonas palustris BisB18	Proteobacteria	Alphaproteobacteria	Rhizobiales	Bradyrhizobiaceae	Rhodopseudomonas	Rhodopseudomonas palustris	5513844	5028
637000238	Rhodopseudomonas palustris BisB5	Proteobacteria	Alphaproteobacteria	Rhizobiales	Bradyrhizobiaceae	Rhodopseudomonas	Rhodopseudomonas palustris	4892717	4501
637000239	Rhodopseudomonas palustris CGA009	Proteobacteria	Alphaproteobacteria	Rhizobiales	Bradyrhizobiaceae	Rhodopseudomonas	Rhodopseudomonas palustris	5467640	4918

637000240	Rhodopseudomonas palustris HaA2	Proteobacteria	Alphaproteobacteria	Rhizobiales	Bradyrhizobiaceae	Rhodopseudomonas	Rhodopseudomonas palustris	5331656	4788
639279312	Rhodopseudomonas palustris BisA53	Proteobacteria	Alphaproteobacteria	Rhizobiales	Bradyrhizobiaceae	Rhodopseudomonas	Rhodopseudomonas palustris	5505494	4996
642555153	Rhodopseudomonas palustris TIE-1	Proteobacteria	Alphaproteobacteria	Rhizobiales	Bradyrhizobiaceae	Rhodopseudomonas	Rhodopseudomonas palustris	5744041	5377
649633091	Rhodopseudomonas palustris DX-1	Proteobacteria	Alphaproteobacteria	Rhizobiales	Bradyrhizobiaceae	Rhodopseudomonas	Rhodopseudomonas palustris	5404117	5081
637000039	Brucella abortus bv. 1 9-941	Proteobacteria	Alphaproteobacteria	Rhizobiales	Brucellaceae	Brucella	Brucella abortus	3286445	3360
637000041	Brucella melitensis bv. 1 Abortus 2308	Proteobacteria	Alphaproteobacteria	Rhizobiales	Brucellaceae	Brucella	Brucella abortus	3278307	3117
642555110	Brucella abortus S19	Proteobacteria	Alphaproteobacteria	Rhizobiales	Brucellaceae	Brucella	Brucella abortus	3283936	3066
2511231108	Brucella abortus A13334	Proteobacteria	Alphaproteobacteria	Rhizobiales	Brucellaceae	Brucella	Brucella abortus	3286032	3401
2597490026	Brucella abortus bv. 6 870	Proteobacteria	Alphaproteobacteria	Rhizobiales	Brucellaceae	Brucella	Brucella abortus	3281154	3172
2597490090	Brucella abortus BFY	Proteobacteria	Alphaproteobacteria	Rhizobiales	Brucellaceae	Brucella	Brucella abortus	3288158	3170
2597490091	Brucella abortus BER	Proteobacteria	Alphaproteobacteria	Rhizobiales	Brucellaceae	Brucella	Brucella abortus	3288518	3174
2597490092	Brucella abortus 63 75	Proteobacteria	Alphaproteobacteria	Rhizobiales	Brucellaceae	Brucella	Brucella abortus	3280310	3158
2597490094	Brucella abortus NCTC 10505	Proteobacteria	Alphaproteobacteria	Rhizobiales	Brucellaceae	Brucella	Brucella abortus	3285279	3174
2597490097	Brucella abortus bv. 9 C68	Proteobacteria	Alphaproteobacteria	Rhizobiales	Brucellaceae	Brucella	Brucella abortus	3279946	3159
2597490098	Brucella abortus bv. 2 86/8/59	Proteobacteria	Alphaproteobacteria	Rhizobiales	Brucellaceae	Brucella	Brucella abortus	3286128	3183
2597490131	Brucella abortus BDW	Proteobacteria	Alphaproteobacteria	Rhizobiales	Brucellaceae	Brucella	Brucella abortus	3289500	3198
2623620632	Brucella abortus bv. 1 9-941 (re-annotation)	Proteobacteria	Alphaproteobacteria	Rhizobiales	Brucellaceae	Brucella	Brucella abortus	3286445	3205
2623620634	Brucella melitensis bv. 1 Abortus 2308 (re-annotation)	Proteobacteria	Alphaproteobacteria	Rhizobiales	Brucellaceae	Brucella	Brucella abortus	3278307	3196
641228480	Brucella canis ATCC 23365	Proteobacteria	Alphaproteobacteria	Rhizobiales	Brucellaceae	Brucella	Brucella canis	3312769	3408
2511231087	Brucella canis HSK A52141	Proteobacteria	Alphaproteobacteria	Rhizobiales	Brucellaceae	Brucella	Brucella canis	3277512	3342
2576861463	Brucella canis SVA13	Proteobacteria	Alphaproteobacteria	Rhizobiales	Brucellaceae	Brucella	Brucella canis	3310315	3093
2597490132	Brucella canis RM6/66	Proteobacteria	Alphaproteobacteria	Rhizobiales	Brucellaceae	Brucella	Brucella canis	3312751	3170
2558309086	Brucella ceti TE10759-12	Proteobacteria	Alphaproteobacteria	Rhizobiales	Brucellaceae	Brucella	Brucella ceti	3278034	2679
2563366595	Brucella ceti TE28753-12	Proteobacteria	Alphaproteobacteria	Rhizobiales	Brucellaceae	Brucella	Brucella ceti	3277545	2477
637000040	Brucella melitensis bv. 1 16M	Proteobacteria	Alphaproteobacteria	Rhizobiales	Brucellaceae	Brucella	Brucella melitensis	3294931	3279
643692012	Brucella melitensis bv. 2 63/9	Proteobacteria	Alphaproteobacteria	Rhizobiales	Brucellaceae	Brucella	Brucella melitensis	3311219	3203
651053010	Brucella melitensis M28	Proteobacteria	Alphaproteobacteria	Rhizobiales	Brucellaceae	Brucella	Brucella melitensis	3311748	3427
651053011	Brucella melitensis M5-90	Proteobacteria	Alphaproteobacteria	Rhizobiales	Brucellaceae	Brucella	Brucella melitensis	3312229	3424
2511231208	Brucella melitensis bv. 3 NI	Proteobacteria	Alphaproteobacteria	Rhizobiales	Brucellaceae	Brucella	Brucella melitensis	3294475	3290
2588253747	Brucella melitensis bv. 2 63/9	Proteobacteria	Alphaproteobacteria	Rhizobiales	Brucellaceae	Brucella	Brucella melitensis	3312958	3201
2597490113	Brucella melitensis bv. 1 16M	Proteobacteria	Alphaproteobacteria	Rhizobiales	Brucellaceae	Brucella	Brucella melitensis	3294775	3191
2597490139	Brucella melitensis bv. 3 Ether	Proteobacteria	Alphaproteobacteria	Rhizobiales	Brucellaceae	Brucella	Brucella melitensis	3310727	3210
2623620633	Brucella melitensis bv. 1 16M (re-annotation)	Proteobacteria	Alphaproteobacteria	Rhizobiales	Brucellaceae	Brucella	Brucella melitensis	3294931	3225
644736332	Brucella microti CCM 4915	Proteobacteria	Alphaproteobacteria	Rhizobiales	Brucellaceae	Brucella	Brucella microti	3337369	3346
640427105	Brucella ovis ATCC 25840	Proteobacteria	Alphaproteobacteria	Rhizobiales	Brucellaceae	Brucella	Brucella ovis	3275590	3193
2623620313	Brucella ovis ATCC 25840 (re-annotation)	Proteobacteria	Alphaproteobacteria	Rhizobiales	Brucellaceae	Brucella	Brucella ovis	3275590	3293
2511231179	Brucella pinnipedialis B2/94	Proteobacteria	Alphaproteobacteria	Rhizobiales	Brucellaceae	Brucella	Brucella pinnipedialis	3399268	3505
2597490130	Brucella pinnipedialis 6/566	Proteobacteria	Alphaproteobacteria	Rhizobiales	Brucellaceae	Brucella	Brucella pinnipedialis	3331029	3195
637000042	Brucella suis bv. 1 1330	Proteobacteria	Alphaproteobacteria	Rhizobiales	Brucellaceae	Brucella	Brucella suis	3315175	3408
641228481	Brucella suis ATCC 23445	Proteobacteria	Alphaproteobacteria	Rhizobiales	Brucellaceae	Brucella	Brucella suis	3324607	3419
2511231120	Brucella suis VBI22	Proteobacteria	Alphaproteobacteria	Rhizobiales	Brucellaceae	Brucella	Brucella suis	3316088	3393
2519103110	Brucella suis bv. 1 1330	Proteobacteria	Alphaproteobacteria	Rhizobiales	Brucellaceae	Brucella	Brucella suis	3315163	3388
2576861461	Brucella suis bv. 1 S2	Proteobacteria	Alphaproteobacteria	Rhizobiales	Brucellaceae	Brucella	Brucella suis	3315275	3294
2585427638	Brucella suis bv. 2 Bs396CITA	Proteobacteria	Alphaproteobacteria	Rhizobiales	Brucellaceae	Brucella	Brucella suis	3328458	3437
2585427647	Brucella suis bv. 2 Bs143CITA	Proteobacteria	Alphaproteobacteria	Rhizobiales	Brucellaceae	Brucella	Brucella suis	3324539	3426
2585427651	Brucella suis bv. 2 Bs364CITA	Proteobacteria	Alphaproteobacteria	Rhizobiales	Brucellaceae	Brucella	Brucella suis	3328972	3435
2585427652	Brucella suis bv. 2 PT09143	Proteobacteria	Alphaproteobacteria	Rhizobiales	Brucellaceae	Brucella	Brucella suis	3324765	3433
2585427653	Brucella suis bv. 2 PT09172	Proteobacteria	Alphaproteobacteria	Rhizobiales	Brucellaceae	Brucella	Brucella suis	3325042	3422
2597490102	Brucella suis 513UK	Proteobacteria	Alphaproteobacteria	Rhizobiales	Brucellaceae	Brucella	Brucella suis	3319697	3145
2597490105	Brucella suis bv. 3 686	Proteobacteria	Alphaproteobacteria	Rhizobiales	Brucellaceae	Brucella	Brucella suis	3297260	3149
2597490119	Brucella suis BSP	Proteobacteria	Alphaproteobacteria	Rhizobiales	Brucellaceae	Brucella	Brucella suis	3313865	3157
2623620635	Brucella suis bv. 1 1330 (re-annotation)	Proteobacteria	Alphaproteobacteria	Rhizobiales	Brucellaceae	Brucella	Brucella suis	3315175	3178
640753038	Ochrobactrum anthropi ATCC 49188	Proteobacteria	Alphaproteobacteria	Rhizobiales	Brucellaceae	Ochrobactrum	Ochrobactrum anthropi	5205777	4952
2597490146	Ochrobactrum anthropi OAB	Proteobacteria	Alphaproteobacteria	Rhizobiales	Brucellaceae	Ochrobactrum	Ochrobactrum anthropi	4901165	4705
648028034	Hyphomicrobium denitrificans ATCC 51888	Proteobacteria	Alphaproteobacteria	Rhizobiales	Hyphomicrobiaceae	Hyphomicrobium	Hyphomicrobium denitrificans	3638969	3600

2506783045	Hyphomicrobium denitrificans 1NES1	Proteobacteria	Alphaproteobacteria	Rhizobiales	Hyphomicrobiaceae	Hyphomicrobium	Hyphomicrobium denitrificans	3808687	3778
2528311098	Hyphomicrobium nitrativorans NL23	Proteobacteria	Alphaproteobacteria	Rhizobiales	Hyphomicrobiaceae	Hyphomicrobium	Hyphomicrobium nitrativorans	3653837	3480
650716042	Hyphomicrobium sp. MC1	Proteobacteria	Alphaproteobacteria	Rhizobiales	Hyphomicrobiaceae	Hyphomicrobium	Hyphomicrobium sp. MC1	4757528	4952
2511231054	Pelagibacterium halotolerans B2	Proteobacteria	Alphaproteobacteria	Rhizobiales	Hyphomicrobiaceae	Pelagibacterium	Pelagibacterium halotolerans	3948887	3940
649633090	Rhodomicrobium vannielii ATCC 17100	Proteobacteria	Alphaproteobacteria	Rhizobiales	Hyphomicrobiaceae	Rhodomicrobium	Rhodomicrobium vannielii	4014469	3739
641228497	Methylobacterium extorquens PA1	Proteobacteria	Alphaproteobacteria	Rhizobiales	Methylobacteriaceae	Methylobacterium	Methylobacterium extorquens	5471154	4939
643348563	Methylobacterium chloromethanicum CM4	Proteobacteria	Alphaproteobacteria	Rhizobiales	Methylobacteriaceae	Methylobacterium	Methylobacterium extorquens	6180732	5847
644736386	Methylobacterium extorquens AM1	Proteobacteria	Alphaproteobacteria	Rhizobiales	Methylobacteriaceae	Methylobacterium	Methylobacterium extorquens	6879778	6294
644736387	Methylobacterium extorquens DM4	Proteobacteria	Alphaproteobacteria	Rhizobiales	Methylobacteriaceae	Methylobacterium	Methylobacterium extorquens	6123851	5829
643348564	Methylobacterium nodulans ORS 2060	Proteobacteria	Alphaproteobacteria	Rhizobiales	Methylobacteriaceae	Methylobacterium	Methylobacterium nodulans	8839022	8885
642555139	Methylobacterium populi BJ001	Proteobacteria	Alphaproteobacteria	Rhizobiales	Methylobacteriaceae	Methylobacterium	Methylobacterium populi	5848997	5538
641522638	Methylobacterium radiotolerans JCM 2831	Proteobacteria	Alphaproteobacteria	Rhizobiales	Methylobacteriaceae	Methylobacterium	Methylobacterium radiotolerans	6899110	6510
641522639	Methylobacterium sp. 4-46	Proteobacteria	Alphaproteobacteria	Rhizobiales	Methylobacteriaceae	Methylobacterium	Methylobacterium sp. 4-46	7737025	7125
2540341127	Methylocystis sp. SC2	Proteobacteria	Alphaproteobacteria	Rhizobiales	Methylocystaceae	Methylocystis	Methylocystis sp. SC2	3773444	3716
637000160	Chelativorans sp. BNC1	Proteobacteria	Alphaproteobacteria	Rhizobiales	Phyllobacteriaceae	Chelativorans	Chelativorans sp. BNC1	4935185	4694
2509276022	Mesorhizobium australicum WSM2073	Proteobacteria	Alphaproteobacteria	Rhizobiales	Phyllobacteriaceae	Mesorhizobium	Mesorhizobium australicum	6200534	6080
649633066	Mesorhizobium ciceri bv. biserrulae WSM1271	Proteobacteria	Alphaproteobacteria	Rhizobiales	Phyllobacteriaceae	Mesorhizobium	Mesorhizobium ciceri	6690028	6531
2597489875	Mesorhizobium ciceri ca181	Proteobacteria	Alphaproteobacteria	Rhizobiales	Phyllobacteriaceae	Mesorhizobium	Mesorhizobium ciceri	7010078	6509
2588253730	Mesorhizobium huakuii 7653R	Proteobacteria	Alphaproteobacteria	Rhizobiales	Phyllobacteriaceae	Mesorhizobium	Mesorhizobium huakuii	6881675	7245
637000159	Mesorhizobium loti MAFF303099	Proteobacteria	Alphaproteobacteria	Rhizobiales	Phyllobacteriaceae	Mesorhizobium	Mesorhizobium loti	7596297	7356
2623620786	Mesorhizobium loti MAFF303099 (re-annotation)	Proteobacteria	Alphaproteobacteria	Rhizobiales	Phyllobacteriaceae	Mesorhizobium	Mesorhizobium loti	7596297	7453
2503198000	Mesorhizobium opportunistum WSM2075 (final version from ORNL)	Proteobacteria	Alphaproteobacteria	Rhizobiales	Phyllobacteriaceae	Mesorhizobium	Mesorhizobium opportunistum	6884444	6747
639279302	Agrobacterium tumefaciens C58-UWash	Proteobacteria	Alphaproteobacteria	Rhizobiales	Rhizobiaceae	Agrobacterium	Agrobacterium fabrum	5674064	5491
2623620883	Agrobacterium tumefaciens C58-UWash (re-annotation)	Proteobacteria	Alphaproteobacteria	Rhizobiales	Rhizobiaceae	Agrobacterium	Agrobacterium fabrum	5674258	5364
650716007	Agrobacterium sp. H13-3	Proteobacteria	Alphaproteobacteria	Rhizobiales	Rhizobiaceae	Agrobacterium	Agrobacterium sp. H13-3	5573770	5417
643348504	Rhizobium rhizogenes bv. II K84	Proteobacteria	Alphaproteobacteria	Rhizobiales	Rhizobiaceae	Agrobacterium	Agrobacterium tumefaciens	7273300	6744
2554235003	Agrobacterium tumefaciens WRT31	Proteobacteria	Alphaproteobacteria	Rhizobiales	Rhizobiaceae	Agrobacterium	Agrobacterium tumefaciens	5877155	5659
2558860256	Agrobacterium tumefaciens LBA4213 (Ach5)	Proteobacteria	Alphaproteobacteria	Rhizobiales	Rhizobiaceae	Agrobacterium	Agrobacterium tumefaciens	5630690	5499
643348505	Agrobacterium vitis bv. III S4	Proteobacteria	Alphaproteobacteria	Rhizobiales	Rhizobiaceae	Agrobacterium	Agrobacterium vitis	6320946	5455
644736336	Candidatus Liberibacter asiaticus psy62	Proteobacteria	Alphaproteobacteria	Rhizobiales	Rhizobiaceae	Candidatus Liberibacter	Candidatus Liberibacter asiaticus	1226704	1162
2540341131	Candidatus Liberibacter asiaticus gxpsy	Proteobacteria	Alphaproteobacteria	Rhizobiales	Rhizobiaceae	Candidatus Liberibacter	Candidatus Liberibacter asiaticus	1268237	1191
649633029	Candidatus Liberibacter solanacearum CLso-ZC1	Proteobacteria	Alphaproteobacteria	Rhizobiales	Rhizobiaceae	Candidatus Liberibacter	Candidatus Liberibacter solanacearum	1258278	1246
2521172690	Liberibacter crescens BT-1	Proteobacteria	Alphaproteobacteria	Rhizobiales	Rhizobiaceae	Candidatus Liberibacter	Liberibacter crescens	1504659	1432
2588253746	Ensifer adhaerens OV14	Proteobacteria	Alphaproteobacteria	Rhizobiales	Rhizobiaceae	Ensifer	Ensifer adhaerens	7709009	5525
2585427633	Neorhizobium galegae bv. officinalis HAMBI 1141	Proteobacteria	Alphaproteobacteria	Rhizobiales	Rhizobiaceae	Neorhizobium	Neorhizobium galegae	6413184	6213
2585427634	Neorhizobium galegae bv. orientalis HAMBI 540	Proteobacteria	Alphaproteobacteria	Rhizobiales	Rhizobiaceae	Neorhizobium	Neorhizobium galegae	6455027	6230
2510065076	Rhizobium leguminosarum bv. trifolii CB782	Proteobacteria	Alphaproteobacteria	Rhizobiales	Rhizobiaceae	Rhizobium	Rhizobium leguminosarum	6703653	6559
2597490046	Rhizobium sp. LPU83	Proteobacteria	Alphaproteobacteria	Rhizobiales	Rhizobiaceae	Rhizobium	Rhizobium sp. LPU83	4195305	4269
2524023199	Rhizobium tropici CIAT899	Proteobacteria	Alphaproteobacteria	Rhizobiales	Rhizobiaceae	Rhizobium	Rhizobium tropici	6686334	6494
643692032	Ensifer fredii NGR234	Proteobacteria	Alphaproteobacteria	Rhizobiales	Rhizobiaceae	Sinorhizobium	Sinorhizobium fredii	6891900	6437
2514885035	Ensifer fredii USDA 257	Proteobacteria	Alphaproteobacteria	Rhizobiales	Rhizobiaceae	Sinorhizobium	Sinorhizobium fredii	7032323	6855
2517572023	Ensifer fredii HH103	Proteobacteria	Alphaproteobacteria	Rhizobiales	Rhizobiaceae	Sinorhizobium	Sinorhizobium fredii	7221188	6851
640753051	Ensifer medicae WSM419	Proteobacteria	Alphaproteobacteria	Rhizobiales	Rhizobiaceae	Sinorhizobium	Sinorhizobium medicae	6817576	6599
637000269	Ensifer meliloti 1021	Proteobacteria	Alphaproteobacteria	Rhizobiales	Rhizobiaceae	Sinorhizobium	Sinorhizobium meliloti	6691694	6295
648276728	Ensifer meliloti BL225C	Proteobacteria	Alphaproteobacteria	Rhizobiales	Rhizobiaceae	Sinorhizobium	Sinorhizobium meliloti	6968865	6769
650716086	Ensifer meliloti AK83, DSM 23913	Proteobacteria	Alphaproteobacteria	Rhizobiales	Rhizobiaceae	Sinorhizobium	Sinorhizobium meliloti	7140471	7022
651053067	Ensifer meliloti SM11	Proteobacteria	Alphaproteobacteria	Rhizobiales	Rhizobiaceae	Sinorhizobium	Sinorhizobium meliloti	7173736	7156
2523533632	Ensifer meliloti GR4	Proteobacteria	Alphaproteobacteria	Rhizobiales	Rhizobiaceae	Sinorhizobium	Sinorhizobium meliloti	7139558	6826
2561511187	Ensifer meliloti Rm41	Proteobacteria	Alphaproteobacteria	Rhizobiales	Rhizobiaceae	Sinorhizobium	Sinorhizobium meliloti	7149690	6908
2562617130	Ensifer meliloti 2011	Proteobacteria	Alphaproteobacteria	Rhizobiales	Rhizobiaceae	Sinorhizobium	Sinorhizobium meliloti	6693185	8251
2597490149	Sinorhizobium meliloti RMO17	Proteobacteria	Alphaproteobacteria	Rhizobiales	Rhizobiaceae	Sinorhizobium	Sinorhizobium meliloti	6733414	6392
2623620857	Ensifer meliloti 1021 (re-annotation)	Proteobacteria	Alphaproteobacteria	Rhizobiales	Rhizobiaceae	Sinorhizobium	Sinorhizobium meliloti	6691694	6396
640753040	Parvibaculum lavamentivorans DS-1	Proteobacteria	Alphaproteobacteria	Rhizobiales	Rhodobiaceae	Parvibaculum	Parvibaculum lavamentivorans	3914745	3714
641228476	Azorhizobium caulinodans ORS 571	Proteobacteria	Alphaproteobacteria	Rhizobiales	Xanthobacteraceae	Azorhizobium	Azorhizobium caulinodans	5369772	4781
648028054	Starkeya novella DSM 506	Proteobacteria	Alphaproteobacteria	Rhizobiales	Xanthobacteraceae	Starkeya	Starkeya novella	4765023	4563

640753059	Xanthobacter autotrophicus Py2	Proteobacteria	Alphaproteobacteria	Rhizobiales	Xanthobacteraceae	Xanthobacter	Xanthobacter autotrophicus	5625098	5169
644736375	Hirschia baltica ATCC 49814	Proteobacteria	Alphaproteobacteria	Rhodobacterales	Hyphomonadaceae	Hirschia	Hirschia baltica	3540114	3266
637000135	Hyphomonas neptunium ATCC 15444	Proteobacteria	Alphaproteobacteria	Rhodobacterales	Hyphomonadaceae	Hyphomonas	Hyphomonas neptunium	3705021	3577
2623620830	Hyphomonas neptunium ATCC 15444 (re-annotation)	Proteobacteria	Alphaproteobacteria	Rhodobacterales	Hyphomonadaceae	Hyphomonas	Hyphomonas neptunium	3705021	3553
2501004205	Dinoroseobacter shibae DFL-12, DSM 16493	Proteobacteria	Alphaproteobacteria	Rhodobacterales	Rhodobacteraceae	Dinoroseobacter	Dinoroseobacter shibae	4417868	4244
637000137	Jannaschia sp. CCS1	Proteobacteria	Alphaproteobacteria	Rhodobacterales	Rhodobacteraceae	Jannaschia	Jannaschia sp. CCS1	4404049	4339
649633059	Ketogulonicigenium vulgare Y25	Proteobacteria	Alphaproteobacteria	Rhodobacterales	Rhodobacteraceae	Ketogulonicigenium	Ketogulonicigenium vulgare	3288404	3287
2511231188	Ketogulonicigenium vulgare WSH-001	Proteobacteria	Alphaproteobacteria	Rhodobacterales	Rhodobacteraceae	Ketogulonicigenium	Ketogulonicigenium vulgare	3277101	3125
2512564009	Leisingera methylohalidivorans MB2, DSM 14336	Proteobacteria	Alphaproteobacteria	Rhodobacterales	Rhodobacteraceae	Leisingera	Leisingera methylohalidivorans	4650996	4608
2510461047	Octadecabacter antarcticus 307	Proteobacteria	Alphaproteobacteria	Rhodobacterales	Rhodobacteraceae	Octadecabacter	Octadecabacter antarcticus	4875481	4939
2512564006	Octadecabacter arcticus 238, DSM 13978	Proteobacteria	Alphaproteobacteria	Rhodobacterales	Rhodobacteraceae	Octadecabacter	Octadecabacter arcticus	5478249	5149
2547132467	Paracoccus aminophilus JCM 7686	Proteobacteria	Alphaproteobacteria	Rhodobacterales	Rhodobacteraceae	Paracoccus	Paracoccus aminophilus	4917798	4642
639633048	Paracoccus denitrificans PD1222	Proteobacteria	Alphaproteobacteria	Rhodobacterales	Rhodobacteraceae	Paracoccus	Paracoccus denitrificans	5236194	5158
2510065028	Phaeobacter gallaeciensis 2.10	Proteobacteria	Alphaproteobacteria	Rhodobacterales	Rhodobacteraceae	Phaeobacter	Phaeobacter gallaeciensis	4160918	3798
2558309061	Phaeobacter gallaeciensis DSM 26640	Proteobacteria	Alphaproteobacteria	Rhodobacterales	Rhodobacteraceae	Phaeobacter	Phaeobacter gallaeciensis	4540155	4437
2510065029	Phaeobacter gallaeciensis DSM 17395	Proteobacteria	Alphaproteobacteria	Rhodobacterales	Rhodobacteraceae	Phaeobacter	Phaeobacter inhibens	4227134	3960
2548877138	Planktomarina temperata RCA23, DSM 22400 (RCA23)	Proteobacteria	Alphaproteobacteria	Rhodobacterales	Rhodobacteraceae	Planktomarina	Planktomarina temperata	3288122	3101
2511231065	Pseudovibrio sp. FO-BEG1	Proteobacteria	Alphaproteobacteria	Rhodobacterales	Rhodobacteraceae	Pseudovibrio	Pseudovibrio sp. FO-BEG1	5916782	5560
646564563	Rhodobacter capsulatus SB1003	Proteobacteria	Alphaproteobacteria	Rhodobacterales	Rhodobacteraceae	Rhodobacter	Rhodobacter capsulatus	3871920	3708
640069327	Rhodobacter sphaeroides 2.4.1, ATCC BAA-808	Proteobacteria	Alphaproteobacteria	Rhodobacterales	Rhodobacteraceae	Rhodobacter	Rhodobacter sphaeroides	4603060	4383
640069328	Rhodobacter sphaeroides ATCC 17029	Proteobacteria	Alphaproteobacteria	Rhodobacterales	Rhodobacteraceae	Rhodobacter	Rhodobacter sphaeroides	4489380	4268
640427138	Rhodobacter sphaeroides ATCC 17025	Proteobacteria	Alphaproteobacteria	Rhodobacterales	Rhodobacteraceae	Rhodobacter	Rhodobacter sphaeroides	4557127	4475
643348570	Rhodobacter sphaeroides KD131	Proteobacteria	Alphaproteobacteria	Rhodobacterales	Rhodobacteraceae	Rhodobacter	Rhodobacter sphaeroides	4711139	4635
639633056	Roseobacter denitrificans OCh 114	Proteobacteria	Alphaproteobacteria	Rhodobacterales	Rhodobacteraceae	Roseobacter	Roseobacter denitrificans	4331234	4201
2623620350	Roseobacter denitrificans OCh 114 (re-annotation)	Proteobacteria	Alphaproteobacteria	Rhodobacterales	Rhodobacteraceae	Roseobacter	Roseobacter denitrificans	4331234	4144
2510065042	Roseobacter litoralis Och 149	Proteobacteria	Alphaproteobacteria	Rhodobacterales	Rhodobacteraceae	Roseobacter	Roseobacter litoralis	4745450	4668
637000267	Ruegeria pomeroyi DSS-3	Proteobacteria	Alphaproteobacteria	Rhodobacterales	Rhodobacteraceae	Ruegeria	Ruegeria pomeroyi	4601053	4355
2623620655	Ruegeria pomeroyi DSS-3 (re-annotation)	Proteobacteria	Alphaproteobacteria	Rhodobacterales	Rhodobacteraceae	Ruegeria	Ruegeria pomeroyi	4601048	4461
637000268	Ruegeria sp. TM1040	Proteobacteria	Alphaproteobacteria	Rhodobacterales	Rhodobacteraceae	Ruegeria	Ruegeria sp. TM1040	4153699	3964
644736321	Acetobacter pasteurianus IFO 3283-01	Proteobacteria	Alphaproteobacteria	Rhodospirillales	Acetobacteraceae	Acetobacter	Acetobacter pasteurianus	3340249	3122
646862301	Acetobacter pasteurianus IFO 3283-01-42C	Proteobacteria	Alphaproteobacteria	Rhodospirillales	Acetobacteraceae	Acetobacter	Acetobacter pasteurianus	3247995	3050
646862302	Acetobacter pasteurianus IFO 3283-03	Proteobacteria	Alphaproteobacteria	Rhodospirillales	Acetobacteraceae	Acetobacter	Acetobacter pasteurianus	3339669	3120
646862303	Acetobacter pasteurianus IFO 3283-07	Proteobacteria	Alphaproteobacteria	Rhodospirillales	Acetobacteraceae	Acetobacter	Acetobacter pasteurianus	3338426	3119
646862304	Acetobacter pasteurianus IFO 3283-12	Proteobacteria	Alphaproteobacteria	Rhodospirillales	Acetobacteraceae	Acetobacter	Acetobacter pasteurianus	3336990	3118
646862305	Acetobacter pasteurianus IFO 3283-22	Proteobacteria	Alphaproteobacteria	Rhodospirillales	Acetobacteraceae	Acetobacter	Acetobacter pasteurianus	3339649	3120
646862306	Acetobacter pasteurianus IFO 3283-26	Proteobacteria	Alphaproteobacteria	Rhodospirillales	Acetobacteraceae	Acetobacter	Acetobacter pasteurianus	3339683	3120
646862307	Acetobacter pasteurianus IFO 3283-32	Proteobacteria	Alphaproteobacteria	Rhodospirillales	Acetobacteraceae	Acetobacter	Acetobacter pasteurianus	3337040	3118
2561511215	Acetobacter pasteurianus 386B	Proteobacteria	Alphaproteobacteria	Rhodospirillales	Acetobacteraceae	Acetobacter	Acetobacter pasteurianus	3078647	2942
640427101	Acidiphilium cryptum JF-5	Proteobacteria	Alphaproteobacteria	Rhodospirillales	Acetobacteraceae	Acidiphilium	Acidiphilium cryptum	3963080	3701
650716002	Acidiphilium multivorum AIU301	Proteobacteria	Alphaproteobacteria	Rhodospirillales	Acetobacteraceae	Acidiphilium	Acidiphilium multivorum	4214744	4004
641228493	Gluconacetobacter diazotrophicus PA1 5	Proteobacteria	Alphaproteobacteria	Rhodospirillales	Acetobacteraceae	Gluconacetobacter	Gluconacetobacter diazotrophicus	3999591	3997
643348555	Gluconacetobacter diazotrophicus PA1 5	Proteobacteria	Alphaproteobacteria	Rhodospirillales	Acetobacteraceae	Gluconacetobacter	Gluconacetobacter diazotrophicus	3914947	3633
637000122	Gluconobacter oxydans 621H	Proteobacteria	Alphaproteobacteria	Rhodospirillales	Acetobacteraceae	Gluconobacter	Gluconobacter oxydans	2922384	2742
2540341122	Gluconobacter oxydans H24	Proteobacteria	Alphaproteobacteria	Rhodospirillales	Acetobacteraceae	Gluconobacter	Gluconobacter oxydans	3602424	3543
2556793022	Gluconobacter oxydans DSM 3504 (GLS)	Proteobacteria	Alphaproteobacteria	Rhodospirillales	Acetobacteraceae	Gluconobacter	Gluconobacter oxydans	2882436	2616
2623620996	Gluconobacter oxydans 621H (re-annotation)	Proteobacteria	Alphaproteobacteria	Rhodospirillales	Acetobacteraceae	Gluconobacter	Gluconobacter oxydans	2922384	2910
637000123	Granulibacter bethesdensis CGDNIH1	Proteobacteria	Alphaproteobacteria	Rhodospirillales	Acetobacteraceae	Granulibacter	Granulibacter bethesdensis	2708355	2510
2558860199	Granulibacter bethesdensis CGDNIH3	Proteobacteria	Alphaproteobacteria	Rhodospirillales	Acetobacteraceae	Granulibacter	Granulibacter bethesdensis	2711143	2682
2558860205	Granulibacter bethesdensis CGDNIH2	Proteobacteria	Alphaproteobacteria	Rhodospirillales	Acetobacteraceae	Granulibacter	Granulibacter bethesdensis	2736589	2725
2558860206	Granulibacter bethesdensis CGDNIH4	Proteobacteria	Alphaproteobacteria	Rhodospirillales	Acetobacteraceae	Granulibacter	Granulibacter bethesdensis	2715337	2681
2623620923	Granulibacter bethesdensis CGDNIH1 (re-annotation)	Proteobacteria	Alphaproteobacteria	Rhodospirillales	Acetobacteraceae	Granulibacter	Granulibacter bethesdensis	2708355	2498
2513237191	Gluconacetobacter xylinus NBRC 3288	Proteobacteria	Alphaproteobacteria	Rhodospirillales	Acetobacteraceae	Komagataeibacter	Komagataeibacter medellinensis	3513191	3269
2558860240	Gluconacetobacter xylinus E25	Proteobacteria	Alphaproteobacteria	Rhodospirillales	Acetobacteraceae	Komagataeibacter	Komagataeibacter xylinus	3905082	3745
2511231222	Azospirillum brasilense Sp245	Proteobacteria	Alphaproteobacteria	Rhodospirillales	Rhodospirillaceae	Azospirillum	Azospirillum brasilense	7530241	7962
2576861778	Azospirillum brasilense Az39	Proteobacteria	Alphaproteobacteria	Rhodospirillales	Rhodospirillaceae	Azospirillum	Azospirillum brasilense	7421756	6405

646311907	Azospirillum sp. B510	Proteobacteria	Alphaproteobacteria	Rhodospirillales	Rhodospirillaceae	Azospirillum	Azospirillum lipoferum	7599738	6417
2511231221	Azospirillum lipoferum 4B	Proteobacteria	Alphaproteobacteria	Rhodospirillales	Rhodospirillaceae	Azospirillum	Azospirillum lipoferum	6846400	6349
637000155	Magnetospirillum magneticum AMB-1	Proteobacteria	Alphaproteobacteria	Rhodospirillales	Rhodospirillaceae	Magnetospirillum	Magnetospirillum magneticum	4967148	4627
2623620854	Magnetospirillum magneticum AMB-1 (re-annotation)	Proteobacteria	Alphaproteobacteria	Rhodospirillales	Rhodospirillaceae	Magnetospirillum	Magnetospirillum magneticum	4967148	4686
643348571	Rhodocista centenaria SW	Proteobacteria	Alphaproteobacteria	Rhodospirillales	Rhodospirillaceae	Rhodospirillum	Rhodospirillum centenum	4355548	4065
2513237204	Rhodospirillum photometricum DSM 122	Proteobacteria	Alphaproteobacteria	Rhodospirillales	Rhodospirillaceae	Rhodospirillum	Rhodospirillum photometricum	3876289	3376
637000241	Rhodospirillum rubrum S1, ATCC 11170	Proteobacteria	Alphaproteobacteria	Rhodospirillales	Rhodospirillaceae	Rhodospirillum	Rhodospirillum rubrum	4406557	3933
2511231162	Rhodospirillum rubrum F11	Proteobacteria	Alphaproteobacteria	Rhodospirillales	Rhodospirillaceae	Rhodospirillum	Rhodospirillum rubrum	4352825	3945
2519103105	Tistrella mobilis KA081020-065	Proteobacteria	Alphaproteobacteria	Rhodospirillales	Rhodospirillaceae	Tistrella	Tistrella mobilis	6513401	5851
637000103	Erythrobacter litoralis HTCC2594	Proteobacteria	Alphaproteobacteria	Sphingomonadales	Erythrobacteraceae	Erythrobacter	Erythrobacter litoralis	3052398	3068
2623620814	Erythrobacter litoralis HTCC2594 (re-annotation)	Proteobacteria	Alphaproteobacteria	Sphingomonadales	Erythrobacteraceae	Erythrobacter	Erythrobacter litoralis	3052398	3012
640427126	Novosphingobium aromaticivorans DSM 12444	Proteobacteria	Alphaproteobacteria	Sphingomonadales	Sphingomonadaceae	Novosphingobium	Novosphingobium aromaticivorans	4233314	4038
2503904014	Sphingobium chlorophenolicum L-1	Proteobacteria	Alphaproteobacteria	Sphingomonadales	Sphingomonadaceae	Sphingobium	Sphingobium chlorophenolicum	4573221	4224
646564570	Sphingobium japonicum UT26S	Proteobacteria	Alphaproteobacteria	Sphingomonadales	Sphingomonadaceae	Sphingobium	Sphingobium japonicum	4424862	4460
2511231055	Sphingobium sp. SYK-6	Proteobacteria	Alphaproteobacteria	Sphingomonadales	Sphingomonadaceae	Sphingobium	Sphingobium sp. SYK-6	4348133	4121
2558860262	Sphingomonas sanxanigenens DSM 19645, NX02	Proteobacteria	Alphaproteobacteria	Sphingomonadales	Sphingomonadaceae	Sphingomonas	Sphingomonas sanxanigenens	6205897	5924
2561511202	Sphingomonas sp. MM-1	Proteobacteria	Alphaproteobacteria	Sphingomonadales	Sphingomonadaceae	Sphingomonas	Sphingomonas sp. MM-1	4633613	4418
640427144	Sphingomonas wittichii RW1	Proteobacteria	Alphaproteobacteria	Sphingomonadales	Sphingomonadaceae	Sphingomonas	Sphingomonas wittichii	5915246	5463
637000356	Zymomonas mobilis mobilis ZM4	Proteobacteria	Alphaproteobacteria	Sphingomonadales	Sphingomonadaceae	Zymomonas	Zymomonas mobilis	2056416	2065
645058785	Zymomonas mobilis mobilis T.H.Delft 1, ATCC 10988	Proteobacteria	Alphaproteobacteria	Sphingomonadales	Sphingomonadaceae	Zymomonas	Zymomonas mobilis	2095199	1894
646311969	Zymomonas mobilis mobilis NCIB 11163	Proteobacteria	Alphaproteobacteria	Sphingomonadales	Sphingomonadaceae	Zymomonas	Zymomonas mobilis	2223520	1978
650716107	Zymomonas mobilis pomaceae Barker 1, ATCC 29192	Proteobacteria	Alphaproteobacteria	Sphingomonadales	Sphingomonadaceae	Zymomonas	Zymomonas mobilis	2061413	1838
2545555880	Zymomonas mobilis mobilis CP4	Proteobacteria	Alphaproteobacteria	Sphingomonadales	Sphingomonadaceae	Zymomonas	Zymomonas mobilis	2163236	1933
2558860209	Zymomonas mobilis mobilis NRRL B-12526	Proteobacteria	Alphaproteobacteria	Sphingomonadales	Sphingomonadaceae	Zymomonas	Zymomonas mobilis	2178998	1922
2558860210	Zymomonas mobilis mobilis CP4	Proteobacteria	Alphaproteobacteria	Sphingomonadales	Sphingomonadaceae	Zymomonas	Zymomonas mobilis	2147181	1895
2623620802	Zymomonas mobilis mobilis ZM4 (re-annotation)	Proteobacteria	Alphaproteobacteria	Sphingomonadales	Sphingomonadaceae	Zymomonas	Zymomonas mobilis	2056363	1817
646564516	Candidatus Puniceispirillum marinum IMCC1322	Proteobacteria	Alphaproteobacteria	unclassified	unclassified	Candidatus Puniceispirillum	Candidatus Puniceispirillum marinum	2753527	2582
2512047034	Polymorphum gilvum SL003B-26A1	Proteobacteria	Alphaproteobacteria	unclassified	unclassified	unclassified	Polymorphum gilvum	4718963	4453
649633001	Achromobacter xylosoxidans A8	Proteobacteria	Betaproteobacteria	Burkholderiales	Alcaligenaceae	Achromobacter	Achromobacter xylosoxidans	7359146	6885
2554235314	Achromobacter xylosoxidans NH44784-1996	Proteobacteria	Betaproteobacteria	Burkholderiales	Alcaligenaceae	Achromobacter	Achromobacter xylosoxidans	6916670	6446
2558309085	Achromobacter xylosoxidans NBRC 15126, ATCC 27061	Proteobacteria	Betaproteobacteria	Burkholderiales	Alcaligenaceae	Achromobacter	Achromobacter xylosoxidans	6683584	6146
2515154053	Advenella kashmirensis WT001	Proteobacteria	Betaproteobacteria	Burkholderiales	Alcaligenaceae	Advenella	Advenella kashmirensis	4423879	3980
2548877156	Advenella mimigardefordensis DPN7, DSM 17166 (MIMI)	Proteobacteria	Betaproteobacteria	Burkholderiales	Alcaligenaceae	Advenella	Advenella mimigardefordensis	4764126	4136
641522607	Bordetella avium 197N	Proteobacteria	Betaproteobacteria	Burkholderiales	Alcaligenaceae	Bordetella	Bordetella avium	3732255	3464
637000032	Bordetella bronchiseptica RB50	Proteobacteria	Betaproteobacteria	Burkholderiales	Alcaligenaceae	Bordetella	Bordetella bronchiseptica	5339179	5086
2619619090	Bordetella bronchiseptica 253	Proteobacteria	Betaproteobacteria	Burkholderiales	Alcaligenaceae	Bordetella	Bordetella bronchiseptica	5264383	4989
2619619093	Bordetella bronchiseptica MO149	Proteobacteria	Betaproteobacteria	Burkholderiales	Alcaligenaceae	Bordetella	Bordetella bronchiseptica	5091817	4757
2623620905	Bordetella bronchiseptica RB50 (re-annotation)	Proteobacteria	Betaproteobacteria	Burkholderiales	Alcaligenaceae	Bordetella	Bordetella bronchiseptica	5339179	5073
637000033	Bordetella parapertussis 12822	Proteobacteria	Betaproteobacteria	Burkholderiales	Alcaligenaceae	Bordetella	Bordetella parapertussis	4773551	4447
2623620628	Bordetella parapertussis 12822 (re-annotation)	Proteobacteria	Betaproteobacteria	Burkholderiales	Alcaligenaceae	Bordetella	Bordetella parapertussis	4773551	4583
637000034	Bordetella pertussis Tohama I	Proteobacteria	Betaproteobacteria	Burkholderiales	Alcaligenaceae	Bordetella	Bordetella pertussis	4086189	3833
651053009	Bordetella pertussis CS	Proteobacteria	Betaproteobacteria	Burkholderiales	Alcaligenaceae	Bordetella	Bordetella pertussis	4124236	3516
2623620629	Bordetella pertussis Tohama I (re-annotation)	Proteobacteria	Betaproteobacteria	Burkholderiales	Alcaligenaceae	Bordetella	Bordetella pertussis	4086189	3953
641228479	Bordetella petrii Se-1111R, DSM 12804	Proteobacteria	Betaproteobacteria	Burkholderiales	Alcaligenaceae	Bordetella	Bordetella petrii	5287950	5092
2619619095	Castellaniella defragrans 65Phen	Proteobacteria	Betaproteobacteria	Burkholderiales	Alcaligenaceae	Castellaniella	Castellaniella defragrans	3952818	3577
650716078	Pusillimonas sp. T7-7	Proteobacteria	Betaproteobacteria	Burkholderiales	Alcaligenaceae	Pusillimonas	Pusillimonas sp. T7-7	3924810	3826
637000047	Burkholderia cepacia AMMD	Proteobacteria	Betaproteobacteria	Burkholderiales	Burkholderiaceae	Burkholderia	Burkholderia ambifaria	7528567	6738
641522608	Burkholderia ambifaria MC40-6	Proteobacteria	Betaproteobacteria	Burkholderiales	Burkholderiaceae	Burkholderia	Burkholderia ambifaria	7642536	6878
637000046	Burkholderia cenocepacia AU 1054	Proteobacteria	Betaproteobacteria	Burkholderiales	Burkholderiaceae	Burkholderia	Burkholderia cenocepacia	7279116	6637
639633014	Burkholderia cenocepacia HI2424	Proteobacteria	Betaproteobacteria	Burkholderiales	Burkholderiaceae	Burkholderia	Burkholderia cenocepacia	7702840	7050
641522609	Burkholderia cenocepacia MC0-3	Proteobacteria	Betaproteobacteria	Burkholderiales	Burkholderiaceae	Burkholderia	Burkholderia cenocepacia	7971389	7169
643348523	Burkholderia cenocepacia gv. III J2315	Proteobacteria	Betaproteobacteria	Burkholderiales	Burkholderiaceae	Burkholderia	Burkholderia cenocepacia	8055782	7229
2597490136	Burkholderia cenocepacia DDS 22E-1	Proteobacteria	Betaproteobacteria	Burkholderiales	Burkholderiaceae	Burkholderia	Burkholderia cenocepacia	8045250	7170
2597490103	Burkholderia cepacia DDS 7H-2	Proteobacteria	Betaproteobacteria	Burkholderiales	Burkholderiaceae	Burkholderia	Burkholderia cepacia	8147114	7432
2526164745	Burkholderia dolosa PC543 (LMG 19468)	Proteobacteria	Betaproteobacteria	Burkholderiales	Burkholderiaceae	Burkholderia	Burkholderia dolosa	6294588	5711

650716013	Burkholderia gladioli BSR3	Proteobacteria	Betaproteobacteria	Burkholderiales	Burkholderiaceae	Burkholderia	Burkholderia gladioli	9052299	7493
643692013	Burkholderia glumae BGR1	Proteobacteria	Betaproteobacteria	Burkholderiales	Burkholderiaceae	Burkholderia	Burkholderia glumae	7284683	5854
2568526014	Burkholderia glumae PG1 (BG)	Proteobacteria	Betaproteobacteria	Burkholderiales	Burkholderiaceae	Burkholderia	Burkholderia glumae	7896538	6583
637000051	Burkholderia cepacia 383	Proteobacteria	Betaproteobacteria	Burkholderiales	Burkholderiaceae	Burkholderia	Burkholderia lata	8676277	7828
637000048	Burkholderia mallei ATCC 23344	Proteobacteria	Betaproteobacteria	Burkholderiales	Burkholderiaceae	Burkholderia	Burkholderia mallei	5835527	5500
639633015	Burkholderia mallei SAVP1	Proteobacteria	Betaproteobacteria	Burkholderiales	Burkholderiaceae	Burkholderia	Burkholderia mallei	5232401	5309
640069303	Burkholderia mallei NCTC 10229	Proteobacteria	Betaproteobacteria	Burkholderiales	Burkholderiaceae	Burkholderia	Burkholderia mallei	5742303	5635
640069304	Burkholderia mallei NCTC 10247	Proteobacteria	Betaproteobacteria	Burkholderiales	Burkholderiaceae	Burkholderia	Burkholderia mallei	5848380	5978
2597490099	Burkholderia mallei BMY	Proteobacteria	Betaproteobacteria	Burkholderiales	Burkholderiaceae	Burkholderia	Burkholderia mallei	5647769	4938
2597490104	Burkholderia mallei 23344	Proteobacteria	Betaproteobacteria	Burkholderiales	Burkholderiaceae	Burkholderia	Burkholderia mallei	5625292	4935
2597490120	Burkholderia mallei BMQ	Proteobacteria	Betaproteobacteria	Burkholderiales	Burkholderiaceae	Burkholderia	Burkholderia mallei	5630231	4950
2597490121	Burkholderia mallei 092700E	Proteobacteria	Betaproteobacteria	Burkholderiales	Burkholderiaceae	Burkholderia	Burkholderia mallei	5827656	5068
2597490126	Burkholderia mallei FMH 23344	Proteobacteria	Betaproteobacteria	Burkholderiales	Burkholderiaceae	Burkholderia	Burkholderia mallei	5835541	5084
2597490138	Burkholderia mallei BMK	Proteobacteria	Betaproteobacteria	Burkholderiales	Burkholderiaceae	Burkholderia	Burkholderia mallei	5874930	5130
2623620294	Burkholderia mallei SAVP1 (re-annotation)	Proteobacteria	Betaproteobacteria	Burkholderiales	Burkholderiaceae	Burkholderia	Burkholderia mallei	5232401	4564
2623620320	Burkholderia mallei NCTC 10247 (re-annotation)	Proteobacteria	Betaproteobacteria	Burkholderiales	Burkholderiaceae	Burkholderia	Burkholderia mallei	5848380	5092
2623620367	Burkholderia mallei NCTC 10229 (re-annotation)	Proteobacteria	Betaproteobacteria	Burkholderiales	Burkholderiaceae	Burkholderia	Burkholderia mallei	5742303	4988
2623621003	Burkholderia mallei ATCC 23344 (re-annotation)	Proteobacteria	Betaproteobacteria	Burkholderiales	Burkholderiaceae	Burkholderia	Burkholderia mallei	5835527	5074
641228482	Burkholderia multivorans ATCC 17616	Proteobacteria	Betaproteobacteria	Burkholderiales	Burkholderiaceae	Burkholderia	Burkholderia multivorans	7008622	6373
642555111	Burkholderia multivorans ATCC 17616	Proteobacteria	Betaproteobacteria	Burkholderiales	Burkholderiaceae	Burkholderia	Burkholderia multivorans	7008810	6193
2597490133	Burkholderia multivorans DDS 15A-1	Proteobacteria	Betaproteobacteria	Burkholderiales	Burkholderiaceae	Burkholderia	Burkholderia multivorans	7281867	6596
2597490124	Burkholderia oklahomensis BDU	Proteobacteria	Betaproteobacteria	Burkholderiales	Burkholderiaceae	Burkholderia	Burkholderia oklahomensis	7313673	6367
2518645580	Burkholderia phenoliruptrix BR3459	Proteobacteria	Betaproteobacteria	Burkholderiales	Burkholderiaceae	Burkholderia	Burkholderia phenoliruptrix	7651131	6605
642555112	Burkholderia phymatum STM815	Proteobacteria	Betaproteobacteria	Burkholderiales	Burkholderiaceae	Burkholderia	Burkholderia phymatum	8676562	7574
642555113	Burkholderia phytofirmans PsJN	Proteobacteria	Betaproteobacteria	Burkholderiales	Burkholderiaceae	Burkholderia	Burkholderia phytofirmans	8214658	7487
637000050	Burkholderia pseudomallei K96243	Proteobacteria	Betaproteobacteria	Burkholderiales	Burkholderiaceae	Burkholderia	Burkholderia pseudomallei	7247547	5942
640069305	Burkholderia pseudomallei 1106a	Proteobacteria	Betaproteobacteria	Burkholderiales	Burkholderiaceae	Burkholderia	Burkholderia pseudomallei	7089249	7278
640069306	Burkholderia pseudomallei 668	Proteobacteria	Betaproteobacteria	Burkholderiales	Burkholderiaceae	Burkholderia	Burkholderia pseudomallei	7040403	7322
2513237222	Burkholderia pseudomallei 1026b	Proteobacteria	Betaproteobacteria	Burkholderiales	Burkholderiaceae	Burkholderia	Burkholderia pseudomallei	7231415	6262
2521172686	Burkholderia pseudomallei BPC006	Proteobacteria	Betaproteobacteria	Burkholderiales	Burkholderiaceae	Burkholderia	Burkholderia pseudomallei	7155061	7230
2554235374	Burkholderia pseudomallei NCTC 13179	Proteobacteria	Betaproteobacteria	Burkholderiales	Burkholderiaceae	Burkholderia	Burkholderia pseudomallei	7337157	6157
2558860228	Burkholderia pseudomallei NAU20B-16	Proteobacteria	Betaproteobacteria	Burkholderiales	Burkholderiaceae	Burkholderia	Burkholderia pseudomallei	7313851	6056
2558860229	Burkholderia pseudomallei MSHR511	Proteobacteria	Betaproteobacteria	Burkholderiales	Burkholderiaceae	Burkholderia	Burkholderia pseudomallei	7316085	6045
2558860230	Burkholderia pseudomallei MSHR146	Proteobacteria	Betaproteobacteria	Burkholderiales	Burkholderiaceae	Burkholderia	Burkholderia pseudomallei	7313103	6045
2558860231	Burkholderia pseudomallei NCTC 13178	Proteobacteria	Betaproteobacteria	Burkholderiales	Burkholderiaceae	Burkholderia	Burkholderia pseudomallei	7391364	6170
2563366587	Burkholderia pseudomallei MSHR305	Proteobacteria	Betaproteobacteria	Burkholderiales	Burkholderiaceae	Burkholderia	Burkholderia pseudomallei	7428072	6188
2563366619	Burkholderia pseudomallei MSHR520	Proteobacteria	Betaproteobacteria	Burkholderiales	Burkholderiaceae	Burkholderia	Burkholderia pseudomallei	7450511	6205
2597490066	Burkholderia pseudomallei BGR	Proteobacteria	Betaproteobacteria	Burkholderiales	Burkholderiaceae	Burkholderia	Burkholderia pseudomallei	7231385	6036
2597490074	Burkholderia pseudomallei MSHR5858	Proteobacteria	Betaproteobacteria	Burkholderiales	Burkholderiaceae	Burkholderia	Burkholderia pseudomallei	7072128	5841
2597490093	Burkholderia pseudomallei BEO	Proteobacteria	Betaproteobacteria	Burkholderiales	Burkholderiaceae	Burkholderia	Burkholderia pseudomallei	7086433	5827
2597490106	Burkholderia pseudomallei BEZ	Proteobacteria	Betaproteobacteria	Burkholderiales	Burkholderiaceae	Burkholderia	Burkholderia pseudomallei	7027950	5867
2597490109	Burkholderia pseudomallei BEX	Proteobacteria	Betaproteobacteria	Burkholderiales	Burkholderiaceae	Burkholderia	Burkholderia pseudomallei	7266604	6027
2597490122	Burkholderia pseudomallei Mahidol-1106a	Proteobacteria	Betaproteobacteria	Burkholderiales	Burkholderiaceae	Burkholderia	Burkholderia pseudomallei	7085397	5824
2597490123	Burkholderia pseudomallei MSHR5848	Proteobacteria	Betaproteobacteria	Burkholderiales	Burkholderiaceae	Burkholderia	Burkholderia pseudomallei	7290434	6070
2597490125	Burkholderia pseudomallei MSHR5855	Proteobacteria	Betaproteobacteria	Burkholderiales	Burkholderiaceae	Burkholderia	Burkholderia pseudomallei	7297804	6078
2597490134	Burkholderia pseudomallei BFB	Proteobacteria	Betaproteobacteria	Burkholderiales	Burkholderiaceae	Burkholderia	Burkholderia pseudomallei	7354216	6130
2597490143	Burkholderia pseudomallei HBPUB10303a	Proteobacteria	Betaproteobacteria	Burkholderiales	Burkholderiaceae	Burkholderia	Burkholderia pseudomallei	7178176	5903
2597490152	Burkholderia pseudomallei BSR	Proteobacteria	Betaproteobacteria	Burkholderiales	Burkholderiaceae	Burkholderia	Burkholderia pseudomallei	7272702	6017
2599185119	Burkholderia pseudomallei HBPUB10134a	Proteobacteria	Betaproteobacteria	Burkholderiales	Burkholderiaceae	Burkholderia	Burkholderia pseudomallei	7218403	5923
2623620318	Burkholderia pseudomallei 1106a (re-annotation)	Proteobacteria	Betaproteobacteria	Burkholderiales	Burkholderiaceae	Burkholderia	Burkholderia pseudomallei	7089249	5841
2623620319	Burkholderia pseudomallei 668 (re-annotation)	Proteobacteria	Betaproteobacteria	Burkholderiales	Burkholderiaceae	Burkholderia	Burkholderia pseudomallei	7040403	5864
2623620907	Burkholderia pseudomallei K96243 (re-annotation)	Proteobacteria	Betaproteobacteria	Burkholderiales	Burkholderiaceae	Burkholderia	Burkholderia pseudomallei	7247547	6037
650716014	Burkholderia rhizoxinica HKI 454	Proteobacteria	Betaproteobacteria	Burkholderiales	Burkholderiaceae	Burkholderia	Burkholderia rhizoxinica	3750138	3938
649633021	Burkholderia sp. CCGE1001	Proteobacteria	Betaproteobacteria	Burkholderiales	Burkholderiaceae	Burkholderia	Burkholderia sp. CCGE1001	6833751	6157
646564515	Burkholderia sp. CCGE1002	Proteobacteria	Betaproteobacteria	Burkholderiales	Burkholderiaceae	Burkholderia	Burkholderia sp. CCGE1002	7884858	7358

648028011	Burkholderia sp. CCGE1003	Proteobacteria	Betaproteobacteria	Burkholderiales	Burkholderiaceae	Burkholderia	Burkholderia sp. CCGE1003	7043595	6188
2519103095	Burkholderia sp. KJ006	Proteobacteria	Betaproteobacteria	Burkholderiales	Burkholderiaceae	Burkholderia	Burkholderia sp. KJ006	6629912	6143
2597489944	Burkholderia sp. RPE64	Proteobacteria	Betaproteobacteria	Burkholderiales	Burkholderiaceae	Burkholderia	Burkholderia sp. RPE64	6964487	6399
2511231144	Burkholderia sp. YI23	Proteobacteria	Betaproteobacteria	Burkholderiales	Burkholderiaceae	Burkholderia	Burkholderia sp. YI23	8896411	7886
637000052	Burkholderia thailandensis E264, ATCC 700388	Proteobacteria	Betaproteobacteria	Burkholderiales	Burkholderiaceae	Burkholderia	Burkholderia thailandensis	6723972	5727
2540341176	Burkholderia thailandensis MSMB121	Proteobacteria	Betaproteobacteria	Burkholderiales	Burkholderiaceae	Burkholderia	Burkholderia thailandensis	6731379	5840
2558860232	Burkholderia thailandensis H0587	Proteobacteria	Betaproteobacteria	Burkholderiales	Burkholderiaceae	Burkholderia	Burkholderia thailandensis	6768375	5707
2558860233	Burkholderia thailandensis 2002721723	Proteobacteria	Betaproteobacteria	Burkholderiales	Burkholderiaceae	Burkholderia	Burkholderia thailandensis	6577133	5611
2558860234	Burkholderia thailandensis E444	Proteobacteria	Betaproteobacteria	Burkholderiales	Burkholderiaceae	Burkholderia	Burkholderia thailandensis	6651696	5659
2585427618	Burkholderia thailandensis USAMRU Malaysia #20	Proteobacteria	Betaproteobacteria	Burkholderiales	Burkholderiaceae	Burkholderia	Burkholderia thailandensis	6684359	5708
2623620862	Burkholderia thailandensis E264, ATCC 700388 (re-annotation)	Proteobacteria	Betaproteobacteria	Burkholderiales	Burkholderiaceae	Burkholderia	Burkholderia thailandensis	6723972	5729
640069307	Burkholderia vietnamiensis G4	Proteobacteria	Betaproteobacteria	Burkholderiales	Burkholderiaceae	Burkholderia	Burkholderia vietnamiensis	8391070	7880
637000053	Burkholderia xenovorans LB400	Proteobacteria	Betaproteobacteria	Burkholderiales	Burkholderiaceae	Burkholderia	Burkholderia xenovorans	9731138	9059
2597490151	Burkholderia xenovorans BXA	Proteobacteria	Betaproteobacteria	Burkholderiales	Burkholderiaceae	Burkholderia	Burkholderia xenovorans	9702951	8768
637000230	Cupriavidus metallidurans CH34	Proteobacteria	Betaproteobacteria	Burkholderiales	Burkholderiaceae	Cupriavidus	Cupriavidus metallidurans	6913352	6430
640427136	Ralstonia eutropha H16	Proteobacteria	Betaproteobacteria	Burkholderiales	Burkholderiaceae	Cupriavidus	Cupriavidus necator	7416678	6718
650716031	Cupriavidus necator N-1, ATCC 43291	Proteobacteria	Betaproteobacteria	Burkholderiales	Burkholderiaceae	Cupriavidus	Cupriavidus necator	8480857	7915
2623620228	Ralstonia eutropha H16 (re-annotation)	Proteobacteria	Betaproteobacteria	Burkholderiales	Burkholderiaceae	Cupriavidus	Cupriavidus necator	7416678	6842
637000229	Ralstonia eutropha JMP134	Proteobacteria	Betaproteobacteria	Burkholderiales	Burkholderiaceae	Cupriavidus	Cupriavidus pinatubonensis	7255290	6631
644736347	Cupriavidus taiwanensis LMG 19424	Proteobacteria	Betaproteobacteria	Burkholderiales	Burkholderiaceae	Cupriavidus	Cupriavidus taiwanensis	6476522	5986
2558309055	Pandoraea pnomenusa 3kgm	Proteobacteria	Betaproteobacteria	Burkholderiales	Burkholderiaceae	Pandoraea	Pandoraea pnomenusa	5435131	4951
2571042567	Pandoraea pnomenusa RB38	Proteobacteria	Betaproteobacteria	Burkholderiales	Burkholderiaceae	Pandoraea	Pandoraea pnomenusa	5378872	4766
642555151	Ralstonia pickettii 12J	Proteobacteria	Betaproteobacteria	Burkholderiales	Burkholderiaceae	Ralstonia	Ralstonia pickettii	5325729	5092
644736400	Ralstonia pickettii 12D	Proteobacteria	Betaproteobacteria	Burkholderiales	Burkholderiaceae	Ralstonia	Ralstonia pickettii	5685358	5518
2554235441	Ralstonia pickettii DTP0602	Proteobacteria	Betaproteobacteria	Burkholderiales	Burkholderiaceae	Ralstonia	Ralstonia pickettii	8125850	7587
637000231	Ralstonia solanacearum GMI1000	Proteobacteria	Betaproteobacteria	Burkholderiales	Burkholderiaceae	Ralstonia	Ralstonia solanacearum	5810922	5204
651053062	Ralstonia solanacearum Po82	Proteobacteria	Betaproteobacteria	Burkholderiales	Burkholderiaceae	Ralstonia	Ralstonia solanacearum	5430263	5080
2518285588	Ralstonia solanacearum FQY_4	Proteobacteria	Betaproteobacteria	Burkholderiales	Burkholderiaceae	Ralstonia	Ralstonia solanacearum	5805250	5152
2561511242	Ralstonia solanacearum CMR15	Proteobacteria	Betaproteobacteria	Burkholderiales	Burkholderiaceae	Ralstonia	Ralstonia solanacearum	5590372	5232
2561511243	Ralstonia solanacearum CFBP2957	Proteobacteria	Betaproteobacteria	Burkholderiales	Burkholderiaceae	Ralstonia	Ralstonia solanacearum	5580762	5231
2597489963	Ralstonia solanacearum Rs-09-161	Proteobacteria	Betaproteobacteria	Burkholderiales	Burkholderiaceae	Ralstonia	Ralstonia solanacearum	5645372	5013
2597490080	Ralstonia solanacearum Rs-10-244	Proteobacteria	Betaproteobacteria	Burkholderiales	Burkholderiaceae	Ralstonia	Ralstonia solanacearum	5659594	5071
2623620877	Ralstonia solanacearum GMI1000 (re-annotation)	Proteobacteria	Betaproteobacteria	Burkholderiales	Burkholderiaceae	Ralstonia	Ralstonia solanacearum	5810922	5141
650716004	Acidovorax avenae avenae ATCC 19860	Proteobacteria	Betaproteobacteria	Burkholderiales	Comamonadaceae	Acidovorax	Acidovorax avenae	5482170	4850
639633002	Acidovorax avenae citrulli AAC00-1	Proteobacteria	Betaproteobacteria	Burkholderiales	Comamonadaceae	Acidovorax	Acidovorax citrulli	5352772	4868
643348541	Acidovorax ebreus TPSY	Proteobacteria	Betaproteobacteria	Burkholderiales	Comamonadaceae	Acidovorax	Acidovorax ebreus	3796573	3606
639633003	Acidovorax sp. JS42	Proteobacteria	Betaproteobacteria	Burkholderiales	Comamonadaceae	Acidovorax	Acidovorax sp. JS42	4585154	4395
2519899535	Acidovorax sp. KKS102	Proteobacteria	Betaproteobacteria	Burkholderiales	Comamonadaceae	Acidovorax	Acidovorax sp. KKS102	5196935	4803
649633004	Alicycliphilus denitrificans BC	Proteobacteria	Betaproteobacteria	Burkholderiales	Comamonadaceae	Alicycliphilus	Alicycliphilus denitrificans	4835713	4708
650716008	Alicycliphilus denitrificans K601	Proteobacteria	Betaproteobacteria	Burkholderiales	Comamonadaceae	Alicycliphilus	Alicycliphilus denitrificans	5070751	4898
2561511126	Comamonas testosteroni sv. Ba CNB-1	Proteobacteria	Betaproteobacteria	Burkholderiales	Comamonadaceae	Comamonas	Comamonas testosteroni	5464824	4985
2597490204	Comamonas testosteroni TK102	Proteobacteria	Betaproteobacteria	Burkholderiales	Comamonadaceae	Comamonas	Comamonas testosteroni	6062703	5575
641228489	Delftia acidovorans SPH-1	Proteobacteria	Betaproteobacteria	Burkholderiales	Comamonadaceae	Delftia	Delftia acidovorans	6767514	6146
650716032	Delftia sp. Cs1-4	Proteobacteria	Betaproteobacteria	Burkholderiales	Comamonadaceae	Delftia	Delftia sp. Cs1-4	6685842	6028
639633051	Polaromonas naphthalenivorans CJ2	Proteobacteria	Betaproteobacteria	Burkholderiales	Comamonadaceae	Polaromonas	Polaromonas naphthalenivorans	5366143	5000
637000208	Polaromonas sp. JS666	Proteobacteria	Betaproteobacteria	Burkholderiales	Comamonadaceae	Polaromonas	Polaromonas sp. JS666	5898676	5634
650716081	Ramlibacter tataouinensis TTB310	Proteobacteria	Betaproteobacteria	Burkholderiales	Comamonadaceae	Ramlibacter	Ramlibacter tataouinensis	4070193	3926
637000235	Rhodoferax ferrireducens T118	Proteobacteria	Betaproteobacteria	Burkholderiales	Comamonadaceae	Rhodoferax	Rhodoferax ferrireducens	4969784	4561
644736413	Variovorax paradoxus S110	Proteobacteria	Betaproteobacteria	Burkholderiales	Comamonadaceae	Variovorax	Variovorax paradoxus	6754997	6450
649633106	Variovorax paradoxus EPS	Proteobacteria	Betaproteobacteria	Burkholderiales	Comamonadaceae	Variovorax	Variovorax paradoxus	6550056	6086
2528311013	Variovorax paradoxus B4	Proteobacteria	Betaproteobacteria	Burkholderiales	Comamonadaceae	Variovorax	Variovorax paradoxus	7148516	6844
639633065	Verminephrobacter eiseniae EF01-2	Proteobacteria	Betaproteobacteria	Burkholderiales	Comamonadaceae	Verminephrobacter	Verminephrobacter eiseniae	5597943	5115
650716028	Collimonas fungivorans Ter331	Proteobacteria	Betaproteobacteria	Burkholderiales	Oxalobacteraceae	Collimonas	Collimonas fungivorans	5186898	4493
648028033	Herbaspirillum seropedicae SmR1	Proteobacteria	Betaproteobacteria	Burkholderiales	Oxalobacteraceae	Herbaspirillum	Herbaspirillum seropedicae	5513887	4799
640069313	Herminiimonas arsenicoxydans ULPAs1	Proteobacteria	Betaproteobacteria	Burkholderiales	Oxalobacteraceae	Herminiimonas	Herminiimonas arsenicoxydans	3424307	3399

2623620298	Herminiimonas arsenicoxydans ULPAs1 (re-annotation)	Proteobacteria	Betaproteobacteria	Burkholderiales	Oxalobacteraceae	Herminiimonas	Herminiimonas arsenicoxydans	3424307	3325
2585427668	Janthinobacterium agaricidamnosum W1R3	Proteobacteria	Betaproteobacteria	Burkholderiales	Oxalobacteraceae	Janthinobacterium	Janthinobacterium agaricidamnosum	5949001	5573
640753030	Janthinobacterium sp. Marseille	Proteobacteria	Betaproteobacteria	Burkholderiales	Oxalobacteraceae	Janthinobacterium	Janthinobacterium sp. Marseille	4110251	3763
2623620337	Janthinobacterium sp. Marseille (re-annotation)	Proteobacteria	Betaproteobacteria	Burkholderiales	Oxalobacteraceae	Janthinobacterium	Janthinobacterium sp. Marseille	4110251	3902
641522635	Leptothrix cholodnii SP-6	Proteobacteria	Betaproteobacteria	Burkholderiales	unclassified	Leptothrix	Leptothrix cholodnii	4909403	4420
640069319	Methylibium petroleiphilum PM1	Proteobacteria	Betaproteobacteria	Burkholderiales	unclassified	Methylibium	Methylibium petroleiphilum	4643639	4601
2513237199	Rubrivivax gelatinosus IL144	Proteobacteria	Betaproteobacteria	Burkholderiales	unclassified	Rubrivivax	Rubrivivax gelatinosus	5043253	4766
651053077	Thiomonas arsenitoxydans 3As	Proteobacteria	Betaproteobacteria	Burkholderiales	unclassified	Thiomonas	Thiomonas arsenitoxydans	3785534	3753
646564585	Thiomonas intermedia K12	Proteobacteria	Betaproteobacteria	Burkholderiales	unclassified	Thiomonas	Thiomonas intermedia	3462095	3321
637000324	Thiobacillus denitrificans ATCC 25259	Proteobacteria	Betaproteobacteria	Hydrogenophilales	Hydrogenophilaceae	Thiobacillus	Thiobacillus denitrificans	2909809	2886
637000165	Methylobacillus flagellatus KT	Proteobacteria	Betaproteobacteria	Methylophilales	Methylophilaceae	Methylobacillus	Methylobacillus flagellatus	2971517	2824
644736388	Methylotenera mobilis JLW8	Proteobacteria	Betaproteobacteria	Methylophilales	Methylophilaceae	Methylotenera	Methylotenera mobilis	2547570	2400
646564551	Methylotenera versatilis 301, JCM 17579	Proteobacteria	Betaproteobacteria	Methylophilales	Methylophilaceae	Methylotenera	Methylotenera versatilis	3059871	2858
644736389	Methylovorus glucosetrophus SIP3-4	Proteobacteria	Betaproteobacteria	Methylophilales	Methylophilaceae	Methylovorus	Methylovorus glucosotrophus	3082007	2976
649633068	Methylovorus sp. MP688	Proteobacteria	Betaproteobacteria	Methylophilales	Methylophilaceae	Methylovorus	Methylovorus sp. MP688	2862391	2765
637000074	Chromobacterium violaceum ATCC 12472	Proteobacteria	Betaproteobacteria	Neisseriales	Chromobacteriaceae	Chromobacterium	Chromobacterium violaceum	4751080	4550
2623620687	Chromobacterium violaceum ATCC 12472 (re-annotation)	Proteobacteria	Betaproteobacteria	Neisseriales	Chromobacteriaceae	Chromobacterium	Chromobacterium violaceum	4751080	4424
643692026	Laribacter hongkongensis HLHK9	Proteobacteria	Betaproteobacteria	Neisseriales	Chromobacteriaceae	Laribacter	Laribacter hongkongensis	3169329	3333
2511231094	Pseudogulbenkiania sp. NH8B	Proteobacteria	Betaproteobacteria	Neisseriales	Chromobacteriaceae	Pseudogulbenkiania	Pseudogulbenkiania sp. NH8B	4332995	4124
637000188	Neisseria gonorrhoeae FA 1090	Proteobacteria	Betaproteobacteria	Neisseriales	Neisseriaceae	Neisseria	Neisseria gonorrhoeae	2153922	2081
642555143	Neisseria gonorrhoeae NCCP11945	Proteobacteria	Betaproteobacteria	Neisseriales	Neisseriaceae	Neisseria	Neisseria gonorrhoeae	2236178	2741
651053047	Neisseria gonorrhoeae TCDC-NG08107	Proteobacteria	Betaproteobacteria	Neisseriales	Neisseriaceae	Neisseria	Neisseria gonorrhoeae	2193889	2254
2623620787	Neisseria gonorrhoeae FA 1090 (re-annotation)	Proteobacteria	Betaproteobacteria	Neisseriales	Neisseriaceae	Neisseria	Neisseria gonorrhoeae	2153922	2206
649633075	Neisseria lactamica 020-06	Proteobacteria	Betaproteobacteria	Neisseriales	Neisseriaceae	Neisseria	Neisseria lactamica	2220606	2049
637000189	Neisseria meningitidis sv. B MC58	Proteobacteria	Betaproteobacteria	Neisseriales	Neisseriaceae	Neisseria	Neisseria meningitidis	2272360	2226
637000190	Neisseria meningitidis sv. A Z2491	Proteobacteria	Betaproteobacteria	Neisseriales	Neisseriaceae	Neisseria	Neisseria meningitidis	2184406	2227
639633045	Neisseria meningitidis sv. C FAM18	Proteobacteria	Betaproteobacteria	Neisseriales	Neisseriaceae	Neisseria	Neisseria meningitidis	2194961	2051
641228498	Neisseria meningitidis sv. C 053442	Proteobacteria	Betaproteobacteria	Neisseriales	Neisseriaceae	Neisseria	Neisseria meningitidis	2153416	2116
644736394	Neisseria meningitidis sv. Cnl ST-53 a-14	Proteobacteria	Betaproteobacteria	Neisseriales	Neisseriaceae	Neisseria	Neisseria meningitidis	2145295	1943
646862337	Neisseria meningitidis 8013	Proteobacteria	Betaproteobacteria	Neisseriales	Neisseriaceae	Neisseria	Neisseria meningitidis	2277550	2206
650377962	Neisseria meningitidis sv. B alpha710	Proteobacteria	Betaproteobacteria	Neisseriales	Neisseriaceae	Neisseria	Neisseria meningitidis	2242947	2132
651053048	Neisseria meningitidis G2136	Proteobacteria	Betaproteobacteria	Neisseriales	Neisseriaceae	Neisseria	Neisseria meningitidis	2184862	2042
651053049	Neisseria meningitidis H44/76	Proteobacteria	Betaproteobacteria	Neisseriales	Neisseriaceae	Neisseria	Neisseria meningitidis	2240883	2096
651053050	Neisseria meningitidis M01-240149	Proteobacteria	Betaproteobacteria	Neisseriales	Neisseriaceae	Neisseria	Neisseria meningitidis	2223518	2056
651053051	Neisseria meningitidis M01-240355	Proteobacteria	Betaproteobacteria	Neisseriales	Neisseriaceae	Neisseria	Neisseria meningitidis	2287777	2094
651053052	Neisseria meningitidis M04-240196	Proteobacteria	Betaproteobacteria	Neisseriales	Neisseriaceae	Neisseria	Neisseria meningitidis	2250449	2093
651053053	Neisseria meningitidis NZ-05/33	Proteobacteria	Betaproteobacteria	Neisseriales	Neisseriaceae	Neisseria	Neisseria meningitidis	2248966	2085
651053054	Neisseria meningitidis sv.A WUE 2594	Proteobacteria	Betaproteobacteria	Neisseriales	Neisseriaceae	Neisseria	Neisseria meningitidis	2227255	2137
2576861469	Neisseria meningitidis NMA510612	Proteobacteria	Betaproteobacteria	Neisseriales	Neisseriaceae	Neisseria	Neisseria meningitidis	2188020	2532
2623620291	Neisseria meningitidis sv. C FAM18 (re-annotation)	Proteobacteria	Betaproteobacteria	Neisseriales	Neisseriaceae	Neisseria	Neisseria meningitidis	2194961	2119
2623620725	Neisseria meningitidis sv. B MC58 (re-annotation)	Proteobacteria	Betaproteobacteria	Neisseriales	Neisseriaceae	Neisseria	Neisseria meningitidis	2272360	2176
2623620954	Neisseria meningitidis sv. A Z2491 (re-annotation)	Proteobacteria	Betaproteobacteria	Neisseriales	Neisseriaceae	Neisseria	Neisseria meningitidis	2184406	2129
2585428136	Snodgrassella alvi wkB2	Proteobacteria	Betaproteobacteria	Neisseriales	Neisseriaceae	Snodgrassella	Snodgrassella alvi	2527978	2370
637000195	Nitrosomonas europaea ATCC 19718	Proteobacteria	Betaproteobacteria	Nitrosomonadales	Nitrosomonadaceae	Nitrosomonas	Nitrosomonas europaea	2812094	2630
637000196	Nitrosomonas eutropha C91	Proteobacteria	Betaproteobacteria	Nitrosomonadales	Nitrosomonadaceae	Nitrosomonas	Nitrosomonas eutropha	2781824	2695
650716066	Nitrosomonas sp. AL212	Proteobacteria	Betaproteobacteria	Nitrosomonadales	Nitrosomonadaceae	Nitrosomonas	Nitrosomonas sp. AL212	3337023	3238
650716067	Nitrosomonas sp. IS79A3	Proteobacteria	Betaproteobacteria	Nitrosomonadales	Nitrosomonadaceae	Nitrosomonas	Nitrosomonas sp. Is79A3	3783444	3597
637000197	Nitrosospira multiformis ATCC 25196	Proteobacteria	Betaproteobacteria	Nitrosomonadales	Nitrosomonadaceae	Nitrosospira	Nitrosospira multiformis	3234309	2885
637000012	Aromatoleum aromaticum EbN1	Proteobacteria	Betaproteobacteria	Rhodocyclales	Rhodocyclaceae	Aromatoleum	Aromatoleum aromaticum	4727255	4686
2623620890	Aromatoleum aromaticum EbN1 (re-annotation)	Proteobacteria	Betaproteobacteria	Rhodocyclales	Rhodocyclaceae	Aromatoleum	Aromatoleum aromaticum	4727255	4590
639633007	Azoarcus sp. BH72	Proteobacteria	Betaproteobacteria	Rhodocyclales	Rhodocyclaceae	Azoarcus	Azoarcus sp. BH72	4376040	4073
2623620205	Azoarcus sp. BH72 (re-annotation)	Proteobacteria	Betaproteobacteria	Rhodocyclales	Rhodocyclaceae	Azoarcus	Azoarcus sp. BH72	4376040	4040
2563366569	Azoarcus sp. KH32C	Proteobacteria	Betaproteobacteria	Rhodocyclales	Rhodocyclaceae	Azoarcus	Azoarcus sp. KH32C	5818755	5267
2508501046	Azospira suillum PS	Proteobacteria	Betaproteobacteria	Rhodocyclales	Rhodocyclaceae	Azospira	Azospira oryzae	3806980	3539
637000088	Dechloromonas aromatica RCB	Proteobacteria	Betaproteobacteria	Rhodocyclales	Rhodocyclaceae	Dechloromonas	Dechloromonas aromatica	4501104	4302

643692051	Thauera aminoaromatica MZ1T	Proteobacteria	Betaproteobacteria	Rhodocyclales	Rhodocyclaceae	Thauera	Thauera sp. MZ1T	4574586	4142
2593339110	Rhodocyclaceae bacterium PG1-Ca6	Proteobacteria	Betaproteobacteria	Rhodocyclales	unclassified	unclassified	unclassified	2934611	2902
2531839478	Sulfuricella denitrificans skB26	Proteobacteria	Betaproteobacteria	Sulfuricellales	Sulfuricellaceae	Sulfuricella	Sulfuricella denitrificans	3238714	3179
644736333	Candidatus Accumulibacter phosphatis Type IIA UW-1	Proteobacteria	Betaproteobacteria	unclassified	unclassified	Candidatus Accumulibacter	Candidatus Accumulibacter phosphatis	5306133	4790
2556921087	Candidatus Accumulibacter sp. BA-91 Ga0063445	Proteobacteria	Betaproteobacteria	unclassified	unclassified	Candidatus Accumulibacter	Candidatus Accumulibacter sp. BA-91	4535688	5067
2556921088	Candidatus Accumulibacter sp. BA-92 Ga0063446	Proteobacteria	Betaproteobacteria	unclassified	unclassified	Candidatus Accumulibacter	Candidatus Accumulibacter sp. BA-92	4947936	4632
2556921089	Candidatus Accumulibacter sp. BA-93 Ga0063447	Proteobacteria	Betaproteobacteria	unclassified	unclassified	Candidatus Accumulibacter	Candidatus Accumulibacter sp. BA-93	4626262	4179
2556921083	Candidatus Accumulibacter sp. SK-01 Ga0063440	Proteobacteria	Betaproteobacteria	unclassified	unclassified	Candidatus Accumulibacter	Candidatus Accumulibacter sp. SK-01	4904783	5182
2556921084	Candidatus Accumulibacter sp. BA-91 Ga0063441	Proteobacteria	Betaproteobacteria	unclassified	unclassified	Candidatus Accumulibacter	Candidatus Accumulibacter sp. SK-02	4810930	4492
2556921085	Candidatus Accumulibacter sp. SK-11 Ga0063442	Proteobacteria	Betaproteobacteria	unclassified	unclassified	Candidatus Accumulibacter	Candidatus Accumulibacter sp. SK-11	4700180	4658
2556921086	Synthetic wastewater microbial communities from Lab-scale EBPR Ga0063443	Proteobacteria	Betaproteobacteria	unclassified	unclassified	Candidatus Accumulibacter	Candidatus Accumulibacter sp. SK-12	4412715	4029
2563366624	Candidatus Kinetoplastibacterium crithidii (ex Angomonas deanei ATCC 30255)	Proteobacteria	Betaproteobacteria	unclassified	unclassified	Kinetoplastibacterium	Candidatus Kinetoplastibacterium crithidii	821813	787
2563366627	Candidatus Kinetoplastibacterium crithidii TCC036E	Proteobacteria	Betaproteobacteria	unclassified	unclassified	Kinetoplastibacterium	Candidatus Kinetoplastibacterium crithidii	821930	785
2563366576	Candidatus Kinetoplastibacterium galatii TCC219	Proteobacteria	Betaproteobacteria	unclassified	unclassified	Kinetoplastibacterium	Candidatus Kinetoplastibacterium galatii	822140	783
2565956558	beta proteobacterium CB	Proteobacteria	Betaproteobacteria	unclassified	unclassified	unclassified	beta proteobacterium CB	2045720	2152
649633096	Stigmatella aurantiaca DW4/3-1	Proteobacteria	Deltaproteobacteria	Myxococcales	Cystobacteraceae	Stigmatella	Stigmatella aurantiaca	10260756	8407
646311933	Haliangium ochraceum SMP-2, DSM 14365	Proteobacteria	Deltaproteobacteria	Myxococcales	Kofleriaceae	Haliangium	Haliangium ochraceum	9446314	6952
2512564078	Corallococcus coralloides DSM 2259	Proteobacteria	Deltaproteobacteria	Myxococcales	Myxococcaceae	Corallococcus	Corallococcus coralloides	10080619	8101
650716065	Myxococcus fulvus HW-1	Proteobacteria	Deltaproteobacteria	Myxococcales	Myxococcaceae	Myxococcus	Myxococcus fulvus	9003593	7362
2521172697	Myxococcus stipitatus DSM 14675	Proteobacteria	Deltaproteobacteria	Myxococcales	Myxococcaceae	Myxococcus	Myxococcus stipitatus	10350586	8128
637000186	Myxococcus xanthus DK 1622	Proteobacteria	Deltaproteobacteria	Myxococcales	Myxococcaceae	Myxococcus	Myxococcus xanthus	9139763	7454
2623620856	Myxococcus xanthus DK 1622 (re-annotation)	Proteobacteria	Deltaproteobacteria	Myxococcales	Myxococcaceae	Myxococcus	Myxococcus xanthus	9139763	7408
641228509	Sorangium cellulosum So ce 56	Proteobacteria	Deltaproteobacteria	Myxococcales	Polyangiaceae	Sorangium	Sorangium cellulosum	13033779	9700
2563366597	Sorangium cellulosum So0157-2	Proteobacteria	Deltaproteobacteria	Myxococcales	Polyangiaceae	Sorangium	Sorangium cellulosum	14782125	10503
650716003	Acidithiobacillus caldus SM-1	Proteobacteria	Gammaproteobacteria	Acidithiobacillales	Acidithiobacillaceae	Acidithiobacillus	Acidithiobacillus caldus	3237599	3239
2510436001	Acidithiobacillus ferrivorans SS3	Proteobacteria	Gammaproteobacteria	Acidithiobacillales	Acidithiobacillaceae	Acidithiobacillus	Acidithiobacillus ferrivorans	3207552	3335
642555101	Acidithiobacillus ferrooxidans ATCC 53993	Proteobacteria	Gammaproteobacteria	Acidithiobacillales	Acidithiobacillaceae	Acidithiobacillus	Acidithiobacillus ferrooxidans	2885038	2951
643348501	Acidithiobacillus ferrooxidans ATCC 23270	Proteobacteria	Gammaproteobacteria	Acidithiobacillales	Acidithiobacillaceae	Acidithiobacillus	Acidithiobacillus ferrooxidans	2982397	3234
639633004	Aeromonas hydrophila hydrophila ATCC 7966	Proteobacteria	Gammaproteobacteria	Aeromonadales	Aeromonadaceae	Aeromonas	Aeromonas hydrophila	4744448	4321
2541047079	Aeromonas hydrophila ML09-119	Proteobacteria	Gammaproteobacteria	Aeromonadales	Aeromonadaceae	Aeromonas	Aeromonas hydrophila	5024500	4577
2563366591	Aeromonas hydrophila 4AK4	Proteobacteria	Gammaproteobacteria	Aeromonadales	Aeromonadaceae	Aeromonas	Aeromonas hydrophila	4527993	4272
2576861445	Aeromonas hydrophila pc104A	Proteobacteria	Gammaproteobacteria	Aeromonadales	Aeromonadaceae	Aeromonas	Aeromonas hydrophila	5023829	4493
2576861447	Aeromonas hydrophila AL09-71	Proteobacteria	Gammaproteobacteria	Aeromonadales	Aeromonadaceae	Aeromonas	Aeromonas hydrophila	5023861	4492
2576861457	Aeromonas hydrophila YL17	Proteobacteria	Gammaproteobacteria	Aeromonadales	Aeromonadaceae	Aeromonas	Aeromonas hydrophila	4806266	4286
2623620201	Aeromonas hydrophila hydrophila ATCC 7966 (re-annotation)	Proteobacteria	Gammaproteobacteria	Aeromonadales	Aeromonadaceae	Aeromonas	Aeromonas hydrophila	4744448	4379
641522605	Aeromonas salmonicida salmonicida A449	Proteobacteria	Gammaproteobacteria	Aeromonadales	Aeromonadaceae	Aeromonas	Aeromonas salmonicida	5040536	4608
650716006	Aeromonas veronii B565	Proteobacteria	Gammaproteobacteria	Aeromonadales	Aeromonadaceae	Aeromonas	Aeromonas veronii	4551783	4161
2512047035	Oceanimonas sp. GK1	Proteobacteria	Gammaproteobacteria	Aeromonadales	Aeromonadaceae	Oceanimonas	Oceanimonas sp. GK1	3527244	3333
643692052	Tolumonas auensis TA 4, DSM 9187	Proteobacteria	Gammaproteobacteria	Aeromonadales	Aeromonadaceae	Tolumonas	Tolumonas auensis	3471292	3288
2585427644	Alteromonas australica H17	Proteobacteria	Gammaproteobacteria	Alteromonadales	Alteromonadaceae	Alteromonas	Alteromonas australica	4308833	3534
642555105	Alteromonas macleodii Deep ecotype, DSM 17117	Proteobacteria	Gammaproteobacteria	Alteromonadales	Alteromonadaceae	Alteromonas	Alteromonas macleodii	4412282	4128
2521172683	Alteromonas macleodii English Channel 673	Proteobacteria	Gammaproteobacteria	Alteromonadales	Alteromonadaceae	Alteromonas	Alteromonas macleodii	4601785	3979
2521172684	Alteromonas macleodii Black Sea 11	Proteobacteria	Gammaproteobacteria	Alteromonadales	Alteromonadaceae	Alteromonas	Alteromonas macleodii	4480507	3858
2521172685	Alteromonas macleodii AltDE1	Proteobacteria	Gammaproteobacteria	Alteromonadales	Alteromonadaceae	Alteromonas	Alteromonas macleodii	4947126	4405
2521172706	Alteromonas macleodii Balearic Sea AD45	Proteobacteria	Gammaproteobacteria	Alteromonadales	Alteromonadaceae	Alteromonas	Alteromonas macleodii	4674885	4054
2545824627	Alteromonas macleodii 'Aegean Sea MED64'	Proteobacteria	Gammaproteobacteria	Alteromonadales	Alteromonadaceae	Alteromonas	Alteromonas macleodii	4397537	3898
2554235399	Alteromonas macleodii 'Ionian Sea U7'	Proteobacteria	Gammaproteobacteria	Alteromonadales	Alteromonadaceae	Alteromonas	Alteromonas macleodii	4442936	3931
2554235400	Alteromonas macleodii 'Ionian Sea U8'	Proteobacteria	Gammaproteobacteria	Alteromonadales	Alteromonadaceae	Alteromonas	Alteromonas macleodii	4395035	3873
2554235401	Alteromonas macleodii 'Ionian Sea UM4b'	Proteobacteria	Gammaproteobacteria	Alteromonadales	Alteromonadaceae	Alteromonas	Alteromonas macleodii	4438767	3942
2554235747	Alteromonas macleodii ATCC 27126	Proteobacteria	Gammaproteobacteria	Alteromonadales	Alteromonadaceae	Alteromonas	Alteromonas macleodii	4653851	3941
2561511203	Alteromonas macleodii 'Ionian Sea U4'	Proteobacteria	Gammaproteobacteria	Alteromonadales	Alteromonadaceae	Alteromonas	Alteromonas macleodii	4624578	4160
2561511204	Alteromonas macleodii 'Ionian Sea UM7'	Proteobacteria	Gammaproteobacteria	Alteromonadales	Alteromonadaceae	Alteromonas	Alteromonas macleodii	4931660	4410
2561511205	Alteromonas macleodii 'English Channel 615'	Proteobacteria	Gammaproteobacteria	Alteromonadales	Alteromonadaceae	Alteromonas	Alteromonas macleodii	4582352	3644
2504136012	Alteromonas sp. SN2	Proteobacteria	Gammaproteobacteria	Alteromonadales	Alteromonadaceae	Alteromonas	Alteromonas sp. SN2	4972148	4442
2511231080	Glaciecola nitratireducens FR1064	Proteobacteria	Gammaproteobacteria	Alteromonadales	Alteromonadaceae	Glaciecola	Glaciecola nitratireducens	4134229	3720

2545824640	Glaciecola psychrophila 170	Proteobacteria	Gammaproteobacteria	Alteromonadales	Alteromonadaceae	Glaciecola	Glaciecola psychrophila	5413691	5695
650716040	Glaciecola agarilytica 4H-3-7+YE-5	Proteobacteria	Gammaproteobacteria	Alteromonadales	Alteromonadaceae	Glaciecola	Glaciecola sp. 4H-3-7+YE-5	5393591	4688
2513237134	Glaciecola sp. ANT9081 (cola82)	Proteobacteria	Gammaproteobacteria	Alteromonadales	Alteromonadaceae	Glaciecola	Glaciecola sp. ANT9081	4996068	4330
650377991	Marinobacter adhaerens HP15	Proteobacteria	Gammaproteobacteria	Alteromonadales	Alteromonadaceae	Marinobacter	Marinobacter adhaerens	4651725	4470
639633037	Marinobacter aquaeolei VT8	Proteobacteria	Gammaproteobacteria	Alteromonadales	Alteromonadaceae	Marinobacter	Marinobacter hydrocarbonoclasticus	4779762	4342
2540341173	Marinobacter hydrocarbonoclasticus ATCC 49840	Proteobacteria	Gammaproteobacteria	Alteromonadales	Alteromonadaceae	Marinobacter	Marinobacter hydrocarbonoclasticus	3989480	3863
2518645575	Marinobacter sp. BSs20148	Proteobacteria	Gammaproteobacteria	Alteromonadales	Alteromonadaceae	Marinobacter	Marinobacter sp. BSs20148	4063864	3944
637000249	Saccharophagus degradans 2-40	Proteobacteria	Gammaproteobacteria	Alteromonadales	Alteromonadaceae	Saccharophagus	Saccharophagus degradans	5057531	4114
637000081	Colwellia psychrerythraea 34H	Proteobacteria	Gammaproteobacteria	Alteromonadales	Colwelliaceae	Colwellia	Colwellia psychrerythraea	5373180	5066
2623620821	Colwellia psychrerythraea 34H (re-annotation)	Proteobacteria	Gammaproteobacteria	Alteromonadales	Colwelliaceae	Colwellia	Colwellia psychrerythraea	5373180	4637
648028026	Ferrimonas balearica PAT, DSM 9799	Proteobacteria	Gammaproteobacteria	Alteromonadales	Ferrimonadaceae	Ferrimonas	Ferrimonas balearica	4279159	3947
637000136	Idiomarina loihiensis L2TR	Proteobacteria	Gammaproteobacteria	Alteromonadales	Idiomarinaceae	Idiomarina	Idiomarina loihiensis	2839318	2706
2554235415	Idiomarina loihiensis GSL 199	Proteobacteria	Gammaproteobacteria	Alteromonadales	Idiomarinaceae	Idiomarina	Idiomarina loihiensis	2839759	2717
2623620929	Idiomarina loihiensis L2TR (re-annotation)	Proteobacteria	Gammaproteobacteria	Alteromonadales	Idiomarinaceae	Idiomarina	Idiomarina loihiensis	2839318	2732
637000216	Pseudoalteromonas atlantica T6c	Proteobacteria	Gammaproteobacteria	Alteromonadales	Pseudoalteromonadaceae	Pseudoalteromonas	Pseudoalteromonas atlantica	5187005	4405
637000217	Pseudoalteromonas haloplanktis TAC125	Proteobacteria	Gammaproteobacteria	Alteromonadales	Pseudoalteromonadaceae	Pseudoalteromonas	Pseudoalteromonas haloplanktis	3850272	3634
2623621007	Pseudoalteromonas haloplanktis TAC125 (re-annotation)	Proteobacteria	Gammaproteobacteria	Alteromonadales	Pseudoalteromonadaceae	Pseudoalteromonas	Pseudoalteromonas haloplanktis	3850272	3548
649633085	Pseudoalteromonas sp. SM9913	Proteobacteria	Gammaproteobacteria	Alteromonadales	Pseudoalteromonadaceae	Pseudoalteromonas	Pseudoalteromonas sp. SM9913	4037671	3799
639633052	Psychromonas ingrahamii 37	Proteobacteria	Gammaproteobacteria	Alteromonadales	Psychromonadaceae	Psychromonas	Psychromonas ingrahamii	4559598	3877
2501651206	Psychromonas sp. CNPT3	Proteobacteria	Gammaproteobacteria	Alteromonadales	Psychromonadaceae	Psychromonas	Psychromonas sp. CNPT3	3053233	2854
2518645527	Shewanella amazonensis SB2B	Proteobacteria	Gammaproteobacteria	Alteromonadales	Shewanellaceae	Shewanella	Shewanella amazonensis	4306142	3803
640069330	Shewanella baltica OS155	Proteobacteria	Gammaproteobacteria	Alteromonadales	Shewanellaceae	Shewanella	Shewanella baltica	5342896	4741
640753049	Shewanella baltica OS185	Proteobacteria	Gammaproteobacteria	Alteromonadales	Shewanellaceae	Shewanella	Shewanella baltica	5312910	4618
641228507	Shewanella baltica OS195	Proteobacteria	Gammaproteobacteria	Alteromonadales	Shewanellaceae	Shewanella	Shewanella baltica	5547544	4857
643348574	Shewanella baltica OS223	Proteobacteria	Gammaproteobacteria	Alteromonadales	Shewanellaceae	Shewanella	Shewanella baltica	5358884	4622
648276724	Shewanella baltica BA175	Proteobacteria	Gammaproteobacteria	Alteromonadales	Shewanellaceae	Shewanella	Shewanella baltica	5198909	4609
651053066	Shewanella baltica OS117	Proteobacteria	Gammaproteobacteria	Alteromonadales	Shewanellaceae	Shewanella	Shewanella baltica	5526018	4985
2524023135	Shewanella denitrificans OS217	Proteobacteria	Gammaproteobacteria	Alteromonadales	Shewanellaceae	Shewanella	Shewanella denitrificans	4545906	3920
2524023134	Shewanella frigidimarina NCIMB 400	Proteobacteria	Gammaproteobacteria	Alteromonadales	Shewanellaceae	Shewanella	Shewanella frigidimarina	4845257	4174
641522648	Shewanella halifaxensis HAW-EB4	Proteobacteria	Gammaproteobacteria	Alteromonadales	Shewanellaceae	Shewanella	Shewanella halifaxensis	5226917	4462
2521172611	Shewanella loihica PV-4	Proteobacteria	Gammaproteobacteria	Alteromonadales	Shewanellaceae	Shewanella	Shewanella loihica	4602594	4043
637000258	*Shewanella oneidensis* MR-1	Proteobacteria	Gammaproteobacteria	Alteromonadales	Shewanellaceae	Shewanella	*Shewanella oneidensis*	5131416	4657
2623620650	*Shewanella oneidensis* MR-1 (re-annotation)	Proteobacteria	Gammaproteobacteria	Alteromonadales	Shewanellaceae	Shewanella	*Shewanella oneidensis*	5131424	4746
641228508	Shewanella pealeana ANG-SQ1, ATCC 700345	Proteobacteria	Gammaproteobacteria	Alteromonadales	Shewanellaceae	Shewanella	Shewanella pealeana	5174581	4434
643348575	Shewanella piezotolerans WP3	Proteobacteria	Gammaproteobacteria	Alteromonadales	Shewanellaceae	Shewanella	Shewanella piezotolerans	5396476	5047
2510436004	Shewanella putrefaciens 200 (Missing data)	Proteobacteria	Gammaproteobacteria	Alteromonadales	Shewanellaceae	Shewanella	Shewanella putrefaciens	4840251	4457
2524023073	Shewanella putrefaciens CN-32	Proteobacteria	Gammaproteobacteria	Alteromonadales	Shewanellaceae	Shewanella	Shewanella putrefaciens	4659220	4083
640753050	Shewanella sediminis HAW-EB3	Proteobacteria	Gammaproteobacteria	Alteromonadales	Shewanellaceae	Shewanella	Shewanella sediminis	5517674	4666
639633058	Shewanella sp. ANA-3	Proteobacteria	Gammaproteobacteria	Alteromonadales	Shewanellaceae	Shewanella	Shewanella sp. ANA-3	5251146	4537
637000259	Shewanella sp. MR-4	Proteobacteria	Gammaproteobacteria	Alteromonadales	Shewanellaceae	Shewanella	Shewanella sp. MR-4	4706287	4098
637000260	Shewanella sp. MR-7	Proteobacteria	Gammaproteobacteria	Alteromonadales	Shewanellaceae	Shewanella	Shewanella sp. MR-7	4799109	4172
639633059	Shewanella sp. W3-18-1	Proteobacteria	Gammaproteobacteria	Alteromonadales	Shewanellaceae	Shewanella	Shewanella sp. W3-18-1	4708380	4237
646564568	Shewanella violacea DSS12	Proteobacteria	Gammaproteobacteria	Alteromonadales	Shewanellaceae	Shewanella	Shewanella violacea	4962103	4515
641522649	Shewanella woodyi MS32, ATCC 51908	Proteobacteria	Gammaproteobacteria	Alteromonadales	Shewanellaceae	Shewanella	Shewanella woodyi	5935403	5085
644736410	Teredinibacter turnerae T7901	Proteobacteria	Gammaproteobacteria	Alteromonadales	unclassified	Teredinibacter	Teredinibacter turnerae	5193164	4308
646564502	*Allochromatium vinosum* DSM 180	Proteobacteria	Gammaproteobacteria	Chromatiales	Chromatiaceae	Allochromatium	*Allochromatium vinosum*	3669074	3366
2510065050	Marichromatium purpuratum 984	Proteobacteria	Gammaproteobacteria	Chromatiales	Chromatiaceae	Marichromatium	Marichromatium purpuratum	3779112	3339
646564556	Nitrosococcus halophilus Nc4	Proteobacteria	Gammaproteobacteria	Chromatiales	Chromatiaceae	Nitrosococcus	Nitrosococcus halophilus	4145260	4086
2508501051	Thiocystis violascens 611, DSM 198	Proteobacteria	Gammaproteobacteria	Chromatiales	Chromatiaceae	Thiocystis	Thiocystis violascens	5017071	4627
2506783059	Thioflavicoccus mobilis 8321	Proteobacteria	Gammaproteobacteria	Chromatiales	Chromatiaceae	Thioflavicoccus	Thioflavicoccus mobilis	4137521	3787
637000005	Alkalilimnicola ehrlichii MLHE-1	Proteobacteria	Gammaproteobacteria	Chromatiales	Ectothiorhodospiraceae	Alkalilimnicola	Alkalilimnicola ehrlichii	3275944	2947
2571042483	Halorhodospira halochloris A	Proteobacteria	Gammaproteobacteria	Chromatiales	Ectothiorhodospiraceae	Halorhodospira	Halorhodospira halochloris	3460134	3142
639633026	Halorhodospira halophila SL1	Proteobacteria	Gammaproteobacteria	Chromatiales	Ectothiorhodospiraceae	Halorhodospira	Halorhodospira halophila	2678452	2470
2554235389	Ectothiorhodospiraceae bacterium M19-40	Proteobacteria	Gammaproteobacteria	Chromatiales	Ectothiorhodospiraceae	Spiribacter	Spiribacter salinus	1739487	1732
2558309070	Spiribacter sp. UAH-SP71	Proteobacteria	Gammaproteobacteria	Chromatiales	Ectothiorhodospiraceae	Spiribacter	Spiribacter sp. UAH-SP71	1926631	1912

2521172692	Thioalkalivibrio nitratireducens DSM 14787	Proteobacteria	Gammaproteobacteria	Chromatiales	Ectothiorhodospiraceae	Thioalkalivibrio	Thioalkalivibrio nitratireducens	4002352	3875
2506520043	Thioalkalivibrio paradoxus ARh 1	Proteobacteria	Gammaproteobacteria	Chromatiales	Ectothiorhodospiraceae	Thioalkalivibrio	Thioalkalivibrio paradoxus	3756729	3557
646564584	Thioalkalivibrio sp. K90mix	Proteobacteria	Gammaproteobacteria	Chromatiales	Ectothiorhodospiraceae	Thioalkalivibrio	Thioalkalivibrio sp. K90mix	2985056	2942
643348585	Thioalkalivibrio sulfidophilus HL-EbGR7	Proteobacteria	Gammaproteobacteria	Chromatiales	Ectothiorhodospiraceae	Thioalkalivibrio	Thioalkalivibrio sulfidiphilus	3464554	3366
646311935	Halothiobacillus neapolitanus c2, ATCC 23641	Proteobacteria	Gammaproteobacteria	Chromatiales	Halothiobacillaceae	Halothiobacillus	Halothiobacillus neapolitanus	2582886	2465
637000043	Buchnera aphidicola APS	Proteobacteria	Gammaproteobacteria	Enterobacteriales	Enterobacteriaceae	Buchnera	Buchnera aphidicola	655725	615
637000045	Buchnera aphidicola Sg	Proteobacteria	Gammaproteobacteria	Enterobacteriales	Enterobacteriaceae	Buchnera	Buchnera aphidicola	641454	588
639633012	Buchnera aphidicola Bp	Proteobacteria	Gammaproteobacteria	Enterobacteriales	Enterobacteriaceae	Buchnera	Buchnera aphidicola	618379	557
643348520	Buchnera aphidicola 5A	Proteobacteria	Gammaproteobacteria	Enterobacteriales	Enterobacteriaceae	Buchnera	Buchnera aphidicola	642122	590
643348522	Buchnera aphidicola Tuc7	Proteobacteria	Gammaproteobacteria	Enterobacteriales	Enterobacteriaceae	Buchnera	Buchnera aphidicola	641895	588
650377915	Buchnera aphidicola JF98	Proteobacteria	Gammaproteobacteria	Enterobacteriales	Enterobacteriaceae	Buchnera	Buchnera aphidicola	641771	512
650377916	Buchnera aphidicola JF99	Proteobacteria	Gammaproteobacteria	Enterobacteriales	Enterobacteriaceae	Buchnera	Buchnera aphidicola	641716	625
650377917	Buchnera aphidicola LL01	Proteobacteria	Gammaproteobacteria	Enterobacteriales	Enterobacteriaceae	Buchnera	Buchnera aphidicola	641799	612
650377918	Buchnera aphidicola TLW03	Proteobacteria	Gammaproteobacteria	Enterobacteriales	Enterobacteriaceae	Buchnera	Buchnera aphidicola	641770	608
2511231158	Buchnera aphidicola Ua	Proteobacteria	Gammaproteobacteria	Enterobacteriales	Enterobacteriaceae	Buchnera	Buchnera aphidicola	627953	590
2511231206	Buchnera aphidicola Ak	Proteobacteria	Gammaproteobacteria	Enterobacteriales	Enterobacteriaceae	Buchnera	Buchnera aphidicola	653223	624
2558860194	Buchnera aphidicola USDA	Proteobacteria	Gammaproteobacteria	Enterobacteriales	Enterobacteriaceae	Buchnera	Buchnera aphidicola	651306	610
2558860195	Buchnera aphidicola G002	Proteobacteria	Gammaproteobacteria	Enterobacteriales	Enterobacteriaceae	Buchnera	Buchnera aphidicola	651316	607
2558860196	Buchnera aphidicola W106	Proteobacteria	Gammaproteobacteria	Enterobacteriales	Enterobacteriaceae	Buchnera	Buchnera aphidicola	651304	608
2558860197	Buchnera aphidicola F009	Proteobacteria	Gammaproteobacteria	Enterobacteriales	Enterobacteriaceae	Buchnera	Buchnera aphidicola	651310	608
2623620289	Buchnera aphidicola Bp (re-annotation)	Proteobacteria	Gammaproteobacteria	Enterobacteriales	Enterobacteriaceae	Buchnera	Buchnera aphidicola	618379	564
2623620679	Buchnera aphidicola APS (re-annotation)	Proteobacteria	Gammaproteobacteria	Enterobacteriales	Enterobacteriaceae	Buchnera	Buchnera aphidicola	655725	634
2623620906	Buchnera aphidicola Sg (re-annotation)	Proteobacteria	Gammaproteobacteria	Enterobacteriales	Enterobacteriaceae	Buchnera	Buchnera aphidicola	641454	642
644736334	Candidatus Hamiltonella defensa 5AT	Proteobacteria	Gammaproteobacteria	Enterobacteriales	Enterobacteriaceae	Candidatus Hamiltonella	Candidatus Hamiltonella defensa	2169363	2200
646564517	Candidatus Riesia pediculicola USDA	Proteobacteria	Gammaproteobacteria	Enterobacteriales	Enterobacteriaceae	Candidatus Riesia	Candidatus Riesia pediculicola	582127	595
2576861439	Citrobacter freundii CFNIH1	Proteobacteria	Gammaproteobacteria	Enterobacteriales	Enterobacteriaceae	Citrobacter	Citrobacter freundii	5371331	5144
640753015	Citrobacter koseri ATCC BAA-895	Proteobacteria	Gammaproteobacteria	Enterobacteriales	Enterobacteriaceae	Citrobacter	Citrobacter koseri	4735357	5171
2623620243	Citrobacter koseri ATCC BAA-895 (re-annotation)	Proteobacteria	Gammaproteobacteria	Enterobacteriales	Enterobacteriaceae	Citrobacter	Citrobacter koseri	4735357	4475
646311913	Citrobacter rodentium ICC168	Proteobacteria	Gammaproteobacteria	Enterobacteriales	Enterobacteriaceae	Citrobacter	Citrobacter rodentium	5444283	5078
640753022	Cronobacter sakazakii ATCC BAA-894	Proteobacteria	Gammaproteobacteria	Enterobacteriales	Enterobacteriaceae	Cronobacter	Cronobacter sakazakii	4530777	4573
2519103108	Cronobacter sakazakii ES15	Proteobacteria	Gammaproteobacteria	Enterobacteriales	Enterobacteriaceae	Cronobacter	Cronobacter sakazakii	4268675	4018
2558309087	Cronobacter sakazakii CMCC 45402	Proteobacteria	Gammaproteobacteria	Enterobacteriales	Enterobacteriaceae	Cronobacter	Cronobacter sakazakii	4559945	4417
2561511151	Cronobacter sakazakii Sp291	Proteobacteria	Gammaproteobacteria	Enterobacteriales	Enterobacteriaceae	Cronobacter	Cronobacter sakazakii	4518784	4287
2623620247	Cronobacter sakazakii ATCC BAA-894 (re-annotation)	Proteobacteria	Gammaproteobacteria	Enterobacteriales	Enterobacteriaceae	Cronobacter	Cronobacter sakazakii	4530777	4333
646311918	Cronobacter turicensis z3032	Proteobacteria	Gammaproteobacteria	Enterobacteriales	Enterobacteriaceae	Cronobacter	Cronobacter turicensis	4599155	4567
2551306348	Dickeya chrysanthemi NCPPB 516	Proteobacteria	Gammaproteobacteria	Enterobacteriales	Enterobacteriaceae	Dickeya	Dickeya chrysanthemi	4614643	4336
644736354	Dickeya dadantii Ech703	Proteobacteria	Gammaproteobacteria	Enterobacteriales	Enterobacteriaceae	Dickeya	Dickeya dadantii	4679450	4133
646311920	Dickeya dadantii Ech586	Proteobacteria	Gammaproteobacteria	Enterobacteriales	Enterobacteriaceae	Dickeya	Dickeya dadantii	4818394	4318
648028024	Dickeya dadantii 3937	Proteobacteria	Gammaproteobacteria	Enterobacteriales	Enterobacteriaceae	Dickeya	Dickeya dadantii	4922802	4664
644736355	Dickeya zeae Ech1591	Proteobacteria	Gammaproteobacteria	Enterobacteriales	Enterobacteriaceae	Dickeya	Dickeya zeae	4813854	4363
644736357	Edwardsiella ictaluri 93-146	Proteobacteria	Gammaproteobacteria	Enterobacteriales	Enterobacteriaceae	Edwardsiella	Edwardsiella ictaluri	3812315	3902
2541047080	Edwardsiella tarda C07-087	Proteobacteria	Gammaproteobacteria	Enterobacteriales	Enterobacteriaceae	Edwardsiella	Edwardsiella piscicida	3857040	3525
646311921	Edwardsiella tarda EIB202	Proteobacteria	Gammaproteobacteria	Enterobacteriales	Enterobacteriaceae	Edwardsiella	Edwardsiella tarda	3804166	3708
648231704	Edwardsiella tarda FL6-60	Proteobacteria	Gammaproteobacteria	Enterobacteriales	Enterobacteriaceae	Edwardsiella	Edwardsiella tarda	3728801	3376
650716035	Enterobacter aerogenes KCTC 2190	Proteobacteria	Gammaproteobacteria	Enterobacteriales	Enterobacteriaceae	Enterobacter	Enterobacter aerogenes	5280350	5021
2554235336	Enterobacter aerogenes EA1509E	Proteobacteria	Gammaproteobacteria	Enterobacteriales	Enterobacteriaceae	Enterobacter	Enterobacter aerogenes	5591105	5613
2576861468	Enterobacter asburiae L1	Proteobacteria	Gammaproteobacteria	Enterobacteriales	Enterobacteriaceae	Enterobacter	Enterobacter asburiae	4561905	4278
646564529	Enterobacter cloacae cloacae ATCC 13047	Proteobacteria	Gammaproteobacteria	Enterobacteriales	Enterobacteriaceae	Enterobacter	Enterobacter cloacae	5598796	5627
650377928	Enterobacter cloacae cloacae NCTC 9394	Proteobacteria	Gammaproteobacteria	Enterobacteriales	Enterobacteriaceae	Enterobacter	Enterobacter cloacae	4908759	3797
2506520035	Enterobacter cloacae EcWSU1	Proteobacteria	Gammaproteobacteria	Enterobacteriales	Enterobacteriaceae	Enterobacter	Enterobacter cloacae	4798091	4740
2518645555	Enterobacter cloacae cloacae ENHKU01	Proteobacteria	Gammaproteobacteria	Enterobacteriales	Enterobacteriaceae	Enterobacter	Enterobacter cloacae	4726582	4445
2519103114	Enterobacter cloacae dissolvens SDM	Proteobacteria	Gammaproteobacteria	Enterobacteriales	Enterobacteriaceae	Enterobacter	Enterobacter cloacae	4968248	4646
2558860275	Enterobacter cloacae P101	Proteobacteria	Gammaproteobacteria	Enterobacteriales	Enterobacteriaceae	Enterobacter	Enterobacter cloacae	5369929	5289
2585427623	Enterobacter cloacae ECNIH2	Proteobacteria	Gammaproteobacteria	Enterobacteriales	Enterobacteriaceae	Enterobacter	Enterobacter cloacae	5502685	5263
2600254962	Enterobacter cloacae ECNIH3	Proteobacteria	Gammaproteobacteria	Enterobacteriales	Enterobacteriaceae	Enterobacter	Enterobacter cloacae	5061388	4935

2602041561	Enterobacter cloacae ECNIH1	Proteobacteria	Gammaproteobacteria	Enterobacteriales	Enterobacteriaceae	Enterobacter	Enterobacter cloacae	4959888	4800
649633041	Enterobacter cloacae SCF1	Proteobacteria	Gammaproteobacteria	Enterobacteriales	Enterobacteriaceae	Enterobacter	Enterobacter lignolyticus	4814049	4556
640427113	Enterobacter sp. 638	Proteobacteria	Gammaproteobacteria	Enterobacteriales	Enterobacteriaceae	Enterobacter	Enterobacter sp. 638	4676461	4444
2561511199	Enterobacter sp. R4-368	Proteobacteria	Gammaproteobacteria	Enterobacteriales	Enterobacteriaceae	Enterobacter	Enterobacter sp. R4-368	5155034	4948
646564530	Erwinia amylovora Ea273, ATCC 49946	Proteobacteria	Gammaproteobacteria	Enterobacteriales	Enterobacteriaceae	Erwinia	Erwinia amylovora	3905604	3665
646564531	Erwinia amylovora CFBP 1430	Proteobacteria	Gammaproteobacteria	Enterobacteriales	Enterobacteriaceae	Erwinia	Erwinia amylovora	3833832	3805
649633042	Erwinia billingiae Eb661	Proteobacteria	Gammaproteobacteria	Enterobacteriales	Enterobacteriaceae	Erwinia	Erwinia billingiae	5372268	5015
646564532	Erwinia pyrifoliae Ep1/96	Proteobacteria	Gammaproteobacteria	Enterobacteriales	Enterobacteriaceae	Erwinia	Erwinia pyrifoliae	4072846	3794
646862321	Erwinia pyrifoliae DSM 12163	Proteobacteria	Gammaproteobacteria	Enterobacteriales	Enterobacteriaceae	Erwinia	Erwinia pyrifoliae	4072827	4134
650377931	Erwinia sp. Ejp617	Proteobacteria	Gammaproteobacteria	Enterobacteriales	Enterobacteriaceae	Erwinia	Erwinia sp. Ejp617	3957675	3769
642555128	Erwinia tasmaniensis Et1/99	Proteobacteria	Gammaproteobacteria	Enterobacteriales	Enterobacteriaceae	Erwinia	Erwinia tasmaniensis	4067864	3730
2558860274	Escherichia albertii KF1	Proteobacteria	Gammaproteobacteria	Enterobacteriales	Enterobacteriaceae	Escherichia	Escherichia albertii	4701875	4529
637000104	*Escherichia coli* sv. O6:K15:H31 536	Proteobacteria	Gammaproteobacteria	Enterobacteriales	Enterobacteriaceae	Escherichia	*Escherichia coli*	4938920	4858
637000105	*Escherichia coli* sv. O6:K2:H1 CFT073	Proteobacteria	Gammaproteobacteria	Enterobacteriales	Enterobacteriaceae	Escherichia	*Escherichia coli*	5231428	5683
637000107	*Escherichia coli* O157:H7 EDL933 (EHEC)	Proteobacteria	Gammaproteobacteria	Enterobacteriales	Enterobacteriaceae	Escherichia	*Escherichia coli*	5620522	5633
637000108	*Escherichia coli* O157:H7 Sakai (EHEC)	Proteobacteria	Gammaproteobacteria	Enterobacteriales	Enterobacteriaceae	Escherichia	*Escherichia coli*	5594477	5556
637000109	*Escherichia coli* UTI89 (UPEC)	Proteobacteria	Gammaproteobacteria	Enterobacteriales	Enterobacteriaceae	Escherichia	*Escherichia coli*	5179971	5398
637000110	*Escherichia coli* K12- W3110	Proteobacteria	Gammaproteobacteria	Enterobacteriales	Enterobacteriaceae	Escherichia	*Escherichia coli*	4646332	4427
640753023	*Escherichia coli* sv. O1:K1:H7 APEC	Proteobacteria	Gammaproteobacteria	Enterobacteriales	Enterobacteriaceae	Escherichia	*Escherichia coli*	5497653	5168
640753024	*Escherichia coli* sv. O139:H28 E24377A (ETEC)	Proteobacteria	Gammaproteobacteria	Enterobacteriales	Enterobacteriaceae	Escherichia	*Escherichia coli*	5249288	5258
640753025	*Escherichia coli* sv. O9 HS	Proteobacteria	Gammaproteobacteria	Enterobacteriales	Enterobacteriaceae	Escherichia	*Escherichia coli*	4643538	4628
641522623	*Escherichia coli* C ATCC 8739	Proteobacteria	Gammaproteobacteria	Enterobacteriales	Enterobacteriaceae	Escherichia	*Escherichia coli*	4746218	4394
641522624	*Escherichia coli* SECEC SMS-3-5	Proteobacteria	Gammaproteobacteria	Enterobacteriales	Enterobacteriaceae	Escherichia	*Escherichia coli*	5215377	5025
641522625	*Escherichia coli* DH10B	Proteobacteria	Gammaproteobacteria	Enterobacteriales	Enterobacteriaceae	Escherichia	*Escherichia coli*	4686137	4271
643348544	*Escherichia coli* 55989	Proteobacteria	Gammaproteobacteria	Enterobacteriales	Enterobacteriaceae	Escherichia	*Escherichia coli*	5154862	4985
643348545	*Escherichia coli* O81 ED1a	Proteobacteria	Gammaproteobacteria	Enterobacteriales	Enterobacteriaceae	Escherichia	*Escherichia coli*	5209548	5116
643348546	*Escherichia coli* IAI1	Proteobacteria	Gammaproteobacteria	Enterobacteriales	Enterobacteriaceae	Escherichia	*Escherichia coli*	4700560	4548
643348547	*Escherichia coli* IAI39	Proteobacteria	Gammaproteobacteria	Enterobacteriales	Enterobacteriaceae	Escherichia	*Escherichia coli*	5132068	4938
643348548	*Escherichia coli* O127:H6 E2348/69 (EPEC)	Proteobacteria	Gammaproteobacteria	Enterobacteriales	Enterobacteriaceae	Escherichia	*Escherichia coli*	5069678	4843
643348549	*Escherichia coli* O157:H7 EC4115	Proteobacteria	Gammaproteobacteria	Enterobacteriales	Enterobacteriaceae	Escherichia	*Escherichia coli*	5704171	5609
643348551	*Escherichia coli* SE11	Proteobacteria	Gammaproteobacteria	Enterobacteriales	Enterobacteriaceae	Escherichia	*Escherichia coli*	5155626	5109
644736359	*Escherichia coli* B REL606	Proteobacteria	Gammaproteobacteria	Enterobacteriales	Enterobacteriaceae	Escherichia	*Escherichia coli*	4629812	4312
644736361	*Escherichia coli* BL21 (DE3)	Proteobacteria	Gammaproteobacteria	Enterobacteriales	Enterobacteriaceae	Escherichia	*Escherichia coli*	4570938	4436
644736362	*Escherichia coli* BW2952	Proteobacteria	Gammaproteobacteria	Enterobacteriales	Enterobacteriaceae	Escherichia	*Escherichia coli*	4578159	4194
644736363	*Escherichia coli* O157:H7 TW14359	Proteobacteria	Gammaproteobacteria	Enterobacteriales	Enterobacteriaceae	Escherichia	*Escherichia coli*	5622737	5500
644736364	*Escherichia coli* O45:K1 S88	Proteobacteria	Gammaproteobacteria	Enterobacteriales	Enterobacteriaceae	Escherichia	*Escherichia coli*	5166121	5037
644736365	*Escherichia coli* O17:K52:H18 UMN026	Proteobacteria	Gammaproteobacteria	Enterobacteriales	Enterobacteriaceae	Escherichia	*Escherichia coli*	5358200	5211
646311923	*Escherichia coli* O103:H2 12009	Proteobacteria	Gammaproteobacteria	Enterobacteriales	Enterobacteriaceae	Escherichia	*Escherichia coli*	5524860	5312
646311924	*Escherichia coli* O111:H 11128	Proteobacteria	Gammaproteobacteria	Enterobacteriales	Enterobacteriaceae	Escherichia	*Escherichia coli*	5766081	5610
646311926	*Escherichia coli* K-12, MG1655	Proteobacteria	Gammaproteobacteria	Enterobacteriales	Enterobacteriaceae	Escherichia	*Escherichia coli*	4639675	4497
646564533	*Escherichia coli* O55:H7 CB9615	Proteobacteria	Gammaproteobacteria	Enterobacteriales	Enterobacteriaceae	Escherichia	*Escherichia coli*	5452353	5294
646862322	*Escherichia coli* O44:H18 042 (EAEC)	Proteobacteria	Gammaproteobacteria	Enterobacteriales	Enterobacteriaceae	Escherichia	*Escherichia coli*	5355323	5153
646862323	*Escherichia coli* BL21 (DE3)	Proteobacteria	Gammaproteobacteria	Enterobacteriales	Enterobacteriaceae	Escherichia	*Escherichia coli*	4557041	4348
646862324	*Escherichia coli* BL21 (DE3)	Proteobacteria	Gammaproteobacteria	Enterobacteriales	Enterobacteriaceae	Escherichia	*Escherichia coli*	4558953	4266
646862325	*Escherichia coli* K12 DH1, ATCC 33849	Proteobacteria	Gammaproteobacteria	Enterobacteriales	Enterobacteriaceae	Escherichia	*Escherichia coli*	4630707	4273
646862326	*Escherichia coli* O18:K1:H7 IHE3034	Proteobacteria	Gammaproteobacteria	Enterobacteriales	Enterobacteriaceae	Escherichia	*Escherichia coli*	5108383	4875
646862327	*Escherichia coli* O150:H5 SE15	Proteobacteria	Gammaproteobacteria	Enterobacteriales	Enterobacteriaceae	Escherichia	*Escherichia coli*	4839683	4594
648028025	*Escherichia coli* O26:H11 11368	Proteobacteria	Gammaproteobacteria	Enterobacteriales	Enterobacteriaceae	Escherichia	*Escherichia coli*	5855531	5713
648231705	*Escherichia coli* ABU 83972	Proteobacteria	Gammaproteobacteria	Enterobacteriales	Enterobacteriaceae	Escherichia	*Escherichia coli*	5132961	4906
648231706	*Escherichia coli* AIEC UM146	Proteobacteria	Gammaproteobacteria	Enterobacteriales	Enterobacteriaceae	Escherichia	*Escherichia coli*	5107563	4891
650377933	*Escherichia coli* O83:H1 NRG 857C	Proteobacteria	Gammaproteobacteria	Enterobacteriales	Enterobacteriaceae	Escherichia	*Escherichia coli*	4894879	4688
650377934	*Escherichia coli* W, ATCC 9637	Proteobacteria	Gammaproteobacteria	Enterobacteriales	Enterobacteriaceae	Escherichia	*Escherichia coli*	5008864	4879
651053020	*Escherichia coli* DH1 (ME8569)	Proteobacteria	Gammaproteobacteria	Enterobacteriales	Enterobacteriaceae	Escherichia	*Escherichia coli*	4621430	4409
651053021	*Escherichia coli* LF82	Proteobacteria	Gammaproteobacteria	Enterobacteriales	Enterobacteriaceae	Escherichia	*Escherichia coli*	4773108	4534
651053022	*Escherichia coli* NA114	Proteobacteria	Gammaproteobacteria	Enterobacteriales	Enterobacteriaceae	Escherichia	*Escherichia coli*	4935241	4944

2511231131	*Escherichia coli* O55:H7 RM12579	Proteobacteria	Gammaproteobacteria	Enterobacteriales	Enterobacteriaceae	Escherichia	*Escherichia coli*	5448306	5255
2511231170	*Escherichia coli* clone D i14	Proteobacteria	Gammaproteobacteria	Enterobacteriales	Enterobacteriaceae	Escherichia	*Escherichia coli*	5038386	5049
2511231198	*Escherichia coli* clone D i2	Proteobacteria	Gammaproteobacteria	Enterobacteriales	Enterobacteriaceae	Escherichia	*Escherichia coli*	5038386	5049
2512047041	*Escherichia coli* O7:K1 CE10	Proteobacteria	Gammaproteobacteria	Enterobacteriales	Enterobacteriaceae	Escherichia	*Escherichia coli*	5378729	5269
2513237200	*Escherichia coli* KO11FL	Proteobacteria	Gammaproteobacteria	Enterobacteriales	Enterobacteriaceae	Escherichia	*Escherichia coli*	5027172	4825
2513237219	*Escherichia coli* P12b	Proteobacteria	Gammaproteobacteria	Enterobacteriales	Enterobacteriaceae	Escherichia	*Escherichia coli*	4935294	4581
2513237251	*Escherichia coli* Xuzhou21	Proteobacteria	Gammaproteobacteria	Enterobacteriales	Enterobacteriaceae	Escherichia	*Escherichia coli*	5516736	5298
2518645559	*Escherichia coli* O104:H4 2009EL-2050	Proteobacteria	Gammaproteobacteria	Enterobacteriales	Enterobacteriaceae	Escherichia	*Escherichia coli*	5438174	5304
2519899779	*Escherichia coli* O104:H4 2011C-3493	Proteobacteria	Gammaproteobacteria	Enterobacteriales	Enterobacteriaceae	Escherichia	*Escherichia coli*	5437407	5268
2519899780	*Escherichia coli* O104:H4 2009EL-2071	Proteobacteria	Gammaproteobacteria	Enterobacteriales	Enterobacteriaceae	Escherichia	*Escherichia coli*	5389708	5247
2521172717	*Escherichia coli* W, ATCC 9637	Proteobacteria	Gammaproteobacteria	Enterobacteriales	Enterobacteriaceae	Escherichia	*Escherichia coli*	5005347	4848
2522572021	*Escherichia coli* O145:H28 RM13514 (RM13514 finished genome)	Proteobacteria	Gammaproteobacteria	Enterobacteriales	Enterobacteriaceae	Escherichia	*Escherichia coli*	5737294	5861
2522572023	*Escherichia coli* O145:H28 RM13516 (RM13516 fininshed genome)	Proteobacteria	Gammaproteobacteria	Enterobacteriales	Enterobacteriaceae	Escherichia	*Escherichia coli*	5559008	5604
2540341091	*Escherichia coli* APEC O78	Proteobacteria	Gammaproteobacteria	Enterobacteriales	Enterobacteriaceae	Escherichia	*Escherichia coli*	4798435	4696
2554235344	*Escherichia coli* K-12 subMDS42	Proteobacteria	Gammaproteobacteria	Enterobacteriales	Enterobacteriaceae	Escherichia	*Escherichia coli*	3976195	3696
2554235419	*Escherichia coli* LY180	Proteobacteria	Gammaproteobacteria	Enterobacteriales	Enterobacteriaceae	Escherichia	*Escherichia coli*	4835601	4624
2558309052	*Escherichia coli* JJ1886	Proteobacteria	Gammaproteobacteria	Enterobacteriales	Enterobacteriaceae	Escherichia	*Escherichia coli*	5308284	5213
2558860227	*Escherichia coli* O145:H28 RM13516	Proteobacteria	Gammaproteobacteria	Enterobacteriales	Enterobacteriaceae	Escherichia	*Escherichia coli*	5559008	5635
2558860253	*Escherichia coli* C321.deltaA	Proteobacteria	Gammaproteobacteria	Enterobacteriales	Enterobacteriaceae	Escherichia	*Escherichia coli*	4643553	4511
2561511241	*Escherichia coli* UMNK88	Proteobacteria	Gammaproteobacteria	Enterobacteriales	Enterobacteriaceae	Escherichia	*Escherichia coli*	5666764	5726
2579778602	*Escherichia coli* O145:H28 RM12761	Proteobacteria	Gammaproteobacteria	Enterobacteriales	Enterobacteriaceae	Escherichia	*Escherichia coli*	5559014	5613
2582580752	*Escherichia coli* O145:H28 RM12581	Proteobacteria	Gammaproteobacteria	Enterobacteriales	Enterobacteriaceae	Escherichia	*Escherichia coli*	5737293	5895
2585427621	*Escherichia coli* O157:H7 SS17	Proteobacteria	Gammaproteobacteria	Enterobacteriales	Enterobacteriaceae	Escherichia	*Escherichia coli*	5655941	5721
2585427657	*Escherichia coli* Nissle 1917	Proteobacteria	Gammaproteobacteria	Enterobacteriales	Enterobacteriaceae	Escherichia	*Escherichia coli*	5441200	5126
2585427658	*Escherichia coli* O157:H7 EDL933	Proteobacteria	Gammaproteobacteria	Enterobacteriales	Enterobacteriaceae	Escherichia	*Escherichia coli*	5639399	5919
2588253749	*Escherichia coli* B7A	Proteobacteria	Gammaproteobacteria	Enterobacteriales	Enterobacteriaceae	Escherichia	*Escherichia coli*	5230440	5492
2597489870	*Escherichia coli* ETEC H10407	Proteobacteria	Gammaproteobacteria	Enterobacteriales	Enterobacteriaceae	Escherichia	*Escherichia coli*	5325888	5325
2597490154	*Escherichia coli* ATCC 25922	Proteobacteria	Gammaproteobacteria	Enterobacteriales	Enterobacteriaceae	Escherichia	*Escherichia coli*	5203440	5014
2597490314	*Escherichia coli* BW25113	Proteobacteria	Gammaproteobacteria	Enterobacteriales	Enterobacteriaceae	Escherichia	*Escherichia coli*	4631469	4497
2623620244	*Escherichia coli* sv. O139:H28 E24377A (ETEC) (re-annotation)	Proteobacteria	Gammaproteobacteria	Enterobacteriales	Enterobacteriaceae	Escherichia	*Escherichia coli*	5249288	5251
2623620245	*Escherichia coli* sv. O9 HS (re-annotation)	Proteobacteria	Gammaproteobacteria	Enterobacteriales	Enterobacteriaceae	Escherichia	*Escherichia coli*	4643538	4535
2623620342	*Escherichia coli* sv. O1:K1:H7 APEC (re-annotation)	Proteobacteria	Gammaproteobacteria	Enterobacteriales	Enterobacteriaceae	Escherichia	*Escherichia coli*	5497653	5485
2623620697	*Escherichia coli* O157:H7 Sakai (EHEC) (re-annotation)	Proteobacteria	Gammaproteobacteria	Enterobacteriales	Enterobacteriaceae	Escherichia	*Escherichia coli*	5594477	5657
2623620757	*Escherichia coli* K12- W3110 (re-annotation)	Proteobacteria	Gammaproteobacteria	Enterobacteriales	Enterobacteriaceae	Escherichia	*Escherichia coli*	4646332	4536
2623620781	*Escherichia coli* UTI89 (UPEC) (re-annotation)	Proteobacteria	Gammaproteobacteria	Enterobacteriales	Enterobacteriaceae	Escherichia	*Escherichia coli*	5179971	5055
2623620919	*Escherichia coli* sv. O6:K15:H31 536 (re-annotation)	Proteobacteria	Gammaproteobacteria	Enterobacteriales	Enterobacteriaceae	Escherichia	*Escherichia coli*	4938920	4734
2623620920	*Escherichia coli* sv. O6:K2:H1 CFT073 (re-annotation)	Proteobacteria	Gammaproteobacteria	Enterobacteriales	Enterobacteriaceae	Escherichia	*Escherichia coli*	5231428	5119
2623620921	*Escherichia coli* O157:H7 EDL933 (EHEC) (re-annotation)	Proteobacteria	Gammaproteobacteria	Enterobacteriales	Enterobacteriaceae	Escherichia	*Escherichia coli*	5620522	5681
643692022	Escherichia fergusonii UMN026, ATCC 35469	Proteobacteria	Gammaproteobacteria	Enterobacteriales	Enterobacteriaceae	Escherichia	Escherichia fergusonii	4643861	4498
2511231124	Klebsiella oxytoca KCTC 1686	Proteobacteria	Gammaproteobacteria	Enterobacteriales	Enterobacteriaceae	Klebsiella	Klebsiella oxytoca	5974109	5598
2561511178	Klebsiella oxytoca E718	Proteobacteria	Gammaproteobacteria	Enterobacteriales	Enterobacteriaceae	Klebsiella	Klebsiella oxytoca	6561678	6170
2585427624	Klebsiella oxytoca KONIH1	Proteobacteria	Gammaproteobacteria	Enterobacteriales	Enterobacteriaceae	Klebsiella	Klebsiella oxytoca	6684898	6322
2585427669	Klebsiella oxytoca M1	Proteobacteria	Gammaproteobacteria	Enterobacteriales	Enterobacteriaceae	Klebsiella	Klebsiella oxytoca	6311385	5837
640753032	Klebsiella pneumoniae pneumoniae MGH78578	Proteobacteria	Gammaproteobacteria	Enterobacteriales	Enterobacteriaceae	Klebsiella	Klebsiella pneumoniae	5694894	5377
643348560	Klebsiella pneumoniae 342	Proteobacteria	Gammaproteobacteria	Enterobacteriales	Enterobacteriaceae	Klebsiella	Klebsiella pneumoniae	5920257	5881
646564538	Klebsiella pneumoniae NTUH-K2044	Proteobacteria	Gammaproteobacteria	Enterobacteriales	Enterobacteriaceae	Klebsiella	Klebsiella pneumoniae	5472672	5385
651053036	Klebsiella pneumoniae KCTC 2242	Proteobacteria	Gammaproteobacteria	Enterobacteriales	Enterobacteriaceae	Klebsiella	Klebsiella pneumoniae	5462423	5264
2511231199	Klebsiella pneumoniae pneumoniae HS11286	Proteobacteria	Gammaproteobacteria	Enterobacteriales	Enterobacteriaceae	Klebsiella	Klebsiella pneumoniae	5682322	5866
2540341071	Klebsiella pneumoniae pneumoniae 1084	Proteobacteria	Gammaproteobacteria	Enterobacteriales	Enterobacteriaceae	Klebsiella	Klebsiella pneumoniae	5386705	5069
2551306645	Klebsiella pneumoniae ATCC BAA-2146	Proteobacteria	Gammaproteobacteria	Enterobacteriales	Enterobacteriaceae	Klebsiella	Klebsiella pneumoniae	5644669	6133
2554235385	Klebsiella pneumoniae CG43	Proteobacteria	Gammaproteobacteria	Enterobacteriales	Enterobacteriaceae	Klebsiella	Klebsiella pneumoniae	5166857	4940
2554235445	Klebsiella pneumoniae JM45	Proteobacteria	Gammaproteobacteria	Enterobacteriales	Enterobacteriaceae	Klebsiella	Klebsiella pneumoniae	5603174	5249
2558860204	Klebsiella pneumoniae pneumoniae Kp13	Proteobacteria	Gammaproteobacteria	Enterobacteriales	Enterobacteriaceae	Klebsiella	Klebsiella pneumoniae	5739888	5868
2561511244	Klebsiella pneumoniae rhinoscleromatis SB3432	Proteobacteria	Gammaproteobacteria	Enterobacteriales	Enterobacteriaceae	Klebsiella	Klebsiella pneumoniae	5384455	5362
2565956562	Klebsiella pneumoniae 30660/NJST258_1	Proteobacteria	Gammaproteobacteria	Enterobacteriales	Enterobacteriaceae	Klebsiella	Klebsiella pneumoniae	5540936	5901

2565956563	Klebsiella pneumoniae 30684/NJST258_2	Proteobacteria	Gammaproteobacteria	Enterobacteriales	Enterobacteriaceae	Klebsiella	Klebsiella pneumoniae	5417216	5702
2588253732	Klebsiella pneumoniae pneumoniae KP5-1	Proteobacteria	Gammaproteobacteria	Enterobacteriales	Enterobacteriaceae	Klebsiella	Klebsiella pneumoniae	5551467	5198
2588253733	Klebsiella pneumoniae pneumoniae KPNIH24	Proteobacteria	Gammaproteobacteria	Enterobacteriales	Enterobacteriaceae	Klebsiella	Klebsiella pneumoniae	5734564	5487
2588253736	Klebsiella pneumoniae pneumoniae KPNIH27	Proteobacteria	Gammaproteobacteria	Enterobacteriales	Enterobacteriaceae	Klebsiella	Klebsiella pneumoniae	6132443	5885
2588253737	Klebsiella pneumoniae pneumoniae KPNIH28	Proteobacteria	Gammaproteobacteria	Enterobacteriales	Enterobacteriaceae	Klebsiella	Klebsiella pneumoniae	5436785	5183
2588253741	Klebsiella pneumoniae pneumoniae PittNDM01	Proteobacteria	Gammaproteobacteria	Enterobacteriales	Enterobacteriaceae	Klebsiella	Klebsiella pneumoniae	5812304	5563
2597490161	Klebsiella pneumoniae blaNDM-1	Proteobacteria	Gammaproteobacteria	Enterobacteriales	Enterobacteriaceae	Klebsiella	Klebsiella pneumoniae	5510332	5352
2597490169	Klebsiella pneumoniae VK055	Proteobacteria	Gammaproteobacteria	Enterobacteriales	Enterobacteriaceae	Klebsiella	Klebsiella pneumoniae	5374834	5087
2623620227	Klebsiella pneumoniae pneumoniae MGH78578 (re-annotation)	Proteobacteria	Gammaproteobacteria	Enterobacteriales	Enterobacteriaceae	Klebsiella	Klebsiella pneumoniae	5694894	5506
646311937	Klebsiella variicola At-22	Proteobacteria	Gammaproteobacteria	Enterobacteriales	Enterobacteriaceae	Klebsiella	Klebsiella variicola	5458505	5213
2547132181	Kosakonia sacchari SP1	Proteobacteria	Gammaproteobacteria	Enterobacteriales	Enterobacteriaceae	Kosakonia	Kosakonia sacchari	4945084	4887
2540341130	Morganella morganii morganii KT	Proteobacteria	Gammaproteobacteria	Enterobacteriales	Enterobacteriaceae	Morganella	Morganella morganii	3799539	3624
2576861443	Halyomorpha halys symbiont	Proteobacteria	Gammaproteobacteria	Enterobacteriales	Enterobacteriaceae	Pantoea	Halyomorpha halys symbiont	1151074	873
646564558	Pantoea ananatis LMG 20103	Proteobacteria	Gammaproteobacteria	Enterobacteriales	Enterobacteriaceae	Pantoea	Pantoea ananatis	4690298	4340
651053056	Pantoea ananatis AJ13355	Proteobacteria	Gammaproteobacteria	Enterobacteriales	Enterobacteriaceae	Pantoea	Pantoea ananatis	4877280	4167
2511231160	Pantoea ananatis PA13	Proteobacteria	Gammaproteobacteria	Enterobacteriales	Enterobacteriaceae	Pantoea	Pantoea ananatis	4867131	4542
2511231178	Pantoea ananatis LMG 5342	Proteobacteria	Gammaproteobacteria	Enterobacteriales	Enterobacteriaceae	Pantoea	Pantoea ananatis	4908144	4763
649633081	Pantoea sp. At-9b	Proteobacteria	Gammaproteobacteria	Enterobacteriales	Enterobacteriaceae	Pantoea	Pantoea sp. At-9b	6312783	6007
648028049	Pantoea vagans C9-1	Proteobacteria	Gammaproteobacteria	Enterobacteriales	Enterobacteriaceae	Pantoea	Pantoea vagans	4888338	4689
637000102	Pectobacterium atrosepticum SCRI1043	Proteobacteria	Gammaproteobacteria	Enterobacteriales	Enterobacteriaceae	Pectobacterium	Pectobacterium atrosepticum	5064019	4637
2576861483	Pectobacterium atrosepticum JG10-08	Proteobacteria	Gammaproteobacteria	Enterobacteriales	Enterobacteriaceae	Pectobacterium	Pectobacterium atrosepticum	5004926	4399
2597490164	Pectobacterium atrosepticum 21a	Proteobacteria	Gammaproteobacteria	Enterobacteriales	Enterobacteriaceae	Pectobacterium	Pectobacterium atrosepticum	5024250	4602
2623620696	Pectobacterium atrosepticum SCRI1043 (re-annotation)	Proteobacteria	Gammaproteobacteria	Enterobacteriales	Enterobacteriaceae	Pectobacterium	Pectobacterium atrosepticum	5064019	4626
644736397	Pectobacterium carotovorum carotovorum PC1	Proteobacteria	Gammaproteobacteria	Enterobacteriales	Enterobacteriaceae	Pectobacterium	Pectobacterium carotovorum	4862913	4395
2518645533	Pectobacterium carotovorum carotovorum PCC21	Proteobacteria	Gammaproteobacteria	Enterobacteriales	Enterobacteriaceae	Pectobacterium	Pectobacterium carotovorum	4842771	4361
2526164723	Pectobacterium sp. SCC3193	Proteobacteria	Gammaproteobacteria	Enterobacteriales	Enterobacteriaceae	Pectobacterium	Pectobacterium sp. SCC3193	5164411	4804
646311947	Pectobacterium wasabiae WPP163	Proteobacteria	Gammaproteobacteria	Enterobacteriales	Enterobacteriaceae	Pectobacterium	Pectobacterium wasabiae	5063892	4717
644736399	Photorhabdus asymbiotica asymbiotica ATCC 43949	Proteobacteria	Gammaproteobacteria	Enterobacteriales	Enterobacteriaceae	Photorhabdus	Photorhabdus asymbiotica	5094138	4555
637000207	Photorhabdus luminescens laumondii TTO1	Proteobacteria	Gammaproteobacteria	Enterobacteriales	Enterobacteriaceae	Photorhabdus	Photorhabdus luminescens	5688987	5052
2623620958	Photorhabdus luminescens laumondii TTO1 (re-annotation)	Proteobacteria	Gammaproteobacteria	Enterobacteriales	Enterobacteriaceae	Photorhabdus	Photorhabdus luminescens	5688987	5048
642555150	Proteus mirabilis HI4320	Proteobacteria	Gammaproteobacteria	Enterobacteriales	Enterobacteriaceae	Proteus	Proteus mirabilis	4099895	3784
2545824633	Proteus mirabilis BB2000	Proteobacteria	Gammaproteobacteria	Enterobacteriales	Enterobacteriaceae	Proteus	Proteus mirabilis	3846754	3558
2513237180	Providencia stuartii MRSN 2154	Proteobacteria	Gammaproteobacteria	Enterobacteriales	Enterobacteriaceae	Providencia	Providencia stuartii	4402109	4196
2597490145	Providencia stuartii 33672	Proteobacteria	Gammaproteobacteria	Enterobacteriales	Enterobacteriaceae	Providencia	Providencia stuartii	4334817	4053
2506520038	Rahnella aquatilis CIP 78.65, ATCC 33071	Proteobacteria	Gammaproteobacteria	Enterobacteriales	Enterobacteriaceae	Rahnella	Rahnella aquatilis	5447000	5118
2561511173	Rahnella aquatilis HX2	Proteobacteria	Gammaproteobacteria	Enterobacteriales	Enterobacteriaceae	Rahnella	Rahnella aquatilis	5656799	5180
649633088	Rahnella sp. Y9602	Proteobacteria	Gammaproteobacteria	Enterobacteriales	Enterobacteriaceae	Rahnella	Rahnella sp. Y9602	5614252	5285
2540341161	Raoultella ornithinolytica B6	Proteobacteria	Gammaproteobacteria	Enterobacteriales	Enterobacteriaceae	Raoultella	Raoultella ornithinolytica	5398151	5013
650716084	Salmonella bongori NCTC 12419	Proteobacteria	Gammaproteobacteria	Enterobacteriales	Enterobacteriaceae	Salmonella	Salmonella bongori	4460105	3969
2561511179	Salmonella bongori Sbon_167	Proteobacteria	Gammaproteobacteria	Enterobacteriales	Enterobacteriaceae	Salmonella	Salmonella bongori	4773537	4878
2585427625	Salmonella bongori sv. 48:z41 RKS3044	Proteobacteria	Gammaproteobacteria	Enterobacteriales	Enterobacteriaceae	Salmonella	Salmonella bongori	4394500	4121
637000251	Salmonella enterica enterica sv. Choleraesuis SC-B67	Proteobacteria	Gammaproteobacteria	Enterobacteriales	Enterobacteriaceae	Salmonella	Salmonella enterica	4944000	4996
637000252	Salmonella enterica enterica sv. Paratyphi A	Proteobacteria	Gammaproteobacteria	Enterobacteriales	Enterobacteriaceae	Salmonella	Salmonella enterica	4585229	4295
637000253	Salmonella enterica enterica sv. Typhi Ty2	Proteobacteria	Gammaproteobacteria	Enterobacteriales	Enterobacteriaceae	Salmonella	Salmonella enterica	4791961	4719
637000254	Salmonella enterica enterica sv. Typhi CT18	Proteobacteria	Gammaproteobacteria	Enterobacteriales	Enterobacteriaceae	Salmonella	Salmonella enterica	5133713	5165
637000255	Salmonella enterica enterica sv. Typhimurium LT2	Proteobacteria	Gammaproteobacteria	Enterobacteriales	Enterobacteriaceae	Salmonella	Salmonella enterica	4951371	4781
641228505	Salmonella enterica arizonae sv. 62:z4,z23 RSK2980	Proteobacteria	Gammaproteobacteria	Enterobacteriales	Enterobacteriaceae	Salmonella	Salmonella enterica	4600800	4695
641228506	Salmonella enterica enterica sv. Paratyphi B SPB7	Proteobacteria	Gammaproteobacteria	Enterobacteriales	Enterobacteriaceae	Salmonella	Salmonella enterica	4858887	5772
642555154	Salmonella enterica sv. Agona SL483	Proteobacteria	Gammaproteobacteria	Enterobacteriales	Enterobacteriaceae	Salmonella	Salmonella enterica	4836638	4720
642555155	Salmonella enterica sv. Dublin CT_02021853	Proteobacteria	Gammaproteobacteria	Enterobacteriales	Enterobacteriaceae	Salmonella	Salmonella enterica	4917459	4721
642555156	Salmonella enterica sv. Heidelberg SL476	Proteobacteria	Gammaproteobacteria	Enterobacteriales	Enterobacteriaceae	Salmonella	Salmonella enterica	4983515	4884
642555157	Salmonella enterica sv. Newport SL254	Proteobacteria	Gammaproteobacteria	Enterobacteriales	Enterobacteriaceae	Salmonella	Salmonella enterica	5007719	4913
642555158	Salmonella enterica sv. Paratyphi A AKU_12601	Proteobacteria	Gammaproteobacteria	Enterobacteriales	Enterobacteriaceae	Salmonella	Salmonella enterica	4581797	4208
642555159	Salmonella enterica enterica sv. Schwarzengrund CVM19633	Proteobacteria	Gammaproteobacteria	Enterobacteriales	Enterobacteriaceae	Salmonella	Salmonella enterica	4823887	4730
643348572	Salmonella enterica enterica sv. Enteritidis P125109	Proteobacteria	Gammaproteobacteria	Enterobacteriales	Enterobacteriaceae	Salmonella	Salmonella enterica	4685848	4312

643348573	Salmonella enterica enterica sv. Gallinarum 287/91	Proteobacteria	Gammaproteobacteria	Enterobacteriales	Enterobacteriaceae	Salmonella	Salmonella enterica	4658697	4062
643692035	Salmonella enterica sv. Paratyphi C RKS4594	Proteobacteria	Gammaproteobacteria	Enterobacteriales	Enterobacteriaceae	Salmonella	Salmonella enterica	4888494	4743
646862339	Salmonella enterica enterica sv, Typhimurium D23580	Proteobacteria	Gammaproteobacteria	Enterobacteriales	Enterobacteriaceae	Salmonella	Salmonella enterica	4879400	4631
646862340	Salmonella enterica enterica sv. Typhimurium 14028S	Proteobacteria	Gammaproteobacteria	Enterobacteriales	Enterobacteriaceae	Salmonella	Salmonella enterica	4964097	5591
650377971	Salmonella enterica enterica sv Typhimurium 4/74	Proteobacteria	Gammaproteobacteria	Enterobacteriales	Enterobacteriaceae	Salmonella	Salmonella enterica	5067451	4948
651053064	Salmonella enterica enterica sv. Typhimurium T000240	Proteobacteria	Gammaproteobacteria	Enterobacteriales	Enterobacteriaceae	Salmonella	Salmonella enterica	5069994	4977
651053065	Salmonella enterica enterica sv. Typhimurium UK-1	Proteobacteria	Gammaproteobacteria	Enterobacteriales	Enterobacteriaceae	Salmonella	Salmonella enterica	4911145	4662
2511231164	Salmonella enterica enterica sv. Typhi P-stx-12	Proteobacteria	Gammaproteobacteria	Enterobacteriales	Enterobacteriaceae	Salmonella	Salmonella enterica	4949783	5120
2511231218	Salmonella enterica enterica sv. Gallinarum RKS5078	Proteobacteria	Gammaproteobacteria	Enterobacteriales	Enterobacteriaceae	Salmonella	Salmonella enterica	4637962	4682
2512564066	Salmonella enterica enterica sv. Typhimurium 798	Proteobacteria	Gammaproteobacteria	Enterobacteriales	Enterobacteriaceae	Salmonella	Salmonella enterica	4970096	4579
2518645560	Salmonella enterica enterica sv. Heidelberg B182	Proteobacteria	Gammaproteobacteria	Enterobacteriales	Enterobacteriaceae	Salmonella	Salmonella enterica	4788046	4626
2540341126	Salmonella enterica enterica sv. Typhi Ty21a	Proteobacteria	Gammaproteobacteria	Enterobacteriales	Enterobacteriaceae	Salmonella	Salmonella enterica	4791958	4609
2541047617	Salmonella enterica enterica sv. Enteritidis CDC_2010K_0968	Proteobacteria	Gammaproteobacteria	Enterobacteriales	Enterobacteriaceae	Salmonella	Salmonella enterica	4733657	4595
2554235325	Salmonella enterica enterica sv. Newport USMARC-S3124.1	Proteobacteria	Gammaproteobacteria	Enterobacteriales	Enterobacteriaceae	Salmonella	Salmonella enterica	4915960	4937
2554235342	Salmonella enterica enterica sv. Thompson RM6836	Proteobacteria	Gammaproteobacteria	Enterobacteriales	Enterobacteriaceae	Salmonella	Salmonella enterica	4707648	4482
2554235343	Salmonella enterica Enterica sv. Heidelberg 41578	Proteobacteria	Gammaproteobacteria	Enterobacteriales	Enterobacteriaceae	Salmonella	Salmonella enterica	4951478	4819
2554235366	Salmonella enterica enterica sv. Gallinarum/Pullorum CDC1983-67	Proteobacteria	Gammaproteobacteria	Enterobacteriales	Enterobacteriaceae	Salmonella	Salmonella enterica	4623089	4669
2554235392	Salmonella enterica enterica sv. Pullorum S06004	Proteobacteria	Gammaproteobacteria	Enterobacteriales	Enterobacteriaceae	Salmonella	Salmonella enterica	4682599	4465
2554235427	Salmonella enterica enterica sv. Heidelberg CFSAN002069	Proteobacteria	Gammaproteobacteria	Enterobacteriales	Enterobacteriaceae	Salmonella	Salmonella enterica	4932086	4744
2554235428	Salmonella enterica enterica sv. Cubana CFSAN002050	Proteobacteria	Gammaproteobacteria	Enterobacteriales	Enterobacteriaceae	Salmonella	Salmonella enterica	5267011	5117
2554235429	Salmonella enterica enterica sv. 4,[5],12:i:- 08-1736	Proteobacteria	Gammaproteobacteria	Enterobacteriales	Enterobacteriaceae	Salmonella	Salmonella enterica	4822189	4624
2554235431	Salmonella enterica enterica sv. Typhimurium var. 5- CFSAN001921	Proteobacteria	Gammaproteobacteria	Enterobacteriales	Enterobacteriaceae	Salmonella	Salmonella enterica	5089224	4934
2558309073	Salmonella enterica enterica sv. Agona 24249	Proteobacteria	Gammaproteobacteria	Enterobacteriales	Enterobacteriaceae	Salmonella	Salmonella enterica	4762840	4543
2558309090	Salmonella enterica enterica sv. Typhimurium DT104	Proteobacteria	Gammaproteobacteria	Enterobacteriales	Enterobacteriaceae	Salmonella	Salmonella enterica	5027665	4765
2558309121	Salmonella enterica enterica sv. Bovismorbificans 3114	Proteobacteria	Gammaproteobacteria	Enterobacteriales	Enterobacteriaceae	Salmonella	Salmonella enterica	4770529	4826
2561511177	Salmonella enterica enterica sv. Typhimurium U288	Proteobacteria	Gammaproteobacteria	Enterobacteriales	Enterobacteriaceae	Salmonella	Salmonella enterica	5017059	4905
2561511194	Salmonella enterica enterica sv. Javiana CFSAN001992	Proteobacteria	Gammaproteobacteria	Enterobacteriales	Enterobacteriaceae	Salmonella	Salmonella enterica	4675267	4512
2561511201	Salmonella enterica enterica sv. Bareilly CFSAN000189	Proteobacteria	Gammaproteobacteria	Enterobacteriales	Enterobacteriaceae	Salmonella	Salmonella enterica	4808805	4576
2563366604	Salmonella enterica enterica sv. Typhimurium SL1344	Proteobacteria	Gammaproteobacteria	Enterobacteriales	Enterobacteriaceae	Salmonella	Salmonella enterica	5067450	4967
2579778582	Salmonella enterica enterica sv. Bredeney CFSAN001080	Proteobacteria	Gammaproteobacteria	Enterobacteriales	Enterobacteriaceae	Salmonella	Salmonella enterica	4603849	4282
2579778758	Salmonella enterica enterica sv. Enteritidis Durban	Proteobacteria	Gammaproteobacteria	Enterobacteriales	Enterobacteriaceae	Salmonella	Salmonella enterica	4738298	4454
2579778962	Salmonella enterica enterica sv. Typhimurium 138736	Proteobacteria	Gammaproteobacteria	Enterobacteriales	Enterobacteriaceae	Salmonella	Salmonella enterica	5025357	4754
2579779080	Salmonella enterica enterica sv. Heidelberg CFSAN002064	Proteobacteria	Gammaproteobacteria	Enterobacteriales	Enterobacteriaceae	Salmonella	Salmonella enterica	4821559	4621
2585427639	Salmonella enterica enterica sv. Typhimurium VNP20009	Proteobacteria	Gammaproteobacteria	Enterobacteriales	Enterobacteriaceae	Salmonella	Salmonella enterica	4857390	4977
2597490089	Salmonella enterica enterica sv. Enteritidis SEJ	Proteobacteria	Gammaproteobacteria	Enterobacteriales	Enterobacteriaceae	Salmonella	Salmonella enterica	4738299	4595
2597490160	Salmonella enterica enterica sv. Choleraesuis C500	Proteobacteria	Gammaproteobacteria	Enterobacteriales	Enterobacteriaceae	Salmonella	Salmonella enterica	4751585	4696
2597490268	Salmonella enterica enterica sv. typhimurium ATCC 13311	Proteobacteria	Gammaproteobacteria	Enterobacteriales	Enterobacteriaceae	Salmonella	Salmonella enterica	4831756	4667
2619619292	Salmonella enterica arizonae sv. 62:z36:- RKS2983 Version 1	Proteobacteria	Gammaproteobacteria	Enterobacteriales	Enterobacteriaceae	Salmonella	Salmonella enterica	4574846	4433
2623620646	Salmonella enterica enterica sv. Choleraesuis SC-B67 (re-annotation)	Proteobacteria	Gammaproteobacteria	Enterobacteriales	Enterobacteriaceae	Salmonella	Salmonella enterica	4944000	5001
2623620647	Salmonella enterica enterica sv. Paratyphi A (re-annotation)	Proteobacteria	Gammaproteobacteria	Enterobacteriales	Enterobacteriaceae	Salmonella	Salmonella enterica	4585229	4541
2623620648	Salmonella enterica enterica sv. Typhi Ty2 (re-annotation)	Proteobacteria	Gammaproteobacteria	Enterobacteriales	Enterobacteriaceae	Salmonella	Salmonella enterica	4791961	4827
2623620649	Salmonella enterica enterica sv. Typhimurium LT2 (re-annotation)	Proteobacteria	Gammaproteobacteria	Enterobacteriales	Enterobacteriaceae	Salmonella	Salmonella enterica	4951371	4842
2623620950	Salmonella enterica enterica sv. Typhi CT18 (re-annotation)	Proteobacteria	Gammaproteobacteria	Enterobacteriales	Enterobacteriaceae	Salmonella	Salmonella enterica	5133713	5266
2558860259	Serratia fonticola RB-25	Proteobacteria	Gammaproteobacteria	Enterobacteriales	Enterobacteriaceae	Serratia	Serratia fonticola	5485588	5028
2563366574	Serratia liquefaciens ATCC 27592	Proteobacteria	Gammaproteobacteria	Enterobacteriales	Enterobacteriaceae	Serratia	Serratia liquefaciens	5282719	5023
2517093050	Serratia marcescens FGI94	Proteobacteria	Gammaproteobacteria	Enterobacteriales	Enterobacteriaceae	Serratia	Serratia marcescens	4858216	4609
2540341169	Serratia marcescens WW4	Proteobacteria	Gammaproteobacteria	Enterobacteriales	Enterobacteriaceae	Serratia	Serratia marcescens	5241455	4919
2585427672	Serratia marcescens marcescens Db11	Proteobacteria	Gammaproteobacteria	Enterobacteriales	Enterobacteriaceae	Serratia	Serratia marcescens	5113802	4831
2506520007	Serratia plymuthica AS9	Proteobacteria	Gammaproteobacteria	Enterobacteriales	Enterobacteriaceae	Serratia	Serratia plymuthica	5442880	5138
2534682368	Serratia odorifera 4Rx13	Proteobacteria	Gammaproteobacteria	Enterobacteriales	Enterobacteriaceae	Serratia	Serratia plymuthica	5403731	4799
2554235463	Serratia plymuthica S13	Proteobacteria	Gammaproteobacteria	Enterobacteriales	Enterobacteriaceae	Serratia	Serratia plymuthica	5467306	5125
2576861477	Serratia plymuthica V4	Proteobacteria	Gammaproteobacteria	Enterobacteriales	Enterobacteriaceae	Serratia	Serratia plymuthica	5513353	5081
640753048	Serratia proteamaculans 568	Proteobacteria	Gammaproteobacteria	Enterobacteriales	Enterobacteriaceae	Serratia	Serratia proteamaculans	5495657	5063
2506520008	Serratia plymuthica AS12	Proteobacteria	Gammaproteobacteria	Enterobacteriales	Enterobacteriaceae	Serratia	Serratia sp. AS12	5443009	5139
2506520009	Serratia plymuthica AS13	Proteobacteria	Gammaproteobacteria	Enterobacteriales	Enterobacteriaceae	Serratia	Serratia sp. AS13	5442549	5139

2526164535	Serratia sp. ATCC 39006	Proteobacteria	Gammaproteobacteria	Enterobacteriales	Enterobacteriaceae	Serratia	Serratia sp. ATCC 39006	4947661	4578
2576861482	Serratia sp. FS14	Proteobacteria	Gammaproteobacteria	Enterobacteriales	Enterobacteriaceae	Serratia	Serratia sp. FS14	5249875	4873
2511231072	Serratia symbiotica Cinara cedri	Proteobacteria	Gammaproteobacteria	Enterobacteriales	Enterobacteriaceae	Serratia	Serratia symbiotica	1762765	772
637000261	Shigella boydii sv. 4 Sb227	Proteobacteria	Gammaproteobacteria	Enterobacteriales	Enterobacteriaceae	Shigella	Shigella boydii	4646520	4640
641522650	Shigella boydii sv. 18 CDC 3083-94	Proteobacteria	Gammaproteobacteria	Enterobacteriales	Enterobacteriaceae	Shigella	Shigella boydii	4874659	4725
2623620651	Shigella boydii sv. 4 Sb227 (re-annotation)	Proteobacteria	Gammaproteobacteria	Enterobacteriales	Enterobacteriaceae	Shigella	Shigella boydii	4646520	5085
640427142	Shigella dysenteriae sv. 1 Sd197	Proteobacteria	Gammaproteobacteria	Enterobacteriales	Enterobacteriaceae	Shigella	Shigella dysenteriae	4560911	4912
2623620233	Shigella dysenteriae sv. 1 Sd197 (re-annotation)	Proteobacteria	Gammaproteobacteria	Enterobacteriales	Enterobacteriaceae	Shigella	Shigella dysenteriae	4560911	5427
637000263	Shigella flexneri sv. 2a 2457T	Proteobacteria	Gammaproteobacteria	Enterobacteriales	Enterobacteriaceae	Shigella	Shigella flexneri	4599354	4604
637000264	Shigella flexneri sv. 2a 301	Proteobacteria	Gammaproteobacteria	Enterobacteriales	Enterobacteriaceae	Shigella	Shigella flexneri	4828821	4865
637000265	Shigella flexneri sv. 5 8401	Proteobacteria	Gammaproteobacteria	Enterobacteriales	Enterobacteriaceae	Shigella	Shigella flexneri	4574284	4466
646862341	Shigella flexneri sv. Fxv 2002017	Proteobacteria	Gammaproteobacteria	Enterobacteriales	Enterobacteriaceae	Shigella	Shigella flexneri	4894492	4829
2597490082	Shigella flexneri 2003036	Proteobacteria	Gammaproteobacteria	Enterobacteriales	Enterobacteriaceae	Shigella	Shigella flexneri	4595814	4918
2597490083	Shigella flexneri Shi06HN006	Proteobacteria	Gammaproteobacteria	Enterobacteriales	Enterobacteriaceae	Shigella	Shigella flexneri	4620903	4968
2623620652	Shigella flexneri sv. 2a 2457T (re-annotation)	Proteobacteria	Gammaproteobacteria	Enterobacteriales	Enterobacteriaceae	Shigella	Shigella flexneri	4599354	4957
2623620653	Shigella flexneri sv. 2a 301 (re-annotation)	Proteobacteria	Gammaproteobacteria	Enterobacteriales	Enterobacteriaceae	Shigella	Shigella flexneri	4828820	5271
2623620654	Shigella flexneri sv. 5 8401 (re-annotation)	Proteobacteria	Gammaproteobacteria	Enterobacteriales	Enterobacteriaceae	Shigella	Shigella flexneri	4574284	4909
640427143	Shigella sonnei Ss046	Proteobacteria	Gammaproteobacteria	Enterobacteriales	Enterobacteriaceae	Shigella	Shigella sonnei	5055316	4841
2558860170	Shigella sonnei 53G	Proteobacteria	Gammaproteobacteria	Enterobacteriales	Enterobacteriaceae	Shigella	Shigella sonnei	5220473	5459
2623620226	Shigella sonnei Ss046 (re-annotation)	Proteobacteria	Gammaproteobacteria	Enterobacteriales	Enterobacteriaceae	Shigella	Shigella sonnei	5055316	5351
2513020017	Shimwellia blattae DSM 4481	Proteobacteria	Gammaproteobacteria	Enterobacteriales	Enterobacteriaceae	Shimwellia	Shimwellia blattae	4158725	4017
2597489865	Endosymbiont of Sitophilus oryzae	Proteobacteria	Gammaproteobacteria	Enterobacteriales	Enterobacteriaceae	Sodalis	Candidatus Sodalis pierantonius	4513140	5355
637000270	Sodalis glossinidius morsitans	Proteobacteria	Gammaproteobacteria	Enterobacteriales	Enterobacteriaceae	Sodalis	Sodalis glossinidius	4292502	2662
2623620656	Sodalis glossinidius morsitans (re-annotation)	Proteobacteria	Gammaproteobacteria	Enterobacteriales	Enterobacteriaceae	Sodalis	Sodalis glossinidius	4292502	5898
2576861453	Sodalis sp. HS1	Proteobacteria	Gammaproteobacteria	Enterobacteriales	Enterobacteriaceae	Sodalis	Sodalis sp. HS1	5159425	4460
2507262057	Enterobacteriaceae bacterium FGI 57	Proteobacteria	Gammaproteobacteria	Enterobacteriales	Enterobacteriaceae	unclassified	Enterobacteriaceae bacterium strain FGI 57	4762179	4548
2509601000	secondary endosymbiont of Ctenarytaina eucalypti Thao2000	Proteobacteria	Gammaproteobacteria	Enterobacteriales	Enterobacteriaceae	unclassified	secondary endosymbiont of Ctenarytaina eucalypti	1441139	965
2512047046	Secondary Endosymbiont of Heteropsylla cubana	Proteobacteria	Gammaproteobacteria	Enterobacteriales	Enterobacteriaceae	unclassified	secondary endosymbiont of Heteropsylla cubana	1121596	625
637000338	Wigglesworthia glossinidia endosymbiont of Glossina brevipalpis	Proteobacteria	Gammaproteobacteria	Enterobacteriales	Enterobacteriaceae	Wigglesworthia	Wigglesworthia glossinidia	703004	659
2511231135	Wigglesworthia glossinidia endosymbiont of Glossina morsitans	Proteobacteria	Gammaproteobacteria	Enterobacteriales	Enterobacteriaceae	Wigglesworthia	Wigglesworthia glossinidia	719535	678
2623620746	Wigglesworthia glossinidia endosymbiont of Glossina brevipalpis (re-annotation)	Proteobacteria	Gammaproteobacteria	Enterobacteriales	Enterobacteriaceae	Wigglesworthia	Wigglesworthia glossinidia	703004	687
646564589	Xenorhabdus bovienii SS-2004	Proteobacteria	Gammaproteobacteria	Enterobacteriales	Enterobacteriaceae	Xenorhabdus	Xenorhabdus bovienii	4225498	4373
649633108	Xenorhabdus nematophila ATCC 19061	Proteobacteria	Gammaproteobacteria	Enterobacteriales	Enterobacteriaceae	Xenorhabdus	Xenorhabdus nematophila	4587917	4583
640069335	Yersinia enterocolitica enterocolitica sv. 0:08 8081	Proteobacteria	Gammaproteobacteria	Enterobacteriales	Enterobacteriaceae	Yersinia	Yersinia enterocolitica	4683620	4303
650716106	Yersinia enterocolitica palearctica 105.5R(r) (O:9)	Proteobacteria	Gammaproteobacteria	Enterobacteriales	Enterobacteriaceae	Yersinia	Yersinia enterocolitica	4621811	4114
651053079	Yersinia enterocolitica palearctica sv. O:3 bt. 4 Y11	Proteobacteria	Gammaproteobacteria	Enterobacteriales	Enterobacteriaceae	Yersinia	Yersinia enterocolitica	4625880	4551
2565956564	Yersinia enterocolitica LC20	Proteobacteria	Gammaproteobacteria	Enterobacteriales	Enterobacteriaceae	Yersinia	Yersinia enterocolitica	4900014	5596
2623620276	Yersinia enterocolitica enterocolitica sv. 0:08 8081 (re-annotation)	Proteobacteria	Gammaproteobacteria	Enterobacteriales	Enterobacteriaceae	Yersinia	Yersinia enterocolitica	4683620	4438
2597490088	Yersinia kristensenii 33639	Proteobacteria	Gammaproteobacteria	Enterobacteriales	Enterobacteriaceae	Yersinia	Yersinia kristensenii	4442328	4099
637000350	Yersinia pestis Antiqua	Proteobacteria	Gammaproteobacteria	Enterobacteriales	Enterobacteriaceae	Yersinia	Yersinia pestis	4879836	4576
637000351	Yersinia pestis sv. Orientalis CO-92	Proteobacteria	Gammaproteobacteria	Enterobacteriales	Enterobacteriaceae	Yersinia	Yersinia pestis	4829855	4368
637000352	Yersinia pestis sv. Mediaevalis KIM 10	Proteobacteria	Gammaproteobacteria	Enterobacteriales	Enterobacteriaceae	Yersinia	Yersinia pestis	4701745	4445
637000353	Yersinia pestis Nepal516	Proteobacteria	Gammaproteobacteria	Enterobacteriales	Enterobacteriaceae	Yersinia	Yersinia pestis	4646286	4306
637000354	Yersinia pestis bv. Microtus 91001	Proteobacteria	Gammaproteobacteria	Enterobacteriales	Enterobacteriaceae	Yersinia	Yersinia pestis	4803217	4462
640427152	Yersinia pestis Pestoides F	Proteobacteria	Gammaproteobacteria	Enterobacteriales	Enterobacteriaceae	Yersinia	Yersinia pestis	4725862	4359
641228512	Yersinia pestis sv. Antiqua Angola	Proteobacteria	Gammaproteobacteria	Enterobacteriales	Enterobacteriaceae	Yersinia	Yersinia pestis	4687014	4439
646564590	Yersinia pestis bv. Antiqua Z176003	Proteobacteria	Gammaproteobacteria	Enterobacteriales	Enterobacteriaceae	Yersinia	Yersinia pestis	4725788	3780
646862350	Yersinia pestis D106004	Proteobacteria	Gammaproteobacteria	Enterobacteriales	Enterobacteriaceae	Yersinia	Yersinia pestis	4812922	3872
646862351	Yersinia pestis bv. Antiqua D182038	Proteobacteria	Gammaproteobacteria	Enterobacteriales	Enterobacteriaceae	Yersinia	Yersinia pestis	4802263	3879
650377987	Yersinia pestis bv. Medievalis Harbin 35	Proteobacteria	Gammaproteobacteria	Enterobacteriales	Enterobacteriaceae	Yersinia	Yersinia pestis	4709501	4548
2511231145	Yersinia pestis A1122	Proteobacteria	Gammaproteobacteria	Enterobacteriales	Enterobacteriaceae	Yersinia	Yersinia pestis	4658411	4333
2623620750	Yersinia pestis sv. Orientalis CO-92 (re-annotation)	Proteobacteria	Gammaproteobacteria	Enterobacteriales	Enterobacteriaceae	Yersinia	Yersinia pestis	4829855	4451
2623620800	Yersinia pestis sv. Mediaevalis KIM 10 (re-annotation)	Proteobacteria	Gammaproteobacteria	Enterobacteriales	Enterobacteriaceae	Yersinia	Yersinia pestis	4701745	4308

2623620801	Yersinia pestis bv. Microtus 91001 (re-annotation)	Proteobacteria	Gammaproteobacteria	Enterobacteriales	Enterobacteriaceae	Yersinia	Yersinia pestis	4803217	4413
637000355	Yersinia pseudotuberculosis sv. I IP 32953	Proteobacteria	Gammaproteobacteria	Enterobacteriales	Enterobacteriaceae	Yersinia	Yersinia pseudotuberculosis	4840898	4276
640753060	Yersinia pseudotuberculosis sv. O:1b IP 31758	Proteobacteria	Gammaproteobacteria	Enterobacteriales	Enterobacteriaceae	Yersinia	Yersinia pseudotuberculosis	4935125	4529
641522661	Yersinia pseudotuberculosis sv. O3 YPIII	Proteobacteria	Gammaproteobacteria	Enterobacteriales	Enterobacteriaceae	Yersinia	Yersinia pseudotuberculosis	4689441	4298
642555171	Yersinia pseudotuberculosis sv. IB PB1/+	Proteobacteria	Gammaproteobacteria	Enterobacteriales	Enterobacteriaceae	Yersinia	Yersinia pseudotuberculosis	4765431	4342
2597490017	Yersinia pseudotuberculosis 6904	Proteobacteria	Gammaproteobacteria	Enterobacteriales	Enterobacteriaceae	Yersinia	Yersinia pseudotuberculosis	4806594	4322
2623620305	Yersinia pseudotuberculosis sv. O:1b IP 31758 (re-annotation)	Proteobacteria	Gammaproteobacteria	Enterobacteriales	Enterobacteriaceae	Yersinia	Yersinia pseudotuberculosis	4935125	4441
2623620751	Yersinia pseudotuberculosis sv. I IP 32953 (re-annotation)	Proteobacteria	Gammaproteobacteria	Enterobacteriales	Enterobacteriaceae	Yersinia	Yersinia pseudotuberculosis	4840898	4364
2565956581	Yersinia similis 228	Proteobacteria	Gammaproteobacteria	Enterobacteriales	Enterobacteriaceae	Yersinia	Yersinia similis	4964409	4356
637000086	Coxiella burnetii Nine Mile phase I / RSA 493	Proteobacteria	Gammaproteobacteria	Legionellales	Coxiellaceae	Coxiella	Coxiella burnetii	2032674	2175
640753021	Coxiella burnetii Dugway 5J108-111	Proteobacteria	Gammaproteobacteria	Legionellales	Coxiellaceae	Coxiella	Coxiella burnetii	2212937	2257
641228487	Coxiella burnetii RSA 331	Proteobacteria	Gammaproteobacteria	Legionellales	Coxiellaceae	Coxiella	Coxiella burnetii	2053744	2263
643348531	Coxiella burnetii CbuG_Q212	Proteobacteria	Gammaproteobacteria	Legionellales	Coxiellaceae	Coxiella	Coxiella burnetii	2008870	1916
643348532	Coxiella burnetii CbuK_Q154	Proteobacteria	Gammaproteobacteria	Legionellales	Coxiellaceae	Coxiella	Coxiella burnetii	2102380	1992
2623620238	Coxiella burnetii Dugway 5J108-111 (re-annotation)	Proteobacteria	Gammaproteobacteria	Legionellales	Coxiellaceae	Coxiella	Coxiella burnetii	2212937	2158
2623620690	Coxiella burnetii Nine Mile phase I / RSA 493 (re-annotation)	Proteobacteria	Gammaproteobacteria	Legionellales	Coxiellaceae	Coxiella	Coxiella burnetii	2032674	2103
648028038	Legionella longbeachae NSW150	Proteobacteria	Gammaproteobacteria	Legionellales	Legionellaceae	Legionella	Legionella longbeachae	4149158	3536
2545824788	Legionella oakridgensis OR-10, ATCC 33761	Proteobacteria	Gammaproteobacteria	Legionellales	Legionellaceae	Legionella	Legionella oakridgensis	2773209	2990
2558860203	Legionella oakridgensis DSM 21215	Proteobacteria	Gammaproteobacteria	Legionellales	Legionellaceae	Legionella	Legionella oakridgensis	2773209	2983
637000146	Legionella pneumophila sv. 1 Lens	Proteobacteria	Gammaproteobacteria	Legionellales	Legionellaceae	Legionella	Legionella pneumophila	3405519	3062
637000147	Legionella pneumophila Paris	Proteobacteria	Gammaproteobacteria	Legionellales	Legionellaceae	Legionella	Legionella pneumophila	3635495	3280
637000148	Legionella pneumophila pneumophila Philadelphia-1	Proteobacteria	Gammaproteobacteria	Legionellales	Legionellaceae	Legionella	Legionella pneumophila	3397754	3003
640427119	Legionella pneumophila Corby	Proteobacteria	Gammaproteobacteria	Legionellales	Legionellaceae	Legionella	Legionella pneumophila	3576470	3266
646564542	Legionella pneumophila sv. 1 2300/99 Alcoy	Proteobacteria	Gammaproteobacteria	Legionellales	Legionellaceae	Legionella	Legionella pneumophila	3516334	3242
2511231146	Legionella pneumophila pneumonophila 570-CO-H, ATCC 43290	Proteobacteria	Gammaproteobacteria	Legionellales	Legionellaceae	Legionella	Legionella pneumophila	3359001	2996
2540341167	Legionella pneumophila pneumophila HL06041035	Proteobacteria	Gammaproteobacteria	Legionellales	Legionellaceae	Legionella	Legionella pneumophila	3492535	3184
2554235362	Legionella pneumophila pneumophila Thunder Bay	Proteobacteria	Gammaproteobacteria	Legionellales	Legionellaceae	Legionella	Legionella pneumophila	3455167	3043
2558860200	Legionella pneumophila pneumophila Hextuple_3a	Proteobacteria	Gammaproteobacteria	Legionellales	Legionellaceae	Legionella	Legionella pneumophila	2682626	2468
2558860201	Legionella pneumophila pneumophila Hextuple_2q	Proteobacteria	Gammaproteobacteria	Legionellales	Legionellaceae	Legionella	Legionella pneumophila	2682626	2468
2561511154	Legionella pneumophila pneumophila Lorraine	Proteobacteria	Gammaproteobacteria	Legionellales	Legionellaceae	Legionella	Legionella pneumophila	3617686	3327
2561511186	Legionella pneumophila pneumophila LPE509	Proteobacteria	Gammaproteobacteria	Legionellales	Legionellaceae	Legionella	Legionella pneumophila	3507714	3383
2623620240	Legionella pneumophila Corby (re-annotation)	Proteobacteria	Gammaproteobacteria	Legionellales	Legionellaceae	Legionella	Legionella pneumophila	3576470	3255
2623620710	Legionella pneumophila Paris (re-annotation)	Proteobacteria	Gammaproteobacteria	Legionellales	Legionellaceae	Legionella	Legionella pneumophila	3635495	3305
2623620934	Legionella pneumophila sv. 1 Lens (re-annotation)	Proteobacteria	Gammaproteobacteria	Legionellales	Legionellaceae	Legionella	Legionella pneumophila	3405519	3070
2623620935	Legionella pneumophila pneumophila Philadelphia-1 (re-annotation)	Proteobacteria	Gammaproteobacteria	Legionellales	Legionellaceae	Legionella	Legionella pneumophila	3397754	3087
2509601034	Frateuria aurantia Kondo 67, DSM 6220	Proteobacteria	Gammaproteobacteria	Lysobacterales	Lysobacteraceae	Frateuria	Frateuria aurantia	3603458	3288
637000166	Methylococcus capsulatus Bath	Proteobacteria	Gammaproteobacteria	Methylococcales	Methylococcaceae	Methylococcus	Methylococcus capsulatus	3304561	3061
2623620716	Methylococcus capsulatus Bath (re-annotation)	Proteobacteria	Gammaproteobacteria	Methylococcales	Methylococcaceae	Methylococcus	Methylococcus capsulatus	3304561	3112
2540341096	Methylomicrobium alcaliphilum 20Z	Proteobacteria	Gammaproteobacteria	Methylococcales	Methylococcaceae	Methylomicrobium	Methylomicrobium alcaliphilum	4668296	4083
2504756059	Methylomonas methanica MC09	Proteobacteria	Gammaproteobacteria	Methylococcales	Methylococcaceae	Methylomonas	Methylomonas methanica	5051681	4664
637000004	Alcanivorax borkumensis SK2	Proteobacteria	Gammaproteobacteria	Oceanospirillales	Alcanivoracaceae	Alcanivorax	Alcanivorax borkumensis	3120143	2817
2623620884	Alcanivorax borkumensis SK2 (re-annotation)	Proteobacteria	Gammaproteobacteria	Oceanospirillales	Alcanivoracaceae	Alcanivorax	Alcanivorax borkumensis	3120143	2880
2521172691	Alcanivorax dieselolei B5	Proteobacteria	Gammaproteobacteria	Oceanospirillales	Alcanivoracaceae	Alcanivorax	Alcanivorax dieselolei	4928223	4470
644736377	Kangiella koreensis SW-125, DSM 16069	Proteobacteria	Gammaproteobacteria	Oceanospirillales	Alcanivoracaceae	Kangiella	Kangiella koreensis	2852073	2694
637000128	Hahella chejuensis KCTC 2396	Proteobacteria	Gammaproteobacteria	Oceanospirillales	Hahellaceae	Hahella	Hahella chejuensis	7215267	6875
2623620924	Hahella chejuensis KCTC 2396 (re-annotation)	Proteobacteria	Gammaproteobacteria	Oceanospirillales	Hahellaceae	Hahella	Hahella chejuensis	7215267	6480
637000075	Chromohalobacter salexigens 1H11, DSM 3043	Proteobacteria	Gammaproteobacteria	Oceanospirillales	Halomonadaceae	Chromohalobacter	Chromohalobacter salexigens	3696649	3412
2576861481	Halomonas campaniensis LS21	Proteobacteria	Gammaproteobacteria	Oceanospirillales	Halomonadaceae	Halomonas	Halomonas campaniensis	4074048	3665
648028030	Halomonas elongata DSM 2581	Proteobacteria	Gammaproteobacteria	Oceanospirillales	Halomonadaceae	Halomonas	Halomonas elongata	4061296	3556
2506210002	Marinomonas mediterranea MMB-1, ATCC 700492	Proteobacteria	Gammaproteobacteria	Oceanospirillales	Oceanospirillaceae	Marinomonas	Marinomonas mediterranea	4684316	4326
2506210003	Marinomonas posidonica IVIA-Po-181	Proteobacteria	Gammaproteobacteria	Oceanospirillales	Oceanospirillaceae	Marinomonas	Marinomonas posidonica	3899940	3651
640753033	Marinomonas sp. MWYL1	Proteobacteria	Gammaproteobacteria	Oceanospirillales	Oceanospirillaceae	Marinomonas	Marinomonas sp. MWYL1	5100344	4609
2563366573	Thalassolituus oleivorans R6-15	Proteobacteria	Gammaproteobacteria	Oceanospirillales	Oceanospirillaceae	Thalassolituus	Thalassolituus oleivorans	3764053	3489
2597489935	Thalassolituus oleivorans MIL-1	Proteobacteria	Gammaproteobacteria	Oceanospirillales	Oceanospirillaceae	Thalassolituus	Thalassolituus oleivorans	3920328	3638
2574179809	Gilliamella apicola	Proteobacteria	Gammaproteobacteria	Orbales	Orbaceae	Gilliamella	Gilliamella apicola	3139412	2869

640069301	Acinetobacter baumannii ATCC 17978	Proteobacteria	Gammaproteobacteria	Pseudomonadales	Moraxellaceae	Acinetobacter	Acinetobacter baumannii	4001457	3464
641522602	Acinetobacter baumannii AYE	Proteobacteria	Gammaproteobacteria	Pseudomonadales	Moraxellaceae	Acinetobacter	Acinetobacter baumannii	4048735	3813
641522603	Acinetobacter baumannii SDF	Proteobacteria	Gammaproteobacteria	Pseudomonadales	Moraxellaceae	Acinetobacter	Acinetobacter baumannii	3477996	3062
642555102	Acinetobacter baumannii ACICU	Proteobacteria	Gammaproteobacteria	Pseudomonadales	Moraxellaceae	Acinetobacter	Acinetobacter baumannii	3996761	3841
643348502	Acinetobacter baumannii AB0057	Proteobacteria	Gammaproteobacteria	Pseudomonadales	Moraxellaceae	Acinetobacter	Acinetobacter baumannii	4059242	3892
643348503	Acinetobacter baumannii AB307-0294	Proteobacteria	Gammaproteobacteria	Pseudomonadales	Moraxellaceae	Acinetobacter	Acinetobacter baumannii	3760981	3542
650377901	Acinetobacter baumannii 1656-2	Proteobacteria	Gammaproteobacteria	Pseudomonadales	Moraxellaceae	Acinetobacter	Acinetobacter baumannii	4023106	3913
650377902	Acinetobacter baumannii TCDC-AB0715	Proteobacteria	Gammaproteobacteria	Pseudomonadales	Moraxellaceae	Acinetobacter	Acinetobacter baumannii	4210417	4003
2511231155	Acinetobacter baumannii MDR-ZJ06	Proteobacteria	Gammaproteobacteria	Pseudomonadales	Moraxellaceae	Acinetobacter	Acinetobacter baumannii	4011434	3975
2519103115	Acinetobacter baumannii TYTH-1	Proteobacteria	Gammaproteobacteria	Pseudomonadales	Moraxellaceae	Acinetobacter	Acinetobacter baumannii	3957368	3771
2519899559	Acinetobacter baumannii AC12	Proteobacteria	Gammaproteobacteria	Pseudomonadales	Moraxellaceae	Acinetobacter	Acinetobacter baumannii	3848312	3688
2519899736	Acinetobacter baumannii AC30	Proteobacteria	Gammaproteobacteria	Pseudomonadales	Moraxellaceae	Acinetobacter	Acinetobacter baumannii	3922859	3789
2554235346	Acinetobacter baumannii BJAB07104	Proteobacteria	Gammaproteobacteria	Pseudomonadales	Moraxellaceae	Acinetobacter	Acinetobacter baumannii	4042229	3953
2554235348	Acinetobacter baumannii BJAB0868	Proteobacteria	Gammaproteobacteria	Pseudomonadales	Moraxellaceae	Acinetobacter	Acinetobacter baumannii	4005822	3912
2558309051	Acinetobacter baumannii ZW85-1	Proteobacteria	Gammaproteobacteria	Pseudomonadales	Moraxellaceae	Acinetobacter	Acinetobacter baumannii	3876878	3671
2558860252	Acinetobacter baumannii AB07	Proteobacteria	Gammaproteobacteria	Pseudomonadales	Moraxellaceae	Acinetobacter	Acinetobacter baumannii	4242611	3955
2561511161	Acinetobacter baumannii BJAB0715	Proteobacteria	Gammaproteobacteria	Pseudomonadales	Moraxellaceae	Acinetobacter	Acinetobacter baumannii	4053889	3999
2563366605	Acinetobacter baumannii D1279779	Proteobacteria	Gammaproteobacteria	Pseudomonadales	Moraxellaceae	Acinetobacter	Acinetobacter baumannii	3711701	3484
2565956548	Acinetobacter baumannii MDR-TJ	Proteobacteria	Gammaproteobacteria	Pseudomonadales	Moraxellaceae	Acinetobacter	Acinetobacter baumannii	4153407	4032
2582580753	Acinetobacter baumannii AC29	Proteobacteria	Gammaproteobacteria	Pseudomonadales	Moraxellaceae	Acinetobacter	Acinetobacter baumannii	3935134	3747
2597490178	Acinetobacter baumannii AB30	Proteobacteria	Gammaproteobacteria	Pseudomonadales	Moraxellaceae	Acinetobacter	Acinetobacter baumannii	4335793	4390
2597490184	Acinetobacter baumannii AB31	Proteobacteria	Gammaproteobacteria	Pseudomonadales	Moraxellaceae	Acinetobacter	Acinetobacter baumannii	3803317	3625
2623620317	Acinetobacter baumannii ATCC 17978 (re-annotation)	Proteobacteria	Gammaproteobacteria	Pseudomonadales	Moraxellaceae	Acinetobacter	Acinetobacter baumannii	4001457	4071
650377903	Acinetobacter calcoaceticus PHEA-2	Proteobacteria	Gammaproteobacteria	Pseudomonadales	Moraxellaceae	Acinetobacter	Acinetobacter calcoaceticus	3862530	3674
648028004	Acinetobacter oleivorans DR1	Proteobacteria	Gammaproteobacteria	Pseudomonadales	Moraxellaceae	Acinetobacter	Acinetobacter oleivorans	4152543	3963
637000002	Acinetobacter baylyi ADP1	Proteobacteria	Gammaproteobacteria	Pseudomonadales	Moraxellaceae	Acinetobacter	Acinetobacter sp. ADP1	3598621	3433
2623620882	Acinetobacter baylyi ADP1 (re-annotation)	Proteobacteria	Gammaproteobacteria	Pseudomonadales	Moraxellaceae	Acinetobacter	Acinetobacter sp. ADP1	3598621	3373
646564552	Moraxella catarrhalis RH4	Proteobacteria	Gammaproteobacteria	Pseudomonadales	Moraxellaceae	Moraxella	Moraxella catarrhalis	1863286	1948
2597490148	Moraxella catarrhalis 25240	Proteobacteria	Gammaproteobacteria	Pseudomonadales	Moraxellaceae	Moraxella	Moraxella catarrhalis	1941566	1794
637000226	Psychrobacter arcticus 273-4	Proteobacteria	Gammaproteobacteria	Pseudomonadales	Moraxellaceae	Psychrobacter	Psychrobacter arcticus	2650701	2215
637000227	Psychrobacter cryohalolentis K5	Proteobacteria	Gammaproteobacteria	Pseudomonadales	Moraxellaceae	Psychrobacter	Psychrobacter cryohalolentis	3101097	2582
2554235460	Psychrobacter sp. G	Proteobacteria	Gammaproteobacteria	Pseudomonadales	Moraxellaceae	Psychrobacter	Psychrobacter sp. G	3113999	2682
640427134	Psychrobacter sp. PRwf-1	Proteobacteria	Gammaproteobacteria	Pseudomonadales	Moraxellaceae	Psychrobacter	Psychrobacter sp. PRwf-1	2995049	2481
643692004	Azotobacter vinelandii DJ, ATCC BAA-1303	Proteobacteria	Gammaproteobacteria	Pseudomonadales	Pseudomonadaceae	Azotobacter	Azotobacter vinelandii	5365318	5133
2541047084	Azotobacter vinelandii CA	Proteobacteria	Gammaproteobacteria	Pseudomonadales	Pseudomonadaceae	Azotobacter	Azotobacter vinelandii	5366370	5212
2541047085	Azotobacter vinelandii CA6	Proteobacteria	Gammaproteobacteria	Pseudomonadales	Pseudomonadaceae	Azotobacter	Azotobacter vinelandii	5323210	5171
642555119	Cellvibrio japonicus Ueda107	Proteobacteria	Gammaproteobacteria	Pseudomonadales	Pseudomonadaceae	Cellvibrio	Cellvibrio japonicus	4576573	3811
637000218	*Pseudomonas aeruginosa* PAO1	Proteobacteria	Gammaproteobacteria	Pseudomonadales	Pseudomonadaceae	Pseudomonas	*Pseudomonas aeruginosa*	6264404	5671
639279310	*Pseudomonas aeruginosa* UCBPP-PA14	Proteobacteria	Gammaproteobacteria	Pseudomonadales	Pseudomonadaceae	Pseudomonas	*Pseudomonas aeruginosa*	6537648	5994
640753042	*Pseudomonas aeruginosa* sv. O12 PA7	Proteobacteria	Gammaproteobacteria	Pseudomonadales	Pseudomonadaceae	Pseudomonas	*Pseudomonas aeruginosa*	6588339	6396
643348568	*Pseudomonas aeruginosa* LESB58	Proteobacteria	Gammaproteobacteria	Pseudomonadales	Pseudomonadaceae	Pseudomonas	*Pseudomonas aeruginosa*	6601757	6026
2511231165	*Pseudomonas aeruginosa* NCGM2.S1	Proteobacteria	Gammaproteobacteria	Pseudomonadales	Pseudomonadaceae	Pseudomonas	*Pseudomonas aeruginosa*	6764661	6358
2511231183	*Pseudomonas aeruginosa* M18	Proteobacteria	Gammaproteobacteria	Pseudomonadales	Pseudomonadaceae	Pseudomonas	*Pseudomonas aeruginosa*	6327754	5769
2519103093	*Pseudomonas aeruginosa* DK2	Proteobacteria	Gammaproteobacteria	Pseudomonadales	Pseudomonadaceae	Pseudomonas	*Pseudomonas aeruginosa*	6402658	5960
2540341118	*Pseudomonas aeruginosa* B136-33	Proteobacteria	Gammaproteobacteria	Pseudomonadales	Pseudomonadaceae	Pseudomonas	*Pseudomonas aeruginosa*	6421010	5904
2558309053	*Pseudomonas aeruginosa* PA1R	Proteobacteria	Gammaproteobacteria	Pseudomonadales	Pseudomonadaceae	Pseudomonas	*Pseudomonas aeruginosa*	6309305	5689
2558309066	*Pseudomonas aeruginosa* PA1	Proteobacteria	Gammaproteobacteria	Pseudomonadales	Pseudomonadaceae	Pseudomonas	*Pseudomonas aeruginosa*	6528877	5876
2558309079	*Pseudomonas aeruginosa* SCV20265	Proteobacteria	Gammaproteobacteria	Pseudomonadales	Pseudomonadaceae	Pseudomonas	*Pseudomonas aeruginosa*	6725183	6380
2558309081	*Pseudomonas aeruginosa* LES431	Proteobacteria	Gammaproteobacteria	Pseudomonadales	Pseudomonadaceae	Pseudomonas	*Pseudomonas aeruginosa*	6550070	6091
2558309082	*Pseudomonas aeruginosa* MTB-1	Proteobacteria	Gammaproteobacteria	Pseudomonadales	Pseudomonadaceae	Pseudomonas	*Pseudomonas aeruginosa*	6580038	6186
2558860267	*Pseudomonas aeruginosa* YL84	Proteobacteria	Gammaproteobacteria	Pseudomonadales	Pseudomonadaceae	Pseudomonas	*Pseudomonas aeruginosa*	6433441	5938
2563366590	*Pseudomonas aeruginosa* RP73	Proteobacteria	Gammaproteobacteria	Pseudomonadales	Pseudomonadaceae	Pseudomonas	*Pseudomonas aeruginosa*	6342034	5864
2565956573	*Pseudomonas aeruginosa* LES400	Proteobacteria	Gammaproteobacteria	Pseudomonadales	Pseudomonadaceae	Pseudomonas	*Pseudomonas aeruginosa*	6591121	6157
2565956574	*Pseudomonas aeruginosa* LESB65	Proteobacteria	Gammaproteobacteria	Pseudomonadales	Pseudomonadaceae	Pseudomonas	*Pseudomonas aeruginosa*	6527005	6075
2565956575	*Pseudomonas aeruginosa* LESlike1	Proteobacteria	Gammaproteobacteria	Pseudomonadales	Pseudomonadaceae	Pseudomonas	*Pseudomonas aeruginosa*	6509070	6054

2565956576	*Pseudomonas aeruginosa* LESlike4	Proteobacteria	Gammaproteobacteria	Pseudomonadales	Pseudomonadaceae	Pseudomonas	*Pseudomonas aeruginosa*	6524053	6066
2565956577	*Pseudomonas aeruginosa* LESlike5	Proteobacteria	Gammaproteobacteria	Pseudomonadales	Pseudomonadaceae	Pseudomonas	*Pseudomonas aeruginosa*	6543350	6077
2565956578	*Pseudomonas aeruginosa* LESlike7	Proteobacteria	Gammaproteobacteria	Pseudomonadales	Pseudomonadaceae	Pseudomonas	*Pseudomonas aeruginosa*	6467914	5994
2579778693	*Pseudomonas aeruginosa* PA96	Proteobacteria	Gammaproteobacteria	Pseudomonadales	Pseudomonadaceae	Pseudomonas	*Pseudomonas aeruginosa*	6444091	5925
2585427615	*Pseudomonas aeruginosa* PAO1H2O	Proteobacteria	Gammaproteobacteria	Pseudomonadales	Pseudomonadaceae	Pseudomonas	*Pseudomonas aeruginosa*	6264404	5628
2597489949	*Pseudomonas aeruginosa* PAO581	Proteobacteria	Gammaproteobacteria	Pseudomonadales	Pseudomonadaceae	Pseudomonas	*Pseudomonas aeruginosa*	6043974	5662
2597489950	*Pseudomonas aeruginosa* PAO1-VE13	Proteobacteria	Gammaproteobacteria	Pseudomonadales	Pseudomonadaceae	Pseudomonas	*Pseudomonas aeruginosa*	6265484	5800
2597489951	*Pseudomonas aeruginosa* PAO1-VE2	Proteobacteria	Gammaproteobacteria	Pseudomonadales	Pseudomonadaceae	Pseudomonas	*Pseudomonas aeruginosa*	6265484	5799
2597489954	*Pseudomonas aeruginosa* c7447m	Proteobacteria	Gammaproteobacteria	Pseudomonadales	Pseudomonadaceae	Pseudomonas	*Pseudomonas aeruginosa*	6262205	5844
2623620225	*Pseudomonas aeruginosa* sv. O12 PA7 (re-annotation)	Proteobacteria	Gammaproteobacteria	Pseudomonadales	Pseudomonadaceae	Pseudomonas	*Pseudomonas aeruginosa*	6588339	6164
2623620735	*Pseudomonas aeruginosa* UCBPP-PA14 (re-annotation)	Proteobacteria	Gammaproteobacteria	Pseudomonadales	Pseudomonadaceae	Pseudomonas	*Pseudomonas aeruginosa*	6537648	6052
2623620964	*Pseudomonas aeruginosa* PAO1 (re-annotation)	Proteobacteria	Gammaproteobacteria	Pseudomonadales	Pseudomonadaceae	Pseudomonas	*Pseudomonas aeruginosa*	6264404	5814
2597490176	Pseudomonas alkylphenolia KL28	Proteobacteria	Gammaproteobacteria	Pseudomonadales	Pseudomonadaceae	Pseudomonas	Pseudomonas alkylphenolia	5764622	5426
650716074	Pseudomonas brassicacearum brassicacearum NFM421	Proteobacteria	Gammaproteobacteria	Pseudomonadales	Pseudomonadaceae	Pseudomonas	Pseudomonas brassicacearum	6843248	6176
2565956580	Pseudomonas brassicacearum DF41	Proteobacteria	Gammaproteobacteria	Pseudomonadales	Pseudomonadaceae	Pseudomonas	Pseudomonas brassicacearum	6652396	5773
2585427645	Pseudomonas chlororaphis PA23	Proteobacteria	Gammaproteobacteria	Pseudomonadales	Pseudomonadaceae	Pseudomonas	Pseudomonas chlororaphis	7122173	6286
2579778861	Pseudomonas cichorii JBC1	Proteobacteria	Gammaproteobacteria	Pseudomonadales	Pseudomonadaceae	Pseudomonas	Pseudomonas cichorii	5986012	5258
2540341139	Pseudomonas denitrificans ATCC 13867	Proteobacteria	Gammaproteobacteria	Pseudomonadales	Pseudomonadaceae	Pseudomonas	Pseudomonas denitrificans	5696307	5135
637000219	Pseudomonas entomophila L48	Proteobacteria	Gammaproteobacteria	Pseudomonadales	Pseudomonadaceae	Pseudomonas	Pseudomonas entomophila	5888780	5293
2623620849	Pseudomonas entomophila L48 (re-annotation)	Proteobacteria	Gammaproteobacteria	Pseudomonadales	Pseudomonadaceae	Pseudomonas	Pseudomonas entomophila	5888780	5287
637000221	Pseudomonas fluorescens Pf0-1	Proteobacteria	Gammaproteobacteria	Pseudomonadales	Pseudomonadaceae	Pseudomonas	Pseudomonas fluorescens	6438405	5857
649633086	Pseudomonas fluorescens SBW25	Proteobacteria	Gammaproteobacteria	Pseudomonadales	Pseudomonadaceae	Pseudomonas	Pseudomonas fluorescens	7147633	6492
2503538034	Pseudomonas fluorescens R124	Proteobacteria	Gammaproteobacteria	Pseudomonadales	Pseudomonadaceae	Pseudomonas	Pseudomonas fluorescens	6299732	5572
2511231156	Pseudomonas fluorescens F113	Proteobacteria	Gammaproteobacteria	Pseudomonadales	Pseudomonadaceae	Pseudomonas	Pseudomonas fluorescens	6845832	5952
2561511156	Pseudomonas fluorescens A506	Proteobacteria	Gammaproteobacteria	Pseudomonadales	Pseudomonadaceae	Pseudomonas	Pseudomonas fluorescens	6019547	5493
2505679082	Pseudomonas fulva 12-X	Proteobacteria	Gammaproteobacteria	Pseudomonadales	Pseudomonadaceae	Pseudomonas	Pseudomonas fulva	4920769	4574
2579778852	Pseudomonas knackmussii B13	Proteobacteria	Gammaproteobacteria	Pseudomonadales	Pseudomonadaceae	Pseudomonas	Pseudomonas knackmussii	6162905	5823
640427131	Pseudomonas mendocina ymp	Proteobacteria	Gammaproteobacteria	Pseudomonadales	Pseudomonadaceae	Pseudomonas	Pseudomonas mendocina	5072807	4730
650716075	Pseudomonas mendocina NK-01	Proteobacteria	Gammaproteobacteria	Pseudomonadales	Pseudomonadaceae	Pseudomonas	Pseudomonas mendocina	5434353	5035
2516653025	Pseudomonas monteilii SB3078 (v1.0)	Proteobacteria	Gammaproteobacteria	Pseudomonadales	Pseudomonadaceae	Pseudomonas	Pseudomonas monteilii	5866715	5525
2516653026	Pseudomonas monteilii SB3101 (v1.0)	Proteobacteria	Gammaproteobacteria	Pseudomonadales	Pseudomonadaceae	Pseudomonas	Pseudomonas monteilii	5817558	5452
2597489942	Pseudomonas moraviensis R28-S	Proteobacteria	Gammaproteobacteria	Pseudomonadales	Pseudomonadaceae	Pseudomonas	Pseudomonas moraviensis	6308216	5804
2540341095	Pseudomonas poae RE*1-1-14	Proteobacteria	Gammaproteobacteria	Pseudomonadales	Pseudomonadaceae	Pseudomonas	Pseudomonas poae	5512241	4877
637000220	Pseudomonas protegens Pf-5	Proteobacteria	Gammaproteobacteria	Pseudomonadales	Pseudomonadaceae	Pseudomonas	Pseudomonas protegens	7074893	6257
2554235341	Pseudomonas fluorescens CHA0	Proteobacteria	Gammaproteobacteria	Pseudomonadales	Pseudomonadaceae	Pseudomonas	Pseudomonas protegens	6867980	6199
2623620736	Pseudomonas protegens Pf-5 (re-annotation)	Proteobacteria	Gammaproteobacteria	Pseudomonadales	Pseudomonadaceae	Pseudomonas	Pseudomonas protegens	7074893	6408
637000222	Pseudomonas putida KT2440	Proteobacteria	Gammaproteobacteria	Pseudomonadales	Pseudomonadaceae	Pseudomonas	Pseudomonas putida	6181863	5481
640427132	Pseudomonas putida F1	Proteobacteria	Gammaproteobacteria	Pseudomonadales	Pseudomonadaceae	Pseudomonas	Pseudomonas putida	5959964	5423
641522645	Pseudomonas putida GB-1	Proteobacteria	Gammaproteobacteria	Pseudomonadales	Pseudomonadaceae	Pseudomonas	Pseudomonas putida	6078430	5515
641522646	Pseudomonas putida W619	Proteobacteria	Gammaproteobacteria	Pseudomonadales	Pseudomonadaceae	Pseudomonas	Pseudomonas putida	5774330	5292
650377963	Pseudomonas putida BIRD-1	Proteobacteria	Gammaproteobacteria	Pseudomonadales	Pseudomonadaceae	Pseudomonas	Pseudomonas putida	5731541	5046
650716076	Pseudomonas putida S16	Proteobacteria	Gammaproteobacteria	Pseudomonadales	Pseudomonadaceae	Pseudomonas	Pseudomonas putida	5984790	5307
2518645549	Pseudomonas putida DOT-T1E	Proteobacteria	Gammaproteobacteria	Pseudomonadales	Pseudomonadaceae	Pseudomonas	Pseudomonas putida	6260702	5802
2518645571	Pseudomonas putida ND6	Proteobacteria	Gammaproteobacteria	Pseudomonadales	Pseudomonadaceae	Pseudomonas	Pseudomonas putida	6304310	6484
2521172712	Pseudomonas putida PC9	Proteobacteria	Gammaproteobacteria	Pseudomonadales	Pseudomonadaceae	Pseudomonas	Pseudomonas putida	5956110	5478
2554235365	Pseudomonas putida NBRC 14164	Proteobacteria	Gammaproteobacteria	Pseudomonadales	Pseudomonadaceae	Pseudomonas	Pseudomonas putida	6156701	5544
2563366586	Pseudomonas putida H8234	Proteobacteria	Gammaproteobacteria	Pseudomonadales	Pseudomonadaceae	Pseudomonas	Pseudomonas putida	6870827	6482
2579778942	Pseudomonas putida DLL-E4	Proteobacteria	Gammaproteobacteria	Pseudomonadales	Pseudomonadaceae	Pseudomonas	Pseudomonas putida	6484062	5946
2623620737	Pseudomonas putida KT2440 (re-annotation)	Proteobacteria	Gammaproteobacteria	Pseudomonadales	Pseudomonadaceae	Pseudomonas	Pseudomonas putida	6181863	5761
2561511189	Pseudomonas resinovorans NBRC 106553	Proteobacteria	Gammaproteobacteria	Pseudomonadales	Pseudomonadaceae	Pseudomonas	Pseudomonas resinovorans	6484828	5961
637000223	Pseudomonas syringae pv. phaseolicola 1448A	Proteobacteria	Gammaproteobacteria	Pseudomonadales	Pseudomonadaceae	Pseudomonas	Pseudomonas savastanoi	6112448	5436
2623620738	Pseudomonas syringae pv. phaseolicola 1448A (re-annotation)	Proteobacteria	Gammaproteobacteria	Pseudomonadales	Pseudomonadaceae	Pseudomonas	Pseudomonas savastanoi	6112448	5702
2585427642	Pseudomonas simiae WCS417	Proteobacteria	Gammaproteobacteria	Pseudomonadales	Pseudomonadaceae	Pseudomonas	Pseudomonas simiae	6169071	5610
2517572175	Pseudomonas sp. FGI182	Proteobacteria	Gammaproteobacteria	Pseudomonadales	Pseudomonadaceae	Pseudomonas	Pseudomonas sp. FGI182	5891312	5365
2558309084	Pseudomonas sp. TKP	Proteobacteria	Gammaproteobacteria	Pseudomonadales	Pseudomonadaceae	Pseudomonas	Pseudomonas sp. TKP	7012672	6413

2585427659	Pseudomonas fluorescens UK4	Proteobacteria	Gammaproteobacteria	Pseudomonadales	Pseudomonadaceae	Pseudomonas	Pseudomonas sp. UK4	6064456	5299
2519899536	Pseudomonas sp. UW4	Proteobacteria	Gammaproteobacteria	Pseudomonadales	Pseudomonadaceae	Pseudomonas	Pseudomonas sp. UW4	6183388	5517
2558309062	Pseudomonas sp. VLB120	Proteobacteria	Gammaproteobacteria	Pseudomonadales	Pseudomonadaceae	Pseudomonas	Pseudomonas sp. VLB120	5966222	5580
2585427641	Pseudomonas sp. WCS374	Proteobacteria	Gammaproteobacteria	Pseudomonadales	Pseudomonadaceae	Pseudomonas	Pseudomonas sp. WCS374	6085054	5469
640427133	Pseudomonas stutzeri A1501	Proteobacteria	Gammaproteobacteria	Pseudomonadales	Pseudomonadaceae	Pseudomonas	Pseudomonas stutzeri	4567418	4237
650716077	Pseudomonas stutzeri Lautrop AB 201, ATCC 17588	Proteobacteria	Gammaproteobacteria	Pseudomonadales	Pseudomonadaceae	Pseudomonas	Pseudomonas stutzeri	4547930	4287
651053060	Pseudomonas stutzeri CMT.A.9, DSM 4166	Proteobacteria	Gammaproteobacteria	Pseudomonadales	Pseudomonadaceae	Pseudomonas	Pseudomonas stutzeri	4689946	4374
2507149015	Pseudomonas stutzeri RCH2	Proteobacteria	Gammaproteobacteria	Pseudomonadales	Pseudomonadaceae	Pseudomonas	Pseudomonas stutzeri	4600489	4412
2518645567	Pseudomonas stutzeri AN10, CCUG 29243	Proteobacteria	Gammaproteobacteria	Pseudomonadales	Pseudomonadaceae	Pseudomonas	Pseudomonas stutzeri	4709064	4374
2519899778	Pseudomonas stutzeri JM3000, DSM 10701	Proteobacteria	Gammaproteobacteria	Pseudomonadales	Pseudomonadaceae	Pseudomonas	Pseudomonas stutzeri	4174118	3888
2565956579	Pseudomonas stutzeri 28a24	Proteobacteria	Gammaproteobacteria	Pseudomonadales	Pseudomonadaceae	Pseudomonas	Pseudomonas stutzeri	4731359	4286
2574179801	Pseudomonas stutzeri 19SMN4	Proteobacteria	Gammaproteobacteria	Pseudomonadales	Pseudomonadaceae	Pseudomonas	Pseudomonas stutzeri	4833395	4485
2623620303	Pseudomonas stutzeri A1501 (re-annotation)	Proteobacteria	Gammaproteobacteria	Pseudomonadales	Pseudomonadaceae	Pseudomonas	Pseudomonas stutzeri	4567418	4297
637000224	Pseudomonas syringae pv. syringae B728a	Proteobacteria	Gammaproteobacteria	Pseudomonadales	Pseudomonadaceae	Pseudomonas	Pseudomonas syringae	6093698	5245
2506783024	Pseudomonas syringae CC1557 (CC1557)	Proteobacteria	Gammaproteobacteria	Pseudomonadales	Pseudomonadaceae	Pseudomonas	Pseudomonas syringae	5714345	5792
2523533564	Pseudomonas syringae pv. syringae B64	Proteobacteria	Gammaproteobacteria	Pseudomonadales	Pseudomonadaceae	Pseudomonas	Pseudomonas syringae	5930035	5021
2597489895	Pseudomonas syringae pv. actinidiae ICMP 18884	Proteobacteria	Gammaproteobacteria	Pseudomonadales	Pseudomonadaceae	Pseudomonas	Pseudomonas syringae	6582853	6084
2597489907	Pseudomonas syringae pv. actinidia ICMP 9617	Proteobacteria	Gammaproteobacteria	Pseudomonadales	Pseudomonadaceae	Pseudomonas	Pseudomonas syringae	6497695	5462
2617270755	Pseudomonas syringae pv. syringae B301D	Proteobacteria	Gammaproteobacteria	Pseudomonadales	Pseudomonadaceae	Pseudomonas	Pseudomonas syringae	6094819	5344
2617270756	Pseudomonas syringae pv. syringae HS191	Proteobacteria	Gammaproteobacteria	Pseudomonadales	Pseudomonadaceae	Pseudomonas	Pseudomonas syringae	5950211	5233
2508501074	Pseudomonas syringae pv. tomato DC3000 (DC3000 gold standard)	Proteobacteria	Gammaproteobacteria	Pseudomonadales	Pseudomonadaceae	Pseudomonas	Pseudomonas syringae group genomosp. 3	6538260	5955
2518645558	Francisella noatunensis orientalis Toba 04	Proteobacteria	Gammaproteobacteria	Thiotrichales	Francisellaceae	Francisella	Francisella noatunensis	1847202	1875
2558309050	Francisella noatunensis LADL--07-285A	Proteobacteria	Gammaproteobacteria	Thiotrichales	Francisellaceae	Francisella	Francisella noatunensis	1858987	1991
641522628	Francisella philomiragia philomiragia ATCC 25017	Proteobacteria	Gammaproteobacteria	Thiotrichales	Francisellaceae	Francisella	Francisella philomiragia	2049711	1964
2516143106	Francisella sp. FSC1006	Proteobacteria	Gammaproteobacteria	Thiotrichales	Francisellaceae	Francisella	Francisella sp. FSC1006	2056490	2041
650716037	Francisella sp. TX077308	Proteobacteria	Gammaproteobacteria	Thiotrichales	Francisellaceae	Francisella	Francisella sp. TX077308	2035931	2025
637000111	Francisella tularensis holarctica LVS	Proteobacteria	Gammaproteobacteria	Thiotrichales	Francisellaceae	Francisella	Francisella tularensis	1895994	2019
637000112	Francisella tularensis holarctica OSU18	Proteobacteria	Gammaproteobacteria	Thiotrichales	Francisellaceae	Francisella	Francisella tularensis	1895727	1922
637000113	Francisella tularensis tularensis FSC 198	Proteobacteria	Gammaproteobacteria	Thiotrichales	Francisellaceae	Francisella	Francisella tularensis	1892616	1841
637000114	Francisella tularensis tularensis CHU S4	Proteobacteria	Gammaproteobacteria	Thiotrichales	Francisellaceae	Francisella	Francisella tularensis	1892819	1842
639633024	Francisella tularensis novicida U112	Proteobacteria	Gammaproteobacteria	Thiotrichales	Francisellaceae	Francisella	Francisella tularensis	1910031	1785
640069311	Francisella tularensis tularensis A.II, Wyoming, WY96-3418	Proteobacteria	Gammaproteobacteria	Thiotrichales	Francisellaceae	Francisella	Francisella tularensis	1898476	1686
640753028	Francisella tularensis holarctica FTA (FTNF002-00)	Proteobacteria	Gammaproteobacteria	Thiotrichales	Francisellaceae	Francisella	Francisella tularensis	1890909	2131
641522629	Francisella tularensis mediasiatica FSC147	Proteobacteria	Gammaproteobacteria	Thiotrichales	Francisellaceae	Francisella	Francisella tularensis	1893886	1454
646862328	Francisella tularensis tularensis NE061598	Proteobacteria	Gammaproteobacteria	Thiotrichales	Francisellaceae	Francisella	Francisella tularensis	1892681	1884
651053025	Francisella novicida 3523	Proteobacteria	Gammaproteobacteria	Thiotrichales	Francisellaceae	Francisella	Francisella tularensis	1945310	1902
651053026	Francisella cf. novicida Fx1	Proteobacteria	Gammaproteobacteria	Thiotrichales	Francisellaceae	Francisella	Francisella tularensis	1913619	1863
2504643031	Francisella tularensis holarctica FSC200	Proteobacteria	Gammaproteobacteria	Thiotrichales	Francisellaceae	Francisella	Francisella tularensis	1894157	2139
2512564026	Francisella tularensis TIGB03	Proteobacteria	Gammaproteobacteria	Thiotrichales	Francisellaceae	Francisella	Francisella tularensis	1968651	1850
2512564035	Francisella tularensis sv. Type A1 TI0902	Proteobacteria	Gammaproteobacteria	Thiotrichales	Francisellaceae	Francisella	Francisella tularensis	1892744	1764
2519899533	Francisella tularensis holarctica F92	Proteobacteria	Gammaproteobacteria	Thiotrichales	Francisellaceae	Francisella	Francisella tularensis	1886888	1890
2558860257	Francisella tularensis holarctica PHIT-FT049	Proteobacteria	Gammaproteobacteria	Thiotrichales	Francisellaceae	Francisella	Francisella tularensis	1881193	2002
2623620231	Francisella tularensis novicida U112 (re-annotation)	Proteobacteria	Gammaproteobacteria	Thiotrichales	Francisellaceae	Francisella	Francisella tularensis	1910031	1847
2623620698	Francisella tularensis tularensis FSC 198 (re-annotation)	Proteobacteria	Gammaproteobacteria	Thiotrichales	Francisellaceae	Francisella	Francisella tularensis	1892616	2081
2623620818	Francisella tularensis tularensis CHU S4 (re-annotation)	Proteobacteria	Gammaproteobacteria	Thiotrichales	Francisellaceae	Francisella	Francisella tularensis	1892775	2081
2623620860	Francisella tularensis holarctica LVS (re-annotation)	Proteobacteria	Gammaproteobacteria	Thiotrichales	Francisellaceae	Francisella	Francisella tularensis	1895994	2150
2623620922	Francisella tularensis holarctica OSU18 (re-annotation)	Proteobacteria	Gammaproteobacteria	Thiotrichales	Francisellaceae	Francisella	Francisella tularensis	1895727	2165
2519899538	Cycloclasticus sp. P1	Proteobacteria	Gammaproteobacteria	Thiotrichales	Piscirickettsiaceae	Cycloclasticus	Cycloclasticus sp. P1	2363215	2292
2561511209	Cycloclasticus zancles 7-ME	Proteobacteria	Gammaproteobacteria	Thiotrichales	Piscirickettsiaceae	Cycloclasticus	Cycloclasticus zancles	2655425	2623
2512564005	Methylophaga frappieri JAM7	Proteobacteria	Gammaproteobacteria	Thiotrichales	Piscirickettsiaceae	Methylophaga	Methylophaga frappieri	2745290	2748
2512564004	Methylophaga nitratireducenticrescens JAM1	Proteobacteria	Gammaproteobacteria	Thiotrichales	Piscirickettsiaceae	Methylophaga	Methylophaga nitratireducenticrescens	3137192	3096
2506783063	Thioalkalimicrobium aerophilum AL3	Proteobacteria	Gammaproteobacteria	Thiotrichales	Piscirickettsiaceae	Thioalkalimicrobium	Thioalkalimicrobium aerophilum	2158359	2111
2505679009	Thioalkalimicrobium cyclicum ALM1, DSM 14477	Proteobacteria	Gammaproteobacteria	Thiotrichales	Piscirickettsiaceae	Thioalkalimicrobium	Thioalkalimicrobium cyclicum	1932455	1734
637000325	Thiomicrospira crunogena XCL-2	Proteobacteria	Gammaproteobacteria	Thiotrichales	Piscirickettsiaceae	Thiomicrospira	Thiomicrospira crunogena	2427734	2263
2619619276	Thioploca ingrica	Proteobacteria	Gammaproteobacteria	Thiotrichales	Thiotrichaceae	Thioploca	Thioploca ingrica	4810005	3956

639633019	Candidatus Ruthia magnifica Cm	Proteobacteria	Gammaproteobacteria	unclassified	unclassified	Candidatus Ruthia	Candidatus Ruthia magnifica	1160782	1118
2519103097	Simiduia agarivorans SA1	Proteobacteria	Gammaproteobacteria	unclassified	unclassified	Simiduia	Simiduia agarivorans	4309711	3836
640427107	Candidatus Vesicomyosocius okutanii HA	Proteobacteria	Gammaproteobacteria	unclassified	unclassified	unclassified	Calyptogena okutanii thioautotrophic gill symbiont	1022154	981
2623620265	Candidatus Vesicomyosocius okutanii HA (re-annotation)	Proteobacteria	Gammaproteobacteria	unclassified	unclassified	unclassified	Calyptogena okutanii thioautotrophic gill symbiont	1022154	1014
637000029	Baumannia cicadellinicola Hc	Proteobacteria	Gammaproteobacteria	unclassified	unclassified	unclassified	Candidatus Baumannia cicadellinicola	686194	655
2623620902	Baumannia cicadellinicola Hc (re-annotation)	Proteobacteria	Gammaproteobacteria	unclassified	unclassified	unclassified	Candidatus Baumannia cicadellinicola	686194	658
2511231104	gamma proteobacterium sp. HdN1	Proteobacteria	Gammaproteobacteria	unclassified	unclassified	unclassified	gamma proteobacterium HdN1	4587455	3864
2609459601	Sedimenticola thiotaurini SIP-G1	Proteobacteria	Gammaproteobacteria	unclassified	unclassified	unclassified	unclassified	3962284	3708
637000334	Aliivibrio fischeri ES114	Proteobacteria	Gammaproteobacteria	Vibrionales	Vibrionaceae	Aliivibrio	Aliivibrio fischeri	4284050	3984
642555167	Vibrio fischeri MJ11	Proteobacteria	Gammaproteobacteria	Vibrionales	Vibrionaceae	Aliivibrio	Aliivibrio fischeri	4503336	4175
2623620826	Aliivibrio fischeri ES114 (re-annotation)	Proteobacteria	Gammaproteobacteria	Vibrionales	Vibrionaceae	Aliivibrio	Aliivibrio fischeri	4273718	3998
643348506	Aliivibrio salmonicida LFI1238	Proteobacteria	Gammaproteobacteria	Vibrionales	Vibrionaceae	Aliivibrio	Aliivibrio salmonicida	4655660	4075
637000206	Photobacterium profundum SS9	Proteobacteria	Gammaproteobacteria	Vibrionales	Vibrionaceae	Photobacterium	Photobacterium profundum	6403280	5746
2623620729	Photobacterium profundum SS9 (re-annotation)	Proteobacteria	Gammaproteobacteria	Vibrionales	Vibrionaceae	Photobacterium	Photobacterium profundum	6403280	6076
2563366623	Vibrio alginolyticus ATCC 17749	Proteobacteria	Gammaproteobacteria	Vibrionales	Vibrionaceae	Vibrio	Vibrio alginolyticus	5146637	4837
650716104	Vibrio anguillarum sv. O1 775	Proteobacteria	Gammaproteobacteria	Vibrionales	Vibrionaceae	Vibrio	Vibrio anguillarum	4052047	3836
2561511216	Listonella anguillarum M3	Proteobacteria	Gammaproteobacteria	Vibrionales	Vibrionaceae	Vibrio	Vibrio anguillarum	4117885	3882
640753058	Vibrio harveyi BB120, ATCC BAA-1116	Proteobacteria	Gammaproteobacteria	Vibrionales	Vibrionaceae	Vibrio	Vibrio campbellii	6058377	6252
2561511217	Vibrio campbellii ATCC BAA-1116	Proteobacteria	Gammaproteobacteria	Vibrionales	Vibrionaceae	Vibrio	Vibrio campbellii	6031829	5674
2623620248	Vibrio harveyi BB120, ATCC BAA-1116 (re-annotation)	Proteobacteria	Gammaproteobacteria	Vibrionales	Vibrionaceae	Vibrio	Vibrio campbellii	6058377	5928
637000333	Vibrio cholerae sv. O1 bv. El Tor N16961	Proteobacteria	Gammaproteobacteria	Vibrionales	Vibrionaceae	Vibrio	Vibrio cholerae	4033464	3998
640427151	Vibrio cholerae sv. O1 O395	Proteobacteria	Gammaproteobacteria	Vibrionales	Vibrionaceae	Vibrio	Vibrio cholerae	4132319	4031
643692053	Vibrio cholerae sv. O1 M66-2	Proteobacteria	Gammaproteobacteria	Vibrionales	Vibrionaceae	Vibrio	Vibrio cholerae	3938905	3812
643692054	Vibrio cholerae MJ-1236	Proteobacteria	Gammaproteobacteria	Vibrionales	Vibrionaceae	Vibrio	Vibrio cholerae	4236368	3894
646862349	Vibrio cholerae sv. O1 O395	Proteobacteria	Gammaproteobacteria	Vibrionales	Vibrionaceae	Vibrio	Vibrio cholerae	4135300	4055
651053078	Vibrio cholerae LMA3894-4	Proteobacteria	Gammaproteobacteria	Vibrionales	Vibrionaceae	Vibrio	Vibrio cholerae	3738715	3270
2511231085	Vibrio cholerae sv. O1 2010EL-1786	Proteobacteria	Gammaproteobacteria	Vibrionales	Vibrionaceae	Vibrio	Vibrio cholerae	4077740	3946
2512564028	Vibrio cholerae IEC224	Proteobacteria	Gammaproteobacteria	Vibrionales	Vibrionaceae	Vibrio	Vibrio cholerae	4079586	3787
2623620264	Vibrio cholerae sv. O1 O395 (re-annotation)	Proteobacteria	Gammaproteobacteria	Vibrionales	Vibrionaceae	Vibrio	Vibrio cholerae	4132319	3886
2623620745	Vibrio cholerae sv. O1 bv. El Tor N16961 (re-annotation)	Proteobacteria	Gammaproteobacteria	Vibrionales	Vibrionaceae	Vibrio	Vibrio cholerae	4033464	3744
650377984	Vibrio furnissii 2510/74, NCTC 11218	Proteobacteria	Gammaproteobacteria	Vibrionales	Vibrionaceae	Vibrio	Vibrio furnissii	4916408	4584
2563366596	Vibrio nigripulchritudo SnF1	Proteobacteria	Gammaproteobacteria	Vibrionales	Vibrionaceae	Vibrio	Vibrio nigripulchritudo	6322155	5807
637000335	Vibrio parahaemolyticus sv. O3:K6 RIMD 2210633	Proteobacteria	Gammaproteobacteria	Vibrionales	Vibrionaceae	Vibrio	Vibrio parahaemolyticus	5165770	5032
2558860260	Vibrio parahaemolyticus UCM-V493	Proteobacteria	Gammaproteobacteria	Vibrionales	Vibrionaceae	Vibrio	Vibrio parahaemolyticus	5232572	4978
2563366579	Vibrio parahaemolyticus BB22OP	Proteobacteria	Gammaproteobacteria	Vibrionales	Vibrionaceae	Vibrio	Vibrio parahaemolyticus	5103524	4689
2623620988	Vibrio parahaemolyticus sv. O3:K6 RIMD 2210633 (re-annotation)	Proteobacteria	Gammaproteobacteria	Vibrionales	Vibrionaceae	Vibrio	Vibrio parahaemolyticus	5165770	4768
2511231129	Vibrio sp. EJY3	Proteobacteria	Gammaproteobacteria	Vibrionales	Vibrionaceae	Vibrio	Vibrio sp. EJY3	5452646	4935
646311966	Vibrio sp. Ex25	Proteobacteria	Gammaproteobacteria	Vibrionales	Vibrionaceae	Vibrio	Vibrio sp. Ex25	5089025	4676
643348587	Vibrio splendidus LGP32	Proteobacteria	Gammaproteobacteria	Vibrionales	Vibrionaceae	Vibrio	Vibrio tasmaniensis	4974821	4572
637000336	Vibrio vulnificus CMCP6	Proteobacteria	Gammaproteobacteria	Vibrionales	Vibrionaceae	Vibrio	Vibrio vulnificus	5126798	4665
637000337	Vibrio vulnificus YJ016	Proteobacteria	Gammaproteobacteria	Vibrionales	Vibrionaceae	Vibrio	Vibrio vulnificus	5260086	5202
649633107	Vibrio vulnificus M06-24/O	Proteobacteria	Gammaproteobacteria	Vibrionales	Vibrionaceae	Vibrio	Vibrio vulnificus	5007768	4701
2597490180	Vibrio vulnificus 93U204	Proteobacteria	Gammaproteobacteria	Vibrionales	Vibrionaceae	Vibrio	Vibrio vulnificus	5127345	4683
2623620875	Vibrio vulnificus CMCP6 (re-annotation)	Proteobacteria	Gammaproteobacteria	Vibrionales	Vibrionaceae	Vibrio	Vibrio vulnificus	5126696	4693
2623620989	Vibrio vulnificus YJ016 (re-annotation)	Proteobacteria	Gammaproteobacteria	Vibrionales	Vibrionaceae	Vibrio	Vibrio vulnificus	5260086	4810
2588253754	Dyella japonica A8	Proteobacteria	Gammaproteobacteria	Xanthomonadales	Xanthomonadaceae	Dyella	Dyella japonica	4831185	3884
2576861470	Dyella jiangningensis SBZ 3-12	Proteobacteria	Gammaproteobacteria	Xanthomonadales	Xanthomonadaceae	Dyella	Dyella jiangningensis	5396991	4765
2512047037	Pseudoxanthomonas spadix BD-a59	Proteobacteria	Gammaproteobacteria	Xanthomonadales	Xanthomonadaceae	Pseudoxanthomonas	Pseudoxanthomonas spadix	3452554	3202
649633087	Pseudoxanthomonas suwonensis 11-1	Proteobacteria	Gammaproteobacteria	Xanthomonadales	Xanthomonadaceae	Pseudoxanthomonas	Pseudoxanthomonas suwonensis	3419049	3171
2506520041	Rhodanobacter sp. 2APBS1	Proteobacteria	Gammaproteobacteria	Xanthomonadales	Xanthomonadaceae	Rhodanobacter	Rhodanobacter denitrificans	4225490	3962
642555161	Stenotrophomonas maltophilia K279a	Proteobacteria	Gammaproteobacteria	Xanthomonadales	Xanthomonadaceae	Stenotrophomonas	Stenotrophomonas maltophilia	4851126	4472
642555162	Stenotrophomonas maltophilia R551-3	Proteobacteria	Gammaproteobacteria	Xanthomonadales	Xanthomonadaceae	Stenotrophomonas	Stenotrophomonas maltophilia	4573969	4129
2513237183	Stenotrophomonas maltophilia D457	Proteobacteria	Gammaproteobacteria	Xanthomonadales	Xanthomonadaceae	Stenotrophomonas	Stenotrophomonas maltophilia	4769156	4239

2619619094	Stenotrophomonas maltophilia JV3	Proteobacteria	Gammaproteobacteria	Xanthomonadales	Xanthomonadaceae	Stenotrophomonas	Stenotrophomonas maltophilia	4544477	4206
2576861471	Stenotrophomonas rhizophila DSM 14405	Proteobacteria	Gammaproteobacteria	Xanthomonadales	Xanthomonadaceae	Stenotrophomonas	Stenotrophomonas rhizophila	4648976	4047
2514752017	Xanthomonas albilineans GPE PC73R (XalbR)	Proteobacteria	Gammaproteobacteria	Xanthomonadales	Xanthomonadaceae	Xanthomonas	Xanthomonas albilineans	3768695	3177
2562617136	Xanthomonas albilineans GPE PC73	Proteobacteria	Gammaproteobacteria	Xanthomonadales	Xanthomonadaceae	Xanthomonas	Xanthomonas albilineans	3852299	3266
2511231060	Xanthomonas axonopodis pv. citrumelo F1	Proteobacteria	Gammaproteobacteria	Xanthomonadales	Xanthomonadaceae	Xanthomonas	Xanthomonas alfalfae	4967469	4241
2597489952	Xanthomonas alfalfae alfalfae CFBP 3836	Proteobacteria	Gammaproteobacteria	Xanthomonadales	Xanthomonadaceae	Xanthomonas	Xanthomonas alfalfae	5075866	4295
2554235395	Xanthomonas axonopodis Xac29-1	Proteobacteria	Gammaproteobacteria	Xanthomonadales	Xanthomonadaceae	Xanthomonas	Xanthomonas axonopodis	5296525	4466
2599185106	Xanthomonas axonopodis pv. glycines CFBP 2526	Proteobacteria	Gammaproteobacteria	Xanthomonadales	Xanthomonadaceae	Xanthomonas	Xanthomonas axonopodis	5251038	4534
2599185107	Xanthomonas axonopodis pv. glycines CFBP 7119	Proteobacteria	Gammaproteobacteria	Xanthomonadales	Xanthomonadaceae	Xanthomonas	Xanthomonas axonopodis	5518124	4785
637000343	Xanthomonas campestris pv. campestris 8004	Proteobacteria	Gammaproteobacteria	Xanthomonadales	Xanthomonadaceae	Xanthomonas	Xanthomonas campestris	5148708	4346
637000344	Xanthomonas campestris pv. Campestris	Proteobacteria	Gammaproteobacteria	Xanthomonadales	Xanthomonadaceae	Xanthomonas	Xanthomonas campestris	5076188	4254
642555169	Xanthomonas campestris pv. campestris B100	Proteobacteria	Gammaproteobacteria	Xanthomonadales	Xanthomonadaceae	Xanthomonas	Xanthomonas campestris	5079002	4529
2511231136	Xanthomonas campestris pv. raphani 756C	Proteobacteria	Gammaproteobacteria	Xanthomonadales	Xanthomonadaceae	Xanthomonas	Xanthomonas campestris	4941214	4597
2623620747	Xanthomonas campestris pv. campestris 8004 (re-annotation)	Proteobacteria	Gammaproteobacteria	Xanthomonadales	Xanthomonadaceae	Xanthomonas	Xanthomonas campestris	5148708	4418
2623620992	Xanthomonas campestris pv. Campestris (re-annotation)	Proteobacteria	Gammaproteobacteria	Xanthomonadales	Xanthomonadaceae	Xanthomonas	Xanthomonas campestris	5076187	4364
2551306373	Xanthomonas cassavae CFBP 4642	Proteobacteria	Gammaproteobacteria	Xanthomonadales	Xanthomonadaceae	Xanthomonas	Xanthomonas cassavae	5263056	4760
637000342	Xanthomonas axonopodis pv. citri 306	Proteobacteria	Gammaproteobacteria	Xanthomonadales	Xanthomonadaceae	Xanthomonas	Xanthomonas citri	5274174	4501
2554235008	Xanthomonas citri pv. malvacearum X20	Proteobacteria	Gammaproteobacteria	Xanthomonadales	Xanthomonadaceae	Xanthomonas	Xanthomonas citri	5216199	4524
2554235009	Xanthomonas citri pv. malvacearum X18	Proteobacteria	Gammaproteobacteria	Xanthomonadales	Xanthomonadaceae	Xanthomonas	Xanthomonas citri	4989917	4290
2561511166	Xanthomonas citri citri Aw	Proteobacteria	Gammaproteobacteria	Xanthomonadales	Xanthomonadaceae	Xanthomonas	Xanthomonas citri	5398685	4820
2623620794	Xanthomonas axonopodis pv. citri 306 (re-annotation)	Proteobacteria	Gammaproteobacteria	Xanthomonadales	Xanthomonadaceae	Xanthomonas	Xanthomonas citri	5274173	4550
637000345	Xanthomonas campestris pv. vesicatoria 85-10	Proteobacteria	Gammaproteobacteria	Xanthomonadales	Xanthomonadaceae	Xanthomonas	Xanthomonas euvesicatoria	5420152	4800
2623620993	Xanthomonas campestris pv. vesicatoria 85-10 (re-annotation)	Proteobacteria	Gammaproteobacteria	Xanthomonadales	Xanthomonadaceae	Xanthomonas	Xanthomonas euvesicatoria	5420152	4768
2554235375	Xanthomonas fuscans fuscans 4834-R	Proteobacteria	Gammaproteobacteria	Xanthomonadales	Xanthomonadaceae	Xanthomonas	Xanthomonas fuscans	5088683	4290
637000346	Xanthomonas oryzae pv. oryzae KACC10331	Proteobacteria	Gammaproteobacteria	Xanthomonadales	Xanthomonadaceae	Xanthomonas	Xanthomonas oryzae	4941439	4300
637000347	Xanthomonas oryzae pv. oryzae MAFF 311018	Proteobacteria	Gammaproteobacteria	Xanthomonadales	Xanthomonadaceae	Xanthomonas	Xanthomonas oryzae	4940217	4443
642555170	Xanthomonas oryzae pv. oryzae PXO99A	Proteobacteria	Gammaproteobacteria	Xanthomonadales	Xanthomonadaceae	Xanthomonas	Xanthomonas oryzae	5240075	5048
2513237179	Xanthomonas oryzae pv. oryzicola BLS256	Proteobacteria	Gammaproteobacteria	Xanthomonadales	Xanthomonadaceae	Xanthomonas	Xanthomonas oryzae	4831739	4561
2623620768	Xanthomonas oryzae pv. oryzae MAFF 311018 (re-annotation)	Proteobacteria	Gammaproteobacteria	Xanthomonadales	Xanthomonadaceae	Xanthomonas	Xanthomonas oryzae	4940217	4809
2623620795	Xanthomonas oryzae pv. oryzae KACC10331 (re-annotation)	Proteobacteria	Gammaproteobacteria	Xanthomonadales	Xanthomonadaceae	Xanthomonas	Xanthomonas oryzae	4941439	4901
637000348	Xylella fastidiosa 9a5c	Proteobacteria	Gammaproteobacteria	Xanthomonadales	Xanthomonadaceae	Xylella	Xylella fastidiosa	2731750	2895
637000349	Xylella fastidiosa Temecula1	Proteobacteria	Gammaproteobacteria	Xanthomonadales	Xanthomonadaceae	Xylella	Xylella fastidiosa	2521148	2102
641522659	Xylella fastidiosa M12	Proteobacteria	Gammaproteobacteria	Xanthomonadales	Xanthomonadaceae	Xylella	Xylella fastidiosa	2475130	2365
641522660	Xylella fastidiosa M23	Proteobacteria	Gammaproteobacteria	Xanthomonadales	Xanthomonadaceae	Xylella	Xylella fastidiosa	2573987	2295
648231719	Xylella fastidiosa fastidiosa GB514	Proteobacteria	Gammaproteobacteria	Xanthomonadales	Xanthomonadaceae	Xylella	Xylella fastidiosa	2517383	2271
2510461048	Xylella fastidiosa 3124 (V3.0)	Proteobacteria	Gammaproteobacteria	Xanthomonadales	Xanthomonadaceae	Xylella	Xylella fastidiosa	2748594	2729
2513237125	Xylella fastidiosa 3124 (Xf3124_v3.0)	Proteobacteria	Gammaproteobacteria	Xanthomonadales	Xanthomonadaceae	Xylella	Xylella fastidiosa	2748594	2729
2514752001	Xylella fastidiosa DixonR (Dixon_LNCC)	Proteobacteria	Gammaproteobacteria	Xanthomonadales	Xanthomonadaceae	Xylella	Xylella fastidiosa	2629797	2710
2514752003	Xylella fastidiosa Ann1R (Ann1_LNCC)	Proteobacteria	Gammaproteobacteria	Xanthomonadales	Xanthomonadaceae	Xylella	Xylella fastidiosa	2617604	2686
2514752005	Xylella fastidiosa DixonR (Dixon_IMGassembly)	Proteobacteria	Gammaproteobacteria	Xanthomonadales	Xanthomonadaceae	Xylella	Xylella fastidiosa	2548335	2540
2514752006	Xylella fastidiosa Ann1R (Ann_1_IMGassembly)	Proteobacteria	Gammaproteobacteria	Xanthomonadales	Xanthomonadaceae	Xylella	Xylella fastidiosa	2729755	2982
2514752007	Xylella fastidiosa 9a5cR (9a5cR)	Proteobacteria	Gammaproteobacteria	Xanthomonadales	Xanthomonadaceae	Xylella	Xylella fastidiosa	2679305	2639
2514752010	Xylella fastidiosa Temecula1R (Temecula1R)	Proteobacteria	Gammaproteobacteria	Xanthomonadales	Xanthomonadaceae	Xylella	Xylella fastidiosa	2519802	2476
2514752012	Xylella fastidiosa GB514R (GB415R)	Proteobacteria	Gammaproteobacteria	Xanthomonadales	Xanthomonadaceae	Xylella	Xylella fastidiosa	2491203	2470
2514752014	Xylella fastidiosa M23R (M23R)	Proteobacteria	Gammaproteobacteria	Xanthomonadales	Xanthomonadaceae	Xylella	Xylella fastidiosa	2535690	2498
2514752016	Xylella fastidiosa M12R (M12R)	Proteobacteria	Gammaproteobacteria	Xanthomonadales	Xanthomonadaceae	Xylella	Xylella fastidiosa	2475130	2419
2514752037	Xylella fastidiosa EB92_1R (EB92.1R)	Proteobacteria	Gammaproteobacteria	Xanthomonadales	Xanthomonadaceae	Xylella	Xylella fastidiosa	2475426	2514
2516493001	Xylella fastidiosa EB92_1R (Xylella fastidiosa EB92_1R2)	Proteobacteria	Gammaproteobacteria	Xanthomonadales	Xanthomonadaceae	Xylella	Xylella fastidiosa	2173663	2090
2585427629	Xylella fastidiosa MUL0034	Proteobacteria	Gammaproteobacteria	Xanthomonadales	Xanthomonadaceae	Xylella	Xylella fastidiosa	2666577	2561
2585427635	Xylella fastidiosa sandyi Ann-1	Proteobacteria	Gammaproteobacteria	Xanthomonadales	Xanthomonadaceae	Xylella	Xylella fastidiosa	2780908	2769
2623620748	Xylella fastidiosa 9a5c (re-annotation)	Proteobacteria	Gammaproteobacteria	Xanthomonadales	Xanthomonadaceae	Xylella	Xylella fastidiosa	2731748	2725
2623620796	Xylella fastidiosa Temecula1 (re-annotation)	Proteobacteria	Gammaproteobacteria	Xanthomonadales	Xanthomonadaceae	Xylella	Xylella fastidiosa	2521148	2478
2505119017	Paulinella chromatophora	unclassified	unclassified	Euglyphida	Paulinellidae	Paulinella	Paulinella chromatophora	1021616	923
**Inferred MSH synthesis: genomes encoding orthologs of Rv0486/mshA + Rv1170/mshB + Rv2130c/mshC + Rv0819/mshD**									

taxon_oid	Genome Name / Sample Name	Phylum	Class	Order	Family	Genus	Species	Genome Size	Gene Count
644736322	Acidimicrobium ferrooxidans ICP, DSM 10331	Actinobacteria	Actinobacteria	Acidimicrobiales	Acidimicrobiaceae	Acidimicrobium	Acidimicrobium ferrooxidans	2158157	2092
2545824624	Ilumatobacter coccineum YM16-304	Actinobacteria	Actinobacteria	Acidimicrobiales	Acidimicrobiaceae	Ilumatobacter	Ilumatobacter coccineus	4830181	4345
639633001	Acidothermus cellulolyticus 11B	Actinobacteria	Actinobacteria	Actinomycetales	Acidothermaceae	Acidothermus	Acidothermus cellulolyticus	2443540	2229
644736339	Catenulispora acidiphila ID139908, DSM 44928	Actinobacteria	Actinobacteria	Actinomycetales	Catenulisporaceae	Catenulispora	Catenulispora acidiphila	10467782	9125
2554235426	Corynebacterium argentoratense DSM 44202	Actinobacteria	Actinobacteria	Actinomycetales	Corynebacteriaceae	Corynebacterium	Corynebacterium argentoratense	2031902	1939
2585427661	Corynebacterium atypicum R2070	Actinobacteria	Actinobacteria	Actinomycetales	Corynebacteriaceae	Corynebacterium	Corynebacterium atypicum	2359448	1765
637000082	Corynebacterium diphtheriae bv. Gravis	Actinobacteria	Actinobacteria	Actinomycetales	Corynebacteriaceae	Corynebacterium	Corynebacterium diphtheriae	2488635	2395
2511231059	Corynebacterium diphtheriae HC03	Actinobacteria	Actinobacteria	Actinomycetales	Corynebacteriaceae	Corynebacterium	Corynebacterium diphtheriae	2478364	2330
2511231075	Corynebacterium diphtheriae 31A	Actinobacteria	Actinobacteria	Actinomycetales	Corynebacteriaceae	Corynebacterium	Corynebacterium diphtheriae	2535346	2458
2511231083	Corynebacterium diphtheriae HC01	Actinobacteria	Actinobacteria	Actinomycetales	Corynebacteriaceae	Corynebacterium	Corynebacterium diphtheriae	2427149	2321
2511231084	Corynebacterium diphtheriae HC04	Actinobacteria	Actinobacteria	Actinomycetales	Corynebacteriaceae	Corynebacterium	Corynebacterium diphtheriae	2484332	2342
2511231088	Corynebacterium diphtheriae INCA 402	Actinobacteria	Actinobacteria	Actinomycetales	Corynebacteriaceae	Corynebacterium	Corynebacterium diphtheriae	2449071	2292
2511231089	Corynebacterium diphtheriae C7 (beta)	Actinobacteria	Actinobacteria	Actinomycetales	Corynebacteriaceae	Corynebacterium	Corynebacterium diphtheriae	2499189	2414
2511231091	Corynebacterium diphtheriae HC02	Actinobacteria	Actinobacteria	Actinomycetales	Corynebacteriaceae	Corynebacterium	Corynebacterium diphtheriae	2468612	2306
2511231099	Corynebacterium diphtheriae BH8	Actinobacteria	Actinobacteria	Actinomycetales	Corynebacteriaceae	Corynebacterium	Corynebacterium diphtheriae	2485519	2436
2511231107	Corynebacterium diphtheriae VA01	Actinobacteria	Actinobacteria	Actinomycetales	Corynebacteriaceae	Corynebacterium	Corynebacterium diphtheriae	2395441	2258
2511231111	Corynebacterium diphtheriae PW8	Actinobacteria	Actinobacteria	Actinomycetales	Corynebacteriaceae	Corynebacterium	Corynebacterium diphtheriae	2530683	2412
2511231115	Corynebacterium diphtheriae CDCE 8392	Actinobacteria	Actinobacteria	Actinomycetales	Corynebacteriaceae	Corynebacterium	Corynebacterium diphtheriae	2433326	2330
2511231126	Corynebacterium diphtheriae 241	Actinobacteria	Actinobacteria	Actinomycetales	Corynebacteriaceae	Corynebacterium	Corynebacterium diphtheriae	2426551	2318
2571042744	Corynebacterium falsenii BL 8171, DSM 44353	Actinobacteria	Actinobacteria	Actinomycetales	Corynebacteriaceae	Corynebacterium	Corynebacterium falsenii	2719616	2401
2576861442	Corynebacterium glycinophilum AJ 3170	Actinobacteria	Actinobacteria	Actinomycetales	Corynebacteriaceae	Corynebacterium	Corynebacterium glycinophilum	3568218	3341
2597490159	Corynebacterium imitans DSM 44264	Actinobacteria	Actinobacteria	Actinomycetales	Corynebacteriaceae	Corynebacterium	Corynebacterium imitans	2565321	2413
637000085	Corynebacterium jeikeium K411	Actinobacteria	Actinobacteria	Actinomycetales	Corynebacteriaceae	Corynebacterium	Corynebacterium jeikeium	2476822	2186
643692019	Corynebacterium kroppenstedtii DSM 44385	Actinobacteria	Actinobacteria	Actinomycetales	Corynebacteriaceae	Corynebacterium	Corynebacterium kroppenstedtii	2446804	2073
2561511185	Corynebacterium maris DSM 45190 Genome sequencing	Actinobacteria	Actinobacteria	Actinomycetales	Corynebacteriaceae	Corynebacterium	Corynebacterium maris	2833547	2653
648028019	Corynebacterium pseudotuberculosis FRC41	Actinobacteria	Actinobacteria	Actinomycetales	Corynebacteriaceae	Corynebacterium	Corynebacterium pseudotuberculosis	2337913	2171
648231702	Corynebacterium pseudotuberculosis sv. Ovis 1002	Actinobacteria	Actinobacteria	Actinomycetales	Corynebacteriaceae	Corynebacterium	Corynebacterium pseudotuberculosis	2335112	2117
648231703	Corynebacterium pseudotuberculosis C231	Actinobacteria	Actinobacteria	Actinomycetales	Corynebacteriaceae	Corynebacterium	Corynebacterium pseudotuberculosis	2328208	2113
650377927	Corynebacterium pseudotuberculosis I19	Actinobacteria	Actinobacteria	Actinomycetales	Corynebacteriaceae	Corynebacterium	Corynebacterium pseudotuberculosis	2337730	2127
2511231067	Corynebacterium pseudotuberculosis sv. Ovis 3/99-5	Actinobacteria	Actinobacteria	Actinomycetales	Corynebacteriaceae	Corynebacterium	Corynebacterium pseudotuberculosis	2337938	2239
2511231142	Corynebacterium pseudotuberculosis 1/06-A	Actinobacteria	Actinobacteria	Actinomycetales	Corynebacteriaceae	Corynebacterium	Corynebacterium pseudotuberculosis	2279118	2127
2511231153	Corynebacterium pseudotuberculosis sv. II CIP52.97	Actinobacteria	Actinobacteria	Actinomycetales	Corynebacteriaceae	Corynebacterium	Corynebacterium pseudotuberculosis	2320595	2194
2511231154	Corynebacterium pseudotuberculosis PAT10	Actinobacteria	Actinobacteria	Actinomycetales	Corynebacteriaceae	Corynebacterium	Corynebacterium pseudotuberculosis	2335323	2200
2511231212	Corynebacterium pseudotuberculosis 42/02-A	Actinobacteria	Actinobacteria	Actinomycetales	Corynebacteriaceae	Corynebacterium	Corynebacterium pseudotuberculosis	2337606	2164
2512564036	Corynebacterium pseudotuberculosis 316	Actinobacteria	Actinobacteria	Actinomycetales	Corynebacteriaceae	Corynebacterium	Corynebacterium pseudotuberculosis	2310415	2234
2512564058	Corynebacterium pseudotuberculosis sv. Ovis P54B96	Actinobacteria	Actinobacteria	Actinomycetales	Corynebacteriaceae	Corynebacterium	Corynebacterium pseudotuberculosis	2337657	2207
2513237214	Corynebacterium pseudotuberculosis 267	Actinobacteria	Actinobacteria	Actinomycetales	Corynebacteriaceae	Corynebacterium	Corynebacterium pseudotuberculosis	2337628	2249
2518645565	Corynebacterium pseudotuberculosis Cp162	Actinobacteria	Actinobacteria	Actinomycetales	Corynebacteriaceae	Corynebacterium	Corynebacterium pseudotuberculosis	2293464	2150
2540341150	Corynebacterium pseudotuberculosis 31	Actinobacteria	Actinobacteria	Actinomycetales	Corynebacteriaceae	Corynebacterium	Corynebacterium pseudotuberculosis	2297010	2170
2588253761	Corynebacterium pseudotuberculosis Ft_2193/67	Actinobacteria	Actinobacteria	Actinomycetales	Corynebacteriaceae	Corynebacterium	Corynebacterium pseudotuberculosis	2338300	2305
2588253762	Corynebacterium pseudotuberculosis CS_10	Actinobacteria	Actinobacteria	Actinomycetales	Corynebacteriaceae	Corynebacterium	Corynebacterium pseudotuberculosis	2338144	2295
2588253763	Corynebacterium pseudotuberculosis 48252	Actinobacteria	Actinobacteria	Actinomycetales	Corynebacteriaceae	Corynebacterium	Corynebacterium pseudotuberculosis	2338139	2290
650716029	Corynebacterium resistens DSM 45100	Actinobacteria	Actinobacteria	Actinomycetales	Corynebacteriaceae	Corynebacterium	Corynebacterium resistens	2601311	2230
2597490150	Corynebacterium sp. 6931	Actinobacteria	Actinobacteria	Actinomycetales	Corynebacteriaceae	Corynebacterium	Corynebacterium sp. ATCC 6931	2471920	2186
2554235357	Corynebacterium terpenotabidum Y-11 Genome sequencing	Actinobacteria	Actinobacteria	Actinomycetales	Corynebacteriaceae	Corynebacterium	Corynebacterium terpenotabidum	2751233	2441
650716030	Corynebacterium ulcerans BR-AD22	Actinobacteria	Actinobacteria	Actinomycetales	Corynebacteriaceae	Corynebacterium	Corynebacterium ulcerans	2606374	2398
651053019	Corynebacterium ulcerans 809	Actinobacteria	Actinobacteria	Actinomycetales	Corynebacteriaceae	Corynebacterium	Corynebacterium ulcerans	2502095	2246
2517093035	Corynebacterium ulcerans 0102	Actinobacteria	Actinobacteria	Actinomycetales	Corynebacteriaceae	Corynebacterium	Corynebacterium ulcerans	2579188	2417
641522620	Corynebacterium urealyticum DSM 7109	Actinobacteria	Actinobacteria	Actinomycetales	Corynebacteriaceae	Corynebacterium	Corynebacterium urealyticum	2369219	2084
2529292687	Corynebacterium urealyticum DSM 7111	Actinobacteria	Actinobacteria	Actinomycetales	Corynebacteriaceae	Corynebacterium	Corynebacterium urealyticum	2316065	2007
2597490209	Corynebacterium ureicelerivorans IMMIB RIV-2301	Actinobacteria	Actinobacteria	Actinomycetales	Corynebacteriaceae	Corynebacterium	Corynebacterium ureicelerivorans	2328278	2357
2511231114	Corynebacterium variabile DSM 44702	Actinobacteria	Actinobacteria	Actinomycetales	Corynebacteriaceae	Corynebacterium	Corynebacterium variabile	3433007	3131
637000115	Frankia alni ACN14a	Actinobacteria	Actinobacteria	Actinomycetales	Frankiaceae	Frankia	Frankia alni	7497934	6795

637000116	Frankia sp. CcI3	Actinobacteria	Actinobacteria	Actinomycetales	Frankiaceae	Frankia	Frankia sp. CcI3	5433628	4621
641228492	Frankia sp. EAN1pec	Actinobacteria	Actinobacteria	Actinomycetales	Frankiaceae	Frankia	Frankia sp. EAN1pec	8982042	7250
649633045	Frankia sp. EuI1c	Actinobacteria	Actinobacteria	Actinomycetales	Frankiaceae	Frankia	Frankia sp. EuI1c	8815781	7262
2506783011	Frankia symbiont of Datisca glomerata Dg1	Actinobacteria	Actinobacteria	Actinomycetales	Frankiaceae	Frankia	Frankia symbiont of Datisca glomerata	5323186	4579
2512564033	Blastococcus saxobsidens DD2	Actinobacteria	Actinobacteria	Actinomycetales	Geodermatophilaceae	Blastococcus	Blastococcus saxobsidens	4875340	4910
646311931	Geodermatophilus obscurus G-20, DSM 43160	Actinobacteria	Actinobacteria	Actinomycetales	Geodermatophilaceae	Geodermatophilus	Geodermatophilus obscurus	5322497	5155
2540341106	Modestobacter multiseptatus BC501	Actinobacteria	Actinobacteria	Actinomycetales	Geodermatophilaceae	Modestobacter	Modestobacter multiseptatus	5575517	5562
646564571	Stackebrandtia nassauensis LLR-40K-21, DSM 44728	Actinobacteria	Actinobacteria	Actinomycetales	Glycomycetaceae	Stackebrandtia	Stackebrandtia nassauensis	6841557	6541
2512564042	Gordonia polyisoprenivorans VH2, DSM 44266	Actinobacteria	Actinobacteria	Actinomycetales	Gordoniaceae	Gordonia	Gordonia polyisoprenivorans	5844299	5188
2523231035	Gordonia sp. KTR9	Actinobacteria	Actinobacteria	Actinomycetales	Gordoniaceae	Gordonia	Gordonia sp. KTR9	5885710	5217
649633057	Intrasporangium calvum 7KIP, DSM 43043	Actinobacteria	Actinobacteria	Actinomycetales	Intrasporangiaceae	Intrasporangium	Intrasporangium calvum	4024382	3710
640753031	Kineococcus radiotolerans SRS30216	Actinobacteria	Actinobacteria	Actinomycetales	Kineosporiaceae	Kineococcus	Kineococcus radiotolerans	4956672	4785
639633005	Arthrobacter aurescens TC1	Actinobacteria	Actinobacteria	Actinomycetales	Micrococcaceae	Arthrobacter	Arthrobacter aurescens	5226648	4793
2623620277	Arthrobacter aurescens TC1 (re-annotation)	Actinobacteria	Actinobacteria	Actinomycetales	Micrococcaceae	Arthrobacter	Arthrobacter aurescens	5226648	4888
643348509	Arthrobacter chlorophenolicus A6	Actinobacteria	Actinobacteria	Actinomycetales	Micrococcaceae	Arthrobacter	Arthrobacter chlorophenolicus	4980870	4744
639633006	Arthrobacter sp. FB24	Actinobacteria	Actinobacteria	Actinomycetales	Micrococcaceae	Arthrobacter	Arthrobacter sp. FB24	5070478	4622
642555133	Kocuria rhizophila DC2201	Actinobacteria	Actinobacteria	Actinomycetales	Micrococcaceae	Kocuria	Kocuria rhizophila	2697540	2413
644736390	Micrococcus luteus NCTC 2665	Actinobacteria	Actinobacteria	Actinomycetales	Micrococcaceae	Micrococcus	Micrococcus luteus	2501097	2342
2554235379	Actinoplanes friuliensis DSM 7358	Actinobacteria	Actinobacteria	Actinomycetales	Micromonosporaceae	Actinoplanes	Actinoplanes friuliensis	9376071	8648
2513237176	Actinoplanes missouriensis NBRC 102363	Actinobacteria	Actinobacteria	Actinomycetales	Micromonosporaceae	Actinoplanes	Actinoplanes missouriensis	8773466	8202
2554235409	Actinoplanes sp. N902-109	Actinobacteria	Actinobacteria	Actinomycetales	Micromonosporaceae	Actinoplanes	Actinoplanes sp. N902-109	9228054	8283
2511231151	Actinoplanes sp. SE50/110	Actinobacteria	Actinobacteria	Actinomycetales	Micromonosporaceae	Actinoplanes	Actinoplanes sp. SE50/110	9239851	8385
648028042	Micromonospora aurantiaca ATCC 27029	Actinobacteria	Actinobacteria	Actinomycetales	Micromonosporaceae	Micromonospora	Micromonospora aurantiaca	7025559	6360
649633069	Micromonospora sp. L5	Actinobacteria	Actinobacteria	Actinomycetales	Micromonosporaceae	Micromonospora	Micromonospora sp. L5	6962533	6326
641228504	Salinispora arenicola CNS-205	Actinobacteria	Actinobacteria	Actinomycetales	Micromonosporaceae	Salinispora	Salinispora arenicola	5786361	5169
640427140	Salinispora tropica CNB-440	Actinobacteria	Actinobacteria	Actinomycetales	Micromonosporaceae	Salinispora	Salinispora tropica	5183331	4664
650716103	Verrucosispora maris AB-18-032	Actinobacteria	Actinobacteria	Actinomycetales	Micromonosporaceae	Verrucosispora	Verrucosispora maris	6732271	6069
650716009	Amycolicicoccus subflavus DQS3-9A1	Actinobacteria	Actinobacteria	Actinomycetales	Mycobacteriaceae	Amycolicicoccus	Amycolicicoccus subflavus	4863490	4759
641522641	Mycobacterium abscessus CIP 104536	Actinobacteria	Actinobacteria	Actinomycetales	Mycobacteriaceae	Mycobacterium	Mycobacterium abscessus	5090491	4991
2517093032	Mycobacterium massiliense GO 06	Actinobacteria	Actinobacteria	Actinomycetales	Mycobacteriaceae	Mycobacterium	Mycobacterium abscessus	5068807	2677
2561511210	Mycobacterium abscessus bolletii 50594	Actinobacteria	Actinobacteria	Actinomycetales	Mycobacteriaceae	Mycobacterium	Mycobacterium abscessus	5270527	5235
2597489897	Mycobacterium abscessus bolletii INCQS 00594	Actinobacteria	Actinobacteria	Actinomycetales	Mycobacteriaceae	Mycobacterium	Mycobacterium abscessus	4941231	4976
650716059	Mycobacterium africanum GM041182	Actinobacteria	Actinobacteria	Actinomycetales	Mycobacteriaceae	Mycobacterium	Mycobacterium africanum	4389314	3880
637000168	Mycobacterium avium paratuberculosis K-10	Actinobacteria	Actinobacteria	Actinomycetales	Mycobacteriaceae	Mycobacterium	Mycobacterium avium	4829781	4415
639633039	Mycobacterium avium 104	Actinobacteria	Actinobacteria	Actinomycetales	Mycobacteriaceae	Mycobacterium	Mycobacterium avium	5475491	5305
2554235361	Mycobacterium avium paratuberculosis MAP4	Actinobacteria	Actinobacteria	Actinomycetales	Mycobacteriaceae	Mycobacterium	Mycobacterium avium	4829424	4376
2623620229	Mycobacterium avium 104 (re-annotation)	Actinobacteria	Actinobacteria	Actinomycetales	Mycobacteriaceae	Mycobacterium	Mycobacterium avium	5475491	5276
637000169	Mycobacterium bovis AF 2122/97	Actinobacteria	Actinobacteria	Actinomycetales	Mycobacteriaceae	Mycobacterium	Mycobacterium bovis	4345492	4014
639633040	Mycobacterium bovis BCG Pasteur 1173P2	Actinobacteria	Actinobacteria	Actinomycetales	Mycobacteriaceae	Mycobacterium	Mycobacterium bovis	4374522	4048
643692028	Mycobacterium bovis BCG Tokyo 172	Actinobacteria	Actinobacteria	Actinomycetales	Mycobacteriaceae	Mycobacterium	Mycobacterium bovis	4371711	3996
2511231152	Mycobacterium bovis BCG Mexico	Actinobacteria	Actinobacteria	Actinomycetales	Mycobacteriaceae	Mycobacterium	Mycobacterium bovis	4350386	4030
2540341164	Mycobacterium bovis BCG Korea 1168P	Actinobacteria	Actinobacteria	Actinomycetales	Mycobacteriaceae	Mycobacterium	Mycobacterium bovis	4304386	4120
2619619103	Mycobacterium bovis BCG Moreau RDJ	Actinobacteria	Actinobacteria	Actinomycetales	Mycobacteriaceae	Mycobacterium	Mycobacterium bovis	4340116	4037
2623620293	Mycobacterium bovis BCG Pasteur 1173P2 (re-annotation)	Actinobacteria	Actinobacteria	Actinomycetales	Mycobacteriaceae	Mycobacterium	Mycobacterium bovis	4374522	4090
650716060	Mycobacterium canettii CIPT 140010059	Actinobacteria	Actinobacteria	Actinomycetales	Mycobacteriaceae	Mycobacterium	Mycobacterium canettii	4482059	3909
2541047045	Mycobacterium canettii CIPT 140070008	Actinobacteria	Actinobacteria	Actinomycetales	Mycobacteriaceae	Mycobacterium	Mycobacterium canettii	4420197	4110
2506783014	Mycobacterium chubuense NBB4	Actinobacteria	Actinobacteria	Actinomycetales	Mycobacteriaceae	Mycobacterium	Mycobacterium chubuense	6342624	6069
640427122	Mycobacterium gilvum PYR-GCK	Actinobacteria	Actinobacteria	Actinomycetales	Mycobacteriaceae	Mycobacterium	Mycobacterium gilvum	5982829	5683
649633070	Mycobacterium gilvum Spyr1	Actinobacteria	Actinobacteria	Actinomycetales	Mycobacteriaceae	Mycobacterium	Mycobacterium gilvum	5783292	5434
2521172703	Mycobacterium indicus pranii MTCC 9506	Actinobacteria	Actinobacteria	Actinomycetales	Mycobacteriaceae	Mycobacterium	Mycobacterium indicus pranii	5589007	5318
2512564041	Mycobacterium intracellulare MOTT-64	Actinobacteria	Actinobacteria	Actinomycetales	Mycobacteriaceae	Mycobacterium	Mycobacterium intracellulare	5501090	5297
2512564050	Mycobacterium intracellulare MOTT-02	Actinobacteria	Actinobacteria	Actinomycetales	Mycobacteriaceae	Mycobacterium	Mycobacterium intracellulare	5409696	5198
2519103109	Mycobacterium intracellulare ATCC 13950	Actinobacteria	Actinobacteria	Actinomycetales	Mycobacteriaceae	Mycobacterium	Mycobacterium intracellulare	5402402	5193
2563366550	Mycobacterium kansasii ATCC 12478	Actinobacteria	Actinobacteria	Actinomycetales	Mycobacteriaceae	Mycobacterium	Mycobacterium kansasii	6577228	5928
637000170	Mycobacterium leprae TN	Actinobacteria	Actinobacteria	Actinomycetales	Mycobacteriaceae	Mycobacterium	Mycobacterium leprae	3268203	2750

643348566	Mycobacterium leprae Br4923	Actinobacteria	Actinobacteria	Actinomycetales	Mycobacteriaceae	Mycobacterium	Mycobacterium leprae	3268071	1654
2563366549	Mycobacterium liflandii 128FXT	Actinobacteria	Actinobacteria	Actinomycetales	Mycobacteriaceae	Mycobacterium	Mycobacterium liflandii	6399543	5570
641522642	Mycobacterium marinum M, ATCC BAA-535	Actinobacteria	Actinobacteria	Actinomycetales	Mycobacteriaceae	Mycobacterium	Mycobacterium marinum	6660144	5501
2551306466	Mycobacterium neoaurum VKM Ac-1815D	Actinobacteria	Actinobacteria	Actinomycetales	Mycobacteriaceae	Mycobacterium	Mycobacterium neoaurum	5400604	5212
2508501106	Mycobacterium rhodesiae NBB3	Actinobacteria	Actinobacteria	Actinomycetales	Mycobacteriaceae	Mycobacterium	Mycobacterium rhodesiae	6415739	6342
639633041	Mycobacterium smegmatis MC2 155	Actinobacteria	Actinobacteria	Actinomycetales	Mycobacteriaceae	Mycobacterium	Mycobacterium smegmatis	6988209	6941
2506783060	Mycobacterium smegmatis JS623	Actinobacteria	Actinobacteria	Actinomycetales	Mycobacteriaceae	Mycobacterium	Mycobacterium smegmatis	7221766	7311
2518645537	Mycobacterium smegmatis MC2 155	Actinobacteria	Actinobacteria	Actinomycetales	Mycobacteriaceae	Mycobacterium	Mycobacterium smegmatis	6988208	6745
2623620257	Mycobacterium smegmatis MC2 155 (re-annotation)	Actinobacteria	Actinobacteria	Actinomycetales	Mycobacteriaceae	Mycobacterium	Mycobacterium smegmatis	6988209	6755
650716061	Mycobacterium sp. JDM601	Actinobacteria	Actinobacteria	Actinomycetales	Mycobacteriaceae	Mycobacterium	Mycobacterium sp. JDM601	4643668	4398
640069320	Mycobacterium sp. JLS	Actinobacteria	Actinobacteria	Actinomycetales	Mycobacteriaceae	Mycobacterium	Mycobacterium sp. JLS	6048425	5855
639633042	Mycobacterium sp. KMS	Actinobacteria	Actinobacteria	Actinomycetales	Mycobacteriaceae	Mycobacterium	Mycobacterium sp. KMS	6256079	6089
637000171	Mycobacterium sp. MCS	Actinobacteria	Actinobacteria	Actinomycetales	Mycobacteriaceae	Mycobacterium	Mycobacterium sp. MCS	5920523	5704
2518645550	Mycobacterium sp. MOTT36Y	Actinobacteria	Actinobacteria	Actinomycetales	Mycobacteriaceae	Mycobacterium	Mycobacterium sp. MOTT36Y	5613626	5177
637000172	Mycobacterium tuberculosis CDC1551	Actinobacteria	Actinobacteria	Actinomycetales	Mycobacteriaceae	Mycobacterium	Mycobacterium tuberculosis	4403837	4300
637000173	Mycobacterium tuberculosis H37Rv	Actinobacteria	Actinobacteria	Actinomycetales	Mycobacteriaceae	Mycobacterium	Mycobacterium tuberculosis	4411532	4062
640427123	Mycobacterium tuberculosis F11 (ExPEC)	Actinobacteria	Actinobacteria	Actinomycetales	Mycobacteriaceae	Mycobacterium	Mycobacterium tuberculosis	4424435	4019
640427124	Mycobacterium tuberculosis H37Ra	Actinobacteria	Actinobacteria	Actinomycetales	Mycobacteriaceae	Mycobacterium	Mycobacterium tuberculosis	4419977	4099
641736194	Mycobacterium tuberculosis Haarlem	Actinobacteria	Actinobacteria	Actinomycetales	Mycobacteriaceae	Mycobacterium	Mycobacterium tuberculosis	4347292	4376
644736391	Mycobacterium tuberculosis KZN 1435 (MDR)	Actinobacteria	Actinobacteria	Actinomycetales	Mycobacteriaceae	Mycobacterium	Mycobacterium tuberculosis	4398250	4107
651053043	Mycobacterium tuberculosis CCDC5079	Actinobacteria	Actinobacteria	Actinomycetales	Mycobacteriaceae	Mycobacterium	Mycobacterium tuberculosis	4398812	3695
651053044	Mycobacterium tuberculosis CCDC5180	Actinobacteria	Actinobacteria	Actinomycetales	Mycobacteriaceae	Mycobacterium	Mycobacterium tuberculosis	4405981	3639
2511231070	Mycobacterium tuberculosis KZN 4207 (DS)	Actinobacteria	Actinobacteria	Actinomycetales	Mycobacteriaceae	Mycobacterium	Mycobacterium tuberculosis	4394985	4043
2511231130	Mycobacterium tuberculosis CTRI-2	Actinobacteria	Actinobacteria	Actinomycetales	Mycobacteriaceae	Mycobacterium	Mycobacterium tuberculosis	4398525	3996
2512564056	Mycobacterium tuberculosis UT205	Actinobacteria	Actinobacteria	Actinomycetales	Mycobacteriaceae	Mycobacterium	Mycobacterium tuberculosis	4418088	3852
2512564073	Mycobacterium tuberculosis RGTB327	Actinobacteria	Actinobacteria	Actinomycetales	Mycobacteriaceae	Mycobacterium	Mycobacterium tuberculosis	4380119	3739
2513237185	Mycobacterium tuberculosis RGTB423	Actinobacteria	Actinobacteria	Actinomycetales	Mycobacteriaceae	Mycobacterium	Mycobacterium tuberculosis	4406587	3670
2526164708	Mycobacterium tuberculosis H37Rv	Actinobacteria	Actinobacteria	Actinomycetales	Mycobacteriaceae	Mycobacterium	Mycobacterium tuberculosis	4411708	4170
2540341098	Mycobacterium tuberculosis Erdman	Actinobacteria	Actinobacteria	Actinomycetales	Mycobacteriaceae	Mycobacterium	Mycobacterium tuberculosis	4392353	4301
2540341146	Mycobacterium tuberculosis	Actinobacteria	Actinobacteria	Actinomycetales	Mycobacteriaceae	Mycobacterium	Mycobacterium tuberculosis	4421197	4042
2545824625	Mycobacterium tuberculosis Beijing/NITR203	Actinobacteria	Actinobacteria	Actinomycetales	Mycobacteriaceae	Mycobacterium	Mycobacterium tuberculosis	4411128	4158
2545824629	Mycobacterium tuberculosis CAS/NITR204	Actinobacteria	Actinobacteria	Actinomycetales	Mycobacteriaceae	Mycobacterium	Mycobacterium tuberculosis	4392876	4008
2545824630	Mycobacterium tuberculosis EAI5/NITR206	Actinobacteria	Actinobacteria	Actinomycetales	Mycobacteriaceae	Mycobacterium	Mycobacterium tuberculosis	4390306	4067
2554235430	Mycobacterium tuberculosis EAI5	Actinobacteria	Actinobacteria	Actinomycetales	Mycobacteriaceae	Mycobacterium	Mycobacterium tuberculosis	4391174	4026
2558860211	Mycobacterium tuberculosis BS1	Actinobacteria	Actinobacteria	Actinomycetales	Mycobacteriaceae	Mycobacterium	Mycobacterium tuberculosis	4407929	4199
2565956582	Mycobacterium tuberculosis BT2	Actinobacteria	Actinobacteria	Actinomycetales	Mycobacteriaceae	Mycobacterium	Mycobacterium tuberculosis	4401899	4197
2588253735	Mycobacterium tuberculosis K	Actinobacteria	Actinobacteria	Actinomycetales	Mycobacteriaceae	Mycobacterium	Mycobacterium tuberculosis	4385518	4194
2588253748	Mycobacterium tuberculosis Korean KIT87190	Actinobacteria	Actinobacteria	Actinomycetales	Mycobacteriaceae	Mycobacterium	Mycobacterium tuberculosis	4410788	4027
2623620310	Mycobacterium tuberculosis F11 (ExPEC) (re-annotation)	Actinobacteria	Actinobacteria	Actinomycetales	Mycobacteriaceae	Mycobacterium	Mycobacterium tuberculosis	4424435	4149
2623620311	Mycobacterium tuberculosis H37Ra (re-annotation)	Actinobacteria	Actinobacteria	Actinomycetales	Mycobacteriaceae	Mycobacterium	Mycobacterium tuberculosis	4419977	4159
642555140	Mycobacterium ulcerans Agy99	Actinobacteria	Actinobacteria	Actinomycetales	Mycobacteriaceae	Mycobacterium	Mycobacterium ulcerans	5805761	4306
639633044	Mycobacterium vanbaalenii PYR-1	Actinobacteria	Actinobacteria	Actinomycetales	Mycobacteriaceae	Mycobacterium	Mycobacterium vanbaalenii	6491865	6047
2541047007	Mycobacterium sp. 05-1390	Actinobacteria	Actinobacteria	Actinomycetales	Mycobacteriaceae	Mycobacterium	Mycobacterium yongonense	5521023	5270
644736393	Nakamurella multipartita Y-104, DSM 44233	Actinobacteria	Actinobacteria	Actinomycetales	Nakamurellaceae	Nakamurella	Nakamurella multipartita	6060298	5471
646311932	Gordonia bronchialis 3410, DSM 43247	Actinobacteria	Actinobacteria	Actinomycetales	Nocardiaceae	Gordonia	Gordonia bronchialis	5290012	5002
2513237372	Nocardia brasiliensis ATCC 700358	Actinobacteria	Actinobacteria	Actinomycetales	Nocardiaceae	Nocardia	Nocardia brasiliensis	9489024	8548
2511231172	Nocardia cyriacigeorgica GUH-2	Actinobacteria	Actinobacteria	Actinomycetales	Nocardiaceae	Nocardia	Nocardia cyriacigeorgica	6194645	5560
637000198	Nocardia farcinica IFM 10152	Actinobacteria	Actinobacteria	Actinomycetales	Nocardiaceae	Nocardia	Nocardia farcinica	6292344	6011
2547132424	Nocardia nova SH22a (NONO)	Actinobacteria	Actinobacteria	Actinomycetales	Nocardiaceae	Nocardia	Nocardia nova	8348532	7641
643692033	Rhodococcus erythropolis PR4	Actinobacteria	Actinobacteria	Actinomycetales	Nocardiaceae	Rhodococcus	Rhodococcus erythropolis	6895538	6505
2561511168	Rhodococcus erythropolis CCM2595	Actinobacteria	Actinobacteria	Actinomycetales	Nocardiaceae	Rhodococcus	Rhodococcus erythropolis	6371421	5899
2597490086	Rhodococcus erythropolis R138	Actinobacteria	Actinobacteria	Actinomycetales	Nocardiaceae	Rhodococcus	Rhodococcus erythropolis	6776569	6361
649633089	Rhodococcus equi 103S	Actinobacteria	Actinobacteria	Actinomycetales	Nocardiaceae	Rhodococcus	Rhodococcus hoagii	5043170	4570
637000234	Rhodococcus jostii RHA1	Actinobacteria	Actinobacteria	Actinomycetales	Nocardiaceae	Rhodococcus	Rhodococcus jostii	9702737	9242
646564564	Rhodococcus opacus B4	Actinobacteria	Actinobacteria	Actinomycetales	Nocardiaceae	Rhodococcus	Rhodococcus opacus	8834939	8259

2576861435	Rhodococcus opacus PD630	Actinobacteria	Actinobacteria	Actinomycetales	Nocardiaceae	Rhodococcus	Rhodococcus opacus	9169032	9005
2516653027	Rhodococcus pyridinivorans SB3094 (v1.0)	Actinobacteria	Actinobacteria	Actinomycetales	Nocardiaceae	Rhodococcus	Rhodococcus pyridinivorans	5164718	4976
2597489953	Rhodococcus sp. BCP1	Actinobacteria	Actinobacteria	Actinomycetales	Nocardiaceae	Rhodococcus	Rhodococcus sp. BCP1	6231823	5789
646311938	Kribbella flavida IFO 14399, DSM 17836	Actinobacteria	Actinobacteria	Actinomycetales	Nocardioidaceae	Kribbella	Kribbella flavida	7579488	7149
639633046	Nocardioides sp. JS614	Actinobacteria	Actinobacteria	Actinomycetales	Nocardioidaceae	Nocardioides	Nocardioides sp. JS614	5293685	4975
2518645556	Nocardiopsis alba ATCC BAA-2165	Actinobacteria	Actinobacteria	Actinomycetales	Nocardiopsaceae	Nocardiopsis	Nocardiopsis alba	5848211	5609
646564557	Nocardiopsis dassonvillei dassonvillei DSM 43111	Actinobacteria	Actinobacteria	Actinomycetales	Nocardiopsaceae	Nocardiopsis	Nocardiopsis dassonvillei	6543312	5647
637000319	Thermobifida fusca YX	Actinobacteria	Actinobacteria	Actinomycetales	Nocardiopsaceae	Thermobifida	Thermobifida fusca	3642249	3195
646311968	Xylanimonas cellulosilytica XIL07, DSM 15894	Actinobacteria	Actinobacteria	Actinomycetales	Promicromonosporaceae	Xylanimonas	Xylanimonas cellulosilytica	3831380	3549
644736323	Actinosynnema mirum 101, DSM 43827	Actinobacteria	Actinobacteria	Actinomycetales	Pseudonocardiaceae	Actinosynnema	Actinosynnema mirum	8248144	7176
2585427649	Amycolatopsis japonica MG417-CF17, DSM 44213	Actinobacteria	Actinobacteria	Actinomycetales	Pseudonocardiaceae	Amycolatopsis	Amycolatopsis japonica	9053857	8500
2597490168	Amycolatopsis methanolica 239	Actinobacteria	Actinobacteria	Actinomycetales	Pseudonocardiaceae	Amycolatopsis	Amycolatopsis methanolica	7237391	7353
2561511175	Amycolatopsis orientalis HCCB10007	Actinobacteria	Actinobacteria	Actinomycetales	Pseudonocardiaceae	Amycolatopsis	Amycolatopsis orientalis	8982090	8232
2565956555	Kutzneria albida DSM 43870	Actinobacteria	Actinobacteria	Actinomycetales	Pseudonocardiaceae	Kutzneria	Kutzneria albida	9874926	8876
651053061	Pseudonocardia dioxanivorans CB1190	Actinobacteria	Actinobacteria	Actinomycetales	Pseudonocardiaceae	Pseudonocardia	Pseudonocardia dioxanivorans	7440794	7071
644736404	Saccharomonospora viridis P101, DSM 43017	Actinobacteria	Actinobacteria	Actinomycetales	Pseudonocardiaceae	Saccharomonospora	Saccharomonospora viridis	4308349	3962
2540341184	Saccharothrix espanaensis DSM 44229	Actinobacteria	Actinobacteria	Actinomycetales	Pseudonocardiaceae	Saccharothrix	Saccharothrix espanaensis	9360653	8495
646564581	Thermobispora bispora R51, DSM 43833	Actinobacteria	Actinobacteria	Actinomycetales	Pseudonocardiaceae	Thermobispora	Thermobispora bispora	4189976	3661
646564566	Segniliparus rotundus CDC 1076, DSM 44985	Actinobacteria	Actinobacteria	Actinomycetales	Segniliparaceae	Segniliparus	Segniliparus rotundus	3157527	3126
2511231086	Kitasatospora setae KM-6054, NBRC 14216	Actinobacteria	Actinobacteria	Actinomycetales	Streptomycetaceae	Kitasatospora	Kitasatospora setae	8783278	7669
2579778836	Streptomyces albulus NK660	Actinobacteria	Actinobacteria	Actinomycetales	Streptomycetaceae	Streptomyces	Streptomyces albulus	9372401	8238
2541047081	Streptomyces albus J1074	Actinobacteria	Actinobacteria	Actinomycetales	Streptomycetaceae	Streptomyces	Streptomyces albus	6841649	5937
646862346	Streptomyces bingchenggensis BCW-1	Actinobacteria	Actinobacteria	Actinomycetales	Streptomycetaceae	Streptomyces	Streptomyces bingchenggensis	11936683	10106
2511231113	Streptomyces cattleya NRRL 8057	Actinobacteria	Actinobacteria	Actinomycetales	Streptomycetaceae	Streptomyces	Streptomyces cattleya	8092553	7585
2511231200	Streptomyces cattleya DSM 46488	Actinobacteria	Actinobacteria	Actinomycetales	Streptomycetaceae	Streptomyces	Streptomyces cattleya	8095515	7650
637000305	Streptomyces coelicolor A3(2)	Actinobacteria	Actinobacteria	Actinomycetales	Streptomycetaceae	Streptomyces	Streptomyces coelicolor	9054847	8325
2554235367	Streptomyces collinus Tu 365	Actinobacteria	Actinobacteria	Actinomycetales	Streptomycetaceae	Streptomyces	Streptomyces collinus	8377286	7205
2561511188	Streptomyces davawensis JCM 4913	Actinobacteria	Actinobacteria	Actinomycetales	Streptomycetaceae	Streptomyces	Streptomyces davawensis	9555950	8697
2561511169	Streptomyces hygroscopicus jinggangensis 5008	Actinobacteria	Actinobacteria	Actinomycetales	Streptomycetaceae	Streptomyces	Streptomyces hygroscopicus	10383684	9194
2561511180	Streptomyces hygroscopicus jinggangensis TL01	Actinobacteria	Actinobacteria	Actinomycetales	Streptomycetaceae	Streptomyces	Streptomyces hygroscopicus	10077952	8964
2597490182	Streptomyces lividans TK24	Actinobacteria	Actinobacteria	Actinomycetales	Streptomycetaceae	Streptomyces	Streptomyces lividans	8345283	7510
2563366593	Streptomyces rapamycinicus NRRL 5491	Actinobacteria	Actinobacteria	Actinomycetales	Streptomycetaceae	Streptomyces	Streptomyces rapamycinicus	12700734	10144
646564576	Streptomyces scabiei 87.22	Actinobacteria	Actinobacteria	Actinomycetales	Streptomycetaceae	Streptomyces	Streptomyces scabiei	10148695	8841
2561511190	Streptomyces sp. PAMC26508	Actinobacteria	Actinobacteria	Actinomycetales	Streptomycetaceae	Streptomyces	Streptomyces sp. PAMC26508	7630245	7158
2523533511	Streptomyces sp. Sv. ACTE SirexAA-E	Actinobacteria	Actinobacteria	Actinomycetales	Streptomycetaceae	Streptomyces	Streptomyces sp. SirexAA-E	7414440	6647
651285011	Streptomyces sp. Tu6071	Actinobacteria	Actinobacteria	Actinomycetales	Streptomycetaceae	Streptomyces	Streptomyces sp. Tu6071	7506727	6733
2524023215	Streptomyces venezuelae Shinobu 719, ATCC 10712	Actinobacteria	Actinobacteria	Actinomycetales	Streptomycetaceae	Streptomyces	Streptomyces venezuelae	8226158	7541
648276750	Streptomyces violaceusniger Tu 4113	Actinobacteria	Actinobacteria	Actinomycetales	Streptomycetaceae	Streptomyces	Streptomyces violaceusniger	10988130	9557
646311958	Streptosporangium roseum NI 9100, DSM 43021	Actinobacteria	Actinobacteria	Actinomycetales	Streptosporangiaceae	Streptosporangium	Streptosporangium roseum	10369518	9510
646311963	Thermomonospora curvata DSM 43183	Actinobacteria	Actinobacteria	Actinomycetales	Thermomonosporaceae	Thermomonospora	Thermomonospora curvata	5639016	5061
646564587	Tsukamurella paurometabola 33, DSM 20162	Actinobacteria	Actinobacteria	Actinomycetales	Tsukamurellaceae	Tsukamurella	Tsukamurella paurometabola	4479724	4391
2623620689	Corynebacterium diphtheriae bv. Gravis (re-annotation)	Actinobacteria	Actinobacteria	Corynebacteriales	Corynebacteriaceae	Corynebacterium	Corynebacterium diphtheriae	2488635	2376
2623620807	Corynebacterium jeikeium K411 (re-annotation)	Actinobacteria	Actinobacteria	Corynebacteriales	Corynebacteriaceae	Corynebacterium	Corynebacterium jeikeium	2476822	2209
2623620944	Mycobacterium avium paratuberculosis K-10 (re-annotation)	Actinobacteria	Actinobacteria	Corynebacteriales	Mycobacteriaceae	Mycobacterium	Mycobacterium avium	4829781	4612
2623620717	Mycobacterium bovis AF 2122/97 (re-annotation)	Actinobacteria	Actinobacteria	Corynebacteriales	Mycobacteriaceae	Mycobacterium	Mycobacterium bovis	4345492	4077
2623620718	Mycobacterium leprae TN (re-annotation)	Actinobacteria	Actinobacteria	Corynebacteriales	Mycobacteriaceae	Mycobacterium	Mycobacterium leprae	3268203	4042
2623620945	Mycobacterium tuberculosis CDC1551 (re-annotation)	Actinobacteria	Actinobacteria	Corynebacteriales	Mycobacteriaceae	Mycobacterium	Mycobacterium tuberculosis	4403837	4149
2623620946	Mycobacterium tuberculosis H37Rv (re-annotation)	Actinobacteria	Actinobacteria	Corynebacteriales	Mycobacteriaceae	Mycobacterium	Mycobacterium tuberculosis	4411532	4147
2623620955	Nocardia farcinica IFM 10152 (re-annotation)	Actinobacteria	Actinobacteria	Corynebacteriales	Nocardiaceae	Nocardia	Nocardia farcinica	6292344	6046
2623620637	Rhodococcus jostii RHA1 (re-annotation)	Actinobacteria	Actinobacteria	Corynebacteriales	Nocardiaceae	Rhodococcus	Rhodococcus jostii	9702737	9137
2623620699	Frankia alni ACN14a (re-annotation)	Actinobacteria	Actinobacteria	Frankiales	Frankiaceae	Frankia	Frankia alni	7497934	6133
650377905	Arthrobacter phenanthrenivorans Sphe3	Actinobacteria	Actinobacteria	Micrococcales	Micrococcaceae	Arthrobacter	Arthrobacter phenanthrenivorans	4535320	4273
2623620975	Streptomyces coelicolor A3(2) (re-annotation)	Actinobacteria	Actinobacteria	Streptomycetales	Streptomycetaceae	Streptomyces	Streptomyces coelicolor	9054847	8254
