# Supplementary material for: Physiological Studies of Chlorobiaceae Suggest that Bacillithiol Derivatives Are the Most Widespread Thiols in Bacteria
Source: mBio. 2018 Nov 27;9(6):e01603-18. doi: 10.1128/mBio.01603-18 (PMC6282198; doi:10.1128/mBio.01603-18)
Supplement: TABLE S3 [file mbo006184195st3.pdf]

Table S3. Strains, plasmids, and primers used in this study.

Strain	Genotype/Description	Antibiotic /Supplement	Source or reference
Cba. tepidum			
WT2321	Plating strain derivative of *Cba. tepidum* TLS1	NA	13
ΔCT1419	Deletion of *bshB* ortholog CT1419	NA	This study
ΔCT1040	Deletion of gene CT1040, encoding a putative SAM-methyltransferase	NA	This study
ΔCT1213	Deletion of gene CT1213, encoding a putative SAM-methyltransferase	NA	This study
E. coli			
DH5α	F^- endA1 glnV44 thi-1 recA1 relA1 gyrA96 deoR nupG Φ80d*lacZ*ΔM15 Δ(*lacZYA-argF*)U169, hsdR17(r_K^- m_K^+), λ–		
β-2155	Conjugal mating strain for plasmid replicons requiring the λ-pir protein.	DAP 30 μg ml^{-1}	23

Plasmids	Genotype/Description	Antibiotic /Supplement	Source or reference
pKO2.0-SmSp	Conjugally mobile counter-selectable suicide vector for *Cba. tepidum* modified from pKO2.0 by replacement of gentamycin resistance marker in pKO2.0 with the Ω fragment from pHP45Ω. Confers resistance to spectinomycin plus streptomycin and sensitivity to sucrose when integrated in the *Cba. tepidum* genome.	*Cba. tepidum*<br>Sm + Sp 100 μg ml^{-1}<br>Sucrose 10 % w/v	This study
pKO2.0	Conjugally mobile counter-selectable suicide vector for *Shewanella oneidensis*.	*E. coli*<br>Gm 100 μg ml^{-1}	23
pHP45Ω	Source of Ω fragment used in pKO2.0-SmSp	*E. coli*<br>Sm + Sp 25 μg ml^{-1}	24

Primer	Sequence 5'-3'	Description
CT1419_5'FlnkFor	CTCGAGGTCGACGGTATCGAT AAGCTTGATGCCCGTGATTGAT AAGGATG	Amplification and fusion of regions spanning 1,329,386-1,329,742 and 1,330,509-

CT1419_5’FlnkRev	GGGTTGGCCCTCTAGATATTAAGCGACGCGGTTTTC	1,330,898 in the *Cba. tepidum* genome in pKO2.0-SmSp for the deletion of CT1419.
CT1419_3’FlnkFor	TCGCTTAATATCTAGAGGGCCAACCCATCAATAGAA	
CT1419_3’FlnkRev	AGTGGATCCCCCGGGCTGCAGGAATTCGATAAACTGAAGTCCTTCGAGGTGAG	
CT1040_5’FlnkFor	CTCGAGGTCGACGGTATCGATAAGCTTGATTGCCAAATGGAAACAAAGTG	Amplification and fusion of regions spanning 978,045-978,405 and 979,205-979,556 in the *Cba. tepidum* genome in pKO2.0-SmSp for the deletion of CT1040.
CT1040_5’FlnkRev	TAGCTGTTGGTCTAGAGAGATGAGACCAGGCAAAAA	
CT1040_3’FlnkFor	GTCTCATCTCTCTAGACCAACAGCTATTTCATTCCTCT	
CT1040_3’FlnkRev	AGTGGATCCCCCGGGCTGCAGGAATTCGATGGCATCAAGCTGTTGAACG	
CT1213_5’FlnkFor	CTCGAGGTCGACGGTATCGATAAGCTTGATAGAGATCTGCATATCCATCCA	Amplification and fusion of regions spanning 1,138,320-1,138,683 and 1,139,910-1,140,250 in the *Cba. tepidum* genome in pKO2.0-SmSp for the deletion of CT1213.
CT1213_5’FlnkRev	ATTTTGTCTATTCTAGAACTCCCGGTTTCATGTTTTG	
CT1213_3’FlnkFor	AACCGGGAGTTCTAGAATAGACAAAATCTGTGACATGACC	
CT1213_3’FlnkRev	AGTGGATCCCCCGGGCTGCAGGAATTCGATATGATATGATAAAAGCGTTCGTGA	
