# Supplementary figures and images for: Physiological Studies of Chlorobiaceae Suggest that Bacillithiol Derivatives Are the Most Widespread Thiols in Bacteria
Source: mBio. 2018 Nov 27;9(6):e01603-18. doi: 10.1128/mBio.01603-18 (PMC6282198; doi:10.1128/mBio.01603-18)

775 **Figure S5.** Synthesis routes for the analytical standards N-Me-BSmB (A) and hCys-BSmB (B).

776 **A)**

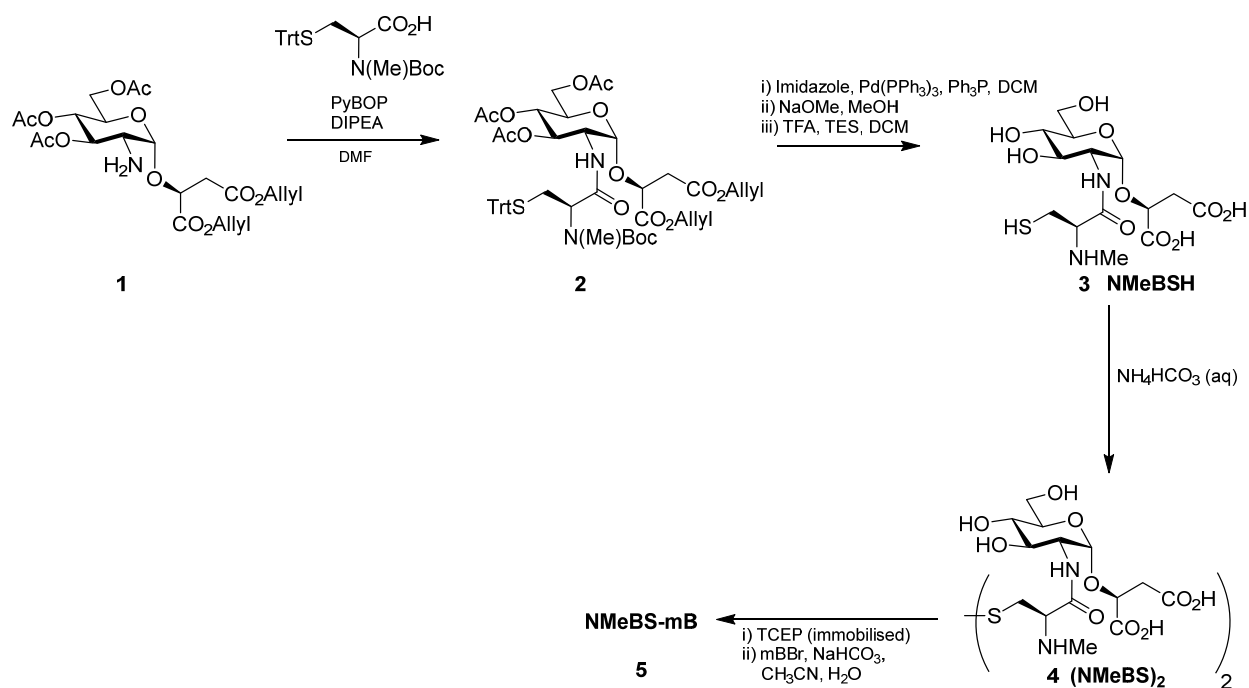

777

778 **B)**

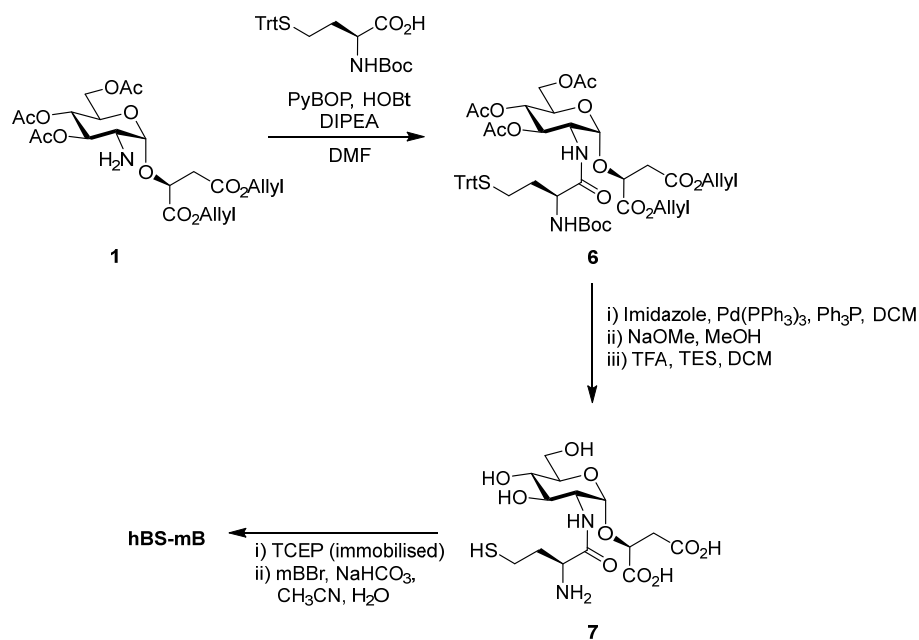

779

Supplement: FIG S5 [file mbo006184195sf5.pdf]
